# Halogenated Reagents in the Ugi Reaction: From Rearrangement Reactions to Ketal Synthesis

**DOI:** 10.1021/acsomega.6c03196

**Published:** 2026-05-05

**Authors:** Beatriz González-Saiz, Carlos Cámara-Herrero, Sandra Díaz-Cabrera, Israel Carreira-Barral, Roberto Quesada, María García-Valverde

**Affiliations:** a Departamento de Química, Facultad de Ciencias, 16725 Universidad de Burgos, Burgos 09001, Spain; b Digital Department of Biomedical and Health Sciences, Faculty of Biomedical and Health Sciences, Universidad Europea de Madrid, Madrid 28670, Spain; c International Research Center in Critical Raw Materials for Advanced Industrial Technologies (ICCRAM), Centro de I+D+I, Universidad de Burgos, Burgos 09001, Spain

## Abstract

The morpholine scaffold constitutes a privileged structural motif in medicinal chemistry, although access to conformationally restricted systems remains challenging. Herein, we report a simple and efficient Ugi/postcondensation strategy that, for the first time, combines two aliphatic halogenated reagents with arylglyoxals to afford fused and bridged morpholines bearing ketal functionalities in a diastereoselective manner. These derivatives serve as precursors to 4-hydroxypyroglutamic acid derivatives, highlighting the synthetic versatility of the approach and providing rapid entry to structurally complex heterocyclic derivatives.

## Graphical abstract

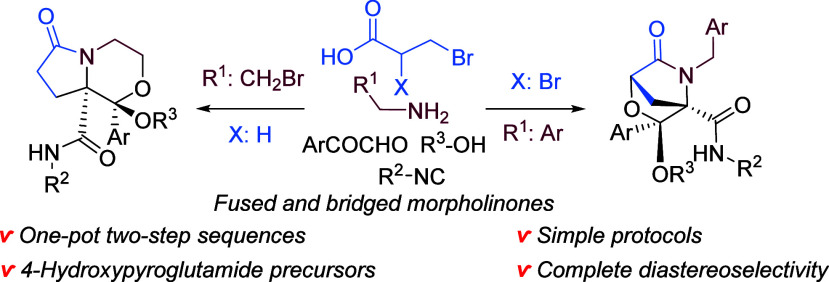

## Introduction

The identification of common structural motifs in bioactive compounds represents a key strategy for the development of new drugs. In this context, the morpholine scaffold is frequently found in therapeutically relevant molecules and plays an important role in the pharmaceutical industry.^1^ Medicinal chemistry aims to enhance the potency and selectivity of such systems through different strategies, with the introduction of conformational restriction being one of the most widely employed,^2^ through the introduction of bridgehead carbons^3^ or the formation of intramolecular hydrogen bonds.^4^ These modifications can improve receptor interactions while modulating physicochemical and pharmacokinetic properties. Some examples include the improved selectivity achieved in the mTOR inhibitor PQR309 through morpholine core modification^5^ as well as the use of acetals and ketals to stabilize favorable conformations,^6^ as demonstrated in the development of aprepitant.^7^ However, the synthesis of conformationally restricted morpholines often involves lengthy and inefficient routes, highlighting the need for new and more efficient synthetic methodologies.^8^

Ugi/postcondensation sequences employing functionalized reagents have proven to be simple and efficient strategies for the synthesis of complex nitrogen-containing heterocycles.^9^ Although halogenated substrates have been extensively explored,^10^ methodologies that exploit two halogenated positions within the starting materials remain scarce. In fact, most of the reported examples involve halogens located on aromatic rings;^11^ however, no examples combining two aliphatic halogenated positions have been described, highlighting a significant gap in the current synthetic methodology. Thus, we envisaged the possibility of exploring the scope of Ugi/postcondensation sequences, seizing the huge potential of halogenated reagents in the construction of highly functionalized heterocycles.

## Results and Discussion

Initially, we conducted a study combining two halogenated reagents, namely, the acid and the amine. For this purpose, we selected 3-bromopropionic acid **2**a and 2-bromoethylamine **1**a as starting materials. The Ugi reaction was subsequently performed under standard conditions, with stirring a mixture of the four components in methanol at room temperature for 24 h. As expected, the Ugi adduct was not isolated; instead, a pyrrolidinone was obtained as a result of a spontaneous cyclization through a selective nucleophilic substitution at the alkyl bromide fragment of the propionamide chain (Table 1).^12^ In addition, Passerini and Ugi (Ugi-OMe) adducts, with the bromine on the amine being replaced by a methoxy group in the case of the latter, were also formed in the reactions, accounting for the moderate yields of derivatives **5** (see ESI, Scheme S1, Figures S1 and S2).

**Table 1:**
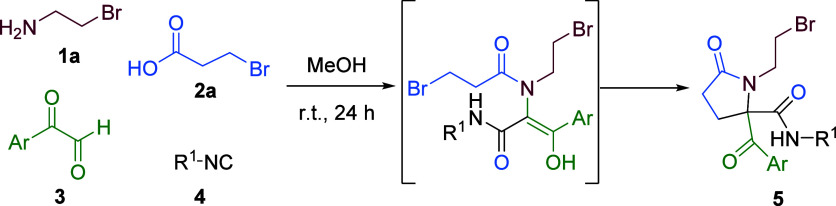
Synthesis of *N*-(2-Bromoethyl)­pyrrolidin-2-ones from 3-Bromopropionic Acid and 2-Bromoethylamine

**entry**	**3** (Ar)	**4** (R^1^)	**5** (%)^a^
1	**3a** (C_6_H_5_)	**4a** (*c*C_6_H_11_)	**5a** (43)^b^
2	**3b** (4-CH_3_OC_6_H_4_)	**4a** (*c*C_6_H_11_)	**5b** (34)^b^
3	**3a** (C_6_H_5_)	**4b** (C(CH_3_)_3_)	**5c** (49)^b^
4	**3c** (4-FC_6_H_4_)	**4b** (C(CH_3_)_3_)	**5d** (52)^b^

a Yield was obtained after purification.

b Passerini and substituted Ugi (Ugi-OMe) adducts were identified as byproducts.

Subsequently, the corresponding pyrrolidinones **5** were treated with cesium carbonate in acetonitrile, aiming to generate pyrrolopiperazin-2-ones through an intramolecular *N*-alkylation reaction, a procedure similar to that used for the synthesis of pyrrolopiperazin-2,6-diones from aminoester derivatives.^13^ Unexpectedly, benzoate derivatives **6** were the only products observed, resulting from the substitution of the bromine atom, accompanied by acyl migration. The structures of these rearranged compounds could be confirmed by single-crystal X-ray diffraction analysis of **6b**. Moreover, the slow evaporation of a solution of this pyrrolidinone in an *n*-butanol/methanol mixture afforded a conglomerate, allowing the spontaneous resolution of enantiomers^14^ (Table 2).

**Table 2:**
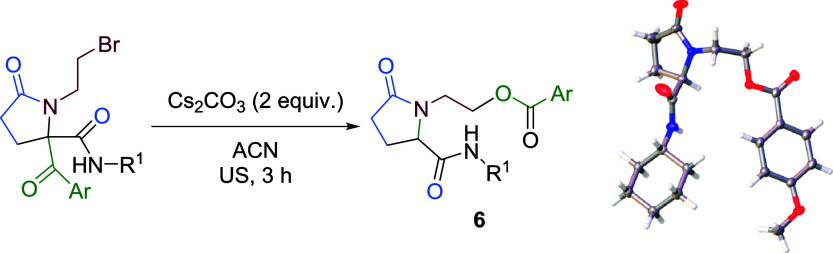
Rearrangement Reaction of Pyrrolidinones **5** and X-ray Molecular Structure of Enantiomer (*R*)-**6b** ^b^

**entry**	**5** (Ar, R^1^)	**6** (%)^a^
1	**5a** (C_6_H_5_, *c*C_6_H_11_)	**6a** (88)
2	**5b** (4-CH_3_OC_6_H_4_, *c*C_6_H_11_)	**6b** (90)
3	**5c** (C_6_H_5_, C(CH_3_)_3_)	**6c** (87)
4	**5d** (4-FC_6_H_4_, C(CH_3_)_3_)	**6d** (97)

a Yield obtained after purification.

b The OLEX2 plot is at the 50% probability level.

A plausible mechanism can be proposed, starting with the nucleophilic attack of a hydroxide anion on the acyl group of **5**, yielding intermediate **A**, which undergoes rapid intramolecular nucleophilic substitution to afford cyclic hemiketal **B**, which is unstable under basic conditions. Although cleavage of this intermediate would lead to alkoxide **E**, which, upon protonation, could afford the corresponding alcohol **7**, through a substitution reaction involving neighboring group participation,^15^ the presence of the amide group prevents this pathway. Consequently, enolate formation and subsequent acyl migration are favored, affording intermediate **D**. In order to confirm this mechanism, we carried out the reaction using 2-aminoethanol instead of 2-bromoethylamine. As expected, treatment of the corresponding *N*-(2-hydroxyethyl)­pyrrolidin-2-one **7** with cesium carbonate led to similar results (see ESI, Scheme S2), probably through an intramolecular reaction from the alkoxide intermediate **E** followed by a retro-Claisen-type condensation (Scheme 1).

**Scheme 1:**
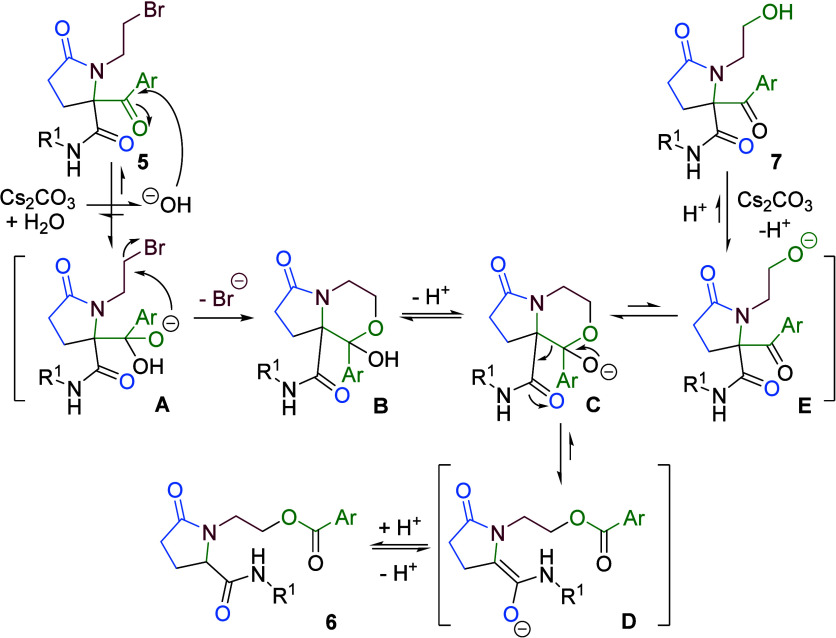
Proposed Mechanism for the Rearrangement Reaction

Taking into account this mechanism, we decided to replace wet acetonitrile by alcohols as the solvent for the reactions involving bromo derivatives **5**, with the goal of forming ketals, which are stable under basic conditions rather than the unstable hemiketals **B**. In this way, treatment of bromoethylamine derivative **5** with cesium carbonate using alcohols as solvents afforded different fused heterocycles (Table 3). The outcome of the reaction depends on steric factors such as the nature of the alcohol (R^2^OH) and the amide substituent (R^1^). Thus, smaller alkoxides (Table 3, entries 3 vs 1) and bulkier R^1^ substituents (Table 3, entries 2 vs 4, or 3 vs 5) favor the formation of the expected ketals **9** over pyrrolopiperazines **8**, the latter being formed through an amide deprotonation, *N-*alkylation, and retro-Claisen sequence. Interestingly, the ketals were obtained as single (1R*,8aR*) diastereomers, as determined by single-crystal X-ray diffraction of **9e**. Moreover, this structure shows an intramolecular hydrogen bond between the NH of the secondary amide and the morpholine oxygen, which restricts the conformation and stabilizes the ketal group. In turn, ketals could be synthesized in a one-pot strategy without isolation of the corresponding pyrrolidinone **5** by carrying out the Ugi reaction in the appropriate alcohol, followed by treatment of the reaction mixture with cesium carbonate.

**Table 3:**
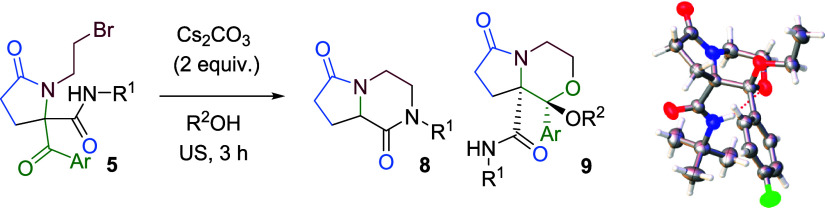
Synthesis of Ketals from Pyrrolidinones **5** and X-ray Molecular Structure of Pyrrolomorpholine **9e** ^e^

**entry**	**5** (Ar, R^1^)	**solvent**	8:9^a^	**8** (%)^b^	**9** (%)^b^	**9** d.r.^a^
1	**5a** (C_6_H_5_, *c*C_6_H_11_)	nBuOH	>97:3	**8a** (50)	-^c^	
2	**5a** (C_6_H_5_, *c*C_6_H_11_)	EtOH	50:50	**8a** (30)	**9a** (22)	>95:5^d^
3	**5a** (C_6_H_5_, *c*C_6_H_11_)	MeOH	33:67	**8a** (25)	**9b** (38)	>95:5^d^
4	**5c** (C_6_H_5_, C(CH_3_)_3_)	EtOH	10:90	**8b** ^c^	**9c** (70)	>95:5^d^
5	**5c** (C_6_H_5_, C(CH_3_)_3_)	MeOH	6:94	**8b** ^c^	**9d** (75)	>95:5^d^
6	**5d** (4-FC_6_H_4_, C(CH_3_)_3_)	EtOH	8:92	**8b** ^c^	**9e** (75)	>95:5^d^
7	**5d** (4-FC_6_H_4_, C(CH_3_)_3_)	MeOH	7:93	**8b** ^c^	**9f** (85)	>95:5^d^

a Determined by ^1^H NMR of the reaction mixture.

b Yield obtained after purification.

c Not isolated.

d Relative configuration of the observed diastereomer (1R*,8aR*).

e The OLEX2 plot is at the 50% probability level.

Encouraged by these results, we explored the synthesis of more complex heterocycles using a doubly halogenated carboxylic acid. In this way, we carried out the Ugi reaction using 2,3-dibromopropionic acid, benzyl amine, phenylglyoxal, and cyclohexyl isocyanide, expecting the spontaneous formation of a 3-bromopyrrolidinone. However, the major product was the Ugi adduct **10**, obtained as a mixture of diastereomers (Scheme 2). Treatment of this Ugi adduct with a base selectively afforded two different heterocycles depending on the base employed. Thus, sonication of the Ugi adduct with triethylamine in methanol for 1 h yielded the 3-bromopyrrolidinone **11a** in high yield but with poor diastereoselectivity, whereas sonication with cesium carbonate in methanol afforded the bridged bicyclic morpholine **12a** in nearly quantitative yield and with complete diastereoselectivity (Scheme 2).

**Scheme 2:**
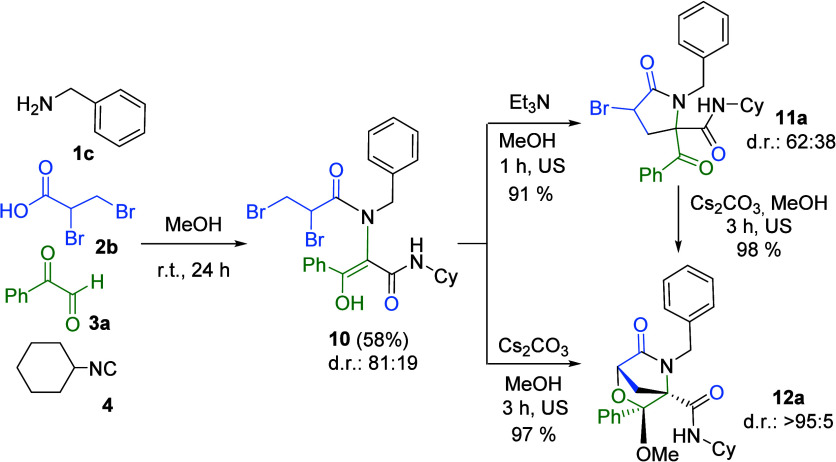
Synthesis of 3-Bromopyrrolidinone **11a** and Fused Pyrrolidinone **12a**

Interestingly, treatment of 3-bromopyrrolidinone **11a** (dr 62:38) with cesium carbonate in methanol quantitatively yielded the bicyclic morpholinone **12a** as a single (1R*,3R*,4R*) diastereomer (Scheme 2). This chemical and stereochemical outcome can be explained by the epimerization of 3-bromopyrrolydin-2-one **11a** at the C3 position, favored by the acidity at this position, with the (3R*,5S*) epimer allowing bimolecular nucleophilic substitution (Scheme 3).

**Scheme 3:**
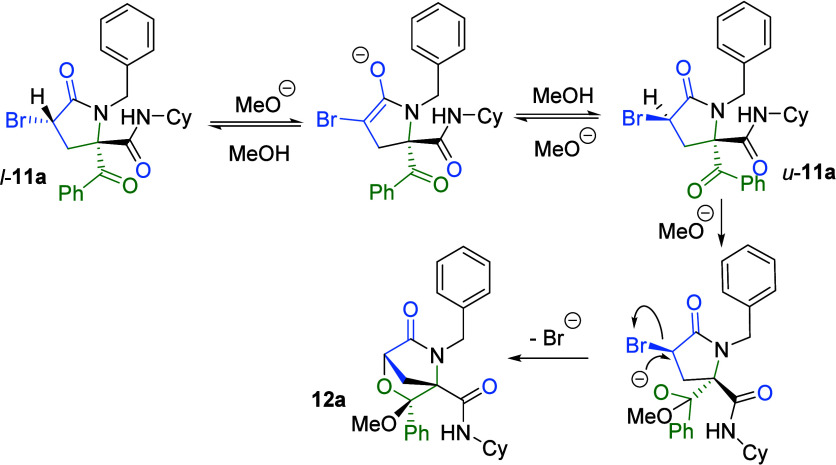
Proposed Mechanism for the Synthesis of Bicyclic Morpholinone **12a**

On the basis of these findings, we attempted to develop one-pot protocols for the synthesis of these heterocycles. Thus, for the preparation of 3-bromopyrrolidin-2-ones **11**, the Ugi reaction was carried out under standard conditions by stirring the four reactants in methanol at room temperature for 24 h, followed by treatment of the reaction mixture with triethylamine. This protocol was successfully applied to a range of amines, arylglyoxals, and isocyanides (Scheme 4).

**Scheme 4:**
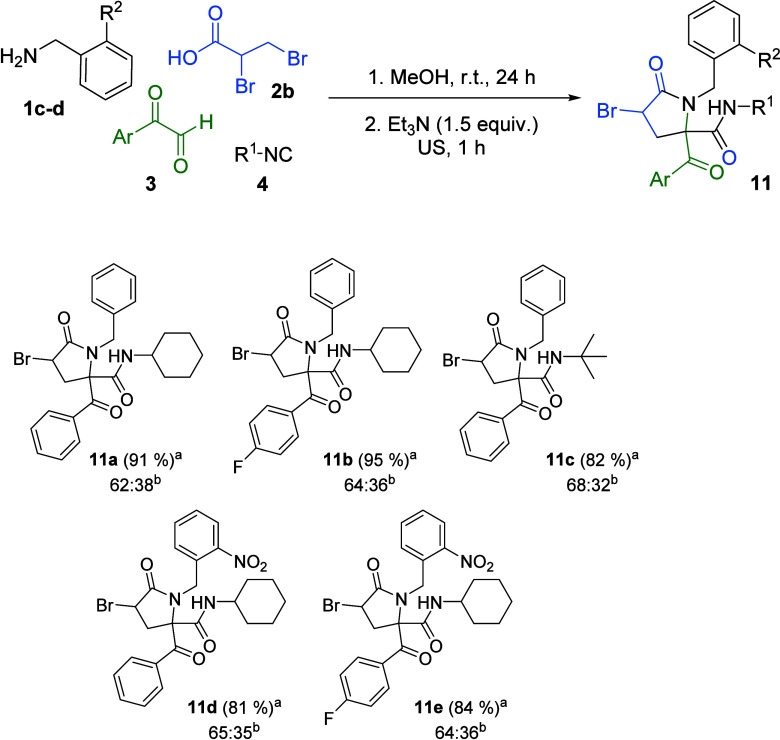
One-Step Synthesis of 3-Bromopyrrolidin-2-ones **11**

a Yield obtained after purification (as a mixture of diastereoisomers).
b Determined by ^1^H NMR of the reaction mixture.

For the selective synthesis of bicyclic morpholinones **12**, a one-pot strategy was also developed using the appropriate alcohol for ketal formation as the solvent for the Ugi reaction. The structure and relative configuration of bicyclic morpholinones were determined by single-crystal X-ray diffraction analysis of **12g**. In this case, the structure shows an intramolecular hydrogen bond between the amide at C4 and the methoxy oxygen (Scheme 5).

**Scheme 5:**
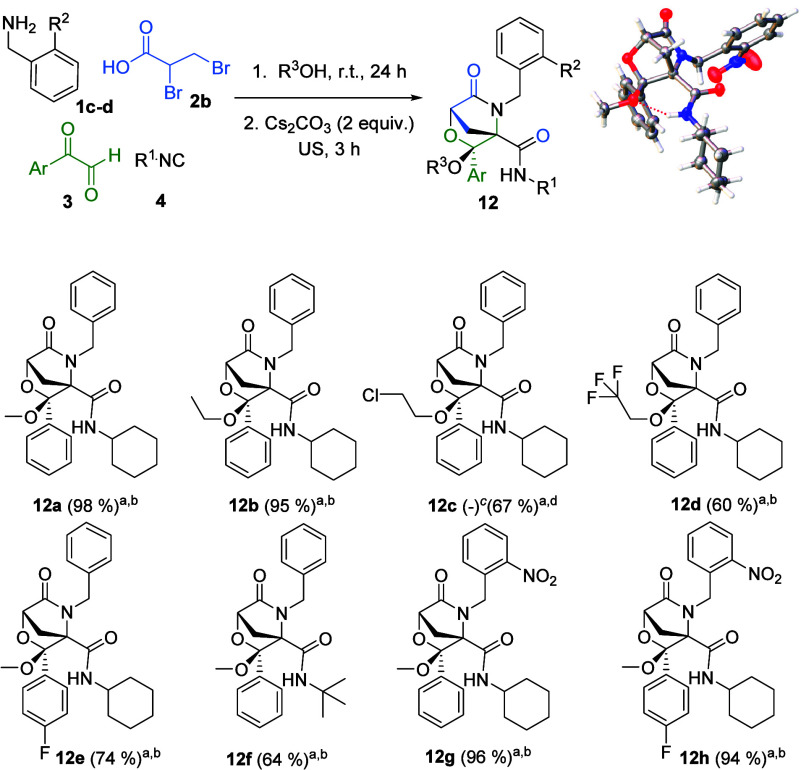
Synthesis of Bicyclic Morpholinones **12** and X-ray Molecular Structure of Bicyclic Morpholinone **12g** ^e^

a Yield was obtained after purification.
b Single diastereomer· observed by ^1^H NMR of the reaction mixture. Relative configuration for the observed diastereomer (1R*,3R*, and 4R*).
c The bicyclic morpholinone was not identified when the complete sequence was performed in 2-chloroethanol.
d The Ugi reaction was performed in methanol and cyclization in 2-chloroethanol.
e The OLEX2 plot is at the 50% probability level.

This approach was unsuccessful in the case of 2-chloroethanol. Therefore, a one-pot, two-step strategy was adopted. First, the Ugi reaction was conducted in methanol, and then the solvent was removed. The crude Ugi adduct was next dissolved in 2-chloroethanol and treated with cesium carbonate, yielding the corresponding ketal after purification.

In addition to the interest of these structures, the complete diastereoselectivity observed in the synthesis of bicyclic morpholinones, together with the presence of a ketal moiety, prompted us to envisage the synthesis of 2-aroyl-4-hydroxypyroglutaramides with a controlled relative configuration. Thus, the bicyclic morpholinones were treated with a hydrochloric acid aqueous solution (1 M) in boiling THF for 16 h, affording highly substituted pyroglutaramide derivatives **13** as single (3R*,5R*) stereoisomers. Single crystals of one of these derivatives, **13a**, were grown and analyzed by X-ray diffraction (Scheme 6).

**Scheme 6:**
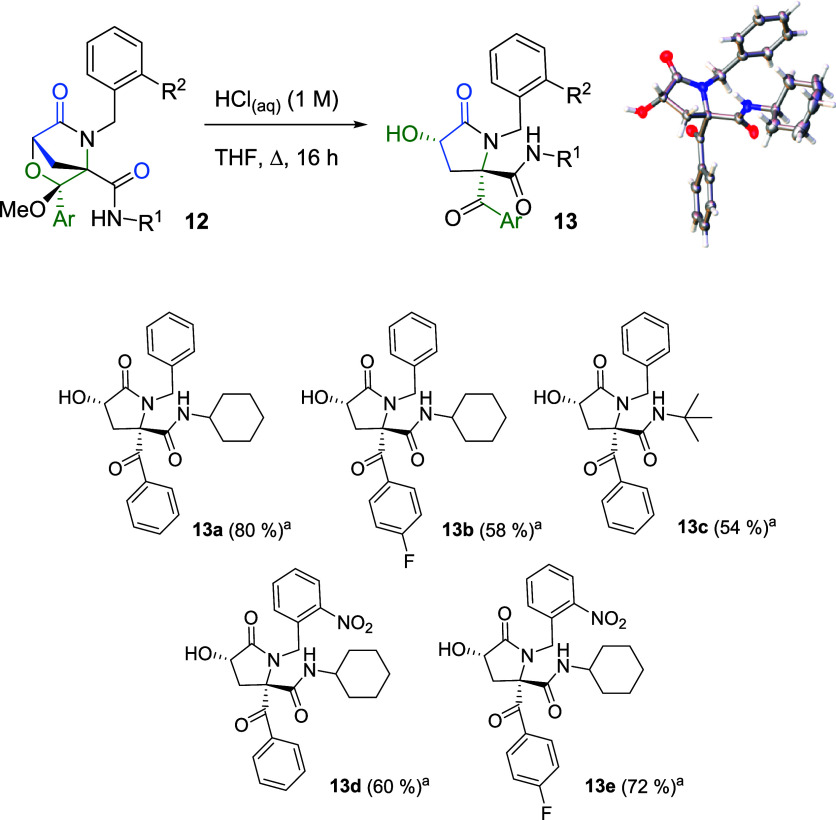
Synthesis of 3-Hydroxypyrrolidinones and X-ray Molecular Structure of **13a** ^c^

a Yield obtained after purification.
b A single diastereomer was observed by ^1^H NMR of the reaction mixture. Relative configuration for the observed diastereomer (3R*,5R*).
c The OLEX2 plot is at the 50% probability level.

On the basis of these results, we decided to synthesize novel pyrroloquinazolines related to natural products with interesting biological activities such as linarinic acid^16^ and vasicine.^17^ In this way, we reduced the nitro group in ketal derivatives **12g,h** using stannous chloride under acidic conditions in refluxing methanol,^18^ yielding pyrroloquinazolines **14**. Subsequent hydrolysis of ketals with hydrochloric acid afforded 1-carbamoyl-3-hydroxypyrroloquinazoline derivatives **15** (Scheme 7).

**Scheme 7:**
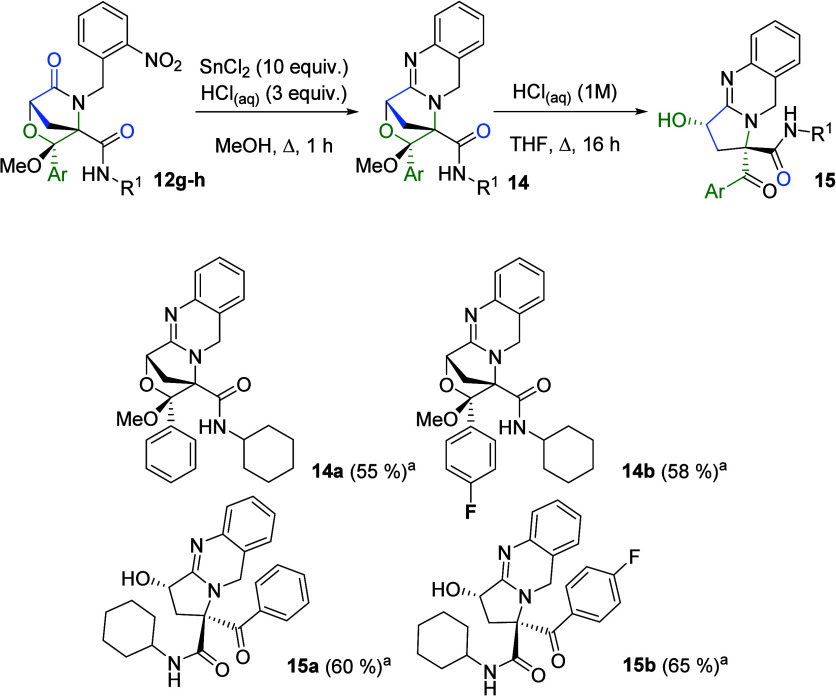
Synthesis of Pyrroloquinazolines **14** and **15**

a Yield obtained after purification.
b A single diastereomer was observed by ^1^H NMR of the reaction mixture. Relative configuration (1R*,3R*,4R*) for the observed diastereomer of **14** and (1R*,3R*) for **15**.

## Conclusions

In this work, we have demonstrated the potential of combining two halogenated reagents with arylglyoxals in the Ugi reaction as an efficient strategy for furnishing a range of highly substituted heterocycles, in many cases with high diastereoselectivity. This approach enables the straightforward synthesis of structurally distinctive bicyclic and bridged morpholinones bearing ketal functionalities stabilized by intramolecular hydrogen bonding. Furthermore, the outcome of base-mediated transformations of brominated pyrrolidinones was found to be highly dependent on the reaction medium. Finally, bridged morpholinone ketals provide access to 4-hydroxypyroglutamic acid derivatives in a diastereoselective manner.

## Experimental Section

### General Information

Reagents were used as purchased. Flash column chromatography was performed using silica gel (SCHlab silica, 230–400 mesh ASTM). Melting points were measured with a Gallenkamp Melting Point apparatus, are given in Celsius degrees, and are not corrected. ^1^H, ^13^C, and DEPT-135 NMR spectra were recorded on a Varian Mercury 300 MHz at 298 K using CDCl_3_. The recorded spectra were referenced to the remaining resonance signals of the deuterated solvent. Coupling constants (*J*) are denoted in Hz and chemical shifts (δ) in ppm. Multiplicities are denoted as follows: s = singlet, d = doublet, t = triplet, q = quartet, m = multiplet. Chemical shifts are given in ppm relative to TMS (0.0 ppm). High-resolution mass spectra (HRMS) were recorded on a 6545 Q-TOF Agilent LC-MS mass spectrometer (positive electrospray ionization mode (ESI+)). Three-dimensional X-ray data were collected on a Bruker D8 VENTURE diffractometer.

### Synthesis of *N-*(2-Bromoethyl)­pyrrolidin-2-ones (**5**)

2-Bromoethylamine hydrobromide **1a** (2.0 mmol, 1.0 equiv) was treated with potassium hydroxide (1.8 mmol, 0.9 equiv) in methanol (10 mL), and the resulting suspension was sonicated for 10 min. Subsequently, 3-bromopropionic acid **2a** (2.0 mmol, 1.0 equiv) was added to the reaction mixture, followed by arylglyoxal hydrate **3** (2.0 mmol, 1.0 equiv) and isocyanide **4** (2.0 mmol, 1.0 equiv). The reaction mixture was stirred at room temperature for 24 h, and after removal of the solvent under reduced pressure, the residue was dissolved in chloroform. The resulting solution was washed with a hydrochloric acid aqueous solution (1 M) and then with a sodium carbonate aqueous solution (1 M). The organic layer was dried over sodium sulfate, filtered, and concentrated to dryness. The crude was purified by column chromatography, employing SiO_2_ as the stationary phase and a hexane/ethyl acetate mixture as the eluent.

#### 5-Benzoyl-1-(2-bromoethyl)-5-(*N*-cyclohexylcarbamoyl)-2-pyrrolidinone (**5a**)

Yellow oil (43%, 362 mg) (3:1 Hex/EtOAc). ^1^H NMR (300 MHz, CDCl_3_) δ: 7.83 (d, *J* = 7.1 Hz, 2H), 7.59 (t, *J* = 7.4 Hz, 1H), 7.46 (t, *J* = 7.7 Hz, 2H), 6.26 (d, *J* = 8.0 Hz, 1H, NH), 3.98–3.76 (m, 2H), 3.62–3.23 (m, 3H), 3.17–2.92 (m, 1H), 2.70–2.52 (m, 1H), 2.45–2.25 (m, 2H), 1.96–0.90 (m, 10H). ^13^C­{^1^H} NMR (75 MHz, CDCl_3_) δ: 193.8 (C_q_), 175.9 (C_q_), 167.1 (C_q_), 134.3 (CH), 133.4 (C_q_), 129.4 (CH), 129.0 (CH), 77.4 (C_q_), 49.7 (CH), 45.4 (CH_2_), 32.7 (CH_2_), 32.3 (CH_2_), 29.0 (CH_2_), 28.9 (CH_2_), 27.5 (CH_2_), 25.4 (CH_2_), 24.9 (CH_2_), 24.8 (CH_2_). HRMS (ESI+) *m*/*z*: [M + H]^+^ calculated for C_20_H_26_BrN_2_O_3_ 421.1121; found 421.1125.

#### 1-(2-Bromoethyl)-5-(*N*-cyclohexylcarbamoyl)-5-(4-methoxybenzoyl)-2-pyrrolidinone (**5b**)

Dark yellow oil (34%, 307 mg) (3:1 Hex/EtOAc). ^1^H NMR (300 MHz, CDCl_3_) δ: 7.83 (d, *J* = 9.0 Hz, 2H), 6.93 (d, *J* = 9.0 Hz, 2H), 5.93 (d, *J* = 8.1 Hz, 1H, NH), 3.89 (s, 3H), 3.87–3.77 (m, 2H), 3.63–3.50 (m, 1H), 3.46–3.28 (m, 2H), 3.04–2.91 (m, 1H), 2.68–2.51 (m, 1H), 2.43–2.25 (m, 2H), 1.84–1.52 (m, 5H), 1.23–1.06 (m, 5H). ^13^C­{^1^H} NMR (75 MHz, CDCl_3_) δ: 176.02 (C_q_), 167.5 (C_q_), 164.4 (C_q_), 132.4 (C_q_), 132.0 (CH), 125.9 (C_q_), 114.2 (CH), 77.2 (C_q_), 55.8 (CH_3_), 49.7 (CH), 45.4 (CH_2_), 32.7 (CH_2_), 32.4 (CH_2_), 29.8 (CH_2_), 29.0 (CH_2_), 27.5 (CH_2_), 25.4 (CH_2_), 24.9 (CH_2_), 24.8 (CH_2_). HRMS (ESI+) *m*/*z*: [M + H]^+^ calculated for C_21_H_28_BrN_2_O_4_ 451.1227; found 451.1233.

#### 5-Benzoyl-1-(2-bromoethyl)-5-(*N*-*tert*-butylcarbamoyl)-2-pyrrolidinone (**5c**)

Yellow oil (49%, 387 mg) (3:1 Hex/EtOAc). ^1^H NMR (300 MHz, CDCl_3_) δ: 7.82 (d, *J* = 7.4 Hz, 2H), 7.60 (t, *J* = 7.4 Hz, 1H), 7.52–7.40 (m, 2H), 5.90 (s, 1H, NH), 3.87 (ddd, *J* = 13.2, 11.4, 4.9 Hz, 1H), 3.59–3.48 (m, 1H), 3.43–3.27 (m, 2H), 3.02–2.82 (m, 1H), 2.65–2.47 (m, 1H), 2.41–2.13 (m, 2H), 1.30 (s, 9H). ^13^C­{^1^H} NMR (75 MHz, CDCl_3_) δ: 196.7 (C_q_), 176.0 (C_q_), 167.0 (C_q_), 134.2 (CH), 133.3 (C_q_), 129.3 (CH), 128.9 (CH), 78.0 (C_q_), 53.0 (C_q_), 45.1 (CH_2_), 28.8 (CH_2_), 28.7 (CH_2_), 28.3 (CH_3_), 27.4 (CH_2_). HRMS (ESI+) *m*/*z*: [M + Na]^+^ calculated for C_18_H_23_BrN_2_NaO_3_ 417.0784; found 417.0793.

#### 1-(2-Bromoethyl)-5-(*N*-*tert*-butylcarbamoyl)-5-(4-fluorobenzoyl)-2-pyrrolidinone (**5d**)

White solid (52%, 430 mg) (3:1 Hex/EtOAc). M.p.: 147–153 °C. ^1^H NMR (300 MHz, CDCl_3_) δ: 7.89 (dd, *J* = 9.0, 5.0 Hz, 2H), 7.16 (dd, *J* = 9.0, 8.1 Hz, 2H), 5.81 (s, 1H, NH), 3.89 (ddd, *J* = 15.2, 12.0, 5.3 Hz, 1H), 3.59 (ddd, *J* = 10.8, 9.5, 5.3 Hz, 1H), 3.46–3.31 (m, 2H), 2.91 (td, *J* = 9.9, 3.7 Hz, 1H), 2.58 (dd, *J* = 12.7, 10.0 Hz, 1H), 2.45–2.19 (m, 2H), 1.33 (s, 9H). ^13^C­{^1^H} NMR (75 MHz, CDCl_3_) δ: 195.4 (C_q_), 175.7 (C_q_), 167.0 (C_q_), 132.3 (d,^3^ *J* = 9.3 Hz, CH), 129.8 (C_q_), 116.3 (d,^2^ *J* = 21.9 Hz, CH), 77.8 (C_q_), 53.1 (C_q_), 45.4 (CH_2_), 28.9 (CH_2_), 28.4 (CH_3_), 27.5 (CH_2_). HRMS (ESI+) *m*/*z*: [M + H]^+^ calculated for C_18_H_23_BrFN_2_O_3_ 413.0871; found 413.0882.

#### Synthesis of 5-(*N*-Cyclohexylcarbamoyl)-1-(2-hydroxyethyl)-5-(4-methoxybenzoyl)-2-pyrrolidinone (**7**)

A solution of ethanolamine **1b** (2.0 mmol, 1.0 equiv) in dry methanol (10 mL) was treated with 3-bromopropionic acid 2a (2.0 mmol, 1.0 equiv), followed by 4-methoxyphenylglyoxal **3b** (2.0 mmol, 1.0 equiv) and cyclohexyl isocyanide 4a (2.0 mmol, 1.0 equiv). The reaction mixture was stirred at room temperature for 24 h, and after removing the solvent under reduced pressure, the residue was dissolved in chloroform. The resulting solution was washed with a hydrochloric acid aqueous solution (1 M) and then with a sodium carbonate aqueous solution (1 M). The organic layer was dried over sodium sulfate, filtered, and concentrated to dryness. The crude was purified by column chromatography, employing SiO_2_ as the stationary phase and a hexane/ethyl acetate mixture as eluent. Orange oil (52% yield, 404 mg) (1:1 Hex/EtOAc). ^1^H NMR (300 MHz, CDCl_3_) δ: 7.76 (d, *J* = 9.0 Hz, 2H), 7.41 (d, *J* = 8.1 Hz, 1H, NH), 6.86 (d, *J* = 9.0 Hz, 2H), 4.57–4.35 (m, 1H, OH), 3.81 (s, 3H), 3.76–3.59 (m, 3H), 3.56–3.30 (m, 2H), 2.99–2.70 (m, 1H), 2.59–2.39 (m, 1H), 2.39–2.23 (m, 2H), 1.90–0.94 (m, 10H). **^13^** C­{**^1^** H} NMR (75 MHz, CDCl_3_) δ: 195.3 (C_q_), 177.7 (C_q_), 167.7 (C_q_), 164.0 (C_q_), 131.7 (CH), 126.4 (C_q_), 113.9 (CH), 77.6 (C_q_), 61.0 (CH_2_), 55.6 (CH_3_), 49.4 (CH), 46.0 (CH_2_), 32.5 (CH_2_), 32.1 (CH_2_), 29.5 (CH_2_), 29.1 (CH_2_), 25.3 (CH_2_), 24.8 (CH_2_), 24.7 (CH_2_). HRMS (ESI+) *m*/*z*: [M + H]^+^ calculated for C_21_H_29_N_2_O_5_ 389.2066; found 389.2078.

### Synthesis of *N*-(2-Aroyloxyethyl)­pyrrolidin-2-ones (**6**)

#### Method A: Synthesis of *N*-(2-aroyloxyethyl)­pyrrolidin-2-ones **6** Starting from *N*-(2-Bromoethyl)­pyrrolidin-2-ones **5**

A mixture of 2-pyrrolidinone **5** (1.0 mmol, 1.0 equiv) and cesium carbonate (2.0 mmol, 2.0 equiv) in wet acetonitrile (5 mL) was stirred under sonication for 3 h. Subsequently, the solvent was removed under reduced pressure, and the residue was dissolved in chloroform. The resulting solution was washed with a hydrochloric acid aqueous solution (1 M), and the organic layer was dried over sodium sulfate, filtered, and concentrated to dryness under reduced pressure. The crude was purified by column chromatography, employing SiO_2_ as the stationary phase and a hexane/ethyl acetate mixture as eluent, or by precipitation, using a dichloromethane/ether mixture. Single crystals of **6b**, suitable for X-ray diffraction analysis, were grown by slow evaporation of a solution of the compound in an *n*-butanol/methanol mixture.

#### Method B: Synthesis of *N*-(2-Aroyloxyethyl)­pyrrolidin-2-ones **6b** Starting from *N*-(2-Hydroxyethyl)­pyrrolidin-2-ones **7**

A mixture of 2-pyrrolidinones **7** (1.0 mmol, 1.0 equiv) and cesium carbonate (2.0 mmol, 2.0 equiv) in acetonitrile (5 mL) was stirred under reflux for 1 h. Subsequently, the solvent was removed under reduced pressure, and the residue was dissolved in chloroform. Henceforth, the crude was treated as indicated in method A.

#### 5-(*N*-Cyclohexylcarbamoyl)-2-(2-oxopyrrolidin-1-yl)­ethyl Benzoate (**6a**)

Yellow oil (88%, 315 mg, method A) (3:1 Hex/EtOAc). ^1^H NMR (300 MHz, CDCl_3_) δ: ^1^H NMR (300 MHz, CDCl_3_) δ 8.01 (d, *J* = 8.2 Hz, 2H), 7.57 (t, *J* = 7.4 Hz, 1H), 7.44 (t, *J* = 7.8 Hz, 2H), 5.93 (d, *J* = 8.4 Hz, 1H, NH), 4.54 (ddd, *J* = 11.0, 6.4, 4.1 Hz, 1H), 4.36 (ddd, *J* = 11.2, 6.7, 3.9 Hz, 1H), 4.18 (dd, *J* = 8.4, 3.5 Hz, 1H), 4.09 (ddd, *J* = 14.8, 6.5, 4.0 Hz, 1H), 3.83–3.67 (m, 1H), 3.29 (ddd, *J* = 14.7, 7.0, 3.8 Hz, 1H), 2.60–2.39 (m, 1H), 2.43–2.25 (m, 2H), 2.04 (ddd, *J* = 13.2, 8.3, 3.5 Hz, 1H), 1.92–0.99 (m, 10H). ^13^C­{^1^H} NMR (75 MHz, CDCl_3_) δ: 176.4 (C_q_), 170.2 (C_q_), 166.5 (C_q_), 133.5 (C_q_), 129.8 (CH), 129.7 (CH), 128.7 (CH), 62.3 (CH_2_), 62.2 (CH), 48.7 (CH), 41.2 (CH_2_), 33.0 (CH_2_), 29.7 (CH_2_), 25.4 (CH_2_), 25.0 (CH_2_), 24.1 (CH_2_). HRMS (ESI+) *m*/*z*: [M + H]^+^ calculated for C_20_H_27_N_2_O_4_ 359.1965; found 359.1974.

#### 5-(*N*-Cyclohexylcarbamoyl)-2-(2-oxopyrrolidin-1-yl)­ethyl 4-methoxybenzoate (**6b**)

White solid (90%, 350 mg, method A), (93%, 360 mg, method B) (3:1 Hex/EtOAc or purification by precipitation in a DCM/ether mixture). M.p.: 135–137 °C. ^1^H NMR (300 MHz, CDCl_3_) δ: 7.94 (d, *J* = 8.7 Hz, 2H), 6.90 (d, *J* = 8.9 Hz, 2H), 6.05 (d, *J* = 8.3 Hz, 1H, NH), 4.48 (ddd, *J* = 10.6, 6.5, 3.8 Hz, 1H), 4.30 (ddd, *J* = 11.0, 6.9, 4.1 Hz, 1H), 4.17 (dd, *J* = 8.7, 3.8 Hz, 1H), 4.05 (ddd, *J* = 14.9, 6.6, 3.9 Hz, 1H), 3.83 (s, 3H), 3.80–3.68 (m, 1H), 3.24 (ddd, *J* = 14.8, 6.8, 4.0 Hz, 1H), 2.57–2.38 (m, 1H), 2.39–2.20 (m, 2H), 2.02 (ddd, *J* = 11.9, 8.3, 3.6 Hz, 1H), 1.90–0.97 (m, 10H). ^13^C­{^1^H} NMR (75 MHz, CDCl_3_) δ: 176.3 (C_q_), 170.2 (C_q_), 166.2 (C_q_), 163.8 (C_q_), 131.8 (CH), 122.1 (C_q_), 113.9 (CH), 62.2 (CH), 61.9 (CH_2_), 55.6 (CH_3_), 48.6 (CH), 41.2 (CH_2_), 33.1 (CH_2_), 29.7 (CH_2_), 25.5 (CH_2_), 25.0 (CH_2_), 24.1 (CH_2_). HRMS (ESI+) *m*/*z*: [M + H]^+^ calculated for C_21_H_29_N_2_O_5_ 389.2071; found 389.2080.

#### 5-(*N*-*tert*-Butylcarbamoyl)-2-(2-oxopyrrolidin-1-yl)­ethyl Benzoate (**6c**)

Yellow oil (87%, 289 mg, method A) (3:1 Hex/EtOAc). ^1^H NMR (300 MHz, CDCl_3_) δ: 8.08–7.99 (m, 2H), 7.57 (t, *J* = 7.4 Hz, 1H), 7.45 (t, *J* = 7.4 Hz, 2H), 5.57 (s, 1H, NH), 4.55 (ddd, *J* = 11.0, 6.9, 4.1 Hz, 1H), 4.34 (ddd, *J* = 11.4, 7.0, 4.2 Hz, 1H), 4.13 (dd, *J* = 6.5, 4.3 Hz, 1H), 4.09 (ddd, *J* = 14.8, 6.9, 4.2 Hz, 1H), 3.27 (ddd, *J* = 14.8, 7.0, 4.1 Hz, 1H), 2.60–2.44 (m, 1H), 2.40–2.23 (m, 2H), 2.13–1.93 (m, 1H), 1.31 (s, 9H). ^13^C­{^1^H} NMR (75 MHz, CDCl_3_) δ: 176.4 (C_q_), 170.5 (C_q_), 166.5 (C_q_), 133.5 (CH), 130.2 (C_q_), 129.8 (CH), 128.7 (CH), 62.7 (CH), 62.4 (CH_2_), 51.9 (C_q_), 41.1 (CH_2_), 29.8 (CH_2_), 28.8 (CH_3_), 23.9 (CH_2_). HRMS (ESI+) *m*/*z*: [M + Na]^+^ calculated for C_18_H_24_N_2_NaO_4_ 355.1628; found 355.1631.

#### 5-(*N*-*tert*-Butylcarbamoyl)-2-(2-oxopyrrolidin-1-yl)­ethyl 4-Fluorobenzoate (**6d**)

Yellow oil (97%, 340 mg, method A) (3:1 Hex/EtOAc). ^1^H NMR (300 MHz, CDCl_3_) δ: 8.16–7.90 (m, 2H), 7.20–6.97 (m, 2H), 5.79 (s, 1H, NH), 4.51 (ddd, *J* = 11.3, 7.0, 4.2 Hz, 1H), 4.31 (ddd, *J* = 11.3, 6.5, 4.3 Hz, 1H), 4.10 (dd, *J* = 7.6, 4.1 Hz, 1H), 4.12–4.02 (m, 1H), 3.28 (ddd, *J* = 14.9, 6.5, 4.0 Hz, 1H), 2.55–2.45 (m, 1H), 2.40–2.20 (m, 2H), 2.07–1.88 (m, 1H), 1.32 (s, 9H). ^13^C­{^1^H} NMR (75 MHz, CDCl_3_) δ: 176.6 (C_q_), 170.5 (C_q_), 167.7 (C_q_), 166.0 (d,^1^ *J* = 255.6 Hz, C_q_), 132.4 (d,^3^ *J* = 9.3 Hz, CH), 126.0 (d,^4^ *J* = 2.9 Hz, C_q_), 115.8 (d,^2^ *J* = 22.0 Hz, CH), 62.6 (CH), 62.1 (CH_2_), 51.9 (C_q_), 41.0 (CH_2_), 29.6 (CH_2_), 28.7 (CH_3_), 23.9 (CH_2_). HRMS (ESI+) *m*/*z*: [M + H]^+^ calculated for C_18_H_24_FN_2_O_4_ 351.1715; found 351.1725.

### Synthesis of Pyrrolopiperazine **8** and Pyrrolomorpholine Ketals **9**

A mixture of 2-pyrrolidinone **5** (1.0 mmol, 1.0 equiv) and cesium carbonate (2.0 mmol, 2.0 equiv) in the corresponding alcohol (10 mL) was stirred under sonication for 3 h. Subsequently, the solvent was removed under reduced pressure, and the residue was dissolved in chloroform. The resulting solution was washed with a hydrochloric acid aqueous solution (1 M), and the organic layer was dried over sodium sulfate, filtered, and concentrated to dryness under reduced pressure. The crude was purified by column chromatography, employing SiO_2_ as the stationary phase and a hexane/ethyl acetate mixture as eluent, or by precipitation using a chloroform/ether mixture or a hexane/ethyl acetate mixture. Single crystals of **9e**, suitable for X-ray diffraction analysis, were grown by the slow evaporation of a solution of the compound in a hexane/chloroform mixture.

#### 2-Cyclohexyltetrahydropyrrolo­[1,2-*a*]­pyrazine-1,6­(2*H*,7*H*)-dione (**8**)

Yellow oil (50% from **5a** and *n*-BuOH, 116 mg) (3:1 Hex/EtOAc). ^1^H NMR (300 MHz, CDCl_3_) δ: 4.43 (tt, *J* = 11.5, 3.8 Hz, 1H), 4.16 (t, *J* = 7.6 Hz, 1H), 3.95 (dt, *J* = 12.7, 4.9 Hz, 1H), 3.41–3.28 (m, 2H), 3.23 (dt, *J* = 12.8, 4.7 Hz, 1H), 2.57–2.32 (m, 3H), 2.27–1.92 (m, 1H), 1.86–1.17 (m, 10H). ^13^C­{^1^H} NMR (75 MHz, CDCl_3_) δ: 173.6 (C_q_), 168.3 (C_q_), 57.7 (CH), 52.5 (CH), 40.1 (CH_2_), 38.0 (CH_2_), 30.9 (CH_2_), 30.0 (CH_2_), 29.9 (CH_2_), 25.7 (CH_2_), 25.6 (CH_2_), 25.5 (CH_2_), 22.5 (CH_2_). HRMS (ESI+) *m*/*z*: [M + H]^+^ calculated for C_13_H_21_N_2_O_2_ 237.1598; found 237.1602.

#### (1R*,8aR*)-8a-(*N*-Cyclohexylcarbamoyl)-1-ethoxy-1-phenylhexahydro-6*H*-pyrrolo­[2,1-*c*]­[1,4]­oxazin-6-one (**9a**)

Yellow oil (22%, 85 mg) (3:1 Hex/EtOAc). ^1^H NMR (300 MHz, CDCl_3_) δ: 7.51 (s, 2H), 7.42–7.31 (m, 3H), 5.83 (d, *J* = 8.1 Hz, 1H, NH), 4.03 (dd, *J* = 12.7, 3.3 Hz, 1H), 3.99–3.88 (m, 2H), 3.57–3.37 (m, 2H), 3.23 (dq, *J* = 9.6, 7.1 Hz, 1H), 2.98 (dq, *J* = 9.6, 7.1 Hz, 1H), 2.92–2.77 (m, 1H), 2.71–2.32 (m, 3H), 1.72–1.20 (m, 5H), 1.37–1.21 (m, 3H), 1.09 (t, *J* = 7.1 Hz, 3H), 0.99–0.67 (m, 2H). ^13^C­{^1^H} NMR (75 MHz, CDCl_3_) δ: 175.0 (C_q_), 167.2 (C_q_), 137.7 (C_q_), 129.2 (CH), 128.6 (CH), 99.0 (C_q_), 72.0 (C_q_), 58.7 (CH_2_), 57.8 (CH_2_), 48.3 (CH), 37.1 (CH_2_), 32.9 (CH_2_), 32.3 (CH_2_), 30.4 (CH_2_), 25.9 (CH_2_), 25.6 (CH_2_), 24.8 (CH_2_), 24.7 (CH_2_), 14.8 (CH_3_). HRMS (ESI+) *m*/*z*: [M + H]^+^ calculated for C_22_H_31_N_2_O_4_ 387.2278; found 387.2281.

#### (1R*,8aR*)-8a-(*N*-Cyclohexylcarbamoyl)-1-methoxy-1-phenylhexahydro-6*H*-pyrrolo­[2,1-*c*]­[1,4]­oxazin-6-one (**9b**)

Yellow oil (38%, 142 mg) (3:1 Hex/EtOAc). ^1^H NMR (300 MHz, CDCl_3_) δ: 7.51 (s, 2H), 7.48–7.34 (m, 3H), 5.76 (d, *J* = 8.0 Hz, 1H, NH), 4.04 (dd, *J* = 13.2, 2.0 Hz, 1H), 3.92 (dd, *J* = 9.1, 3.0 Hz, 2H), 3.55–3.34 (m, 2H), 2.93 (s, 3H), 2.85–2.67 (m, 1H), 2.60–2.36 (m, 3H), 1.72–1.02 (m, 10H). ^13^C­{^1^H} NMR (75 MHz, CDCl_3_) δ: 174.9 (C_q_), 167.2 (C_q_), 137.0 (C_q_), 129.3 (CH), 128.6 (CH), 99.2 (C_q_), 72.0 (C_q_), 58.7 (CH_3_), 49.6 (CH_2_), 48.4 (CH), 37.0 (CH_2_), 32.9 (CH_2_), 32.3 (CH_2_), 30.3 (CH_2_), 25.8 (CH_2_), 25.6 (CH_2_), 24.8 (CH_2_), 24.7 (CH_2_). HRMS (ESI+) *m*/*z*: [M + H]^+^ calculated for C_21_H_29_N_2_O_4_ 373.2122; found 373.2141.

#### (1R*,8aR*)-8a-(*N*-*tert*-Butylcarbamoyl)-1-ethoxy-1-phenylhexahydro-6*H*-pyrrolo­[2,1-*c*]­[1,4]­oxazin-6-one (**9c**)

Pale yellow oil (70%, 252 mg) (3:1 Hex/EtOAc). ^1^H NMR (300 MHz, CDCl_3_) δ: 7.61–7.33 (m, 5H), 5.75 (s, 1H, NH), 4.03 (dd, *J* = 12.9, 3.2 Hz, 1H), 3.98–3.86 (m, 2H), 3.48 (ddd, *J* = 12.2, 10.5, 6.2 Hz, 1H), 3.23 (dq, *J* = 9.5, 7.1 Hz, 1H), 2.97 (dq, *J* = 9.5, 7.1 Hz, 1H), 2.88–2.72 (m, 1H), 2.63–2.33 (m, 3H), 1.09 (t, *J* = 7.1 Hz, 3H), 0.98 (s, 9H). ^13^C­{^1^H} NMR (75 MHz, CDCl_3_) δ: 175.0 (C_q_), 167.2 (C_q_), 137.9 (C_q_), 129.2 (CH), 128.6 (CH), 99.0 (C_q_), 72.5 (C_q_), 58.8 (CH_2_), 57.8 (CH_2_), 51.2 (C_q_), 37.0 (CH_2_), 30.4 (CH_2_), 28.3 (CH_3_), 25.9 (CH_2_), 14.8 (CH_3_). HRMS (ESI+) *m*/*z*: [M + Na]^+^ calculated for C_20_H_28_N_2_NaO_4_ 383.1941; found 383.1964.

#### (1R*,8aR*)-8a-(*N*-*tert*-Butylcarbamoyl)-1-methoxy-1-phenylhexahydro-6*H*-pyrrolo­[2,1-*c*]­[1,4]­oxazin-6-one (**9d**)

Yellow oil (75%, 260 mg) (3:1 Hex/EtOAc). ^1^H NMR (300 MHz, CDCl_3_) δ: 7.66–7.30 (m, 5H), 5.69 (s, 1H, NH), 4.04 (dd, *J* = 13.4, 2.8 Hz, 1H), 3.94–3.89 (m, 2H), 3.50 (dt, *J* = 13.9, 9.0 Hz, 1H), 2.93 (s, 3H), 2.84–2.69 (m, 1H), 2.62–2.35 (m, 3H), 0.99 (s, 9H). ^13^C­{^1^H} NMR (75 MHz, CDCl_3_) δ: 174.9 (C_q_), 167.2 (C_q_), 137.2 (C_q_), 129.3 (CH), 128.7 (CH), 99.2 (C_q_), 72.5 (C_q_), 58.7 (CH_2_), 51.3 (C_q_), 49.7 (CH_3_), 37.0 (CH_2_), 30.3 (CH_2_), 28.3 (CH_3_), 25.8 (CH_2_). HRMS (ESI+) *m*/*z*: [MH + H]^+^ calculated for C_19_H_28_N_2_O_4_ 348.2044; found 348.2025.

#### (1R*,8aR*)-8a-(*N*-*tert*-Butylcarbamoyl)-1-ethoxy-1-(4-fluorophenyl)­hexahydro-6*H*-pyrrolo­[2,1-*c*]­[1,4]­oxazin-6-one (**9e**)

Pale yellow solid (75%, 284 mg) (3:1 Hex/EtOAc). M.p.: 157–159 °C. ^1^H NMR (300 MHz, CDCl_3_) δ: 7.64–7.07 (m, 4H), 5.91 (s, 1H, NH), 4.10–3.98 (m, 1H), 4.04 (dd, *J* = 12.1, 3.4 Hz, 1H), 3.51–3.31 (m, 2H), 3.21 (dq, *J* = 9.1, 7.4 Hz, 1H), 3.02–2.85 (m, 1H), 2.77–2.32 (m, 4H), 1.08 (t, *J* = 7.0 Hz, 3H), 1.02 (s, 9H). ^13^C­{^1^H} NMR (75 MHz, CDCl_3_) δ: 175.0 (C_q_), 166.7 (C_q_), 163.0 (d,^1^ *J* = 249.3 Hz, C_q_), 133.8 (d,^4^ *J* = 3.4 Hz, C_q_), 128.6 (d,^2^ *J* = 40.2 Hz, CH), 115.5 (d,^3^ *J* = 21.5 Hz, CH), 98.6 (C_q_), 72.3 (C_q_), 58.8 (CH_2_), 57.8 (CH_2_), 51.3 (C_q_), 36.9 (CH_2_), 30.4 (CH_2_), 28.4 (CH_3_), 25.6 (CH_2_), 14.8 (CH_3_). HRMS (ESI+) *m*/*z*: [MH + H]^+^ calculated for C_20_H_29_FN_2_O_4_ 380.2106; found 380.2096.

#### (1R*,8aR*)-8a-(*N*-*tert*-Butylcarbamoyl)-1-(4-fluorophenyl)-1-methoxyhexahydro-6*H*-pyrrolo­[2,1-*c*]­[1,4]­oxazin-6-one (**9f**)

Yellow oil (85%, 310 mg) (3:1 Hex/EtOAc). ^1^H NMR (300 MHz, CDCl_3_) δ: 7.50–6.99 (m, 4H), 5.86 (s, 1H, NH), 4.04 (dd, *J* = 12.9, 4.1 Hz, 1H), 3.93–3.87 (m, 2H), 3.62–3.29 (m, 1H), 2.92 (s, 3H), 2.81–2.28 (m, 4H), 1.03 (s, 9H).^13^C­{^1^H} NMR (75 MHz, CDCl_3_) δ: 174.8 (C_q_), 166.9 (C_q_), 133.1 (d,^4^ *J* = 4.0 Hz, C_q_), 129.1 (d,^3^ *J* = 12.1 Hz, CH), 115.6 (d,^2^ *J* = 21.3 Hz, CH), 99.0 (C_q_), 72.3 (C_q_), 58.8 (CH_2_), 51.4 (C_q_), 49.6 (CH_3_), 36.9 (CH_2_), 30.3 (CH_2_), 28.4 (CH_3_), 25.5 (CH_2_). HRMS (ESI+) *m*/*z*: [M + H]^+^ calculated for C_19_H_26_FN_2_O_4_ 365.1871; found 365.1881.

### Synthesis of (*E*)-2-(*N*-Benzyl-2,3-dibromopropanamido)-*N*-cyclohexyl-3-hydroxy-3-phenylacrylamide **10**

A solution of benzylamine **1c** (2.0 mmol, 1.0 equiv) in methanol (10 mL) was treated with 2,3-dibromopropionic acid **2b** (2.0 mmol, 1.0 equiv), followed by phenylglyoxal hydrate **3a** (2.0 mmol, 1.0 equiv) and cyclohexyl isocyanide **4a** (2.0 mmol, 1.0 equiv). The reaction mixture was stirred at room temperature for 24 h, and the obtained precipitate was isolated by vacuum filtration, washed with cold methanol, and dried *in vacuo*. The Ugi adduct **10** was purified by recrystallization using a chloroform/ether mixture. Pink solid (58%, 655 mg). M.p.: 138–140 °C. ^1^H NMR (300 MHz, CDCl_3_) δ: 16.03 (s, 1H, OH), 7.86 (dd, *J* = 7.0, 2.9 Hz, 2H), 7.52–7.28 (m, 8H), 5.77 (d, *J* = 13.8 Hz, 1H), 5.25 (d, *J* = 7.6 Hz, 1H, NH), 4.54 (dd, *J* = 11.5, 3.9 Hz, 1H), 4.18 (dd, *J* = 11.5, 9.3 Hz, 1H), 3.69 (dd, *J* = 9.3, 4.0 Hz, 1H), 3.43 (d, *J* = 13.7 Hz, 1H), 3.49–3.35 (m, 1H), 1.92–1.04 (m, 10H). ^13^C­{^1^H} NMR (75 MHz, CDCl_3_) δ: 169.9 (C_q_), 169.8 (C_q_), 169.1 (C_q_), 137.5 (C_q_), 132.9 (C_q_), 131.2 (CH), 129.4 (CH), 129.3 (CH), 128.9 (CH), 128.6 (CH), 128.4 (CH), 106.0 (C_q_), 54.6 (CH_2_), 49.2 (CH), 38.7 (CH), 32.3 (CH_2_), 31.6 (CH_2_), 31.5 (CH_2_), 25.3 (CH_2_), 25.0 (CH_2_), 24.9 (CH_2_). HRMS (ESI+) *m*/*z*: [M + Na]^+^ calculated for C_25_H_28_Br_2_N_2_NaO_3_ 585.0364; found 585.0361.

### Synthesis of 3-Bromo-2-pyrrolidinones (**11**)

#### Method A: Synthesis of 3-Bromo-2-pyrrolidinones **11** Starting from Ugi Adducts **10**

A mixture of Ugi adduct **10** (1.0 mmol, 1.0 equiv) and triethylamine (1.5 mmol, 1.5 equiv) in methanol (10 mL) was stirred under sonication for 1 h. Subsequently, the solvent was removed under reduced pressure, and the residue was dissolved in chloroform. The resulting solution was washed with a hydrochloric acid aqueous solution (1 M), and the organic layer was dried over sodium sulfate, filtered, and concentrated to dryness under reduced pressure. The crude was purified by precipitation using a chloroform/ether mixture or a hexane/ethyl acetate mixture. Diastereomers were separated by column chromatography, employing SiO_2_ as the stationary phase and a hexane/ethyl acetate mixture as the eluent.

#### Method B: Synthesis of 3-Bromo-2-pyrrolidinones **11** by a One-Pot Two-Step Sequence

Before use, 2-nitrobenzylamine hydrochloride **1d** (2.0 mmol, 1.0 equiv) was treated with potassium hydroxide (1.8 mmol, 0.9 equiv) in methanol (10 mL) under ultrasonic conditions for 10 min. A solution of the corresponding benzylamine **1c**–**d** (2.0 mmol, 1.0 equiv) in methanol (10 mL) was treated with 2,3-dibromopropionic acid **2b** (2.0 mmol, 1.0 equiv), followed by arylglyoxal hydrate **3** (2.0 mmol, 1.0 equiv) and isocyanide **4** (2.0 mmol, 1.0 equiv). The reaction mixture was stirred at room temperature for 24 h. Then, triethylamine was added (3.0 mmol, 1.5 equiv), and the mixture was stirred under sonication for 1 h. Subsequently, the solvent was removed under reduced pressure, and the residue was dissolved in chloroform. Henceforth, the crude was treated as indicated in method A.

#### 5-Benzoyl-1-benzyl-3-bromo-5-(*N*-cyclohexylcarbamoyl)-2-pyrrolidinone (**11a**)

Yellow foam (91%, 440 mg, method A), (91%, 882 mg, method B) (purification by precipitation in a DCM/ether mixture). M.p.: 173–176 °C (as diastereomer mixture 62:38). ^1^H NMR (300 MHz, CDCl_3_) δ: 7.89–7.03 (m, 10H), 6.30 (d, *J* = 7.6 Hz, 0.38 H, NH minor diast), 5.99 (d, *J* = 7.5 Hz, 0.62H, NH major diast), 5.22 (d, *J* = 16.0 Hz, 0.62H major diast), 5.08 (d, *J* = 16.2 Hz, 0.38H minor diast), 4.78 (dd, *J* = 8.1, 4.8 Hz, 0.38H minor diast), 4.55 (t, *J* = 6.9 Hz, 0.62H major diast), 4.46 (d, *J* = 16.1 Hz, 0.38H minor diast), 4.26 (d, *J* = 16.1 Hz, 0.62H major diast), 3.68 (dd, *J* = 14.9, 8.5 Hz, 0.38H minor diast), 3.40–3.28 (m, 1H), 3.23 (d, *J* = 6.7 Hz, 1.24H major diast), 2.83 (dd, *J* = 14.7, 4.3 Hz, 0.38H minor diast), 1.65–0.59 (m, 10H). ^13^C­{^1^H} NMR (75 MHz, CDCl_3_) δ: 194.5 (C_q_), 194.4 (C_q_), 172.0 (C_q_), 171.9 (C_q_), 166.3 (C_q_), 165.7 (C_q_), 136.8 (C_q_), 135.5 (C_q_), 134.2 (CH), 134.0 (CH), 133.6 (C_q_), 133.5 (C_q_), 129.4 (CH), 129.2 (CH), 128.9 (CH), 128.8 (CH), 128.6 (CH), 128.4 (CH), 127.7 (CH), 127.6 (CH), 127.5 (CH), 127.4 (CH), 75.8 (C_q_), 75.2 (C_q_), 49.7 (CH), 49.4 (CH), 47.8 (CH_2_), 47.1 (CH_2_), 40.3 (CH), 40.1 (CH), 39.9 (CH_2_), 39.0 (CH_2_), 32.0 (CH_2_), 31.8 (CH_2_), 31.5 (CH_2_), 25.3 (CH_2_), 24.6 (CH_2_), 24.5 (CH_2_). HRMS (ESI+) *m*/*z*: [M + H]^+^ calculated for C_25_H_28_BrN_2_O_3_ 483.1278; found 483.1278.

#### (3R*,5S*)-1-Benzyl-3-bromo-5-(*N*-cyclohexylcarbamoyl)-5-(4-fluorobenzoyl)-2-pyrrolidinone (**11b**, diastereomer **1**)

Pale yellow oil (25%, 251 mg, method B) (3:1 Hex/EtOAc). ^1^H NMR (300 MHz, CDCl_3_) δ: 7.90 (dd, *J* = 9.0, 5.3 Hz, 2H), 7.34–7.23 (m, 5H), 7.17 (t, *J* = 8.6 Hz, 2H), 6.29 (d, *J* = 7.5 Hz, 1H, NH), 5.21 (d, *J* = 15.7 Hz, 1H), 4.57 (t, *J* = 4.4 Hz, 1H), 4.32 (d, *J* = 16.2 Hz, 1H), 3.54–3.08 (m, 3H), 1.64–0.85 (m, 10H). ^13^C­{^1^H} NMR (75 MHz, CDCl_3_) δ: 192.9 (C_q_), 171.8 (C_q_), 166.0 (C_q_), 165.9 (d,^1^ *J* = 257.6 Hz, C_q_), 136.7 (C_q_), 132.0 (d,^3^ *J* = 9.5 Hz, CH), 129.8 (d,^4^ *J* = 3.3 Hz, C_q_), 128.5 (CH), 127.4 (CH), 127.4 (CH), 116.0 (d,^2^ *J* = 22.2 Hz, CH), 75.8 (C_q_), 49.5 (CH), 47.0 (CH_2_), 40.1 (CH), 38.7 (CH_2_), 31.8 (CH_2_), 31.4 (CH_2_), 25.2 (CH_2_), 24.7 (CH_2_), 24.6 (CH_2_). HRMS (ESI+) *m*/*z*: [M + H]^+^ calculated for C_25_H_27_BrFN_2_O_3‑_501.1184; found 501.1188.

#### (3R*,5R*)-1-Benzyl-3-bromo-5-(*N*-cyclohexylcarbamoyl)-5-(4-fluorobenzoyl)-2-pyrrolidinone (**11b**, diastereomer **2**)

Pale yellow oil (39%, 391 mg, method B) (3:1 Hex/EtOAc). ^1^H NMR (300 MHz, CDCl_3_) δ: 7.84 (dd, *J* = 9.1, 5.1 Hz, 2H), 7.20–7.02 (m, 7H), 6.39 (d, *J* = 7.6 Hz, 1H, NH), 4.92 (d, *J* = 16.0 Hz, 1H), 4.77 (dd, *J* = 8.6, 4.9 Hz, 1H), 4.47 (d, *J* = 16.0 Hz, 1H), 3.55 (dd, *J* = 14.9, 8.6 Hz, 1H), 3.38 (tdt, *J* = 11.0, 7.7, 3.8 Hz, 1H), 2.79 (dd, *J* = 14.8, 5.0 Hz, 1H), 1.74–1.02 (m, 10H). ^13^C­{^1^H} NMR (75 MHz, CDCl_3_) δ: 195.3 (C_q_), 172.1 (C_q_), 165.9 (C_q_), 166.0 (d,^1^ *J* = 257.9 Hz, C_q_), 135.4 (C_q_), 132.2 (d,^3^ *J* = 9.5 Hz, CH), 130.1 (d,^4^ *J* = 4.7 Hz, C_q_), 128.4 (CH), 127.9 (CH), 127.6 (CH), 116.1 (d,^2^ *J* = 21.9 Hz, CH), 75.0 (C_q_), 49.7 (CH), 47.9 (CH_2_), 40.1 (CH), 39.9 (CH_2_), 32.0 (CH_2_), 31.9 (CH_2_), 25.3 (CH_2_), 24.7 (CH_2_), 24.6 (CH_2_). HRMS (ESI+) *m*/*z*: [M + H]^+^ calculated for C_25_H_27_BrFN_2_O_3‑_501.1184; found 501.1189.

#### (3R*,5S*)-5-Benzoyl-1-benzyl-3-bromo-5-(*N*-*tert*-butylcarbamoyl)-2-pyrrolidinone (**11c**, diastereomer **1**)

Pale yellow oil (12%, 110 mg, method B) (3:1 Hex/EtOAc). ^1^H NMR (300 MHz, CDCl_3_) δ: 7.89–7.79 (m, 2H), 7.59 (t, *J* = 7.4 Hz, 1H), 7.44 (t, *J* = 7.7 Hz, 2H), 7.23–7.04 (m, 5H), 5.94 (s, 1H, NH), 5.11 (d, *J* = 16.4 Hz, 1H), 4.76 (dd, *J* = 8.5, 4.4 Hz, 1H), 4.44 (d, *J* = 16.4 Hz, 1H), 3.69 (dd, *J* = 15.0, 8.5 Hz, 1H), 2.83 (dd, *J* = 14.9, 4.4 Hz, 1H), 0.94 (s, 9H). ^13^C­{^1^H} NMR (75 MHz, CDCl_3_) δ: 194.9 (C_q_), 172.2 (C_q_), 166.1 (C_q_), 136.9 (C_q_), 134.1 (CH), 133.6 (C_q_), 129.3 (CH), 128.8 (CH), 128.7 (CH), 127.5 (CH), 127.3 (CH), 76.6 (C_q_), 52.6 (C_q_), 47.2 (CH_2_), 40.0 (CH), 39.2 (CH_2_), 27.6 (CH_3_). HRMS (ESI+) *m*/*z*: [M + H]^+^ calculated for C_23_H_26_BrN_2_O_3_ 457.1127; found 457.1131.

#### (3R*,5R*)-5-Benzoyl-1-benzyl-3-bromo-5-(*N*-*tert*-butylcarbamoyl)-2-pyrrolidinone (**11c**, diastereomer **2**)

Pale yellow oil (25%, 229 mg, method B) (3:1 Hex/EtOAc). ^1^H NMR (300 MHz, CDCl_3_) δ: 7.83 (d, *J* = 7.4 Hz, 2H), 7.60 (t, *J* = 7.4 Hz, 1H), 7.44 (t, *J* = 7.7 Hz, 2H), 7.24–6.97 (m, 5H), 5.94 (s, 1H, NH), 5.11 (d, *J* = 16.4 Hz, 1H), 4.76 (dd, *J* = 8.5, 4.4 Hz, 1H), 4.44 (d, *J* = 16.4 Hz, 1H), 3.69 (dd, *J* = 14.9, 8.5 Hz, 1H), 2.83 (dd, *J* = 14.8, 4.4 Hz, 1H), 0.94 (s, 9H). ^13^C­{^1^H} NMR (75 MHz, CDCl_3_) δ: 197.3 (C_q_), 172.2 (C_q_), 135.7 (C_q_), 134.3 (CH), 133.7 (C_q_), 129.4 (CH), 129.0 (CH), 128.7 (CH), 127.6 (CH), 127.5 (CH), 75.8 (C_q_), 52.7 (C_q_), 47.9 (CH_2_), 40.2 (CH), 39.9 (CH_2_), 27.8 (CH_3_). HRMS (ESI+) *m*/*z*: [M + H]^+^ calculated for C_23_H_26_BrN_2_O_3_ 457.1127; found 457.1131.

#### (3R*,5S*)-5-Benzoyl-1-(2-nitrobenzyl)-3-bromo-5-(*N*-cyclohexylcarbamoyl)-2-pyrrolidinone (**11d**, diastereomer **1**)

Pale yellow oil (28%, 297 mg, method B) (3:1 Hex/EtOAc). ^1^H NMR (300 MHz, CDCl_3_) δ: 7.96 (dd, *J* = 8.2, 1.7 Hz, 1H), 7.77 (dd, *J* = 8.1, 1.2 Hz, 2H), 7.56 (t, *J* = 7.7 Hz, 2H), 7.43–7.30 (m, 4H), 6.00 (d, *J* = 7.9 Hz, 1H), 5.40 (d, *J* = 18.4 Hz, 1H), 4.81 (d, *J* = 18.4 Hz, 1H), 4.49 (dd, *J* = 9.0, 7.5 Hz, 1H), 3.65 (dd, *J* = 14.7, 7.5 Hz, 1H), 3.37–3.28 (m, 1H), 3.12 (dd, *J* = 14.7, 9.0 Hz, 1H), 1.44–0.47 (m, 5H). ^13^C­{^1^H} NMR (75 MHz, CDCl_3_) δ: 194.0 (C_q_), 172.1 (C_q_), 164.6 (C_q_), 147.3 (C_q_), 134.4 (CH), 133.9 (CH), 132.5 (C_q_), 132.3 (C_q_), 129.1 (CH), 128.8 (CH), 127.8 (CH), 125.0 (CH), 76.3 (C_q_), 49.7 (CH), 44.7 (CH_2_), 39.4 (CH), 38.0 (CH_2_), 31.5 (CH_2_), 31.3 (CH_2_), 25.0 (CH_2_), 24.5 (CH_2_). HRMS (ESI+) *m*/*z*: [M + H]^+^ calculated for C_25_H_27_BrN_3_O_5‑_528.1129; found 528.1132.

#### (3R*,5R*)-5-Benzoyl-1-(2-nitrobenzyl)-3-bromo-5-(*N*-cyclohexylcarbamoyl)-2-pyrrolidinone (**11d**, diastereomer **2**)

Pale yellow oil (19%, 200 mg, method B) (3:1 Hex/EtOAc). ^1^H NMR (300 MHz, CDCl_3_) δ: 7.95 (dd, *J* = 8.3, 1.5 Hz, 1H), 7.79 (dd, *J* = 8.4, 1.3 Hz, 2H), 7.66–7.31 (m, 6H), 6.06 (br s, 1H), 5.26 (d, *J* = 17.9 Hz, 1H), 4.90 (d, *J* = 17.9 Hz, 1H), 4.77 (dd, *J* = 8.4, 4.2 Hz, 1H), 3.88 (dd, *J* = 14.9, 8.4 Hz, 1H), 3.45–3–35 (m, 1H), 2.81 (dd, *J* = 14.9, 4.2 Hz, 1H), 1.53–0.62 (m, 10 H). ^13^C­{^1^H} NMR (75 MHz, CDCl_3_) δ: 195.7 (C_q_), 172.3 (C_q_), 164.9 (C_q_), 147.4 (C_q_), 134.3 (CH), 133.8 (CH), 133.2 (C_q_), 131.9 (C_q_), 129.2 (CH), 128.9 (CH), 128.0 (CH), 124.9 (CH), 76.1 (C_q_), 49.8 (CH), 44.7 (CH_2_), 39.6 (CH), 39.5 (CH_2_), 31.7 (CH_2_), 31.5 (CH_2_), 25.0 (CH_2_), 24.5 (CH_2_). HRMS (ESI+) *m*/*z*: [M + H]^+^ calculated for C_25_H_27_BrN_3_O_5‑_528.1129; found 528.1132.

#### 3-Bromo-5-(*N*-cyclohexylcarbamoyl)-5-(4-fluorobenzoyl)-1-nitrobenzyl-2-pyrrolidinone (**11e**)

Pale yellow oil (84%, 916 mg, method B) (3:1 Hex/EtOAc). ^1^H NMR (300 MHz, CDCl_3_) δ (as diastereomer mixture 64:36): 8.01–7.82 (m, 3H), 7.63–7.35 (m, 3H), 7.16–7.07 (m, 2H), 6.06 (d, *J* = 7.2 Hz, 0.64H major diast), 5.61 (d, *J* = 7.4 Hz, 0.36H minor diast), 5.43 (d, *J* = 18.2 Hz, 0.36H minor diast), 5.23 (d, *J* = 17.1 Hz, 0.64H major diast), 4.90 (d, *J* = 17.1 Hz, 0.64H major diast), 4.84–4.78 (m, 1H), 4.52 (dd, *J* = 9.1, 7.0 Hz, 0.36H minor diast), 3.83 (dd, *J* = 14.8, 8.6 Hz, 0.64H major diast), 3.62 (dd, *J =* 14.9, 7.0 Hz, 0.36H minor diast), 3.48–3.32 (m, 1H), 3.13 (dd, *J* = 14.8, 9.1 Hz, 0.36H minor diast), 2.77 (dd, *J* = 14.8, 4,8 Hz, 0.64H major diast), 1.79–0.59 (m, 10H). ^13^C­{^1^H} NMR (75 MHz, CDCl_3_) δ: 194.7 (C_q_), 192.7 (C_q_), 172.5 (C_q_), 172.0 (C_q_), 165.0 (C_q_), 164.7 (C_q_), 147.6 (C_q_), 133.9 (CH), 133.8 (CH), 132.1 (d,^3^ *J* = 9.5 Hz, CH), 132.1 (d,^3^ *J* = 9.7 Hz, CH), 132.0 (C_q_), 131.7 (C_q_), 129.9 (CH), 129.7 (d,^4^ *J* = 3.0 Hz, C_q_), 128.3 (CH), 128.1 (CH), 125.1 (CH), 124.9 (CH), 116.3 (d,^2^ *J* = 22.1 Hz, CH), 116.3 (d,^2^ *J* = 22.0 Hz, CH), 76.2 (C_q_), 75.8 (C_q_), 49.8 (CH), 46.1 (CH_2_), 44.6 (CH_2_), 44.5 (CH_2_), 39.9 (CH_2_), 39.6 (CH), 39.1 (CH), 38.2 (CH_2_), 32.0 (CH_2_), 31.8 (CH_2_), 31.8 (CH_2_), 31.6 (CH_2_), 25.1 (CH_2_), 24.5 (CH_2_). HRMS (ESI+) *m*/*z*: [M + H]^+^ calculated for C_25_H_26_FBrN_3_O_5‑_546.1034; found 546.1037.

### Synthesis of Bicyclic Ketals (**12**)

#### Method A: Synthesis of Bicyclic Ketal **12a** Starting from Ugi Adduct **10**

A mixture of Ugi adduct **10** (1.0 mmol, 1.0 equiv) and cesium carbonate (2.0 mmol, 2.0 equiv) in methanol (10 mL) was stirred under sonication for 3 h. Subsequently, the solvent was removed under reduced pressure, and the residue was dissolved in chloroform. The resulting solution was washed with a hydrochloric acid aqueous solution (1 M), and the organic layer was dried over sodium sulfate, filtered, and concentrated to dryness under reduced pressure. The crude was purified by column chromatography, employing SiO_2_ as the stationary phase and a hexane/ethyl acetate mixture as the eluent.

#### Method B: Synthesis of Bicyclic Ketals **12** by a One-Pot Sequence

Before use, 2-nitrobenzylamine hydrochloride **1d** (2.0 mmol, 1.0 equiv) was treated with potassium hydroxide (1.8 mmol, 0.9 equiv) under ultrasonic conditions for 10 min. The solution of benzylamine **1c**–**d** in the alcohol (10 mL) was treated with 2,3-dibromopropionic acid **2b** (2.0 mmol, 1.0 equiv), followed by arylglyoxal hydrate **3** (2.0 mmol, 1.0 equiv) and isocyanide **4** (2.0 mmol, 1.0 equiv). The reaction mixture was stirred at room temperature for 24 h. Then, cesium carbonate (4.0 mmol, 2.0 equiv) was added, and the mixture was stirred under sonication for 3 h. Subsequently, the solvent was removed under reduced pressure, and the residue was dissolved in chloroform. Henceforth, the crude was treated as indicated in method A. Single crystals of **12g**, suitable for X-ray diffraction analysis, were grown by the slow evaporation of a solution of the compound in a hexane/ethyl acetate mixture.

#### Method C: Synthesis of Bicyclic Ketal **12c** by a One-Pot Two-Step Sequence

The solution of benzylamine **1c** in methanol (10 mL) was treated with 2,3-dibromopropionic acid **2b** (2.0 mmol, 1.0 equiv), followed by phenylglyoxal hydrate **3a** (2.0 mmol, 1.0 equiv) and isocyanide **4a** (2.0 mmol, 1.0 equiv). The reaction mixture was stirred at room temperature for 24 h, and then, the solvent was removed under reduced pressure. The residue was dissolved in 2-chloroethanol (10 mL), and cesium carbonate (4.0 mmol, 2.0 equiv) was added; the mixture was stirred under sonication for 3 h. Subsequently, the solvent was removed under reduced pressure, and the residue was dissolved in chloroform. Henceforth, the crude was treated as indicated in method A.

#### (1R*,3R*,4R*)-5-Benzyl-4-(*N*-cyclohexylcarbamoyl)-3-methoxy-3-phenyl-2-oxa-5-azabicyclo­[2.2.1]­heptan-6-one (**12a**)

Purple oil (97%, 421 mg, method A); (98%, 852 mg, method B) (4:1 Hex/EtOAc). ^1^H NMR (300 MHz, CDCl_3_) δ: 7.49–7.39 (m, 5H), 7.30 (d, *J* = 8.1 Hz, 1H, NH), 7.22–7.09 (m, 5H), 4.72 (d, *J* = 1.9 Hz, 1H), 3.92–3.79 (m, 1H), 3.64 (d, *J* = 15.3 Hz, 1H), 3.33 (d, *J* = 15.4 Hz, 1H), 3.28 (s, 3H), 2.85 (dd, *J* = 9.8, 2.5 Hz, 1H), 2.40 (d, *J* = 9.7 Hz, 1H), 1.93–1.50 (m, 5H), 1.53–1.11 (m, 5H). ^13^C­{^1^H} NMR (75 MHz, CDCl_3_) δ: 173.7 (C_q_), 163.9 (C_q_), 138.4 (C_q_), 135.5 (C_q_), 129.7 (CH), 128.8 (CH), 128.0 (CH), 127.8 (CH), 127.2 (CH), 126.5 (CH), 107.8 (C_q_), 79.7 (CH), 78.4 (C_q_), 51.4 (CH_3_), 48.0 (CH), 46.3 (CH_2_), 45.6 (CH_2_), 33.1 (CH_2_), 32.8 (CH_2_), 25.6 (CH_2_), 24.8 (CH_2_), 24.7 (CH_2_). HRMS (ESI+) *m*/*z*: [M + Na]^+^ calculated for C_26_H_30_N_2_NaO_4_ 457.2098; found 457.2109.

#### (1R*,3R*,4R*)-5-Benzyl-4-(*N*-cyclohexylcarbamoyl)-3-ethoxy-3-phenyl-2-oxa-5-azabicyclo­[2.2.1]­heptan-6-one (**12b**)

Purple oil (95%, 852 mg, method B) (4:1 Hex/EtOAc). ^1^H NMR (300 MHz, CDCl_3_) δ: 7.64 (d, *J* = 8.3 Hz, 1H, NH), 7.50–7.38 (m, 5H), 7.21–7.11 (m, 5H), 4.71 (d, *J* = 2.2 Hz, 1H), 3.96–3.82 (m, 1H), 3.73 (dq, *J* = 9.6, 7.0 Hz, 1H), 3.56 (d, *J* = 15.3 Hz, 1H), 3.34 (dq, *J* = 9.3, 7.1 Hz, 1H), 3.30 (d, *J* = 15.5 Hz, 1H), 2.87 (dd, *J* = 9.9, 2.5 Hz, 1H), 2.42 (d, *J* = 9.8 Hz, 1H), 2.09–1.53 (m, 5H), 1.49–1.26 (m, 5H), 1.23 (t, *J* = 6.9 Hz, 3H). ^13^C­{^1^H} NMR (75 MHz, CDCl_3_) δ: 173.7 (C_q_), 164.2 (C_q_), 138.6 (C_q_), 136.4 (C_q_), 129.7 (CH), 128.9 (CH), 128.4 (CH), 128.0 (CH), 127.3 (CH), 126.5 (CH), 107.6 (C_q_), 79.8 (CH), 78.5 (C_q_), 59.9 (CH_2_), 47.9 (CH), 46.6 (CH_2_), 45.9 (CH_2_), 33.2 (CH_2_), 32.9 (CH_2_), 25.6 (CH_2_), 24.7 (CH_2_), 24.6 (CH_2_), 15.5 (CH_3_). HRMS (ESI+) *m*/*z*: [M + Na]^+^ calculated for C_27_H_32_N_2_NaO_4_ 471.2254; found 471.2255.

#### (1R*,3R*,4R*)-5-Benzyl-3-(2-chloroethoxy)-4-(*N*-cyclohexylcarbamoyl)-3-phenyl-2-oxa-5-azabicyclo­[2.2.1]­heptan-6-one (**12c**)

Purple oil (67%, 647 mg, method C) (4:1 Hex/EtOAc). ^1^H NMR (300 MHz, CDCl_3_) δ: 7.52–7.40 (m, 6H), 7.21–7.12 (m, 4H), 4.73 (d, *J* = 2.2 Hz, 1H), 4.01–3.91 (m, 1H), 3.90–3.75 (m, 1H), 3.74–3.63 (m, 2H), 3.60 (d, *J* = 15.7 Hz, 1H), 3.56–3.50 (m, 2H), 3.35 (d, *J* = 15.3 Hz, 1H), 2.91 (dd, *J* = 10.0, 2.4 Hz, 1H), 2.44 (d, *J* = 9.9 Hz, 1H), 2.11–1.16 (m, 10H). ^13^C­{^1^H} NMR (75 MHz, CDCl_3_) δ: 173.5 (C_q_), 163.7 (C_q_), 138.4 (C_q_), 135.4 (C_q_), 130.0 (CH), 129.0 (CH), 128.0 (CH), 127.9 (CH) 127.2 (CH), 126.5 (CH), 107.3 (C_q_), 80.0 (CH), 78.7 (C_q_), 63.7 (CH_2_), 48.4 (CH), 46.5 (CH_2_), 45.7 (CH_2_), 43.9 (CH_2_), 33.2 (CH_2_), 32.9 (CH_2_), 25.5 (CH_2_), 25.0 (CH_2_), 24.9 (CH_2_). HRMS (ESI+) *m*/*z*: [M + H]^+^ calculated for C_27_H_32_ClN_2_O_4_ 483.2045; found 483.2037.

#### (1R*,3R*,4R*)-5-Benzyl-4-(*N*-cyclohexylcarbamoyl)-3-phenyl-3-(2,2,2-trifluoroethoxy)-2-oxa-5-azabicyclo­[2.2.1]­heptan-6-one (**12d**)

Pink oil (60%, 302 mg, method B) (4:1 Hex/EtOAc). ^1^H NMR (300 MHz, CDCl_3_) δ: 7.50–7.42 (m, 5H), 7.24–7.11 (m, 5H), 6.82 (d, *J* = 8.0 Hz, 1H, NH), 4.78 (d, *J* = 2.1 Hz, 1H), 4.04 (dq, *J* = 11.4, 8.5 Hz, 1H), 3.88–3.80 (m, 1H), 3.75 (d, *J* = 15.2 Hz, 1H), 3.65 (dq, *J* = 11.4, 8.5 Hz, 1H), 3.40 (d, *J* = 15.2 Hz, 1H), 2.86 (dd, *J* = 10.0, 2.5 Hz, 1H), 2.42 (d, *J* = 10.1 Hz, 1H), 2.08–1.03 (m, 10H). ^13^C­{^1^H} NMR (75 MHz, CDCl_3_) δ: 173.1 (C_q_), 163.0 (C_q_), 138.3 (C_q_), 133.9 (C_q_), 130.4 (CH), 129.1 (CH), 128.1 (CH), 127.9 (CH), 127.3 (CH), 126.7 (CH), 107.4 (C_q_), 80.3 (CH), 77.9 (C_q_), 61.4 (q,^2^ *J* = 35.1 Hz, CH_2_), 48.4 (CH), 46.1 (CH_2_), 45.2 (CH_2_), 32.9 (CH_2_), 32.7 (CH_2_), 25.5 (CH_2_), 24.8 (CH_2_), 24.7 (CH_2_). HRMS (ESI+) *m*/*z*: [M + Na]^+^ calculated for C_27_H_29_F_3_N_2_NaO_4_ 525.1972; found 525.1978.

#### (1R*,3R*,4R*)-5-Benzyl-4-(*N*-cyclohexylcarbamoyl)-3-(4-fluorophenyl)-3-methoxy-2-oxa-5-azabicyclo­[2.2.1]­heptan-6-one (**12e**)

Pink oil (74%, 670 mg, method B) (4:1 Hex/EtOAc). ^1^H NMR (300 MHz, CDCl_3_) δ: 7.48–7.40 (m, 2H), 7.22–6.99 (m, 7H), 7.01 (d, *J* = 8.3 Hz, 1H, NH), 4.71 (d, *J* = 2.3 Hz, 1H), 3.91–3.64 (m, 1H), 3.77 (d, *J* = 15.3 Hz, 1H), 3.37 (d, *J* = 15.2 Hz, 1H), 3.25 (s, 3H), 2.84 (dd, *J* = 9.8, 2.5 Hz, 1H), 2.36 (d, *J* = 9.7 Hz, 1H), 1.94–0.92 (m, 10H). ^13^C­{^1^H} NMR (75 MHz, CDCl_3_) δ: 173.4 (C_q_), 163.6 (d,^1^ *J* = 249.2 Hz, C_q_), 163.6 (C_q_), 138.3 (C_q_), 131.3 (d,^4^ *J* = 3.2 Hz, C_q_), 130.1 (d,^3^ *J* = 7.2 Hz, CH), 128.1 (CH), 127.3 (CH), 126.7 (CH), 115.7 (d,^2^ *J* = 21.6 Hz, CH), 107.6 (C_q_), 79.6 (CH), 77.9 (C_q_), 51.4 (CH_3_), 48.2 (CH), 46.1 (CH_2_), 45.3 (CH_2_), 33.1 (CH_2_), 32.7 (CH_2_), 25.5 (CH_2_), 24.8 (CH_2_), 24.7 (CH_2_). HRMS (ESI+) *m*/*z*: [M + H]^+^ calculated for C_26_H_30_FN_2_O_4_ 453.2184; found 453.2196.

#### (1R*,3R*,4R*)-5-Benzyl-4-(*N*-*tert*-butylcarbamoyl)-3-methoxy-3-phenyl-2-oxa-5-azabicyclo­[2.2.1]­heptan-6-one (**12f**)

Orange solid (64%, 523 mg, method B) (4:1 Hex/EtOAc). M.p.: 147–149 °C. ^1^H NMR (300 MHz, CDCl_3_) δ: 7.48–7.39 (m, 6H), 7.18–7.10 (m, 4H), 7.01 (s, 1H, NH), 4.69 (d, *J* = 2.3 Hz, 1H), 3.70 (d, *J* = 15.3 Hz, 1H), 3.33 (d, *J* = 15.3 Hz, 1H), 3.26 (s, 3H), 2.83 (dd, *J* = 9.7, 2.7 Hz, 1H), 2.35 (d, *J* = 9.7 Hz, 1H), 1.34 (s, 9H). ^13^C­{^1^H} NMR (75 MHz, CDCl_3_) δ: 173.6 (C_q_), 163.5 (C_q_), 138.4 (C_q_), 135.4 (C_q_), 129.7 (CH), 128.9 (CH), 128.7 (CH), 128.0 (CH), 127.5 (CH), 126.6 (CH), 107.7 (C_q_), 79.5 (CH), 78.4 (C_q_), 51.6 (C_q_), 51.3 (CH_3_), 46.1 (CH_2_), 45.2 (CH_2_), 28.7 (CH_3_). HRMS (ESI+) *m*/*z*: [M + H]^+^ calculated for C_24_H_29_N_2_O_4_ 409.2122; found 409.2133.

#### (1R*,3R*,4R*)-4-(*N*-Cyclohexylcarbamoyl)-3-methoxy-5-(2-nitrobenzyl)-3-phenyl-2-oxa-5-azabicyclo­[2.2.1]­heptan-6-one (**12g**)

Pink solid (96%, 921 mg, method B) (4:1 Hex/EtOAc). M.p.: 177–179 °C. ^1^H NMR (300 MHz, CDCl_3_) δ: 7.94 (d, *J* = 8.2 Hz, 1H), 7.67 (d, *J* = 8.4 Hz, 1H, NH), 7.59–7.38 (m, 7H), 7.28–7.20 (m, 1H), 4.76 (d, *J* = 2.2 Hz, 1H), 4.01–3.74 (m, 1H), 3.86 (d, *J* = 19.0 Hz, 1H), 3.55 (d, *J* = 17.8 Hz, 1H), 3.31 (s, 3H), 2.92 (dd, *J* = 10.2, 2.4 Hz, 1H), 2.51 (d, *J* = 10.2 Hz, 1H), 2.09–0.98 (m, 10H). ^13^C­{^1^H} NMR (75 MHz, CDCl_3_) δ: 174.0 (C_q_), 164.0 (C_q_), 146.7 (C_q_), 135.0 (C_q_), 134.8 (C_q_), 133.4 (CH), 130.2 (CH), 129.5 (CH), 129.2 (CH), 128.7 (CH), 128.4 (CH), 127.1 (CH), 124.6 (CH), 107.7 (C_q_), 79.5 (CH), 78.9 (C_q_), 51.5 (CH_3_), 47.9 (CH), 45.7 (CH_2_), 44.9 (CH_2_), 33.0 (CH_2_), 32.8 (CH_2_), 25.4 (CH_2_), 24.6 (CH_2_), 24.5 (CH_2_). HRMS (ESI+) *m*/*z*: [M + H]^+^ calculated for C_26_H_30_N_3_O_6_ 480.2129; found 480.2129.

#### (1R*,3R*,4R*)-4-(*N*-Cyclohexylcarbamoyl)-3-(4-fluorophenyl)-3-methoxy-5-(2-nitrobenzyl)-2-oxa-5-azabicyclo­[2.2.1]­heptan-6-one (**12h**)

Pink oil (69%, 687 mg, method B) (4:1 Hex/EtOAc). ^1^H NMR (300 MHz, CDCl_3_) δ: 7.97 (d, *J* = 7.0 Hz, 1H, NH), 7.63–7.37 (m, 4H), 7.34–7.07 (m, 4H), 4.77 (d, *J* = 2.3 Hz, 1H), 3.93 (d, *J* = 17.6 Hz, 1H), 3.88–3.70 (m, 1H), 3.63 (d, *J* = 17.6 Hz, 1H), 3.30 (s, 3H), 2.91 (dd, *J* = 10.2, 2.5 Hz, 1H), 2.51 (d, *J* = 10.1 Hz, 1H), 2.06–0.92 (m, 10H). ^13^C­{^1^H} NMR (75 MHz, CDCl_3_) δ: 173.9 (C_q_), 168.2 (C_q_), 163.9 (C_q_), 163.9 (d,^1^ *J* = 250.9 Hz, C_q_), 148.0 (C_q_), 147.0 (C_q_), 134.8 (C_q_), 133.5 (d,^4^ *J* = 1.3 Hz, CH), 131.0 (d,^3^ *J* = 3.2 Hz, C_q_), 128.8 (CH), 127.2 (CH), 124.7 (CH), 116.3 (d,^2^ *J* = 21.7 Hz, CH), 107.5 (C_q_), 79.6 (CH), 78.9 (C_q_), 51.5 (CH_3_), 48.1 (CH), 45.7 (CH_2_), 44.9 (CH_2_), 33.1 (CH_2_), 32.8 (CH_2_), 25.5 (CH_2_), 24.7 (CH_2_), 24.6 (CH_2_). HRMS (ESI+) *m*/*z*: [M + H]^+^ calculated for C_26_H_29_FN_3_O_6_ 498.2035; found 498.2041.

### Synthesis of 3-Hydroxy-2-pyrrolidinones (**13**)

A mixture of ketal **12** (1.0 mmol, 1.0 equiv) and a hydrochloric acid aqueous solution (1 M) (2 mL) in tetrahydrofuran (10 mL) was stirred under reflux for 16 h. Subsequently, the solvent was removed under reduced pressure, and the residue was dissolved in chloroform. The resulting solution was washed with a sodium bicarbonate aqueous solution (1 M), and the organic layer was dried over sodium sulfate, filtered, and concentrated to dryness under reduced pressure. The crude was purified by column chromatography, employing SiO_2_ as the stationary phase and a hexane/ethyl acetate mixture as the eluent, or by precipitation, using a hexane/ethyl acetate mixture. Single crystals of **13a**, suitable for X-ray diffraction analysis, were grown by the slow evaporation of a solution of the compound in a hexane/ether mixture.

#### (3R*,5R*)-5-Benzoyl-1-benzyl-5-(*N*-cyclohexylcarbamoyl)-3-hydroxy-2-pyrrolidinone (**13a**)

White solid (80%, 336 mg) (purification by precipitation in a Hex/EtOAc mixture). M.p.: 135–137 °C. ^1^H NMR (300 MHz, CDCl_3_) δ: 7.81 (d, *J* = 8.7 Hz, 2H), 7.60–7.50 (m, 1H), 7.41 (t, *J* = 7.7 Hz, 2H), 7.21–7.12 (m, 3H), 7.03 (dd, *J* = 6.7, 3.0 Hz, 2H), 6.47 (d, *J* = 7.7 Hz, 1H, NH), 4.90 (d, *J* = 15.8 Hz, 1H), 4.80 (dd, *J* = 8.7, 6.9 Hz, 1H), 4.34 (d, *J* = 15.9 Hz, 1H), 3.83–3.57 (m, 1H), 3.57–3.30 (m, 1H, OH), 3.16 (dd, *J* = 13.7, 8.7 Hz, 1H), 2.40 (dd, *J* = 13.7, 6.9 Hz, 1H), 2.06–0.78 (m, 10H). ^13^C­{^1^H} NMR (75 MHz, CDCl_3_) δ: 197.5 (C_q_), 176.6 (C_q_), 167.2 (C_q_), 135.3 (C_q_), 134.1 (C_q_), 134.0 (CH), 129.5 (CH), 128.9 (CH), 128.5 (CH), 128.3 (CH), 127.6 (CH), 73.6 (C_q_), 68.4 (CH), 49.3 (CH), 47.3 (CH_2_), 38.9 (CH_2_), 32.1 (CH_2_), 32.0 (CH_2_), 25.4 (CH_2_), 24.7 (CH_2_), 24.6 (CH_2_). HRMS (ESI+) *m*/*z*: [M + H]^+^ calculated for C_25_H_29_N_2_O_4_ 421.2127; found 421.2116.

#### (3R*,5R*)-1-Benzyl-5-(*N*-cyclohexylcarbamoyl)-5-(4-fluorobenzoyl)-3-hydroxy-2-pyrrolidinone (**13b**)

Orange solid (58%, 254 mg) (4:1 Hex/EtOAc). M.p.: 175–177 °C. ^1^H NMR (300 MHz, CDCl_3_) δ: 7.93–7.76 (m, 2H), 7.16–6.98 (m, 7H), 6.71 (d, *J* = 7.6 Hz, 1H, NH), 4.85–4.67 (m, 1H), 4.76 (d, *J* = 16.0 Hz, 1H), 4.42 (d, *J* = 15.8 Hz, 1H), 4.28–4.04 (m, 1H, OH), 3.58–3.33 (m, 1H), 3.12 (dd, *J* = 13.8, 8.6 Hz, 1H), 2.37 (dd, *J* = 13.8, 6.7 Hz, 1H), 1.89–0.72 (m, 10H). ^13^C­{^1^H} NMR (75 MHz, CDCl_3_) δ: 195.9 (C_q_), 176.9 (C_q_), 173.6 (d,^1^ *J* = 265.8 Hz, C_q_), 167.2 (C_q_), 135.2 (C_q_), 132.3 (d,^3^ *J* = 9.3 Hz, CH), 130.3 (d,^4^ *J* = 3.4 Hz, C_q_), 128.5 (CH), 128.4 (CH), 127.7 (CH), 116.0 (d,^2^ *J* = 22.1 Hz, CH), 73.5 (C_q_), 68.3 (CH), 49.4 (CH), 47.4 (CH_2_), 38.8 (CH_2_), 32.2 (CH_2_), 32.1 (CH_2_), 25.4 (CH_2_), 24.7 (CH_2_), 24.6 (CH_2_). HRMS (ESI+) *m*/*z*: [M + H]^+^ calculated for C_25_H_28_FN_2_O_4_ 439.2033; found 439.2028.

#### (3R*,5R*)-5-Benzoyl-1-benzyl-5-(*N*-*tert*-butylcarbamoyl)-3-hydroxy-2-pyrrolidinone (**13c**)

Pink oil (54%, 213 mg) (4:1 Hex/EtOAc). M.p.: 155–157 °C. ^1^H NMR (300 MHz, CDCl_3_) δ: 7.82 (d, *J* = 7.5 Hz, 2H), 7.60–7.48 (m, 1H), 7.40 (t, *J* = 7.9 Hz, 2H), 7.15 (dd, *J* = 5.1, 1.8 Hz, 3H), 7.05 (dd, *J* = 6.9, 3.0 Hz, 2H), 6.35 (s, 1H), 4.92 (d, *J* = 16.0 Hz, 1H), 4.80 (dd, *J* = 8.7, 6.6 Hz, 1H), 4.35 (d, *J* = 16.0 Hz, 1H), 3.20 (dd, *J* = 13.8, 8.7 Hz, 1H), 2.38 (dd, *J* = 13.8, 6.6 Hz, 1H), 1.00 (s, 9H). ^13^C­{^1^H} NMR (75 MHz, CDCl_3_) δ: 197.4 (C_q_), 177.0 (C_q_), 166.7 (C_q_), 135.6 (C_q_), 133.9 (CH), 129.4 (CH), 128.8 (CH), 128.5 (CH), 128.1 (CH), 127.5 (CH), 74.4 (C_q_), 68.2 (CH), 52.3 (C_q_), 47.3 (CH_2_), 38.8 (CH_2_), 28.0 (CH_3_). HRMS (ESI+) *m*/*z*: [M + H]^+^ calculated for C_23_H_27_N_2_O_4_ 395.1965; found 395.19670.

#### (3R*,5R*)-5-Benzoyl-5-(*N*-cyclohexylcarbamoyl)-3-hydroxy-1-(2-nitrobenzyl)-2-pyrrolidinone (**13d**)

Orange oil (60%, 280 mg) (4:1 Hex/EtOAc). ^1^H NMR (300 MHz, CDCl_3_) δ: 7.92 (d, *J* = 8.2 Hz, 1H), 7.85–7.77 (m, 2H), 7.65–7.31 (m, 6H), 6.32 (d, *J* = 8.1 Hz, 1H), 5.12 (d, *J* = 17.3 Hz, 1H), 4.81 (d, *J* = 17.2 Hz, 1H), 4.89–4.75 (m, 1H), 3.76–3.56 (m, 1H), 3.53–3.45 (m, 1H), 3.39 (dd, *J* = 13.7, 8.5 Hz, 1H), 2.36 (dd, *J* = 13.6, 6.7 Hz, 1H), 1.39–1.10 (m, 10H). ^13^C­{^1^H} NMR (75 MHz, CDCl_3_) δ: 196.7 (C_q_), 176.9 (C_q_), 166.3 (C_q_), 134.2 (C_q_), 133.8 (CH), 132.0 (C_q_), 130.4 (CH), 129.3 (CH), 129.1 (CH), 129.0 (CH), 128.2 (CH), 124.9 (CH), 74.7 (C_q_), 68.3 (CH), 49.6 (CH), 43.9 (CH_2_), 38.7 (CH_2_), 32.1 (CH_2_), 32.0 (CH_2_), 25.3 (CH_2_), 24.7 (CH_2_), 24.6 (CH_2_). HRMS (ESI+) *m*/*z*: [M + H]^+^ calculated for C_25_H_28_N_3_O_6_ 466.1973; found 466.1984.

#### (3R*,5R*)-5-(*N*-Cyclohexylcarbamoyl)-5-(4-fluorobenzoyl)-3-hydroxy-1-(2-nitrobenzyl)-2-pyrrolidinone (**13e**)

Brown oil (72%, 348 mg) (purification by precipitation in a Hex/EtOAc mixture). ^1^H NMR (300 MHz, CDCl_3_) δ: 7.90 (d, *J* = 7.9 Hz, 1H), 7.84 (dd, *J* = 8.7, 5.2 Hz, 2H), 7.53 (t, *J* = 7.3 Hz, 1H), 7.36 (d, *J* = 8.0 Hz, 2H), 7.08 (t, *J* = 8.4 Hz, 2H), 6.48 (d, *J* = 7.9 Hz, 1H, NH), 5.09 (d, *J* = 17.3 Hz, 1H), 4.91–4.71 (m, 1H), 4.83 (d, *J* = 17.1 Hz, 1H), 4.13–3.55 (m, 1H), 3.60–3.29 (m, 1H), 3.39 (dd, *J* = 13.7, 8.5 Hz, 1H), 2.35 (dd, *J* = 13.9, 5.6 Hz, 1H), 1.74–0.61 (m, 10H). ^13^C­{^1^H} NMR (75 MHz, CDCl_3_) δ: 195.0 (C_q_), 177.1 (C_q_), 167.1 (C_q_), 166.1 (d,^1^ *J* = 258.5 Hz, C_q_), 166.0 (C_q_), 147.6 (C_q_), 133.8 (CH), 132.3 (d,^3^ *J* = 9.1 Hz, CH), 131.9 (CH), 129.9 (d,^4^ *J* = 7.9 Hz, C_q_), 128.2 (CH), 124.9 (CH), 116.2 (d,^2^ *J* = 22.0 Hz, CH), 74.8 (C_q_), 68.1 (CH), 49.6 (CH), 44.0 (CH_2_), 38.3 (CH_2_), 32.0 (CH_2_), 31.8 (CH_2_), 25.2 (CH_2_), 24.7 (CH_2_), 24.6 (CH_2_). HRMS (ESI+) *m*/*z*: [M + H]^+^ calculated for C_25_H_27_FN_3_O_6_ 484.1878; found 484.1889.

### Synthesis of Morpholinequinazolines (**14**)

A mixture of ketal **12g**–**h** (1.0 mmol, 1.0 equiv), tin­(II) chloride (10.0 mmol, 10.0 equiv), and a hydrochloric acid aqueous solution (1 M) (3.0 mmol, 3.0 equiv) in methanol (10 mL) was stirred under reflux for 1 h. Subsequently, the solvent was removed under reduced pressure, and the residue was dissolved in chloroform. The resulting solution was washed with a sodium carbonate aqueous solution (1 M), and the organic layer was dried over sodium sulfate, filtered, and concentrated to dryness under reduced pressure.

#### (1R*,3R*,4R*)-4-(*N*-Cyclohexylcarbamoyl)-3-methoxy-3-phenyl-1,3,4,6-tetrahydro-1,4-methano­[1,4]­oxazino­[3,4-*b*]­quinazoline (**14a**)

Orange solid (55%, 237 mg) (4:1 Hex/EtOAc). M.p.: 108–110 °C. ^1^H NMR (300 MHz, CDCl_3_) δ: 7.54–7.08 (m, 9H), 6.57 (d, *J* = 7.7 Hz, 1H, NH), 6.17 (*J* = 2.0 Hz, 1H), 5.16 (d, *J* = 15.8 Hz, 1H), 3.98–3.80 (m, 1H), 3.30 (s, 3H), 3.07 (dd, *J* = 10.9, 2.0 Hz, 1H), 2.96 (d, *J* = 15.3 Hz, 1H), 2.64 (d, *J* = 10.7 Hz, 1H), 2.14–1.15 (m, 10H). ^13^C­{^1^H} NMR (75 MHz, CDCl_3_) δ: 163.0 (C_q_), 161.8 (C_q_), 133.7 (C_q_), 130.6 (CH), 129.5 (CH), 129.4 (CH), 129.1 (C_q_), 128.3 (CH), 126.5 (CH), 119.5 (CH), 119.4 (CH), 117.3 (C_q_), 108.0 (C_q_), 80.7 (CH), 77.1 (C_q_), 52.2 (CH_3_), 48.9 (CH), 46.8 (CH_2_), 45.2 (CH_2_), 33.0 (CH_2_), 32.6 (CH_2_), 25.5 (CH_2_), 24.7 (CH_2_), 24.6 (CH_2_). HRMS (ESI+) *m*/*z*: [M + H]^+^ calculated for C_26_H_30_N_3_O_3_ 432.2282; found 432.2288.

#### (1R*,3R*,4R*)-4-(*N*-Cyclohexylcarbamoyl)-3-(4-fluorophenyl)-3-methoxy-1,3,4,6-tetrahydro-1,4-methano­[1,4]­oxazino­[3,4-*b*]­quinazoline (**14b**)

Orange oil (58%, 261 mg) (4:1 Hex/EtOAc). ^1^H NMR (300 MHz, CDCl_3_) δ: 7.46 (d, *J* = 7.9 Hz, 1H), 7.40 (dd, *J* = 8.9, 5.0 Hz, 3H), 7.26–7.15 (m, 2H), 7.10 (t, *J* = 7.5 Hz, 2H), 6.64 (d, *J* = 7.6 Hz, 1H, NH), 6.10 (d, *J* = 2.2 Hz, 1H), 5.15 (d, *J* = 15.4 Hz, 1H), 3.98–3.79 (m, 1H), 3.28 (s, 3H), 3.14 (d, *J* = 16.1 Hz, 1H), 3.08 (dd, *J* = 10.5, 2.3 Hz, 1H), 2.68 (d, *J* = 10.8 Hz, 1H), 2.13–1.16 (m, 10H). ^13^C­{^1^H} NMR (75 MHz, CDCl_3_) δ: 163.8 (d,^1^ *J* = 251.5 Hz, C_q_), 162.9 (C_q_), 161.6 (C_q_), 129.7 (d,^4^ *J* = 2.9 Hz, C_q_), 129.4 (d,^3^ *J* = 5.0 Hz, CH), 129.3 (CH), 128.4 (CH), 126.6 (CH), 119.6 (CH), 117.1 (C_q_), 116.5 (d,^2^ *J* = 21.8 Hz, CH), 107.7 (C_q_), 80.5 (C_q_), 77.0 (CH), 52.1 (CH_3_), 49.0 (CH), 46.7 (CH_2_), 45.2 (CH_2_), 33.0 (CH_2_), 32.6 (CH_2_), 25.4 (CH_2_), 24.8 (CH_2_), 24.7 (CH_2_). HRMS (ESI+) *m*/*z*: [M + H]^+^ calculated for C_26_H_29_FN_3_O_3_ 450.2187; found 450.2200.

### Synthesis of Pyrrolo­[2,1-*b*]­quinazolin-3-ols (**15**)

A mixture of pyrroloquinazoline **14** (1.0 mmol, 1.0 equiv) and a hydrochloric acid aqueous solution (1 M) (2 mL) in tetrahydrofuran (10 mL) was stirred under reflux for 16 h. Subsequently, the solvent was removed under reduced pressure, and the residue was dissolved in chloroform. The resulting solution was washed with a sodium bicarbonate aqueous solution (1 M), and the organic layer was dried over sodium sulfate, filtered, and concentrated to dryness under reduced pressure. The crude was purified by precipitation using a hexane/ethyl acetate mixture.

#### (1R*,3R*)-1-Benzoyl-1-(*N*-cyclohexylcarbamoyl)-3-hydroxy-1,2,3,9-tetrahydropyrrolo­[2,1-*b*]­quinazoline (**15a**)

Orange oil (60%, 251 mg) (4:1 Hex/EtOAc). ^1^H NMR (300 MHz, CDCl_3_) δ: 7.98–7.87 (m, 2H), 7.63–7.53 (m, 1H), 7.43 (dd, *J* = 8.4, 7.2 Hz, 2H), 7.17 (d, *J* = 7.7 Hz, 2H), 6.99 (td, *J* = 7.2, 1.8 Hz, 1H), 6.79 (d, *J* = 7.5 Hz, 1H), 6.78 (d, *J* = 7.5 Hz, 1H, NH), 5.18 (t, *J* = 7.6 Hz, 1H), 4.59 (d, *J* = 13.1 Hz, 1H), 4.54 (d, *J* = 12.3 Hz, 1H), 3.96–3.80 (m, 1H), 3.73–3.55 (m, 1H, OH), 3.18 (dd, *J* = 13.6, 8.2 Hz, 1H), 2.42 (dd, *J* = 13.6, 6.6 Hz, 1H), 1.95–0.95 (m, 10H). ^13^C­{^1^H} NMR (75 MHz, CDCl_3_) δ: 163.2 (C_q_), 161.7 (C_q_), 133.6 (C_q_), 130.6 (CH), 129.6 (C_q_), 129.5 (CH), 129.4 (CH), 128.9 (CH), 128.4 (CH), 126.5 (CH), 119.3 (CH), 117.2 (C_q_), 108.0 (C_q_), 80.8 (C_q_), 52.2 (CH), 48.8 (CH), 46.7 (CH_2_), 45.2 (CH_2_), 33.0 (CH_2_), 32.6 (CH_2_), 25.4 (CH_2_), 24.7 (CH_2_), 24.7 (CH_2_). HRMS (ESI+) *m*/*z*: [M + H]^+^ calculated for C_25_H_28_N_3_O_3_ 418.2125; found 418.2126.

#### (1R*,3R*)-1-(*N*-Cyclohexylcarbamoyl)-1-(4-fluorobenzoyl)-3-hydroxy-1,2,3,9-tetrahydropyrrolo­[2,1-*b*]­quinazoline (**15b**)

Orange oil (65%, 283 mg) (4:1 Hex/EtOAc). ^1^H NMR (300 MHz, CDCl_3_) δ: 8.81 (d, *J* = 8.0 Hz, 1H, NH), 7.88 (dd, *J* = 8.9, 5.2 Hz, 2H), 7.20–7.10 (m, 4H), 7.07–6.97 (m, 2H), 5.97 (dd, *J* = 8.8, 4.1 Hz, 1H), 5.15 (d, *J* = 15.2 Hz, 1H), 4.65 (d, *J* = 15.2 Hz, 1H), 3.89 (dd, *J* = 15.0, 8.8 Hz, 1H), 3.85–3.72 (m, 1H), 3.68–3.42 (m, 1H, OH), 2.46 (dd, *J* = 14.9, 4.0 Hz, 1H), 2.01–1.47 (m, 6H), 1.41–1.11 (m, 4H). ^13^C­{^1^H} NMR (75 MHz, CDCl_3_) δ: 190.6 (C_q_), 164.4 (C_q_), 164.3 (C_q_), 132.1 (d,^3^ *J* = 9.5 Hz, CH), 130.3 (C_q_), 129.4 (CH), 129.2 (d,^4^ *J* = 3.3 Hz, C_q_), 127.9 (CH), 127.0 (CH), 118.1 (CH), 117.0 (C_q_), 116.2 (d,^2^ *J* = 22.1 Hz, CH), 81.0 (C_q_), 70.1 (CH), 50.5 (CH), 45.6 (CH_2_), 37.1 (CH_2_), 32.5 (CH_2_), 32.3 (CH_2_), 25.4 (CH_2_), 25.4 (CH_2_), 25.3 (CH_2_). HRMS (ESI+) *m*/*z*: [M + H]^+^ calculated for C_25_H_27_FN_3_O_3_ 436.2031; found 436.2042.

## Supplementary Material



## Data Availability

The data underlying this study are available in the published article and its Supporting Information.

## Notes

Copies of ^1^H, ^13^C, and DEPT-135 NMR and ESI-HRMS spectra; X-ray crystal data, refinement details, and Olex2 plots for (*R*)-**6b**, **9e**, **12g**, and **13a** (PDF)

## Notes

Deposition Numbers 2526378 [(*R*)-**6b**], 2526379 (**9e**), 2526380 (**12g**), and 2526381 (**13a**) contain the crystallographic supplementary data for this paper. These data can be obtained free of CHge via the joint Cambridge Crystallographic Data Centre (CCDC) and Fachinformationszentrum Karlsruhe.
