# Supplementary material for: Halogenated Reagents in the Ugi Reaction: From Rearrangement Reactions to Ketal Synthesis
Source: ACS Omega. 2026 May 5;11(19):29224–36. doi: 10.1021/acsomega.6c03196 (PMC13191683; doi:10.1021/acsomega.6c03196)
Supplement: Supplementary file 1 [file ao6c03196_si_001.pdf]

The Journal of Organic Chemistry

Supporting Information

Halogenated reagents in the Ugi reaction. From rearrangement reactions to ketal synthesis

Beatriz González-Saiz, Carlos Cámara-Herrero, Sandra Díaz-Cabrera, Israel Carreira-Barral, Roberto Quesada, María García-Valverde*

Corresponding author e-mail: magaval@ubu.es

CONTENTS

1. **Additional experimental results…………………………………………………………………… S2**
   1.1. **Competitive reactions in the Ugi reaction with 2-chloroethylamine**
   1.2. **Synthesis of rearrangement products from 2-aminoethanol**
2. **NMR and HRMS spectra of compounds synthetized……………………………………… S4**
3. **X-Ray diffraction studies……………………………………………………………………………… S87**

1. ADDITIONAL EXPERIMENTAL RESULTS

1.1. COMPETITIVE REACTIONS IN THE UGI REACTION WITH 2-CHLOROETHYLAMINE

Products observed in the reaction between 2-bromoethylamine **1a**, 3-bromopopanoic acid **2a**, arylglyoxals **3** and isocyanides **4**.

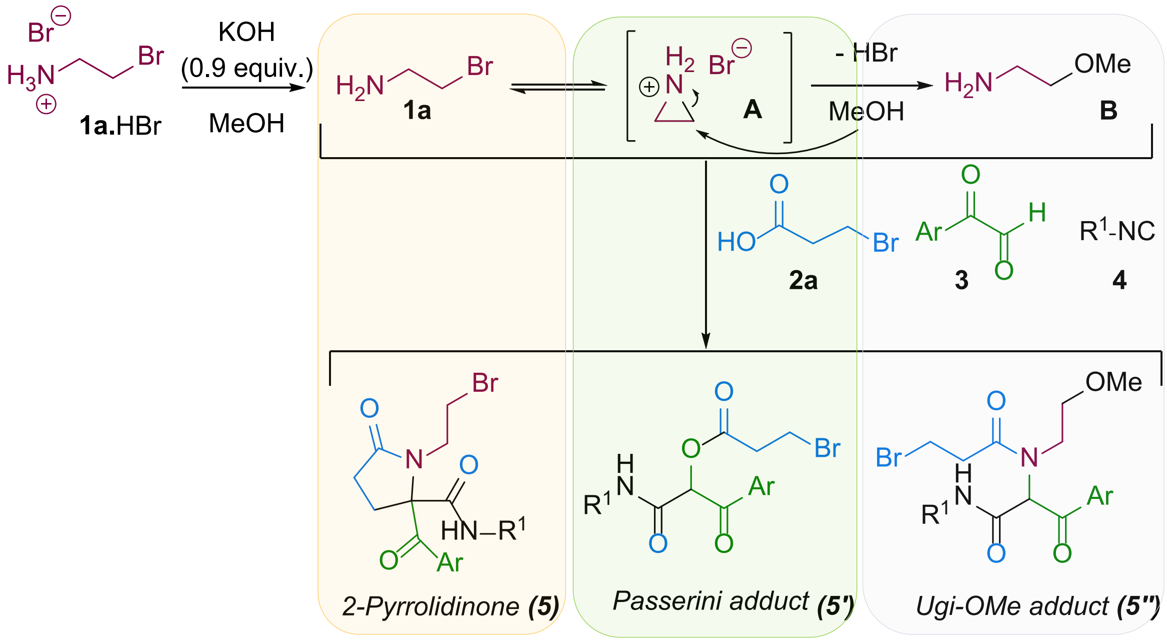

Scheme S1. Competitive reactions in the Ugi reaction with 2-bromoethylamine.

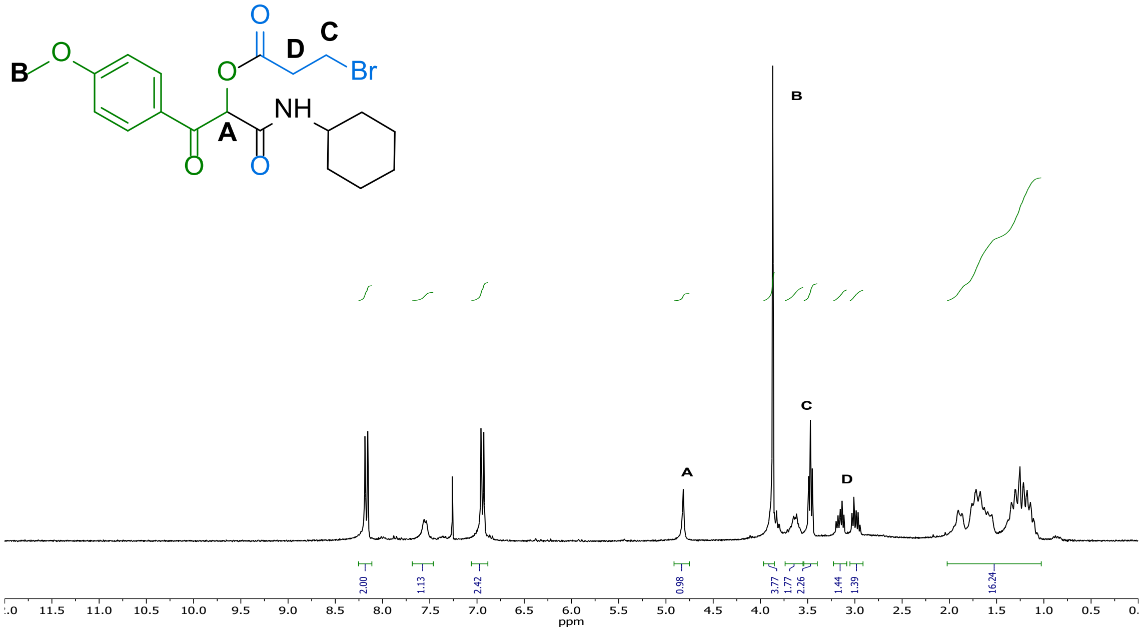

Figure S1. ^{1}H NMR spectrum of Passerini adduct 5' (300 MHz, $CDCl_3$).

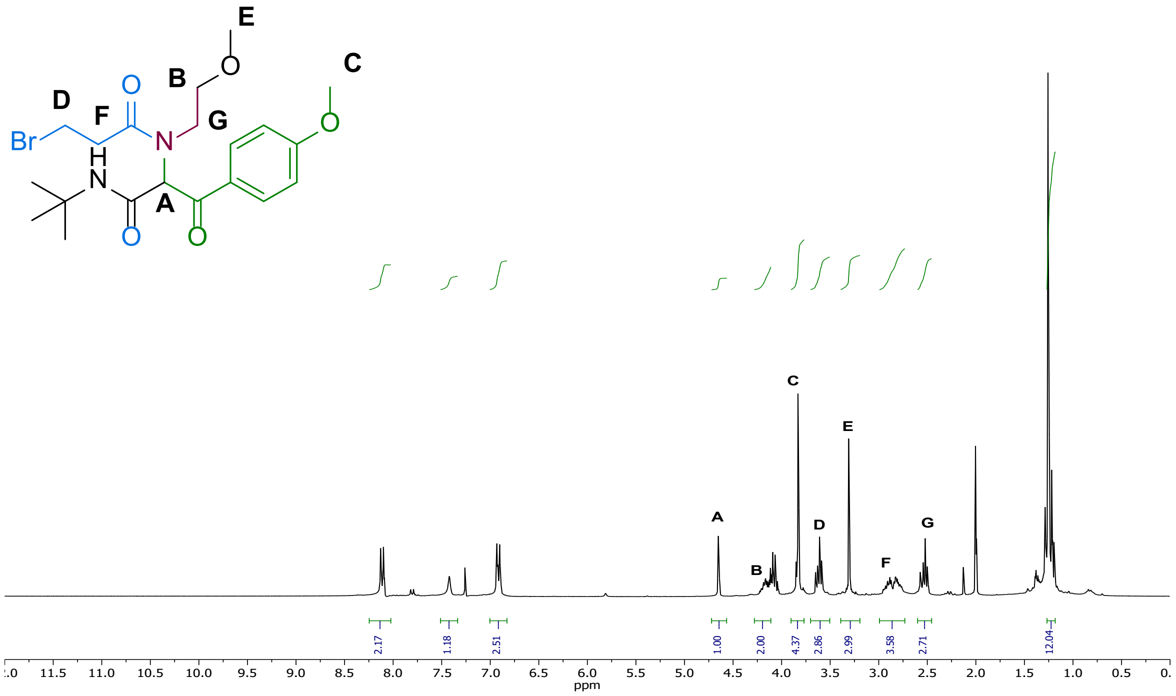

Figure S2. ^{1}**H NMR spectrum of Ugi-OMe adduct 5'' (300 MHz, CDCl**$_3$**).**

1.2. SYNTHESIS OF REARRANGEMENT PRODUCTS FROM 2-AMINOETHANOL

The Ugi reaction with 2-aminoethanol was carried out in dry methanol[1] and the *N*-(2-hydroxyethyl)pyrrolidine-2-one **7** was treated with cesium carbonate in refluxing acetonitrile for 1 h, affording the rearrangement product **6b**.

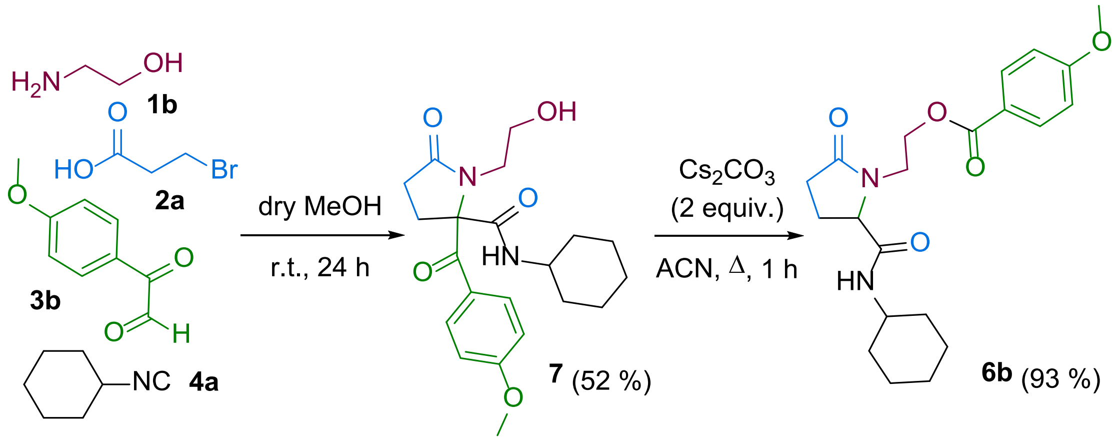

Scheme S2. Ugi/rearrangement sequence starting from 2-aminoethanol.

[1] L. Banfi, A. Basso, G. Guanti, P. Lecinska, R. Riva, Multicomponent synthesis of dihydrobenzoxazepinones by coupling Ugi and Mitsunobu reactions. *Org. Biomol. Chem.*, **2006**, *4*, 4236 – 4240.

2. NMR AND HRMS SPECTRA OF COMPOUNDS SYNTHETIZED

5-Benzoyl-1-(2-bromoethyl)-5-(*N*-cyclohexylcarbamoyl)-2-pyrrolidinone (5a).

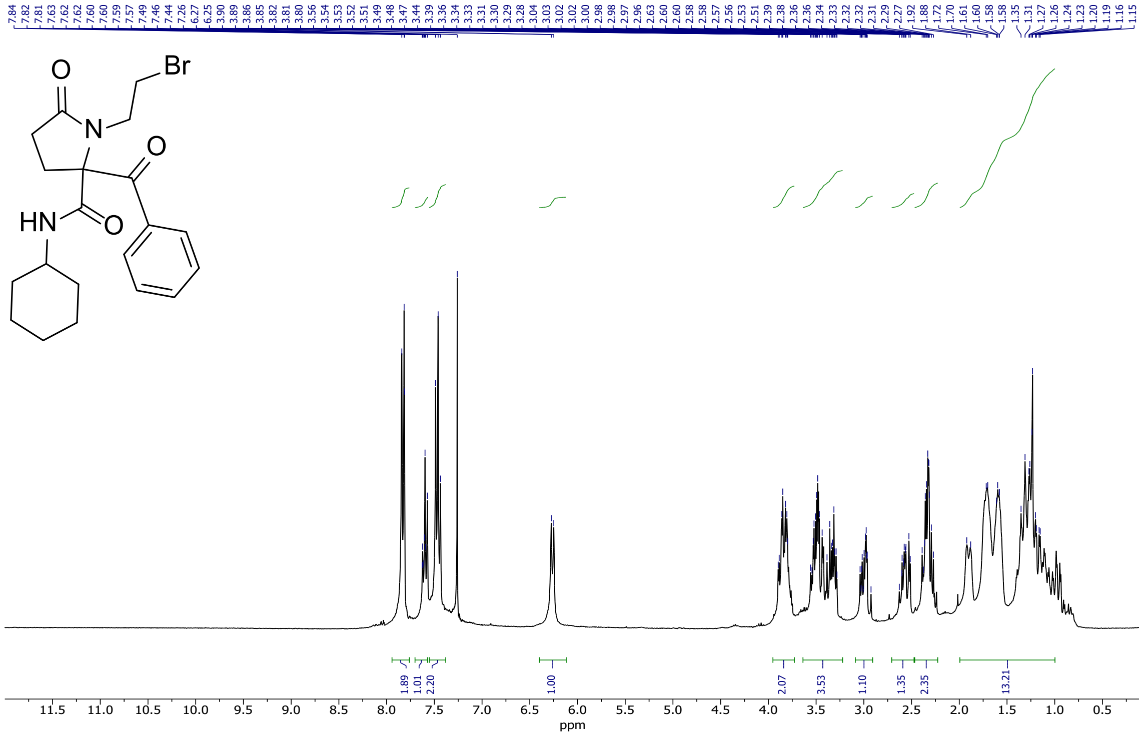

Figure S3. ^{1}H NMR spectrum of 5a (300 MHz, $CDCl_3$).

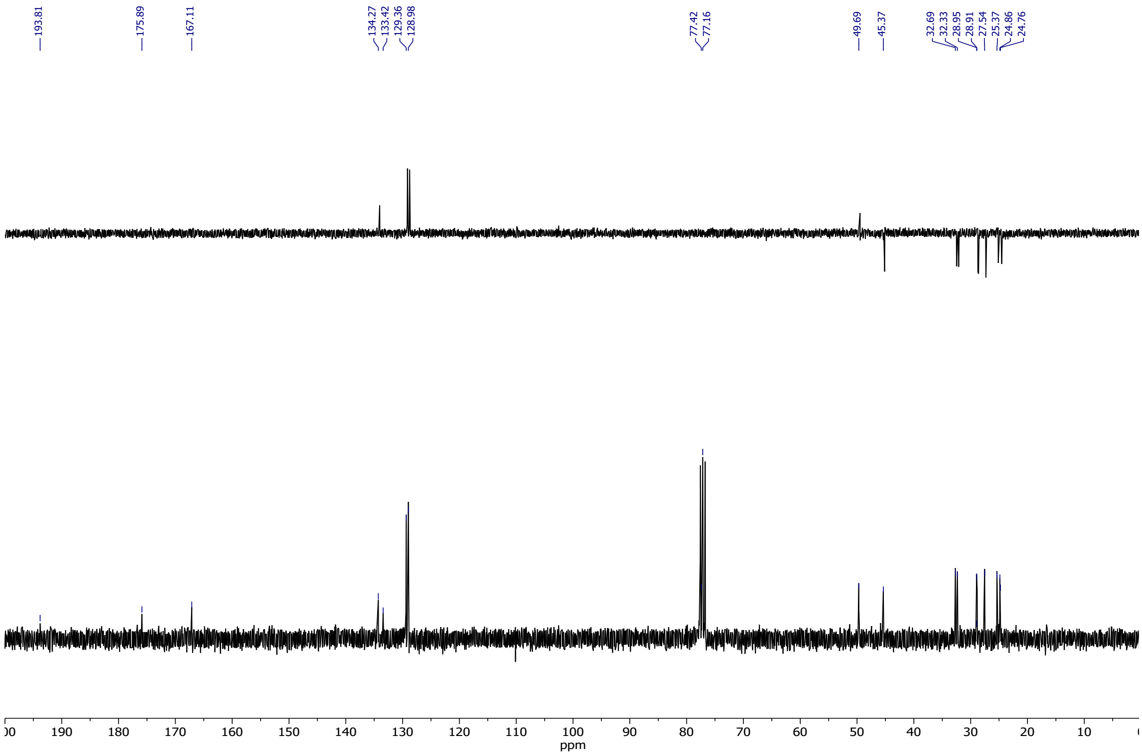

Figure S4. ^{13}C and DEPT NMR spectra of 5a (75 MHz, $CDCl_3$).

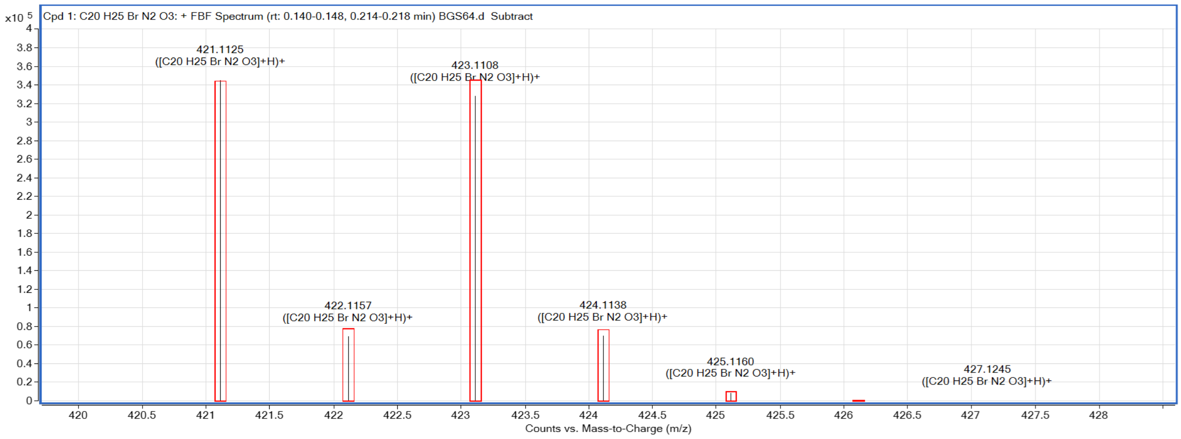

Figure S5. HRMS spectrum of 5a.

1-(2-Bromoethyl)-5-(*N*-cyclohexylcarbamoyl)-5-(4-methoxybenzoyl)-2-pyrrolidinone (5b).

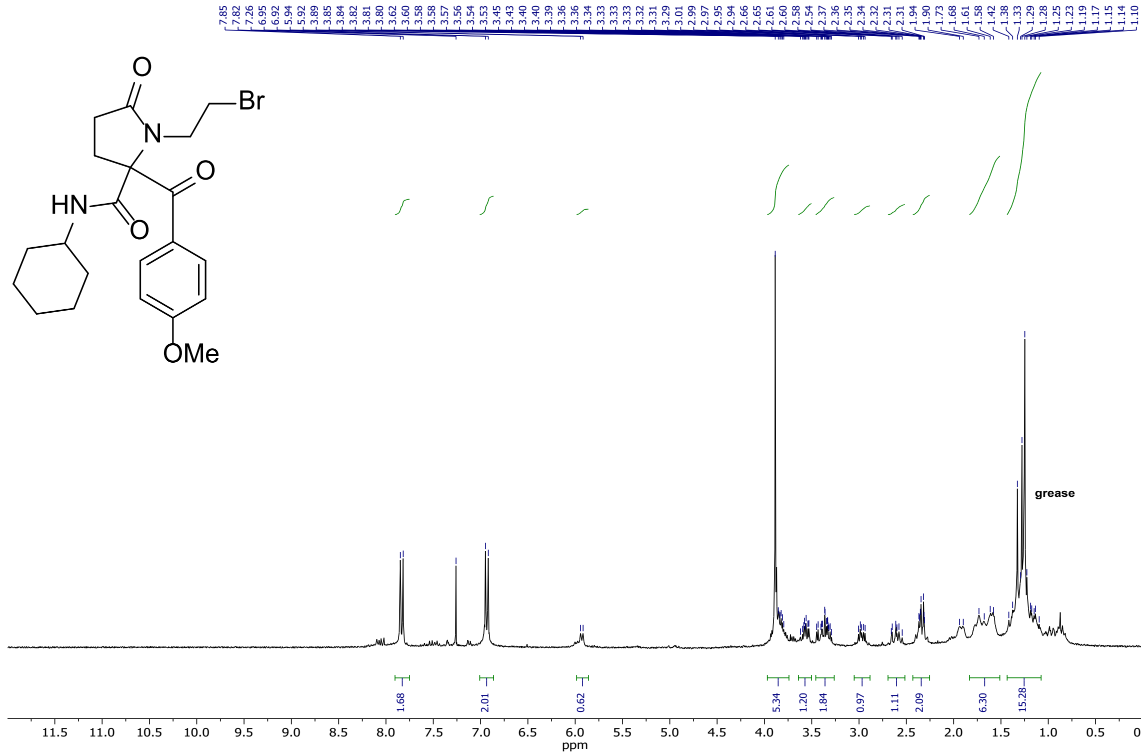

Figure S6. ^{1}H **NMR spectrum of 5b (300 MHz, $CDCl_3$).**

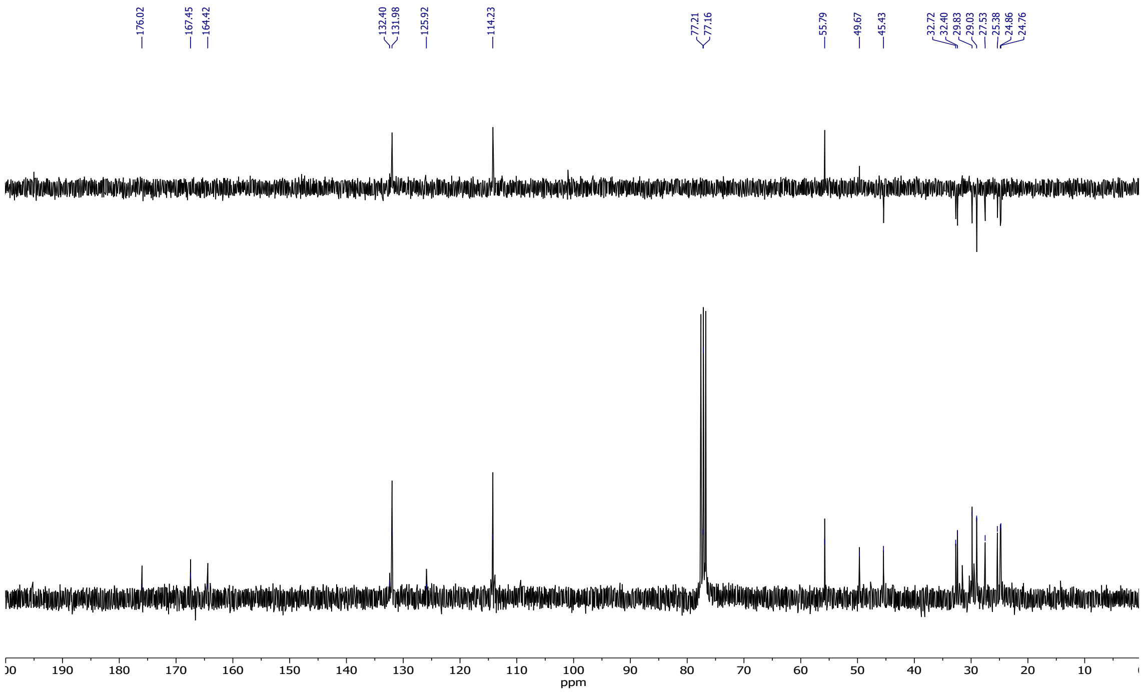

Figure S7. ^{13}C **and DEPT NMR spectra of 5b (75 MHz, $CDCl_3$).**

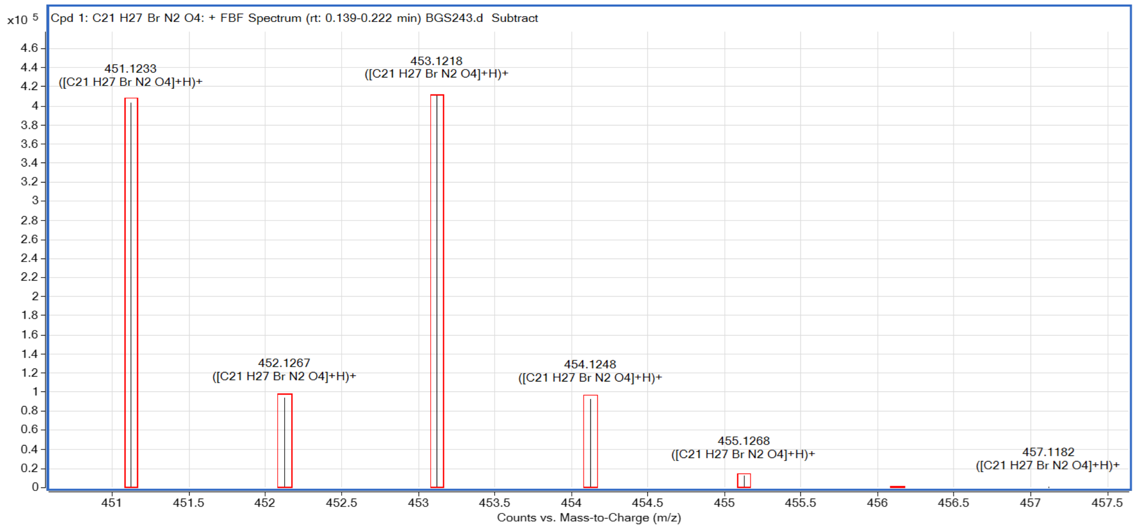

Figure S8. **HRMS spectrum of 5b.**

5-Benzoyl-1-(2-bromoethyl)-5-(*N*-*tert*-butylcarbamoyl)-2-pyrrolidinone (5c).

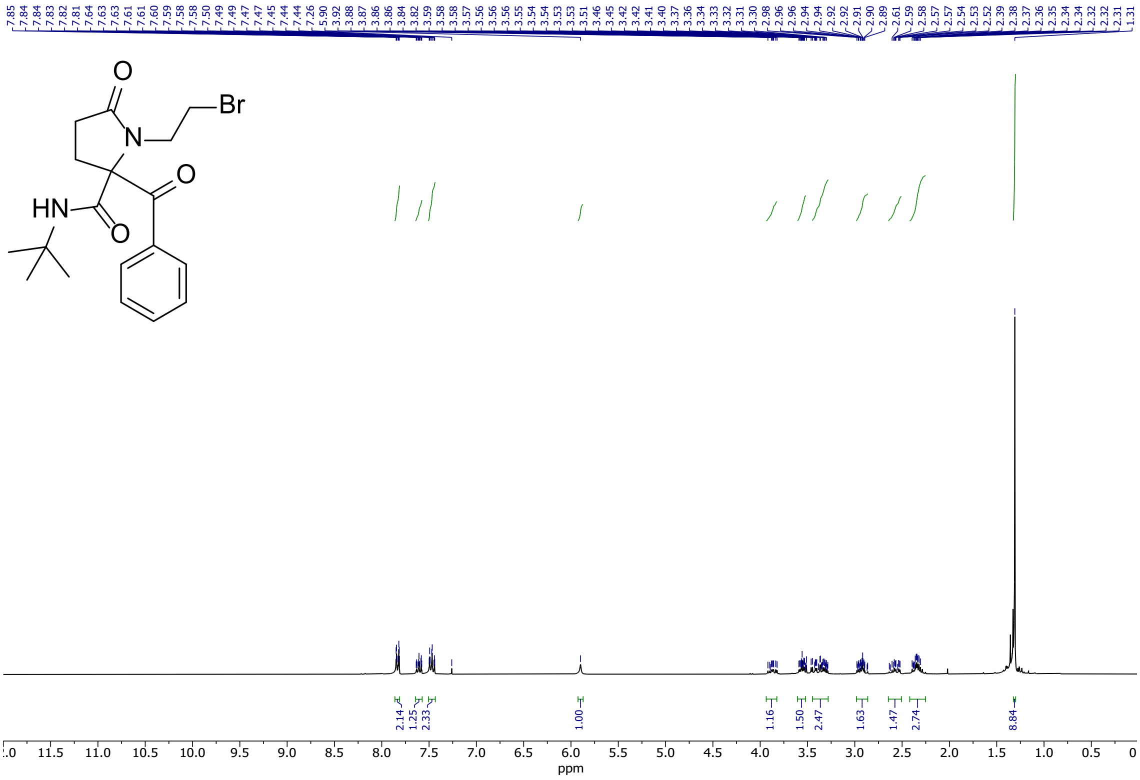

Figure S9. ^{1}H NMR spectrum of 5c (300 MHz, $CDCl_3$).

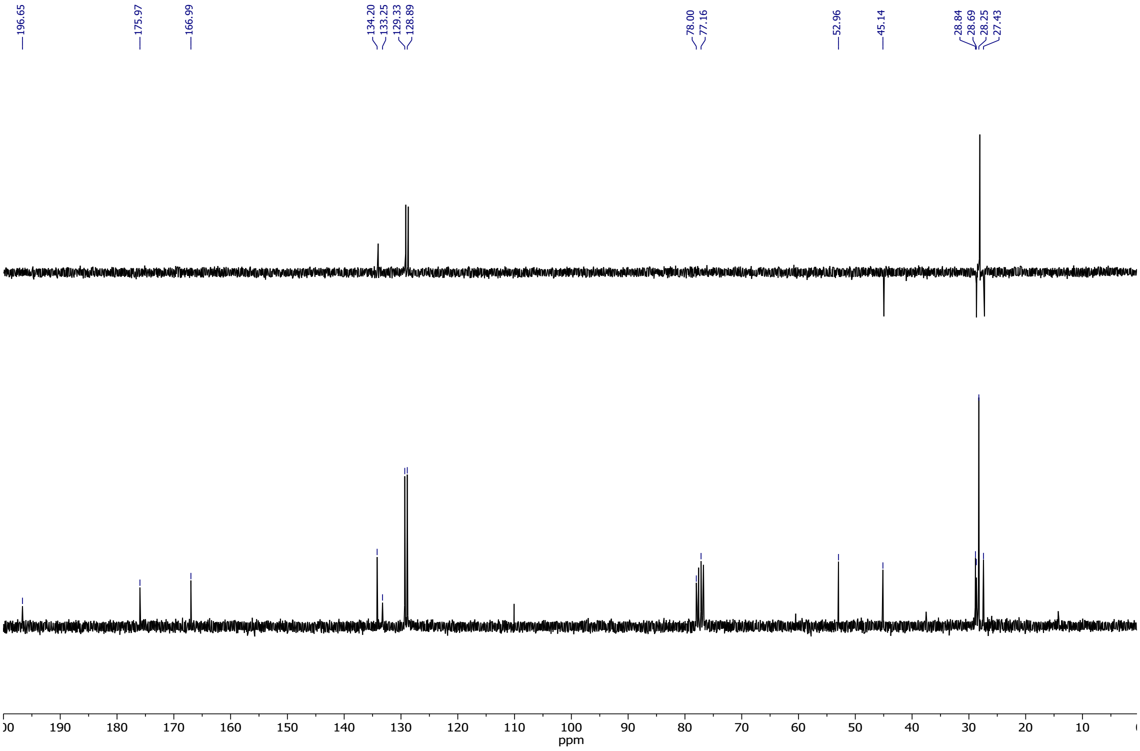

Figure S10. ^{13}C and DEPT NMR spectra of 5c (75 MHz, $CDCl_3$).

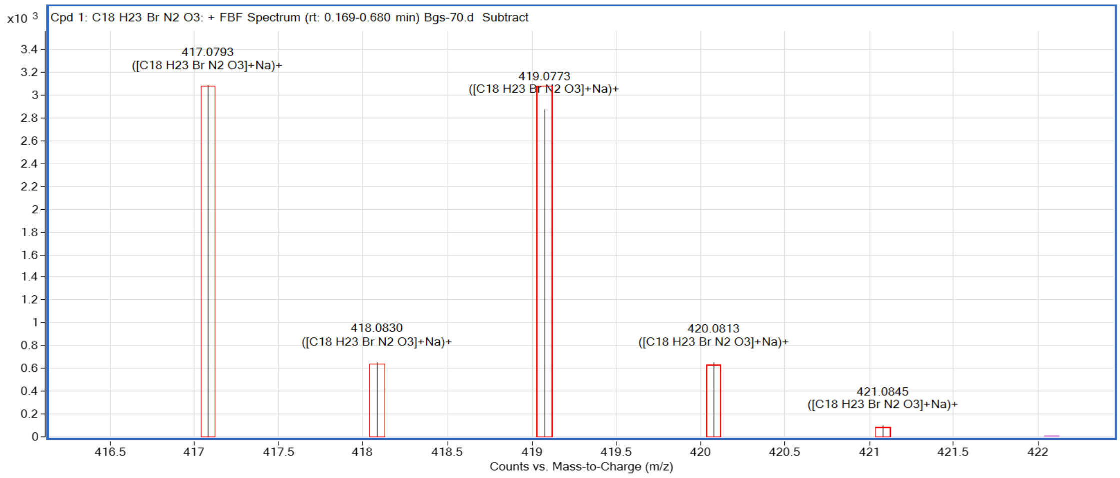

Figure S11. HRMS spectrum of 5c.

1-(2-Bromoethyl)-5-(*N*-*tert*-butylcarbamoyl)-5-(4-fluorobenzoyl)-2-pyrrolidinone (5d).

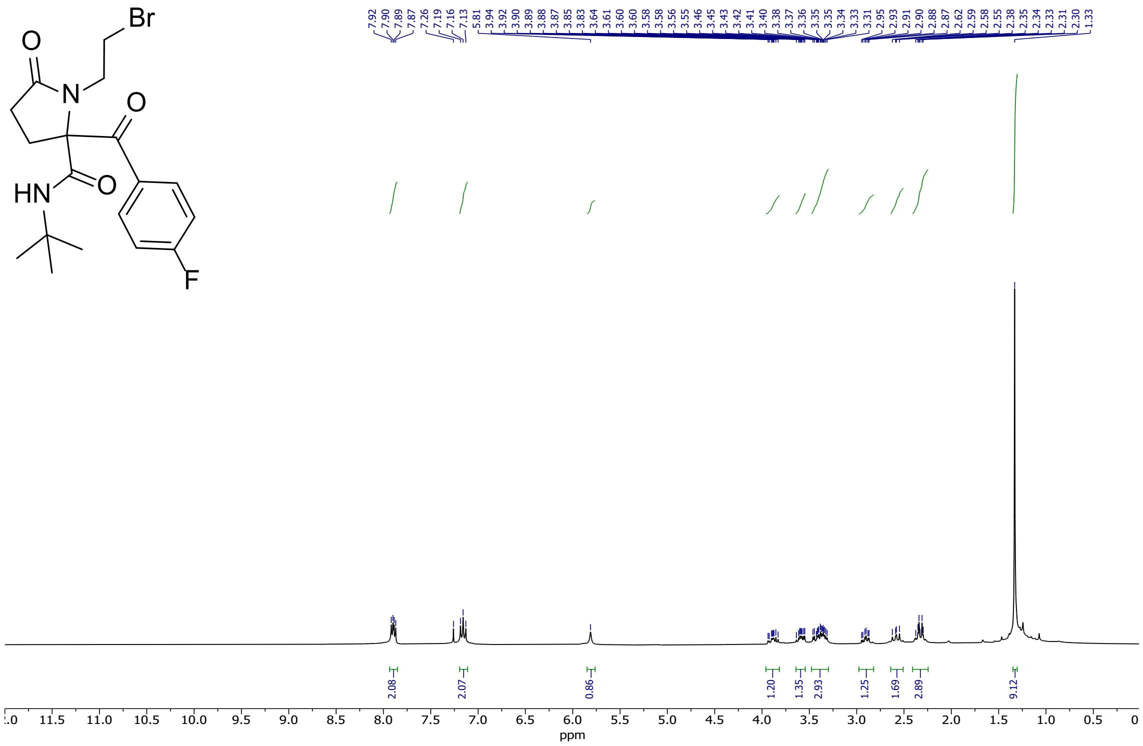

Figure S12. ^{1}H NMR spectrum of 5d (300 MHz, $CDCl_3$).

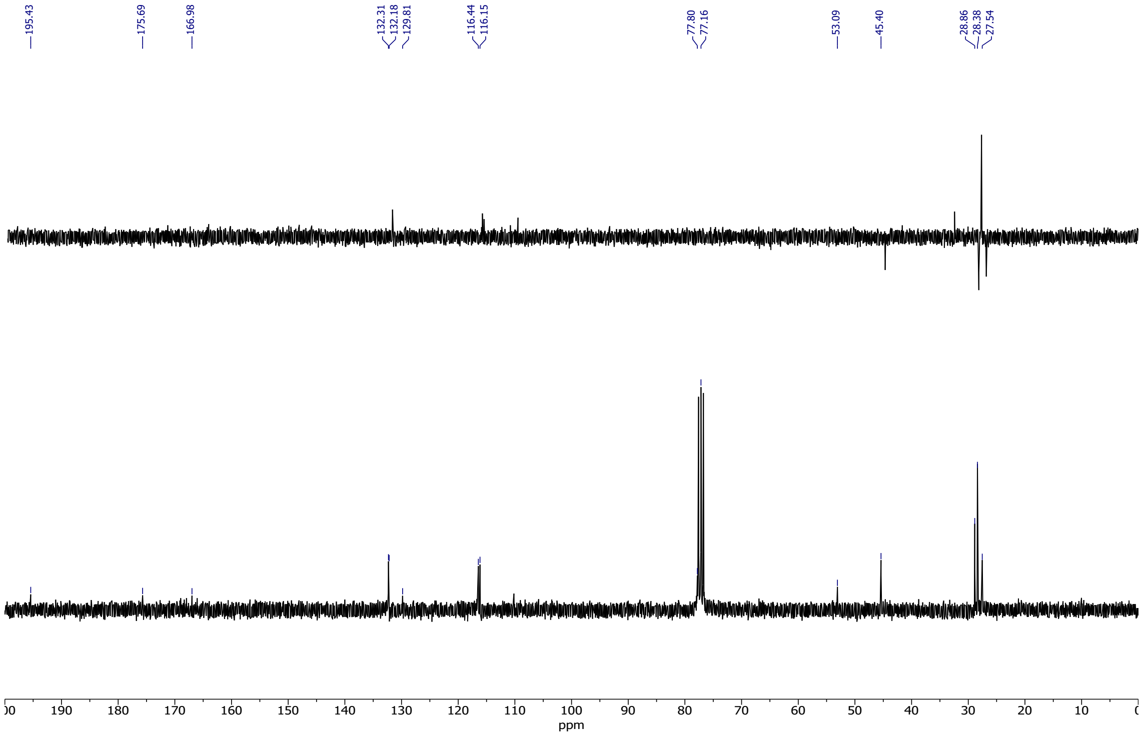

Figure S13. ^{13}C and DEPT NMR spectra of 5d (75 MHz, $CDCl_3$).

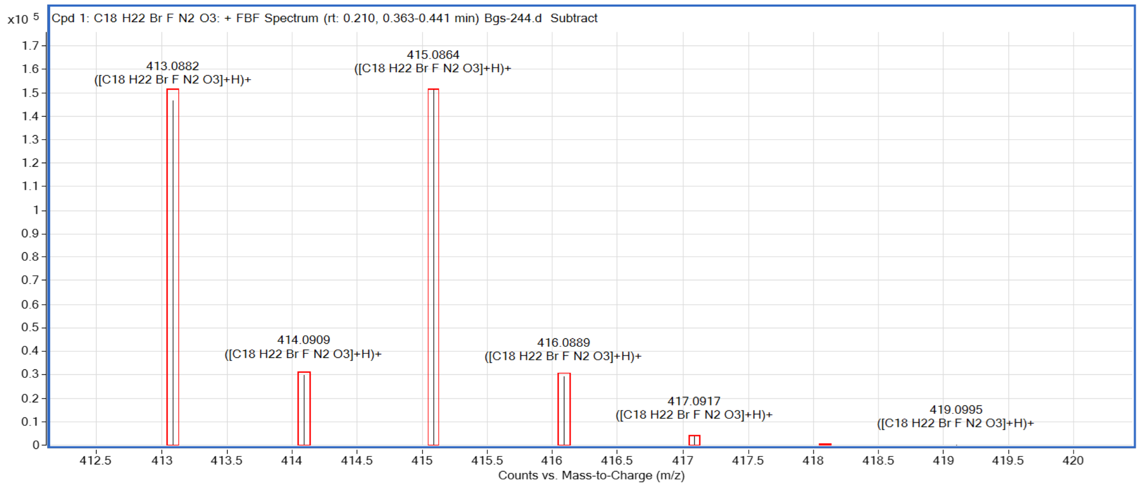

Figure S14. **HRMS spectrum of 5d.**

5-(*N*-Cyclohexylcarbamoyl)-2-(2-oxopyrrolidin-1-yl)ethyl benzoate (6a).

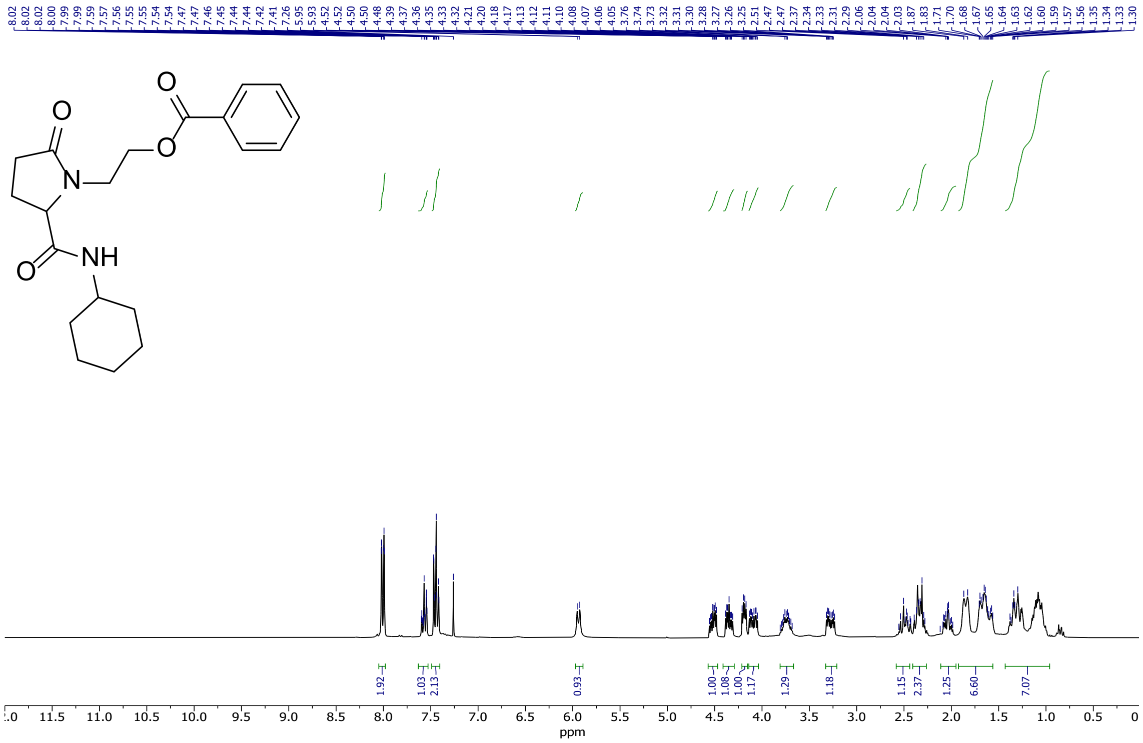

Figure S15. ^{1}H **NMR spectrum of 6a (300 MHz, $CDCl_3$).**

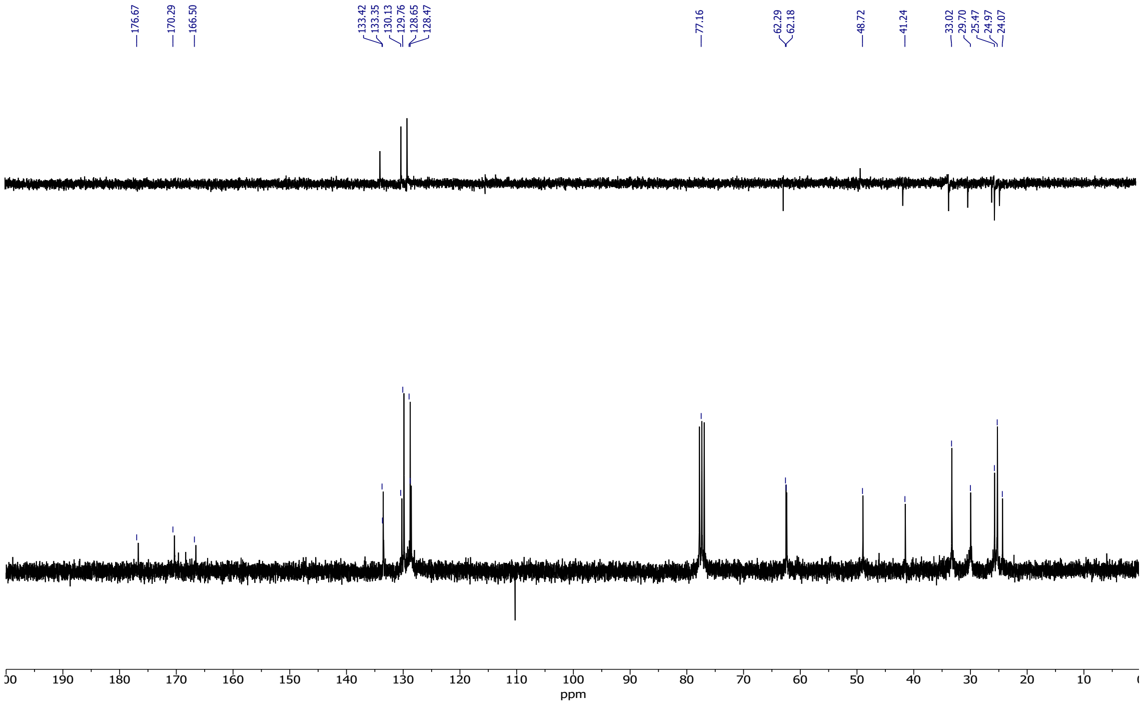

Figure S16. ^{13}C **and DEPT NMR spectra of 6a (75 MHz, $CDCl_3$).**

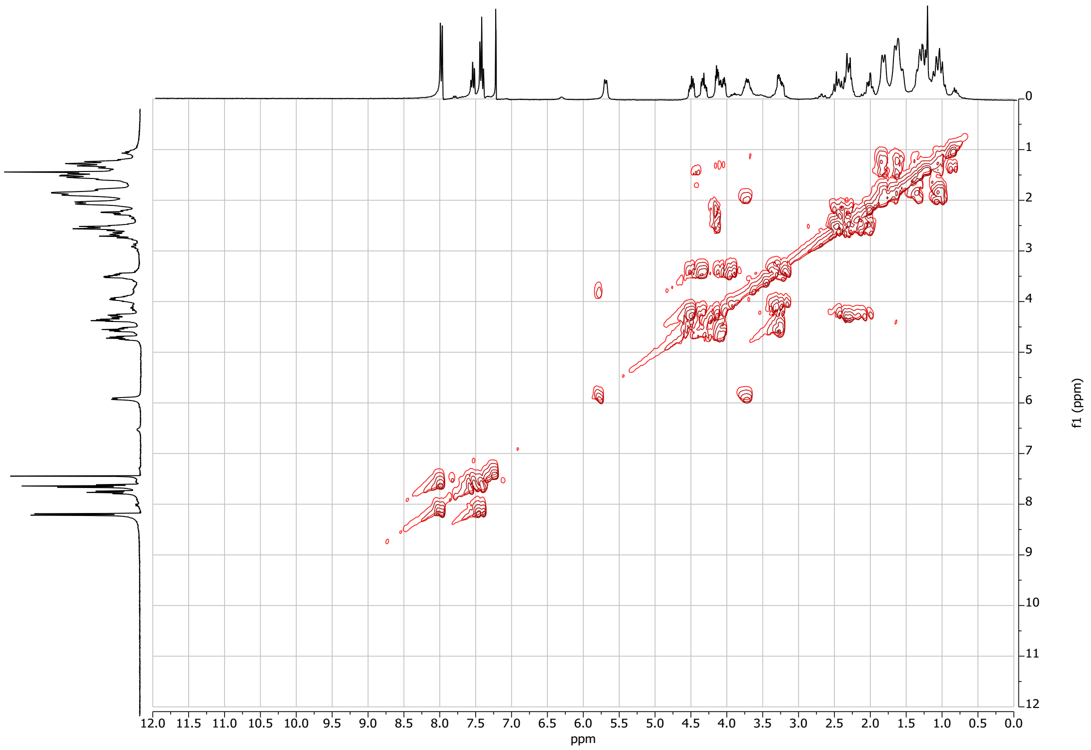

Figure S17. **COSY spectrum of 6a ($CDCl_3$).**

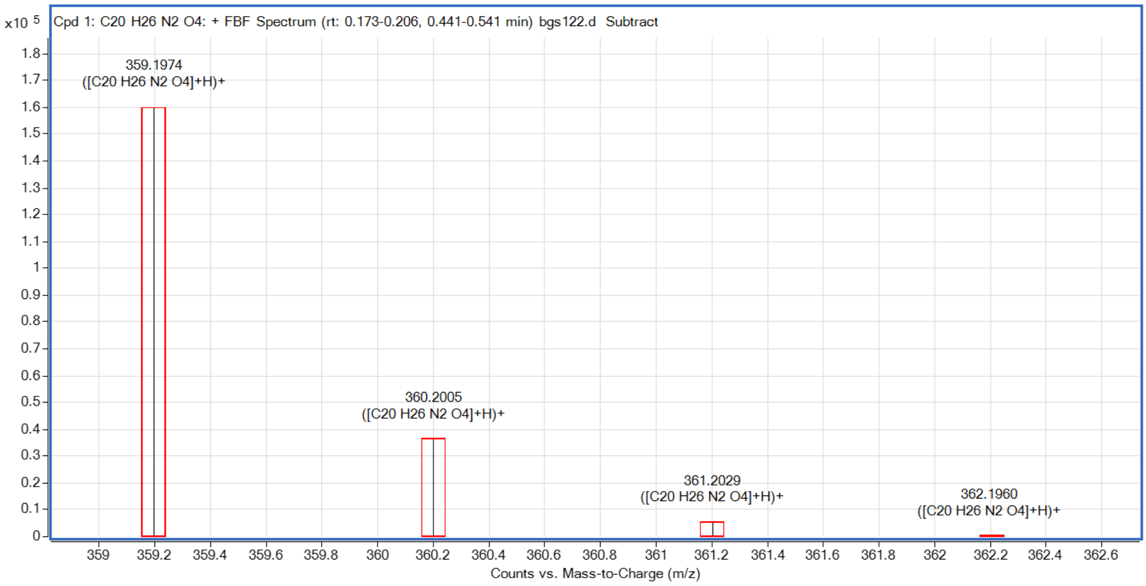

Figure S18. **HRMS spectrum of 6a.**

5-(*N*-Cyclohexylcarbamoyl)-2-(2-oxopyrrolidin-1-yl)ethyl 4-methoxybenzoate (6b).

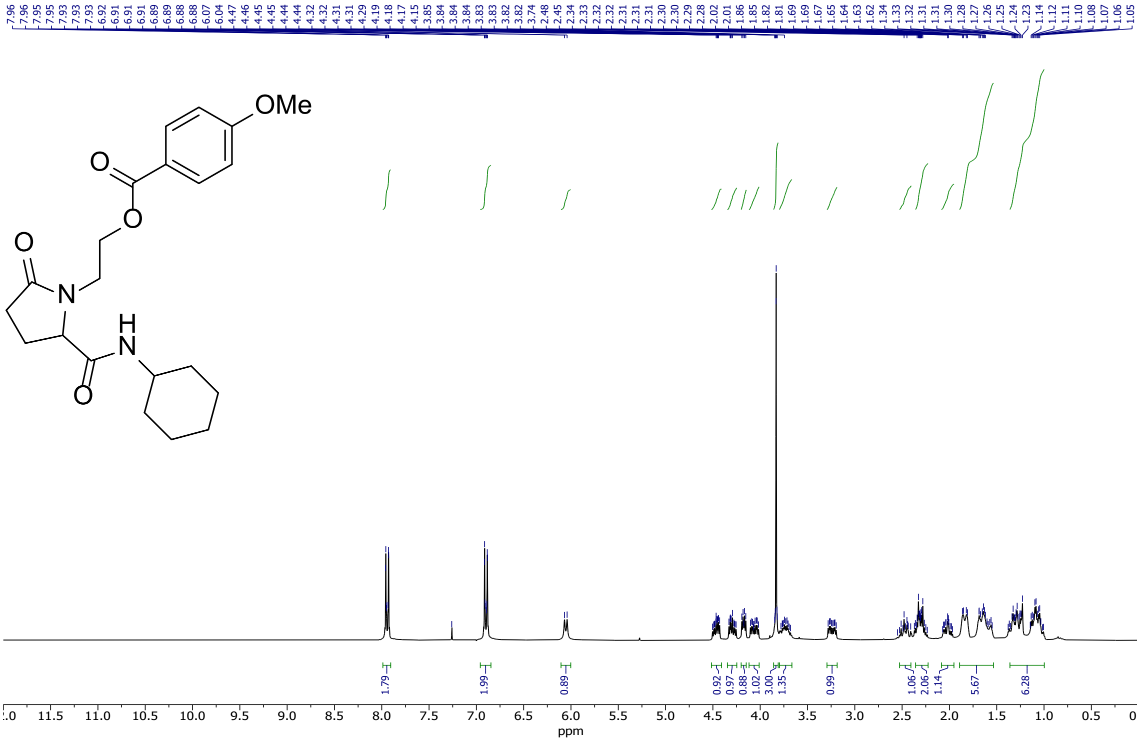

Figure S19. ^{1}H NMR spectrum of 6b (300 MHz, $CDCl_3$).

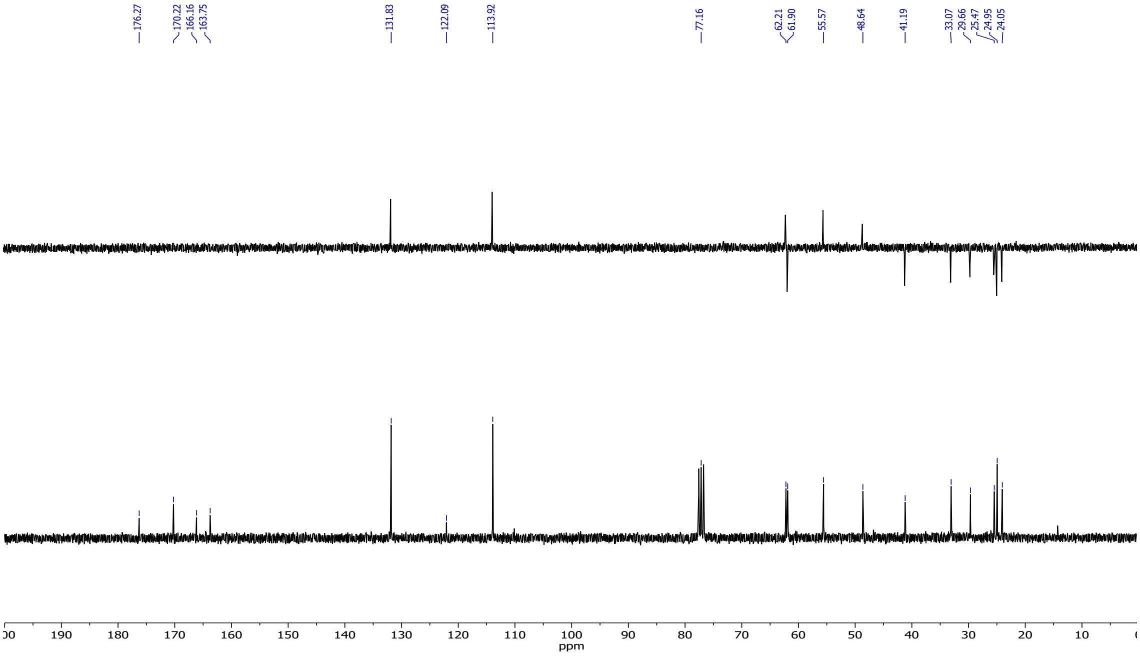

Figure S20. ^{13}C and DEPT NMR spectra of 6b (75 MHz, $CDCl_3$).

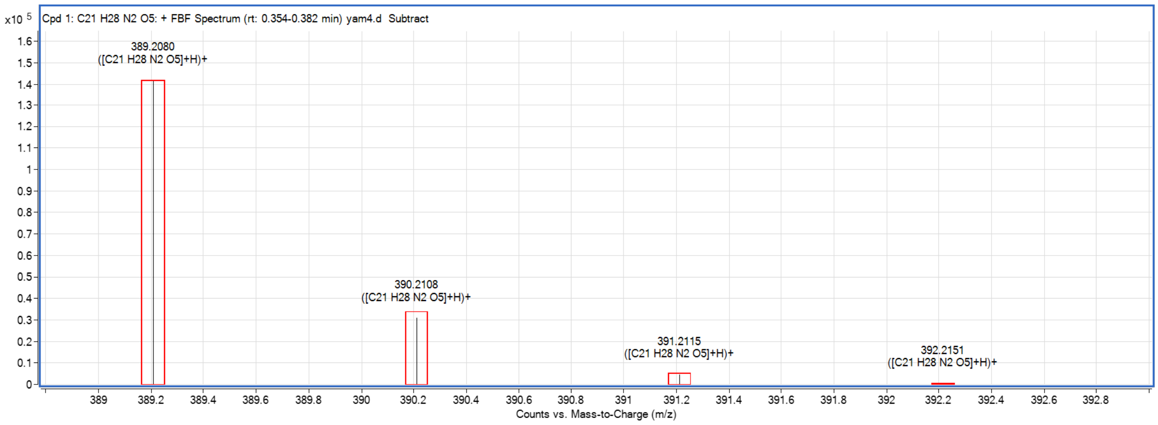

Figure S21. HRMS spectrum of 6b.

5-(*N-tert*-Butylcarbamoyl)-2-(2-oxopyrrolidin-1-yl)ethyl benzoate (6c).

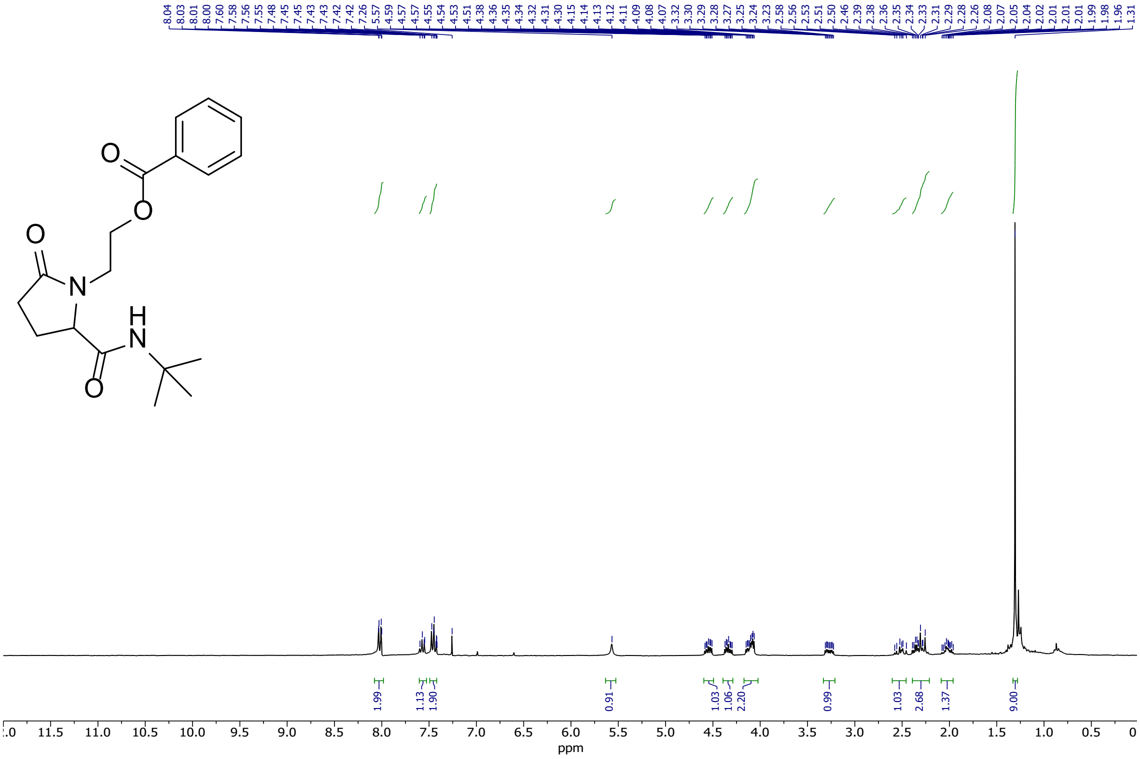

Figure S22. ^{1}H NMR spectrum of 6c (300 MHz, $CDCl_3$).

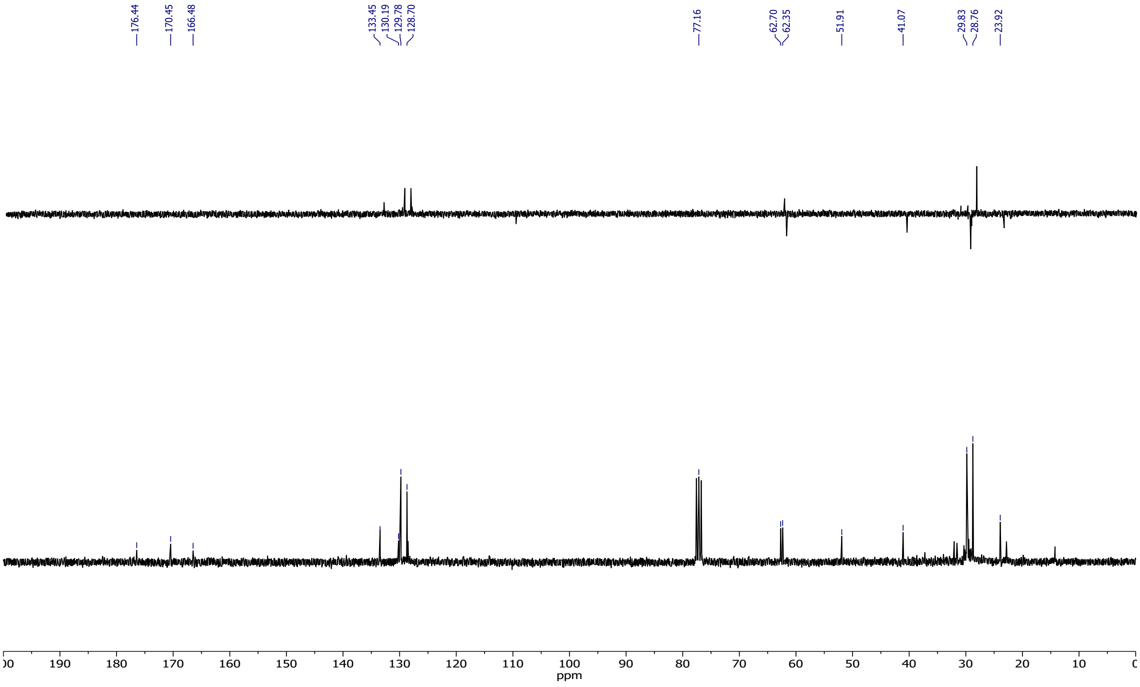

Figure S23. ^{13}C and DEPT NMR spectra of 6c (75 MHz, $CDCl_3$).

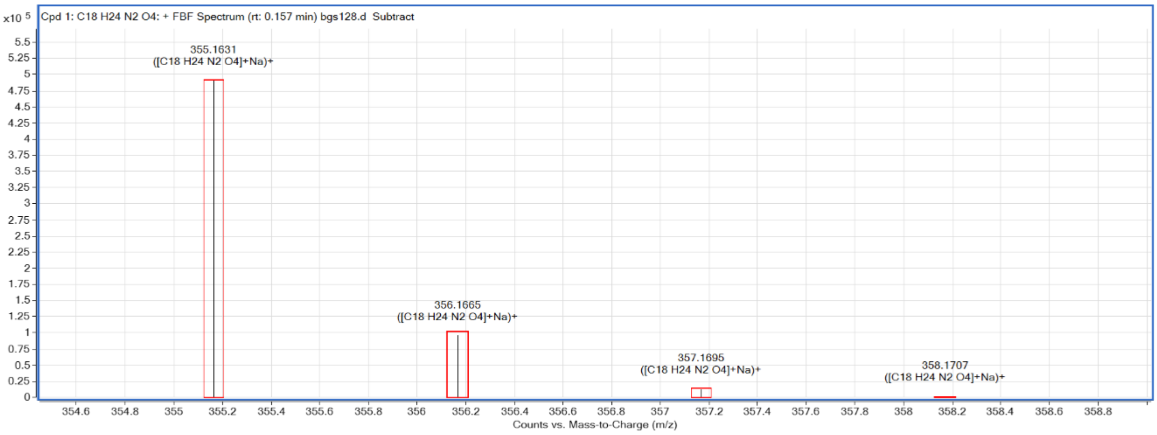

Figure S24. HRMS spectrum of 6c.

5-(*N-tert*-Butylcarbamoyl)-2-(2-oxopyrrolidin-1-yl)ethyl 4-fluorobenzoate (6d).

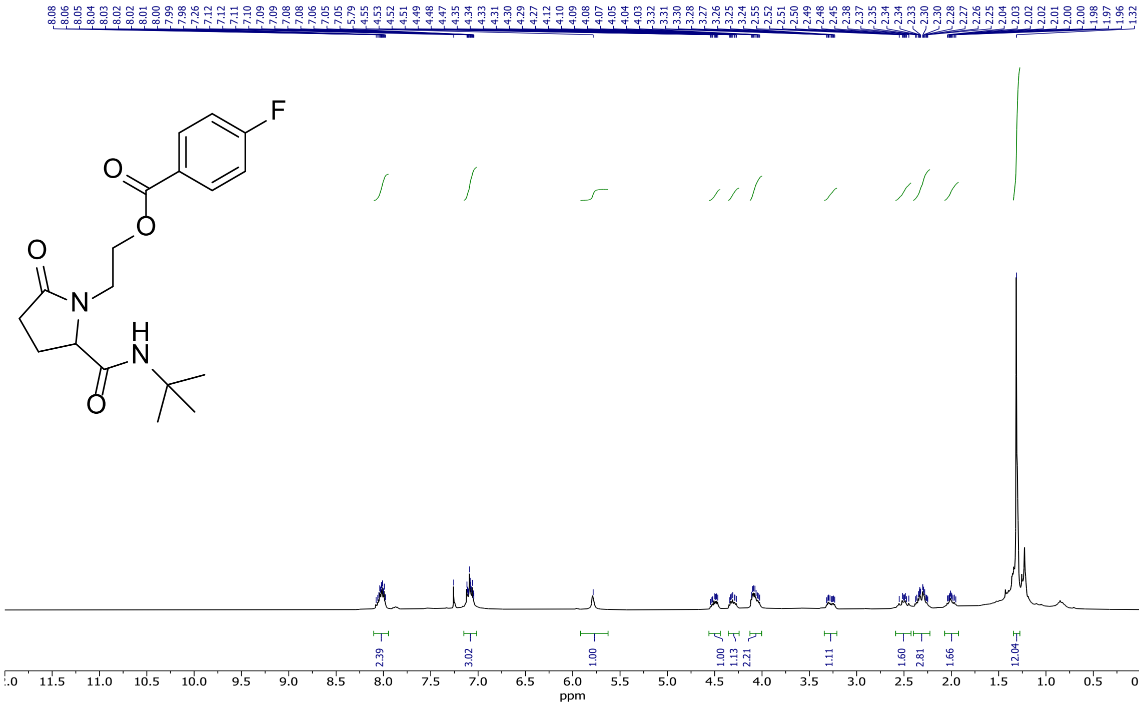

Figure S25. ^{1}H NMR spectrum of 6d (300 MHz, $CDCl_3$).

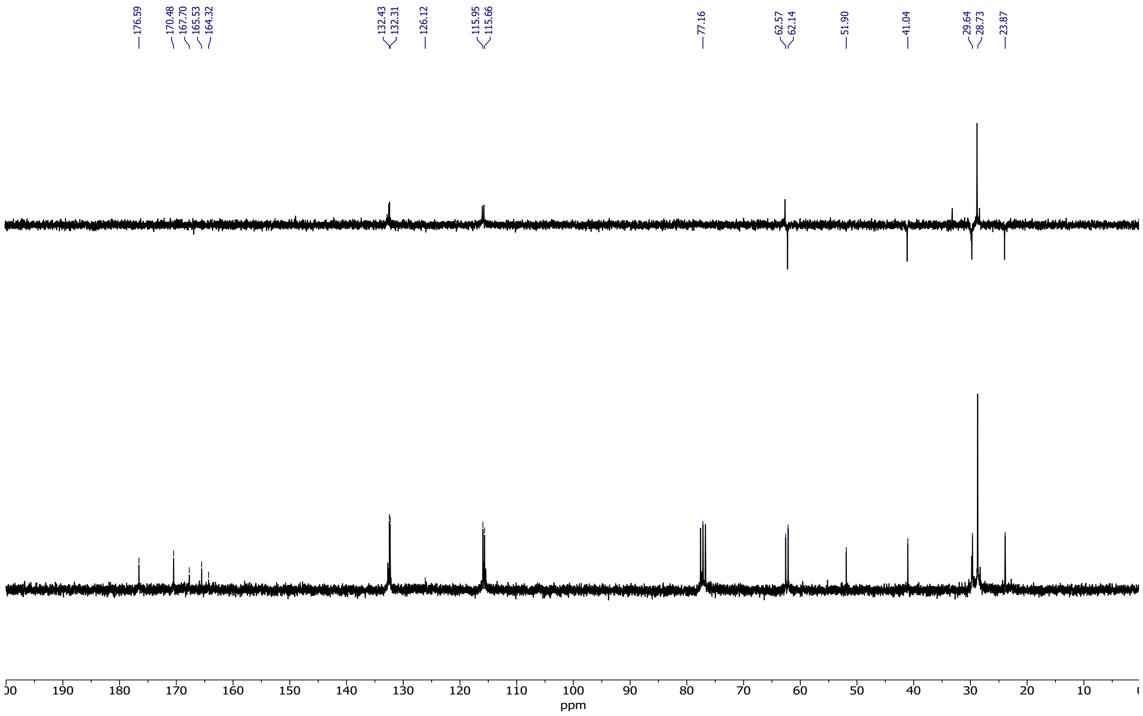

Figure S26. ^{13}C and DEPT NMR spectra of 6d (75 MHz, $CDCl_3$).

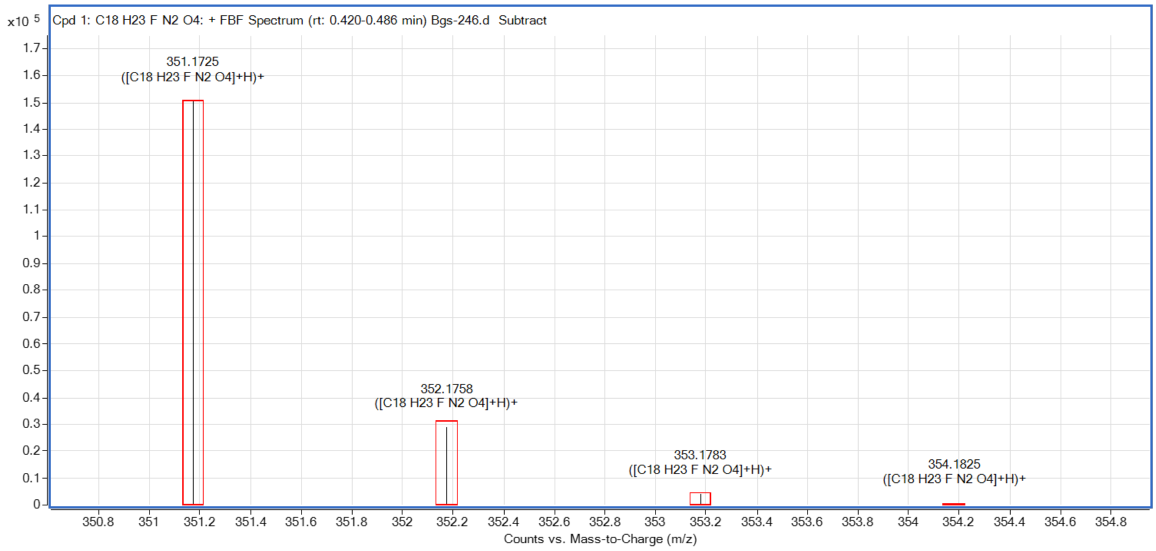

Figure S27. HRMS spectrum of 6d.

2-Cyclohexyltetrahydropyrrolo[1,2-*a*]pyrazine-1,6(2*H*,7*H*)-dione (8).

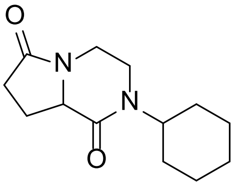

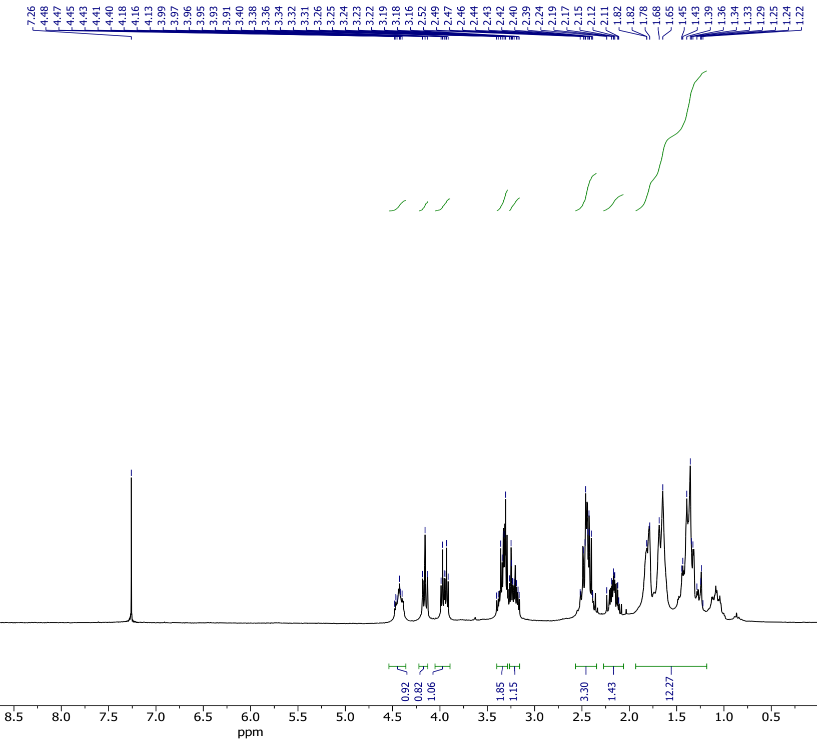

Figure S28. ^{1}H **NMR spectrum of 8 (300 MHz, $CDCl_3$).**

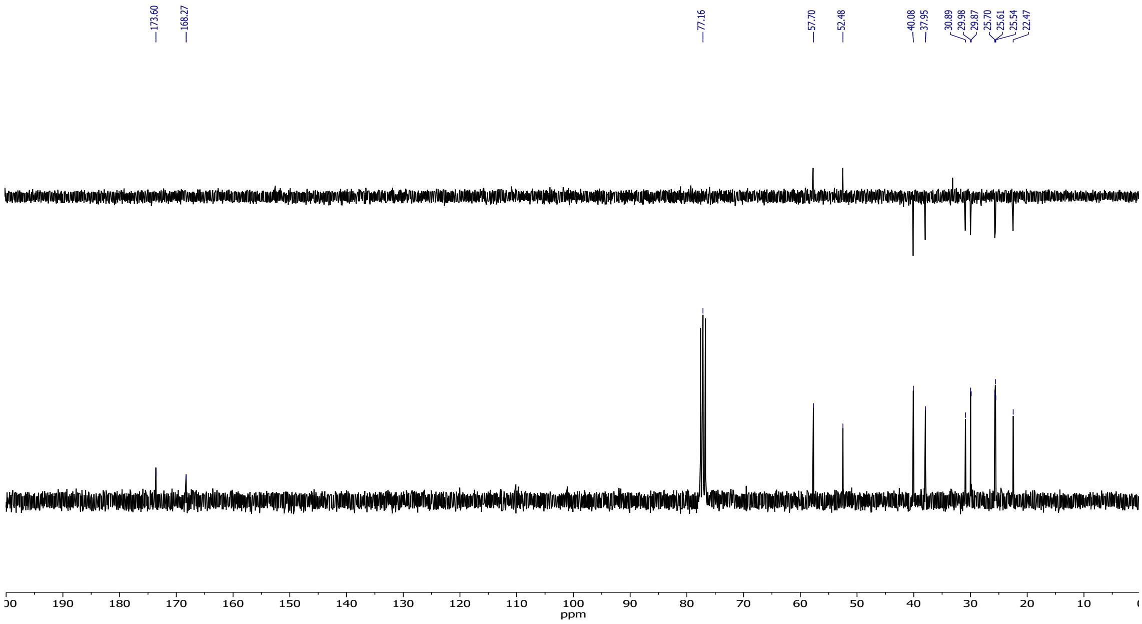

Figure S29. ^{13}C **and DEPT NMR spectra of 8 (75 MHz, $CDCl_3$).**

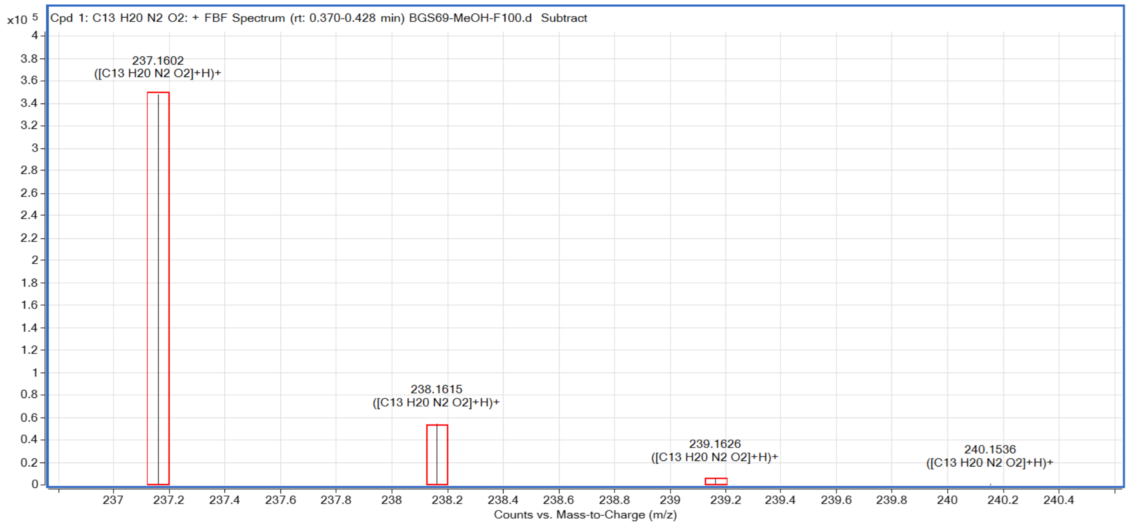

Figure S30. **HRMS spectrum of 8.**

(1*R,8a*R**)-8a-(*N*-Cyclohexylcarbamoyl)-1-ethoxy-1-phenylhexahydro-6*H*-pyrrolo[2,1-*c*][1,4]oxazin-6-one (9a).**

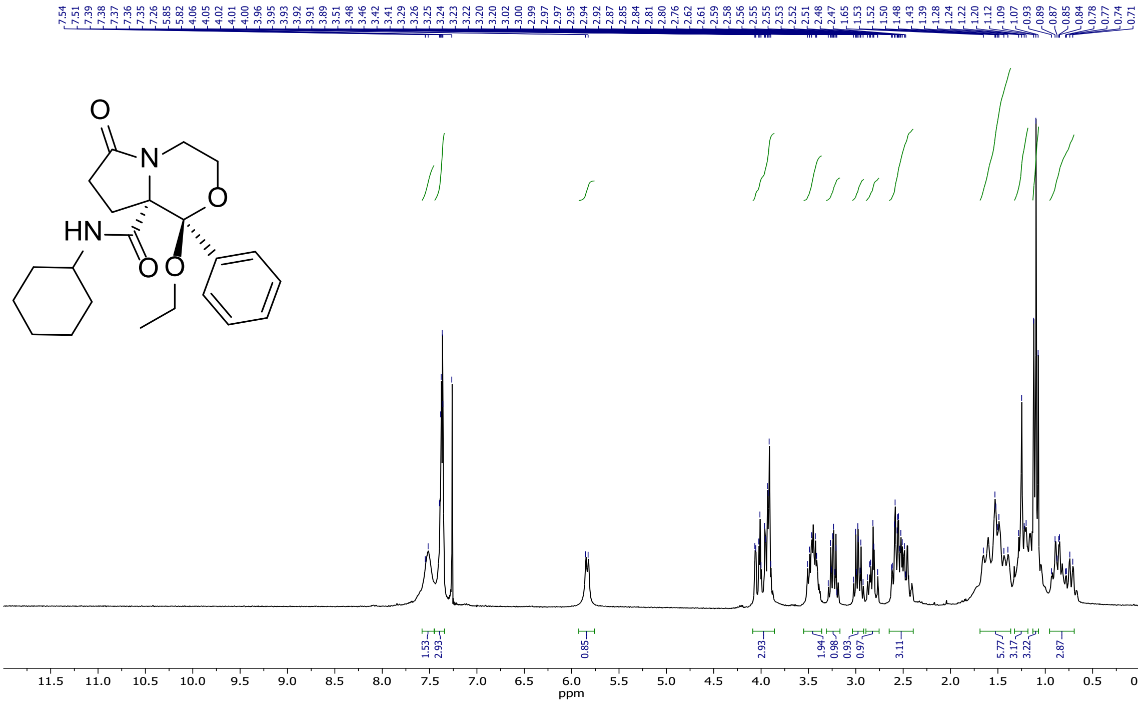

Figure S31. ^{1}H **NMR spectrum of 9a (300 MHz, CDCl$_3$).**

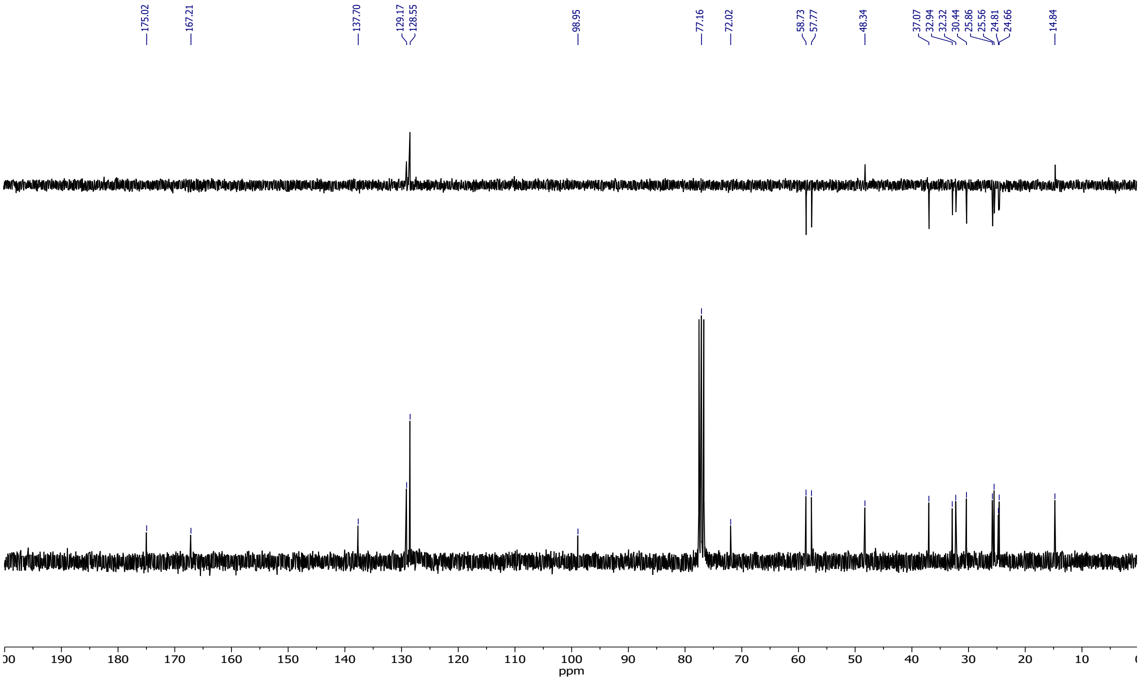

Figure S32. ^{13}C **and DEPT NMR spectra of 9a (75 MHz, CDCl$_3$).**

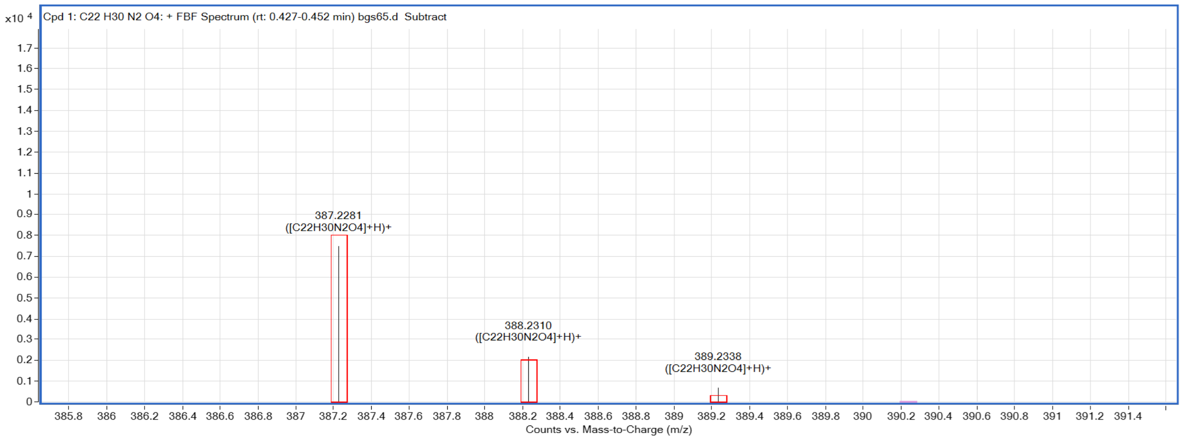

Figure S33. HRMS spectrum of 9a.

(1*R,8a*R**)-8a-(*N*-Cyclohexylcarbamoyl)-1-methoxy-1-phenylhexahydro-6*H*-pyrrolo[2,1-*c*][1,4]oxazin-6-one (9b).**

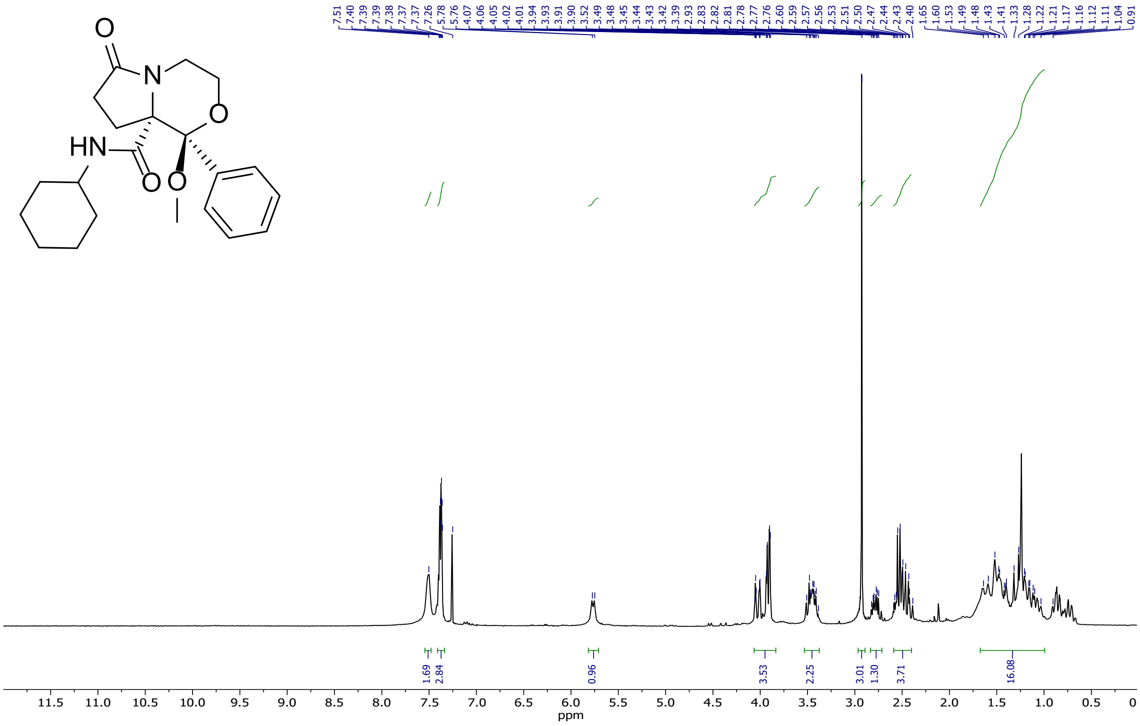

Figure S34. ^{1}H NMR spectrum of 9b (300 MHz, CDCl$_3$).

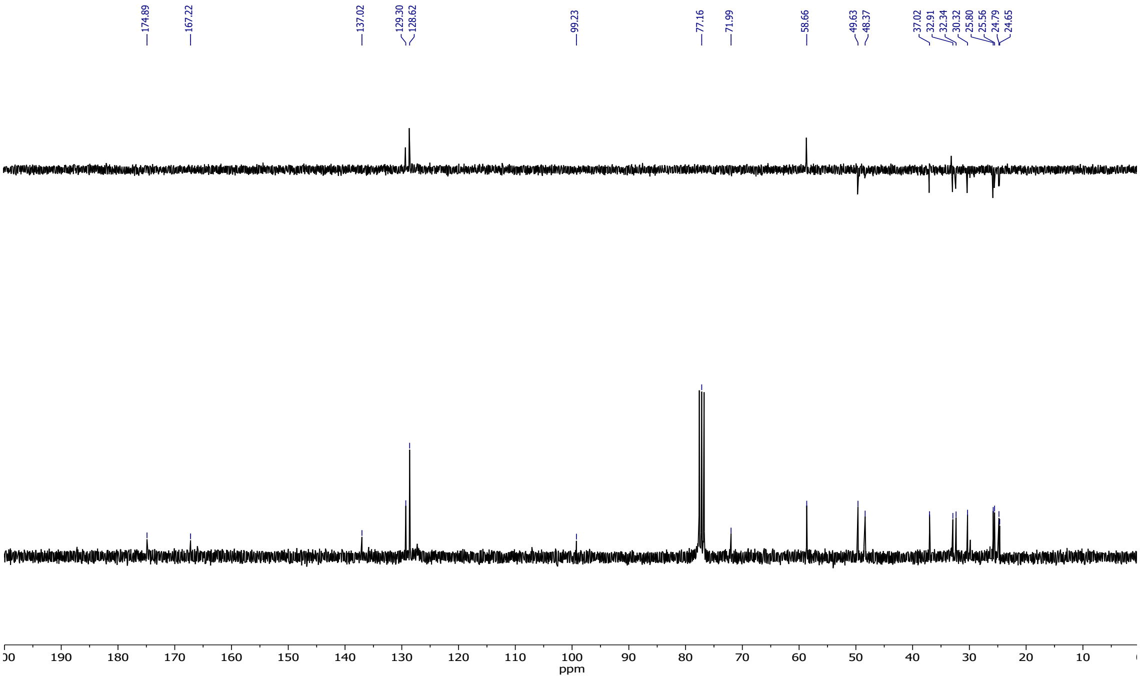

Figure S35. ^{13}C and DEPT NMR spectra of 9b (75 MHz, CDCl$_3$).

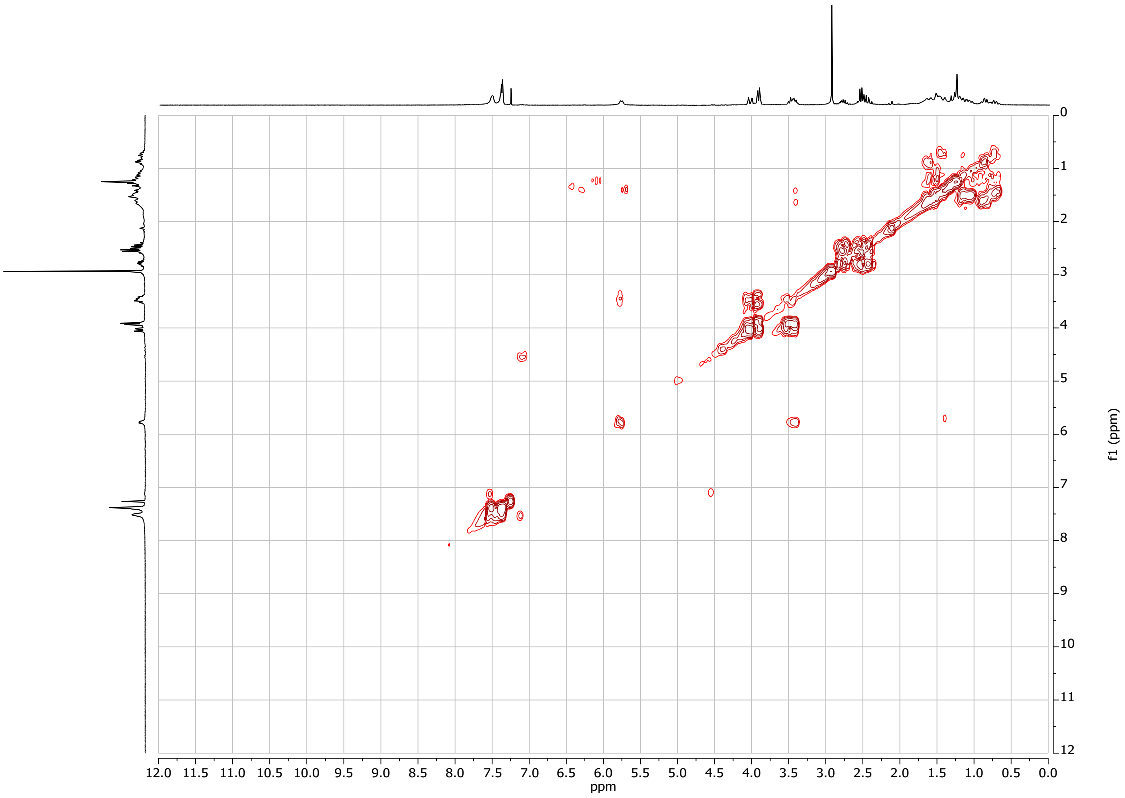

Figure S36. COSY spectrum of 9b ($CDCl_3$).

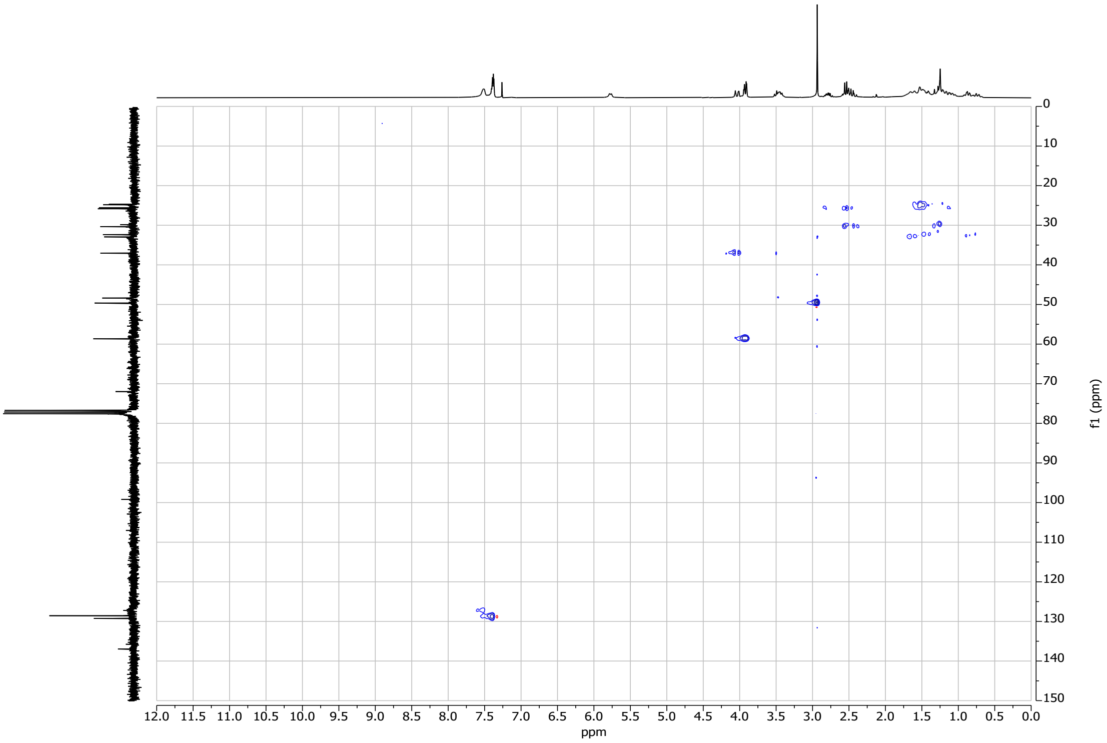

Figure S37. HMQC spectrum of 9b ($CDCl_3$).

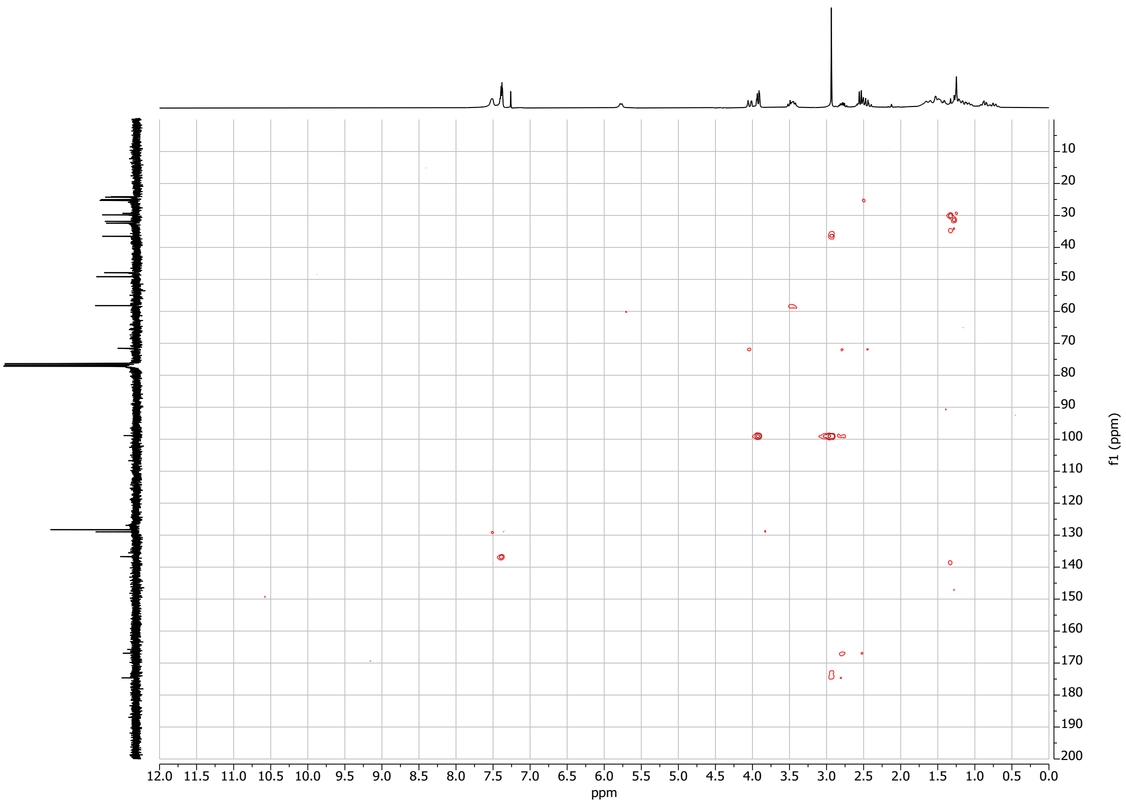

Figure S38. HMBC spectrum of 9b ($CDCl_3$).

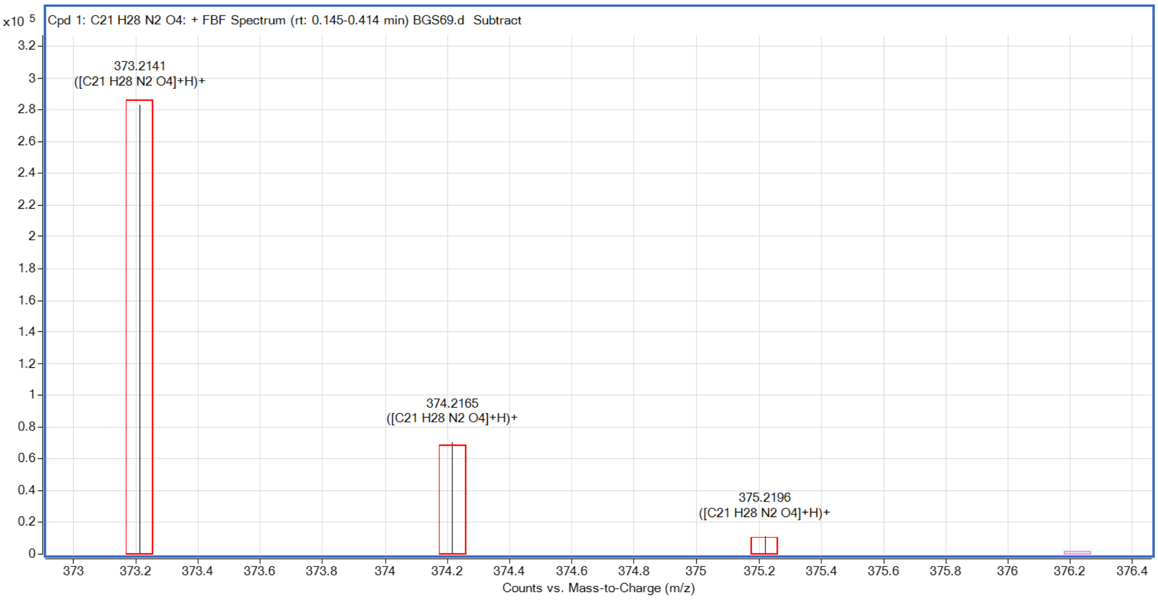

Figure S39. HRMS spectrum of 9b.

(1*R,8a*R**)-8a-(*N*-*tert*-Butylcarbamoyl)-1-ethoxy-1-phenylhexahydro-6*H*-pyrrolo[2,1-*c*][1,4]oxazin-6-one (9c).**

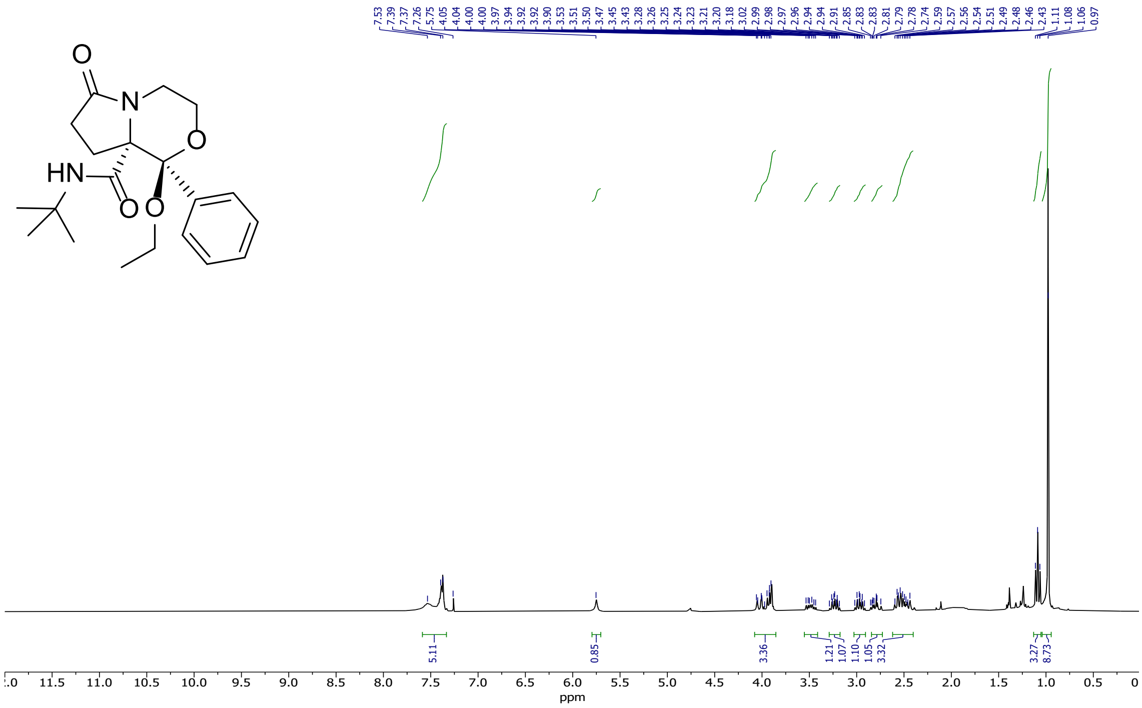

Figure S40. ^{1}H **NMR spectrum of 9c (300 MHz, CDCl$_3$).**

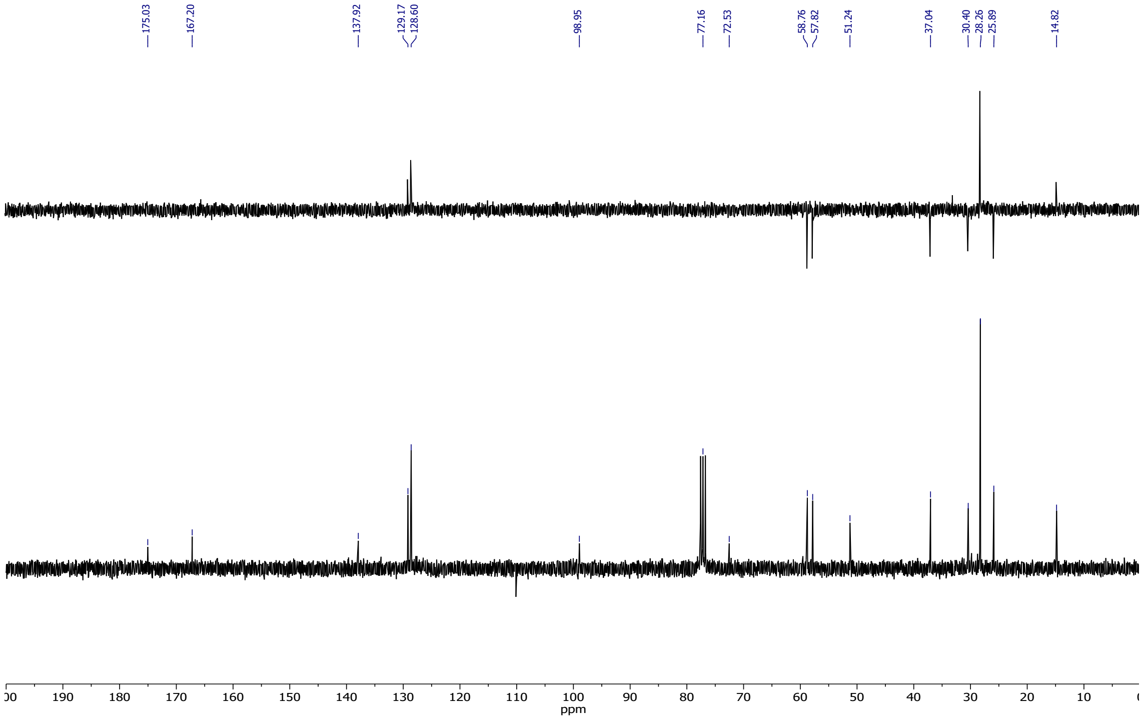

Figure S41. ^{13}C **and DEPT NMR spectra of 9c (75 MHz, CDCl$_3$).**

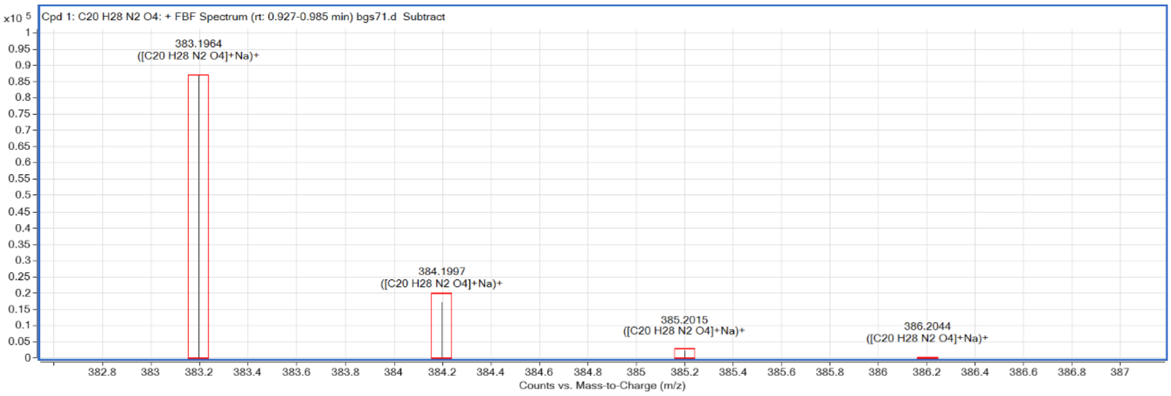

Figure S42. HRMS spectrum of 9c.

(1*R,8a*R**)-8a-(*N*-*tert*-Butylcarbamoyl)-1-methoxy-1-phenylhexahydro-6*H*-pyrrolo[2,1-*c*][1,4]oxazin-6-one (9d).**

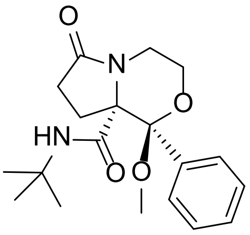

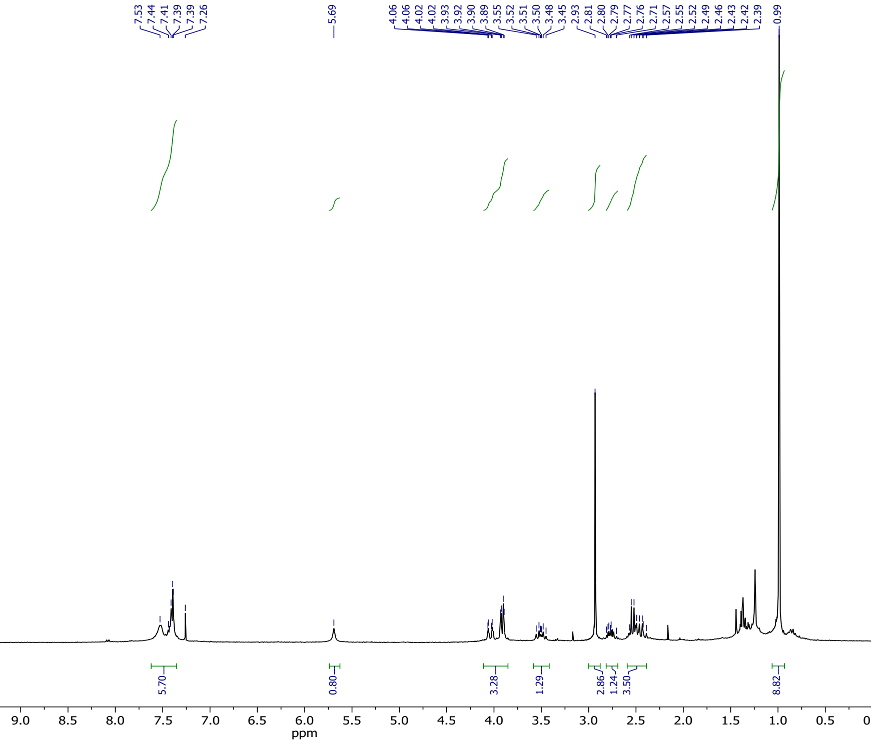

Figure S43. ^{1}H **NMR spectrum of 9d (300 MHz, CDCl$_3$).**

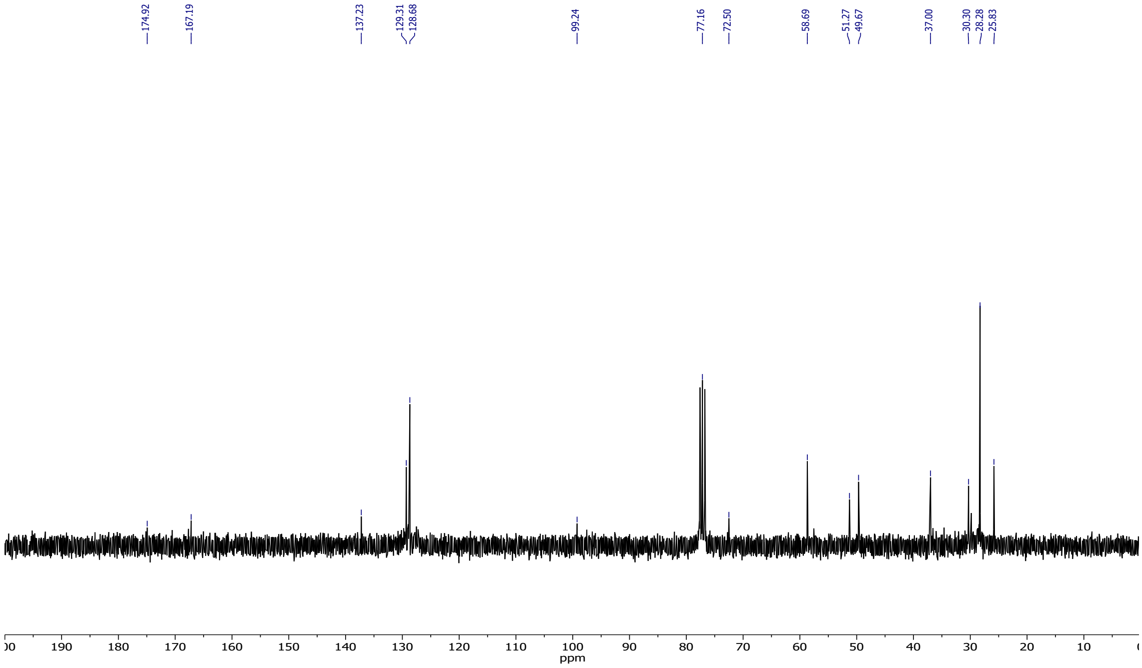

Figure S44. ^{13}C **NMR spectrum of 9d (75 MHz, CDCl$_3$).**

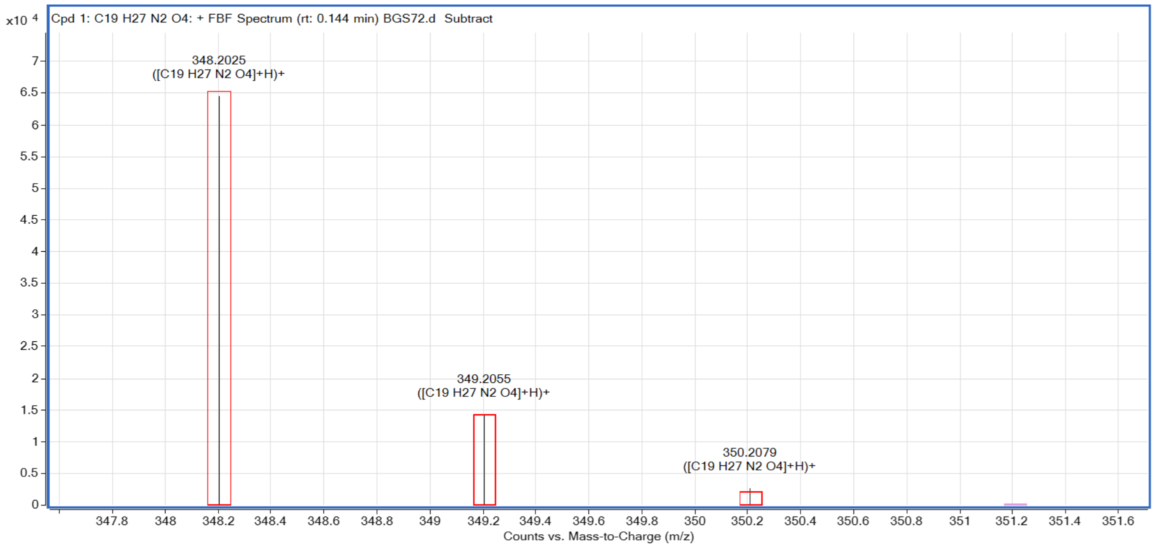

Figure S45. HRMS spectrum of 9d.

(1*R,8a*R**)-8a-(*N*-*tert*-Butylcarbamoyl)-1-ethoxy-1-(4-fluorophenyl)hexahydro-6*H*-pyrrolo[2,1-*c*][1,4]oxazin-6-one (9e).**

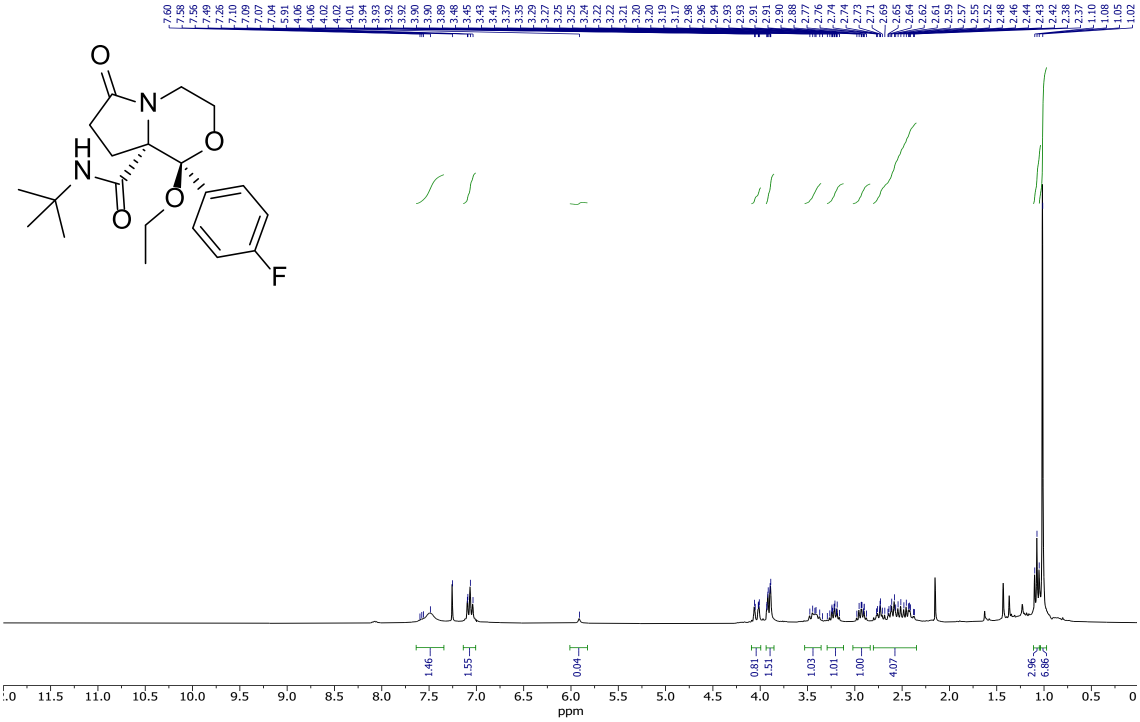

Figure S46. ^{1}H **NMR spectrum of 9e (300 MHz, CDCl$_3$).**

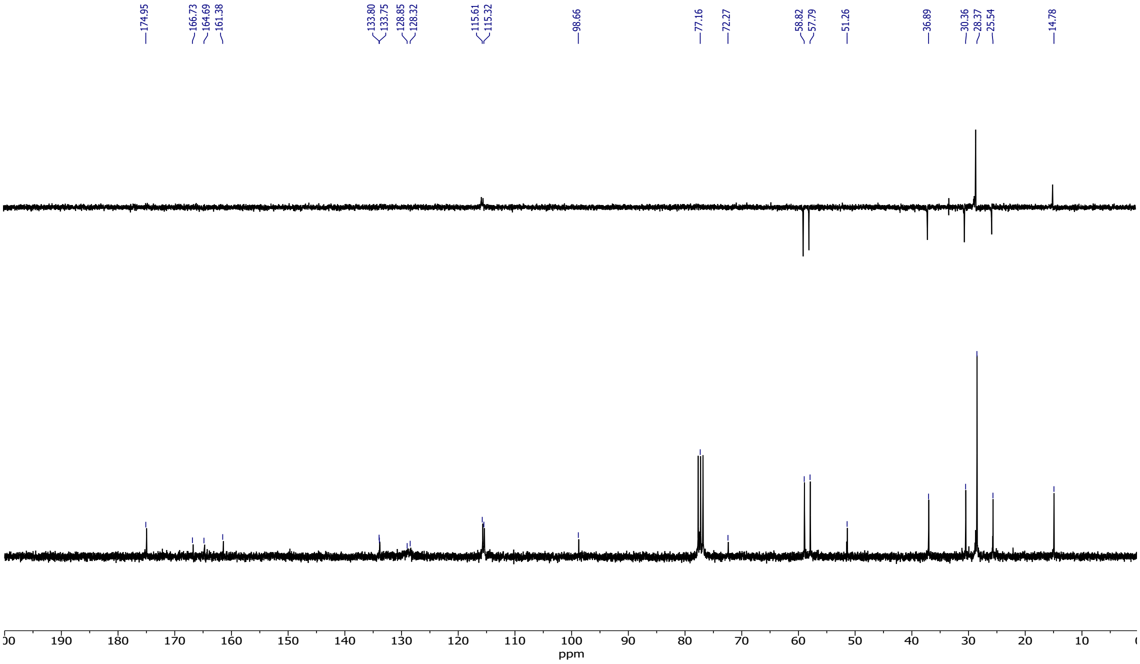

Figure S47. ^{13}C **and DEPT NMR spectra of 9e (75 MHz, CDCl$_3$).**

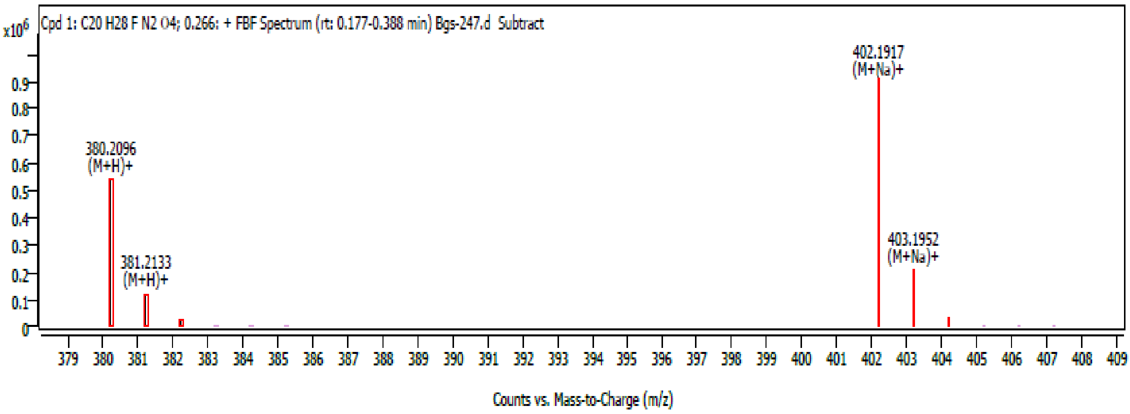

Figure S48. **HRMS spectrum of 9e.**

(1*R,8a*R**)-8a-(*N*-*tert*-Butylcarbamoyl)-1-(4-fluorophenyl)-1-methoxyhexahydro-6*H*-pyrrolo[2,1-*c*][1,4]oxazin-6-one (9f).**

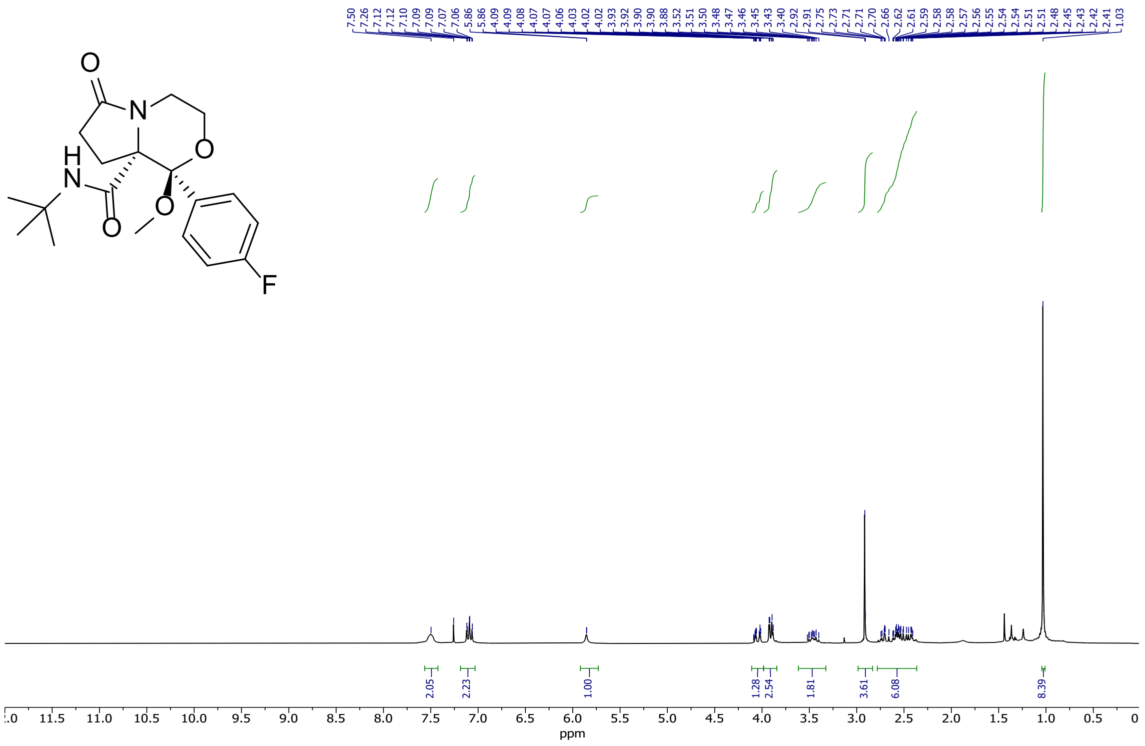

Figure S49. **^{1}H NMR spectrum of 9f (300 MHz, $CDCl_3$).**

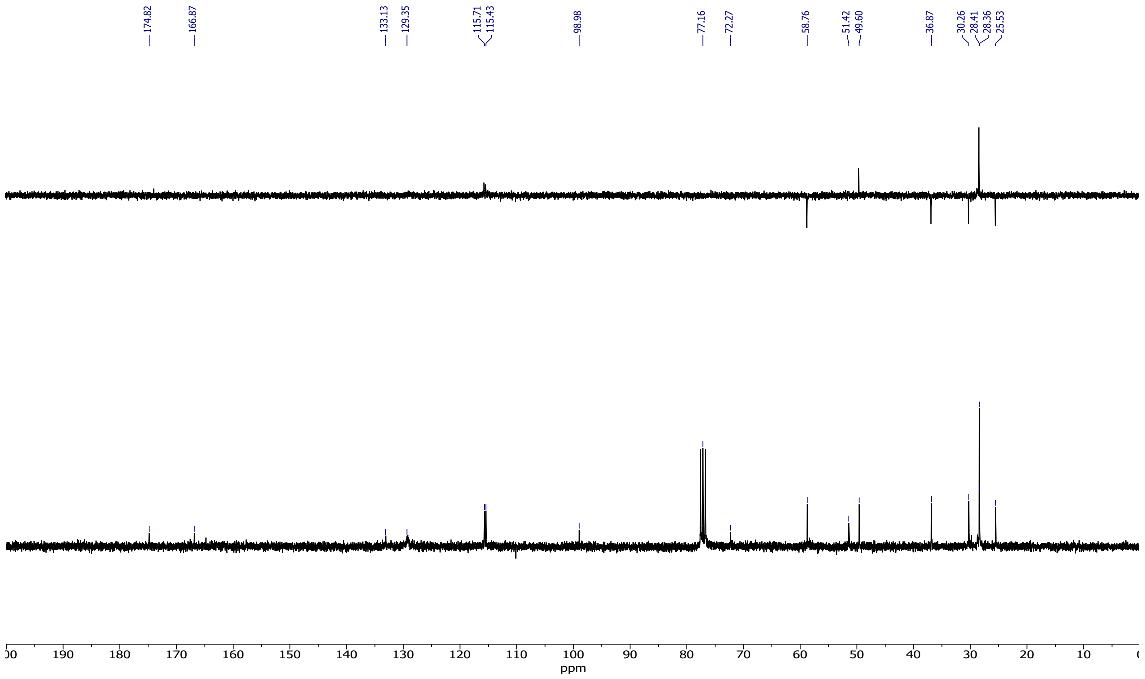

Figure S50. **^{13}C and DEPT NMR spectra of 9f (75 MHz, $CDCl_3$).**

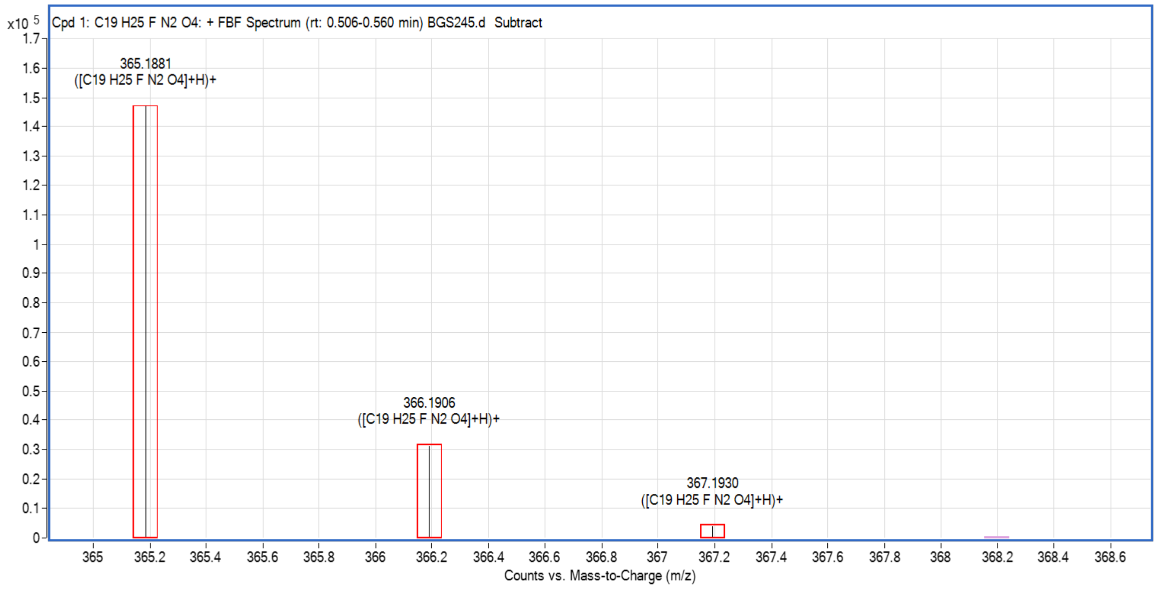

Figure S51. HRMS spectrum of 9f.

(*E*)-2-(*N*-Benzyl-2,3-dibromopropanamido)-*N*-cyclohexyl-3-hydroxy-3-phenylacrylamide (10).

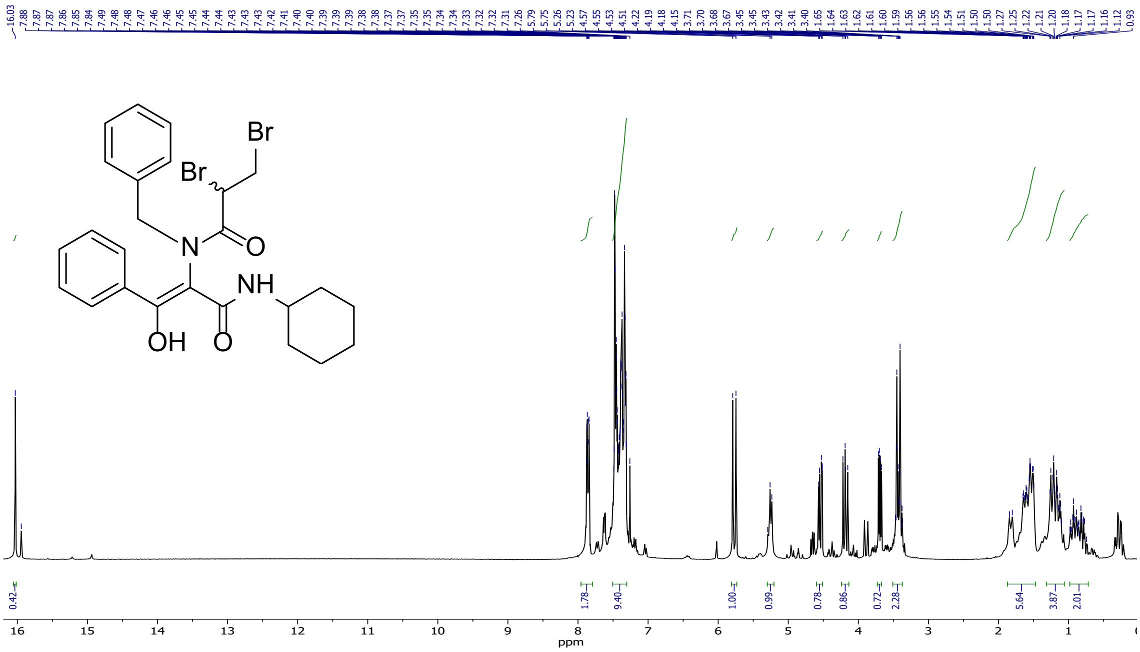

Figure S52. ^{1}H **NMR spectrum of 10 (300 MHz, $CDCl_3$).**

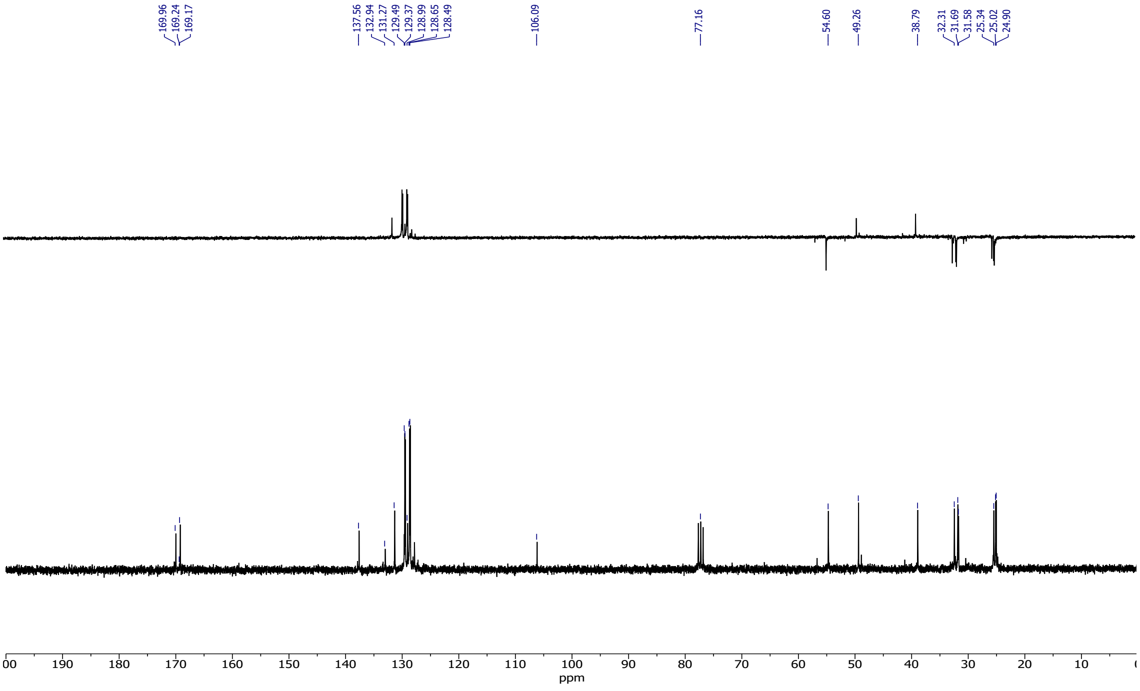

Figure S53. ^{13}C **and DEPT NMR spectra of 10 (75 MHz, $CDCl_3$).**

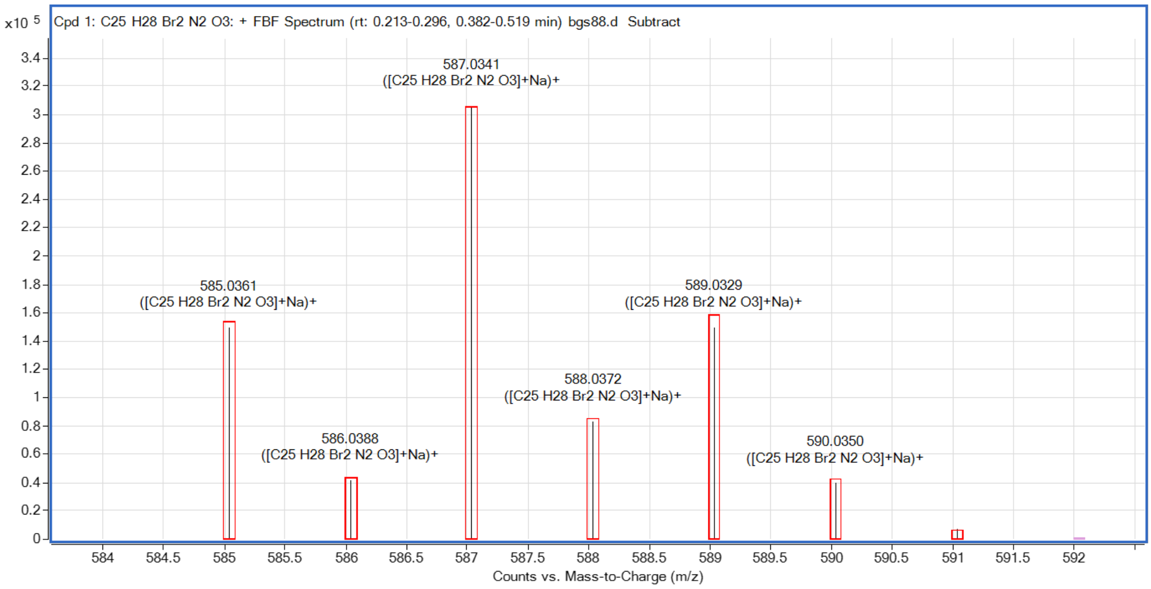

Figure S54. **HRMS spectrum of 10.**

5-Benzoyl-1-benzyl-3-bromo-5-(*N*-cyclohexylcarbamoyl)-2-pyrrolidinone (11a).

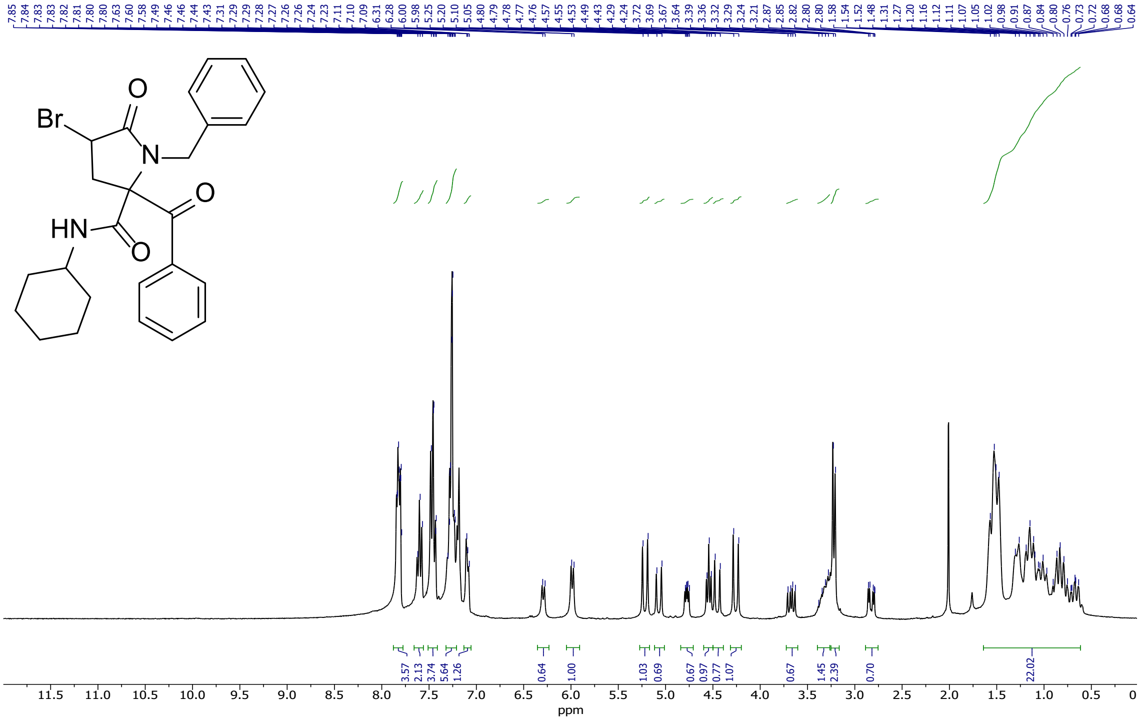

Figure S55. **^{1}H NMR spectrum of 11a, mixture of diastereomers (300 MHz, $CDCl_3$).**

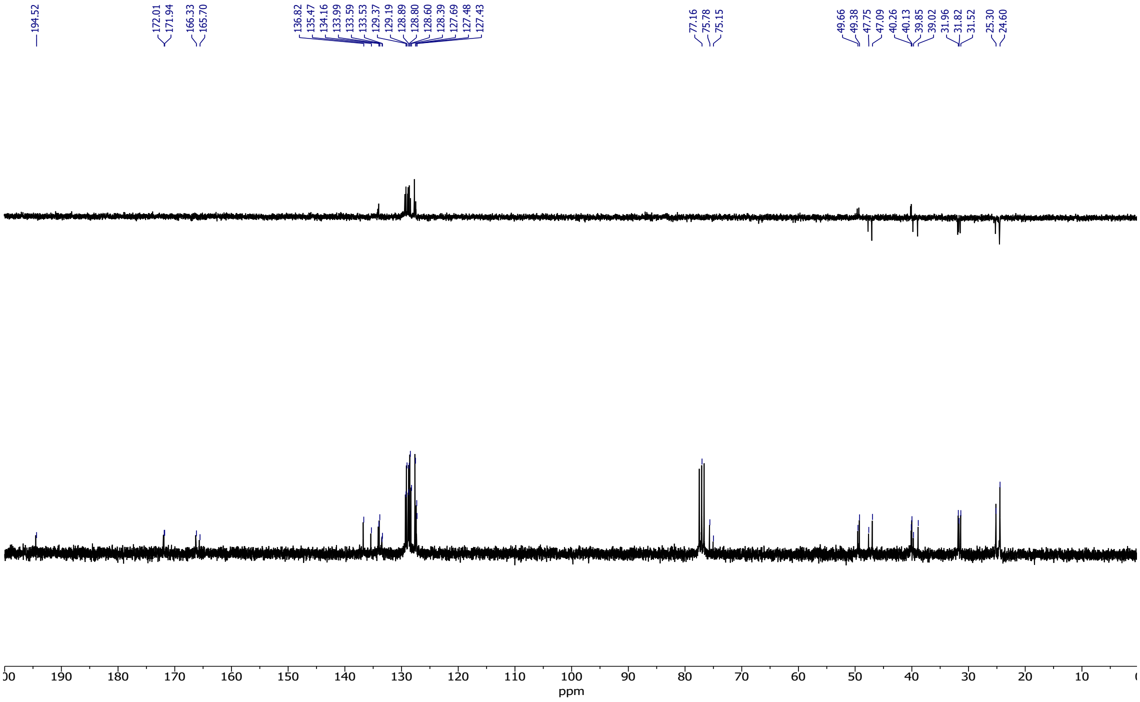

Figure S56. **^{13}C and DEPT NMR spectra of 11a, mixture of diastereomers (75 MHz, $CDCl_3$).**

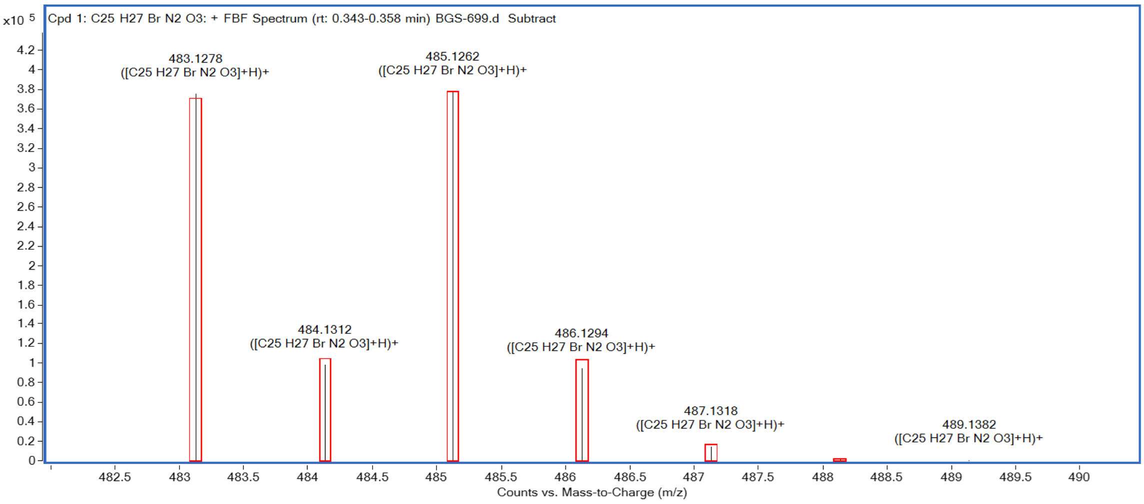

Figure S57. **HRMS spectrum of 11a.**

(3*R,5*S**)-1-Benzyl-3-bromo-5-(*N*-cyclohexylcarbamoyl)-5-(4-fluorobenzoyl)-2-pyrrolidinone (11b, diastereomer 1).**

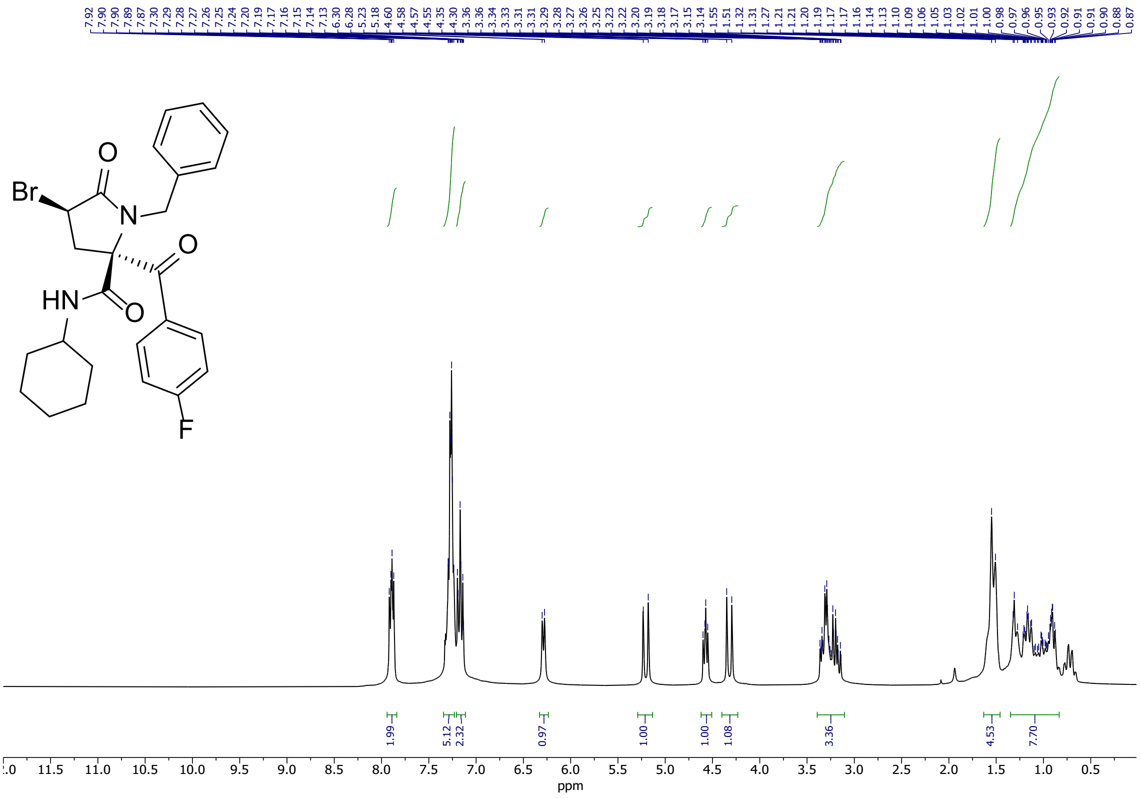

Figure S58. ^{1}H **NMR spectrum of 11b, diastereomer 1 (300 MHz, $CDCl_3$).**

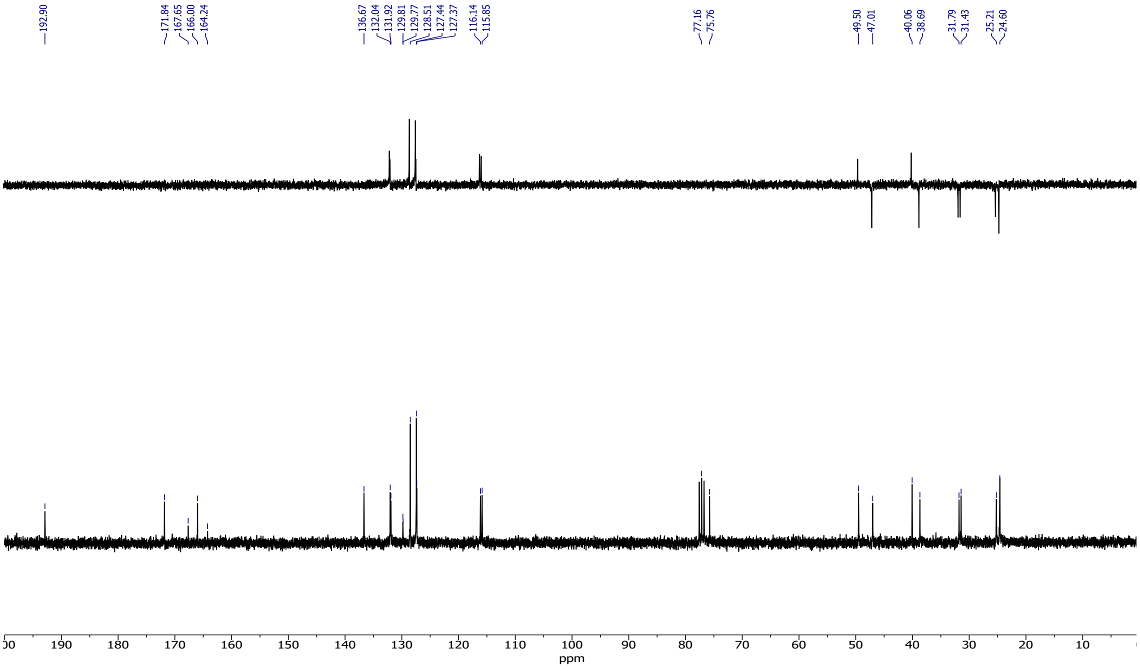

Figure S59. ^{13}C **and DEPT NMR spectra of 11b, diastereomer 1 (75 MHz, $CDCl_3$).**

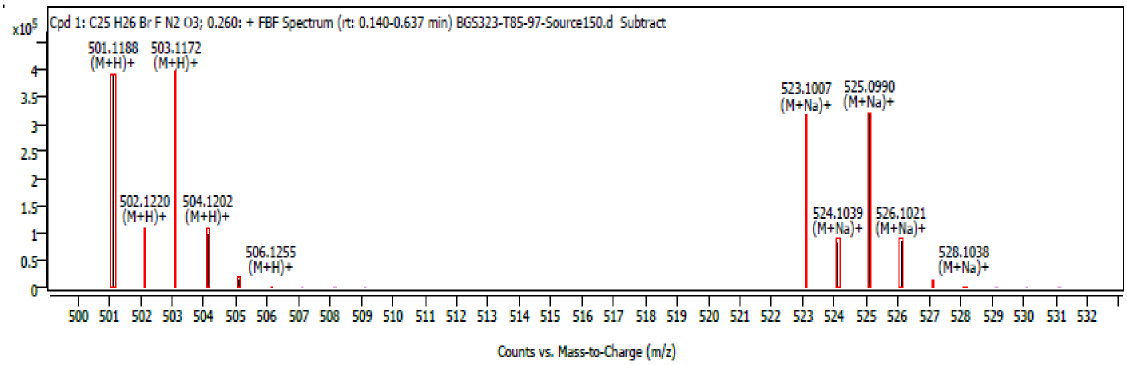

Figure S60. HRMS spectrum of 11b, diastereomer 1.

(3*R,5*R**)-1-Benzyl-3-bromo-5-(*N*-cyclohexylcarbamoyl)-5-(4-fluorobenzoyl)-2-pyrrolidinone (11b, diastereomer 2).**

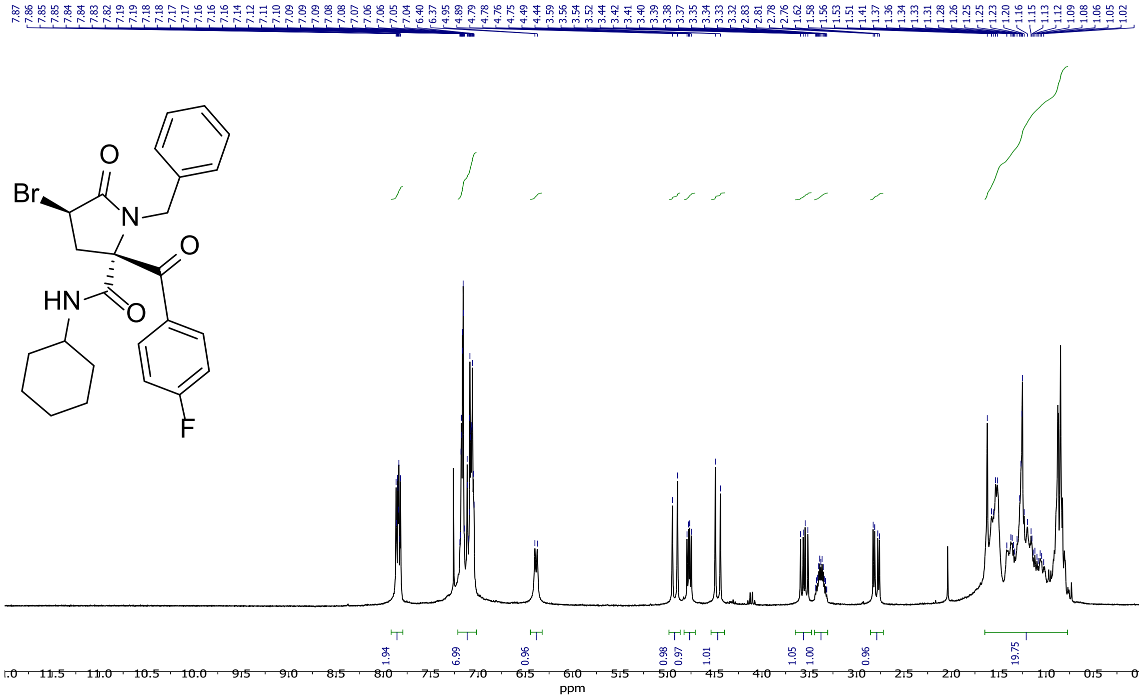

Figure S61. ^{1}H **NMR spectrum of 11b, diastereomer 2 (300 MHz, $CDCl_3$).**

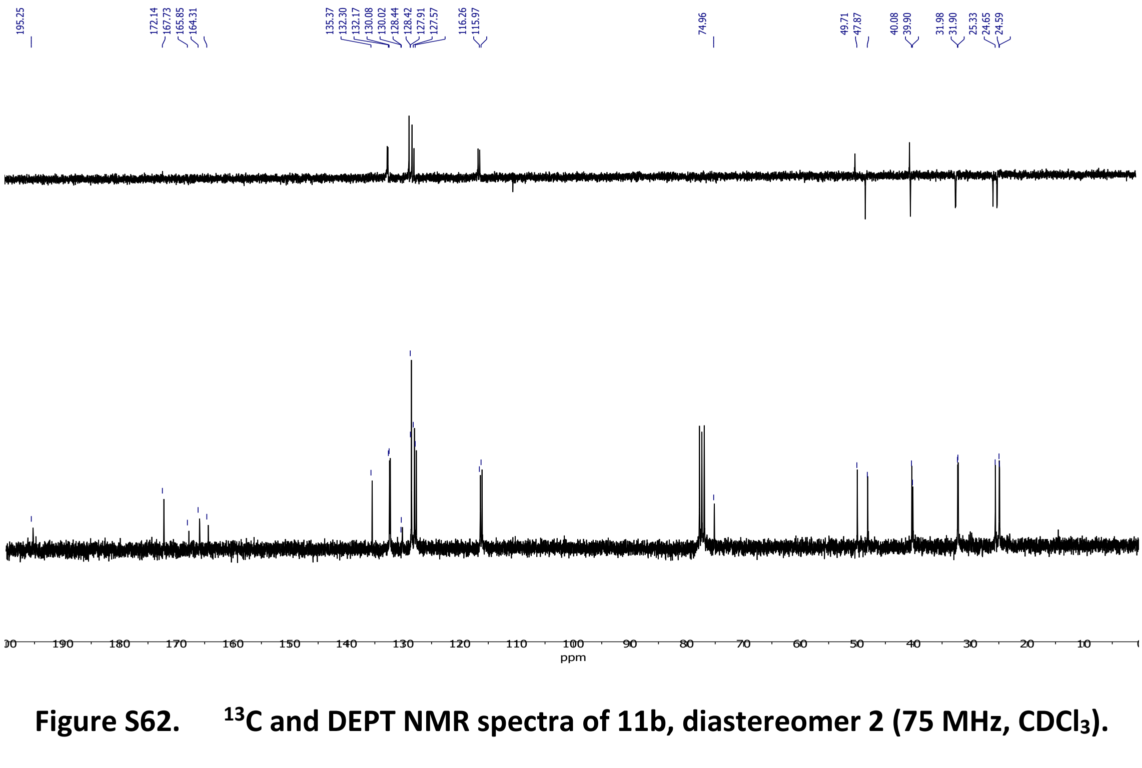

Figure S62. ^{13}C **and DEPT NMR spectra of 11b, diastereomer 2 (75 MHz, $CDCl_3$).**

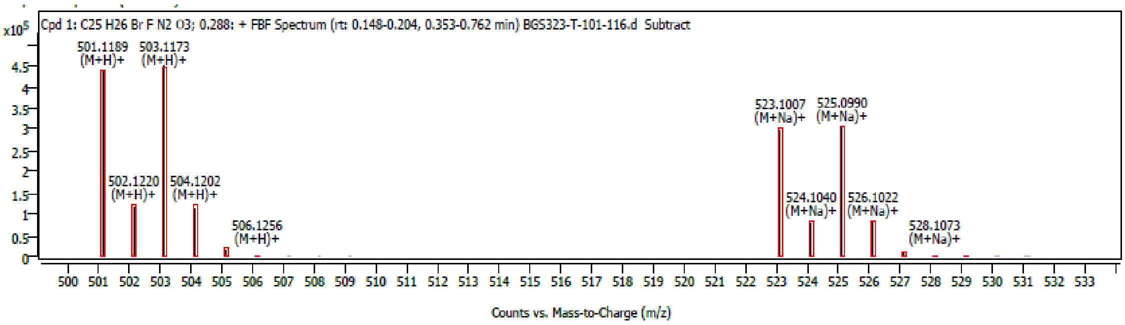

Figure S63. **HRMS spectrum of 11b, diastereomer 2.**

(3*R,5*S**)-5-Benzoyl-1-benzyl-3-bromo-5-(*N*-*tert*-butylcarbamoyl)-2-pyrrolidinone (11c, diastereomer 1).**

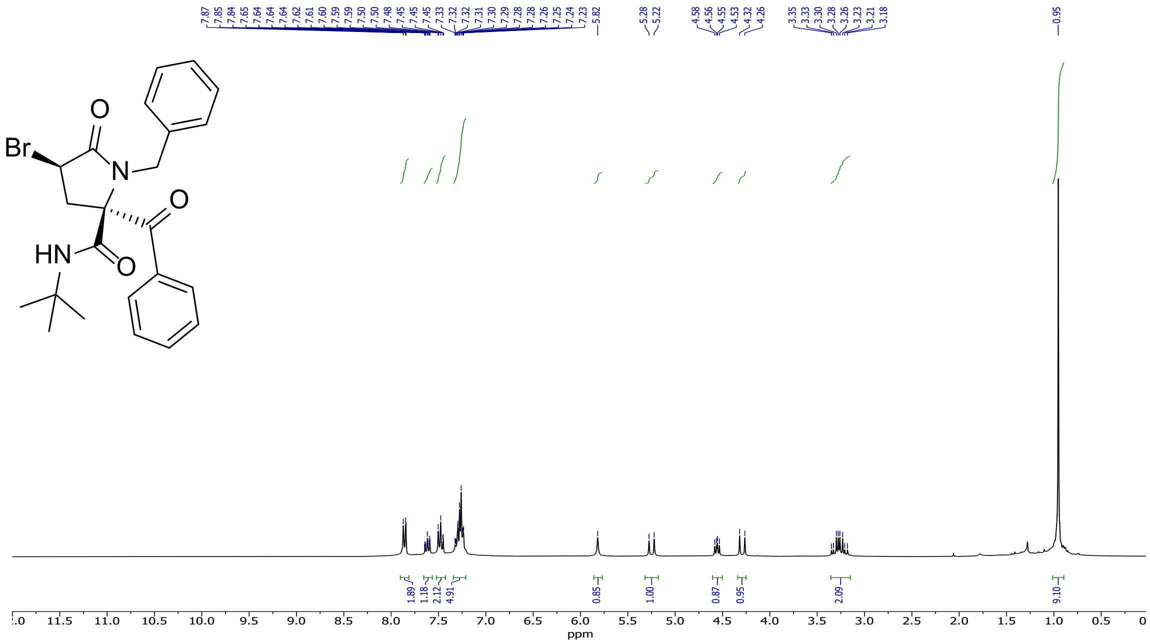

Figure S64. ^{1}H NMR spectrum of 11c, diastereomer 1 (300 MHz, $CDCl_3$).

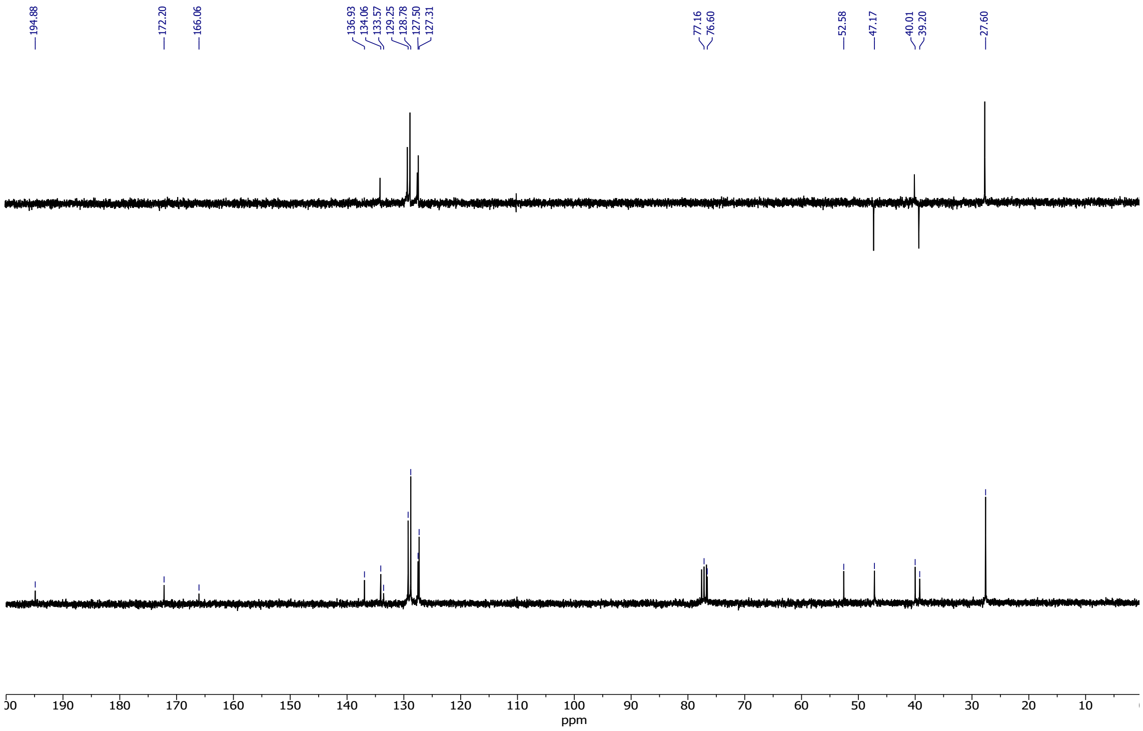

Figure S65. ^{13}C and DEPT NMR spectra of 11c, diastereomer 1 (75 MHz, $CDCl_3$).

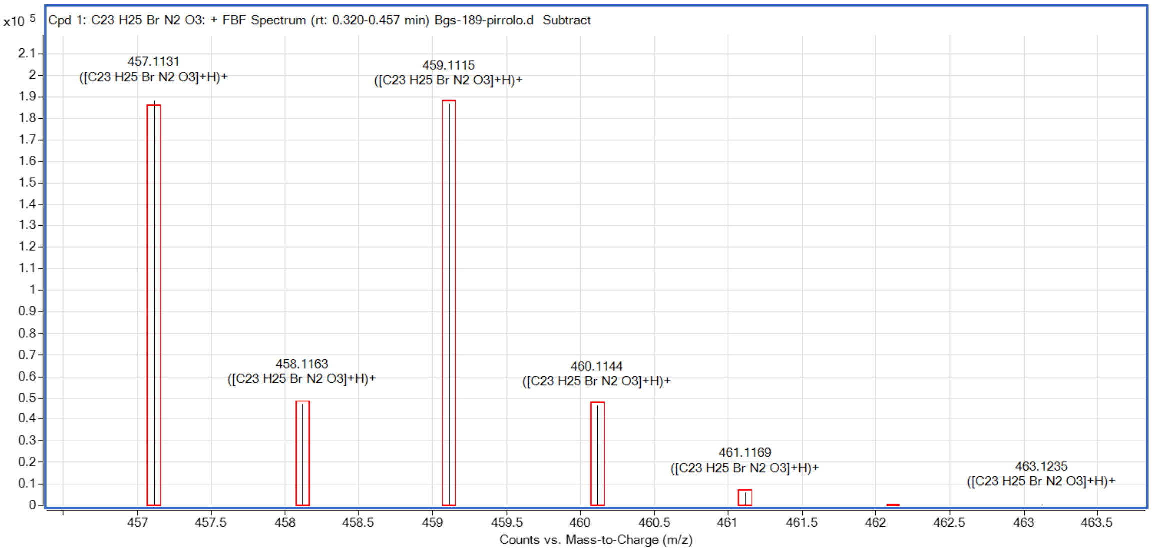

Figure S66. HRMS spectrum of 11c, diastereomer 1.

(3*R,5*R**)-5-Benzoyl-1-benzyl-3-bromo-5-(*N*-*tert*-butylcarbamoyl)-2-pyrrolidinone (11c, diastereomer 2).**

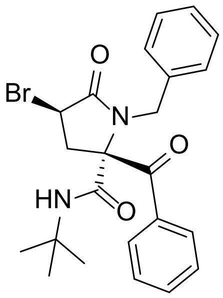

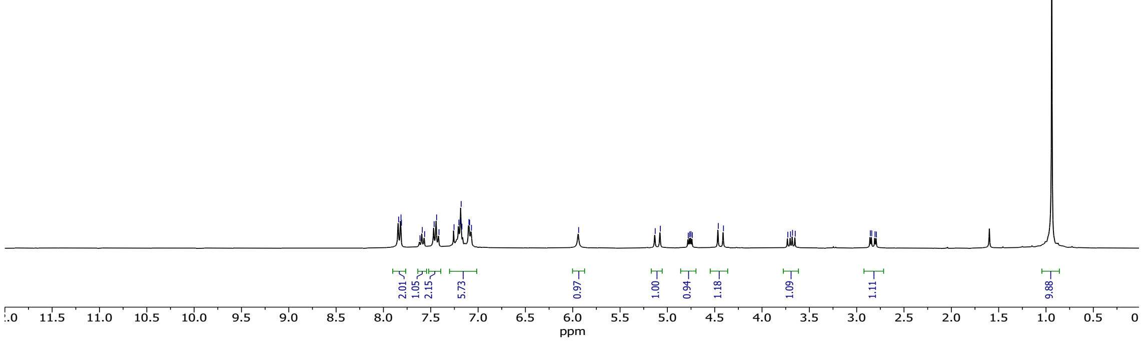

Figure S67. ^{1}H **NMR spectrum of 11c, diastereomer 2 (300 MHz,** $CDCl_3$**).**

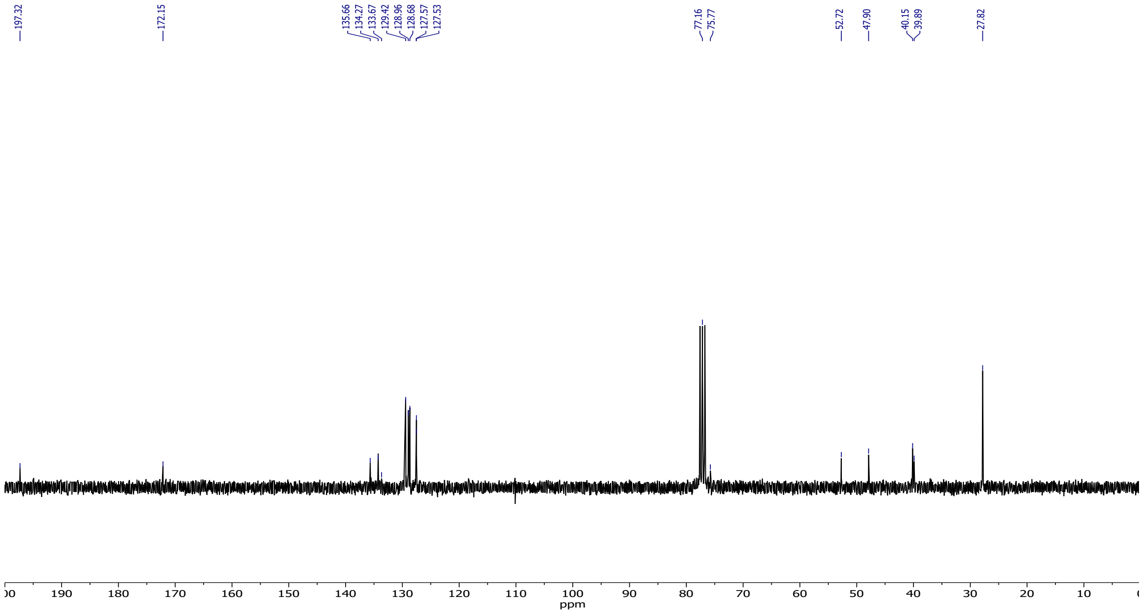

Figure S68. ^{13}C **spectrum of 11c, diastereomer 2 (75 MHz,** $CDCl_3$**).**

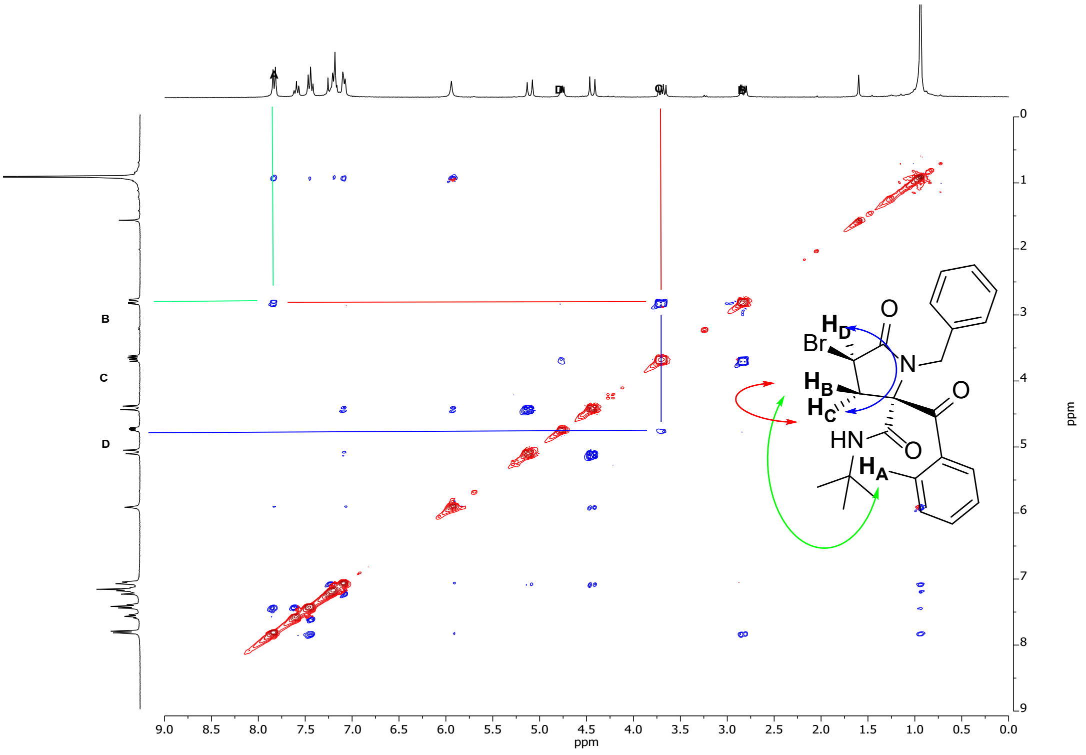

Figure S69. NOESY spectrum of 11c, diastereomer 2 ($CDCl_3$).

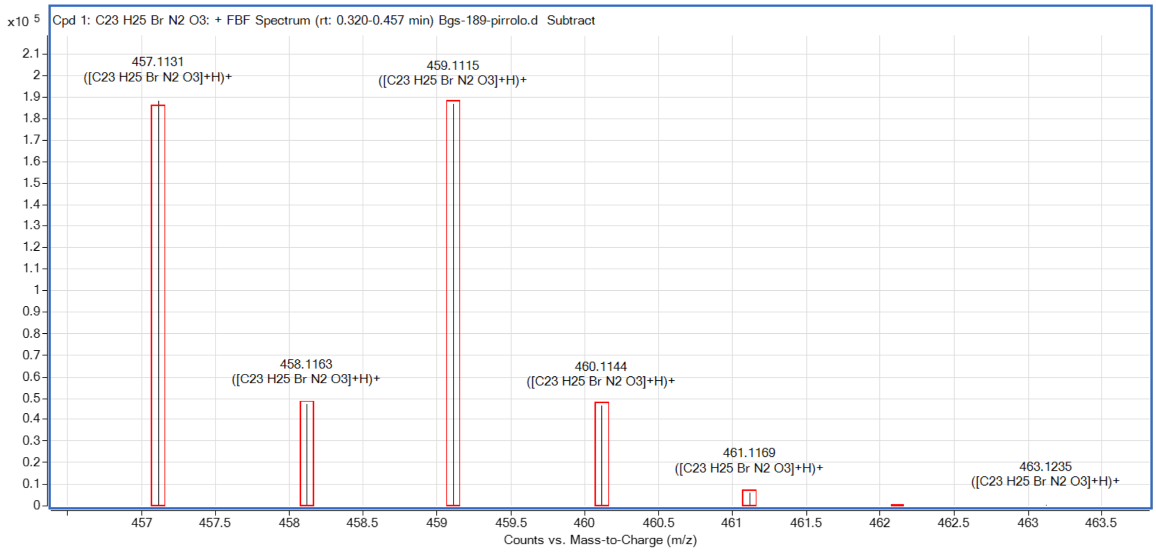

Figure S70. HRMS spectrum of 11c, diastereomer 2.

(3R*,5S*)-5-Benzoyl-1-(2-nitrobenzyl)-3-bromo-5-(*N*-cyclohexylcarbamoyl)-2-pyrrolidinone (11d, diastereomer 1)

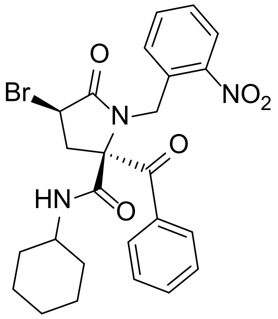

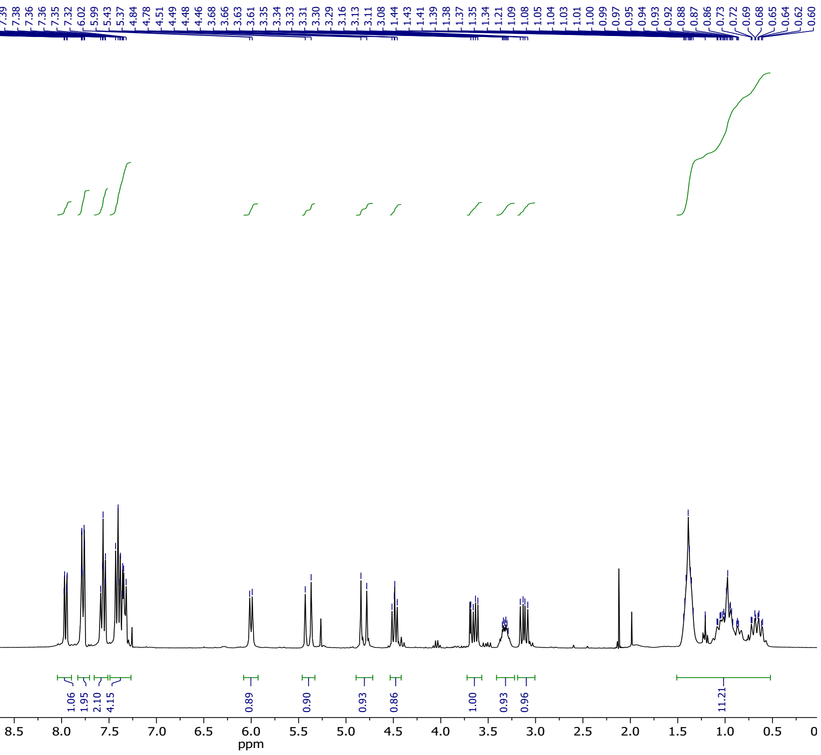

Figure S71. ^{1}H NMR spectrum of 11d, diastereomer 1 (300 MHz, $CDCl_3$).

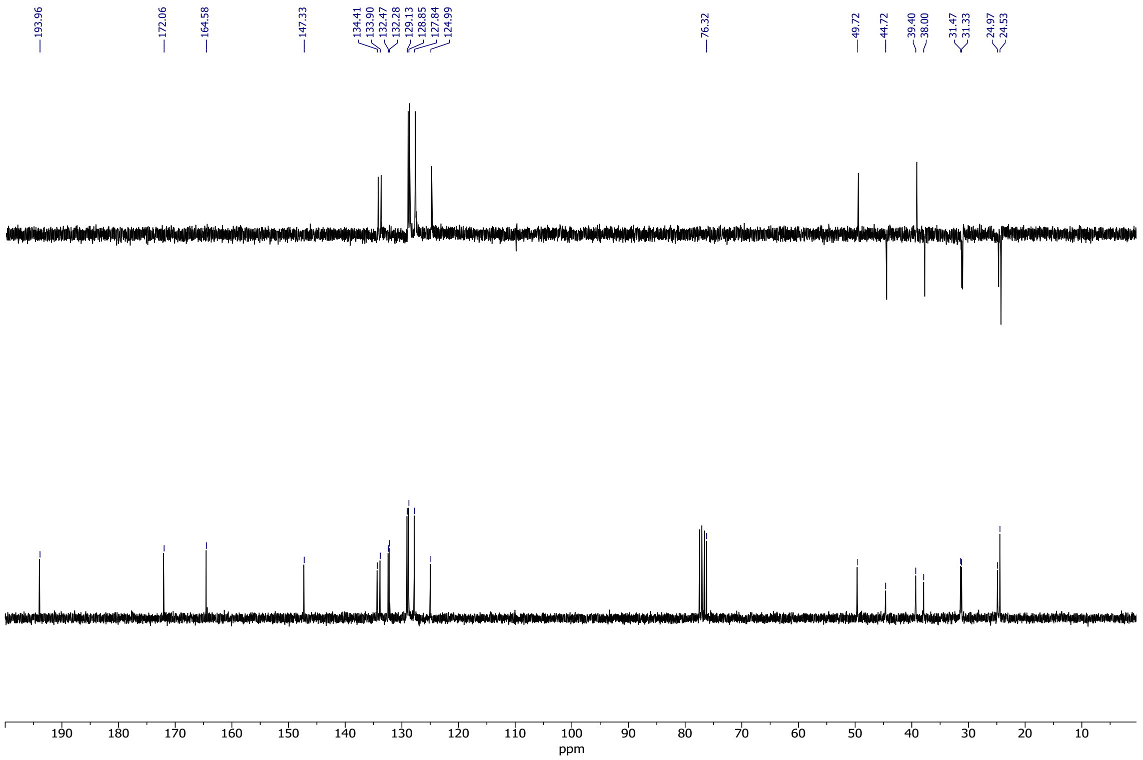

Figure S72. ^{13}C and DEPT NMR spectra of 11d, diastereomer 1 (75 MHz, $CDCl_3$).

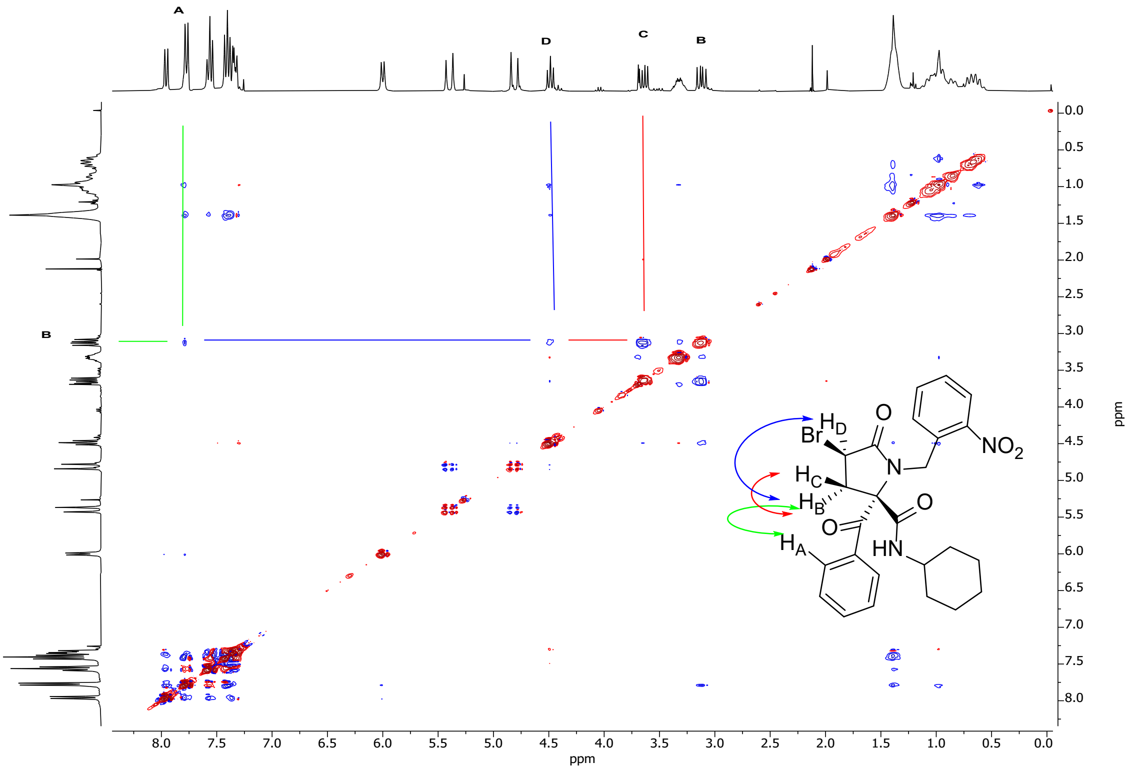

Figure S73. NOESY spectrum of 11d, diastereomer 1 ($CDCl_3$).

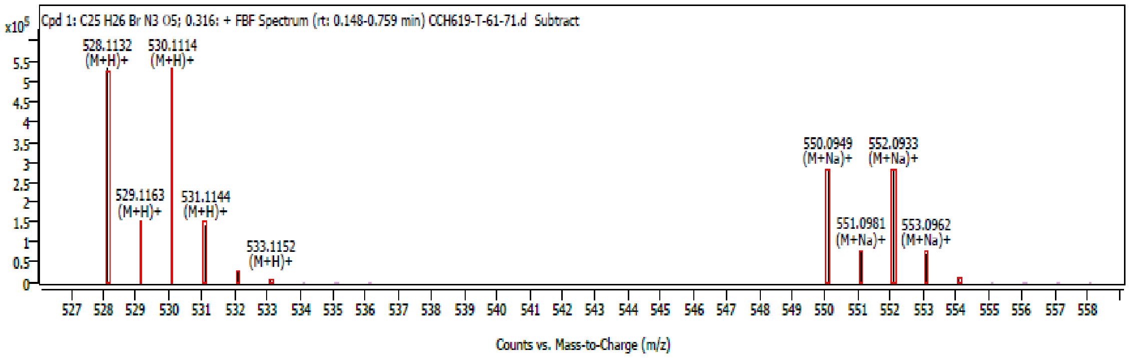

Figure S74. HRMS spectrum of 11d, diastereomer 1.

(3R*,5R*)-5-Benzoyl-1-(2-nitrobenzyl)-3-bromo-5-(*N*-cyclohexylcarbamoyl)-2-pyrrolidinone (11d, diastereomer 2)

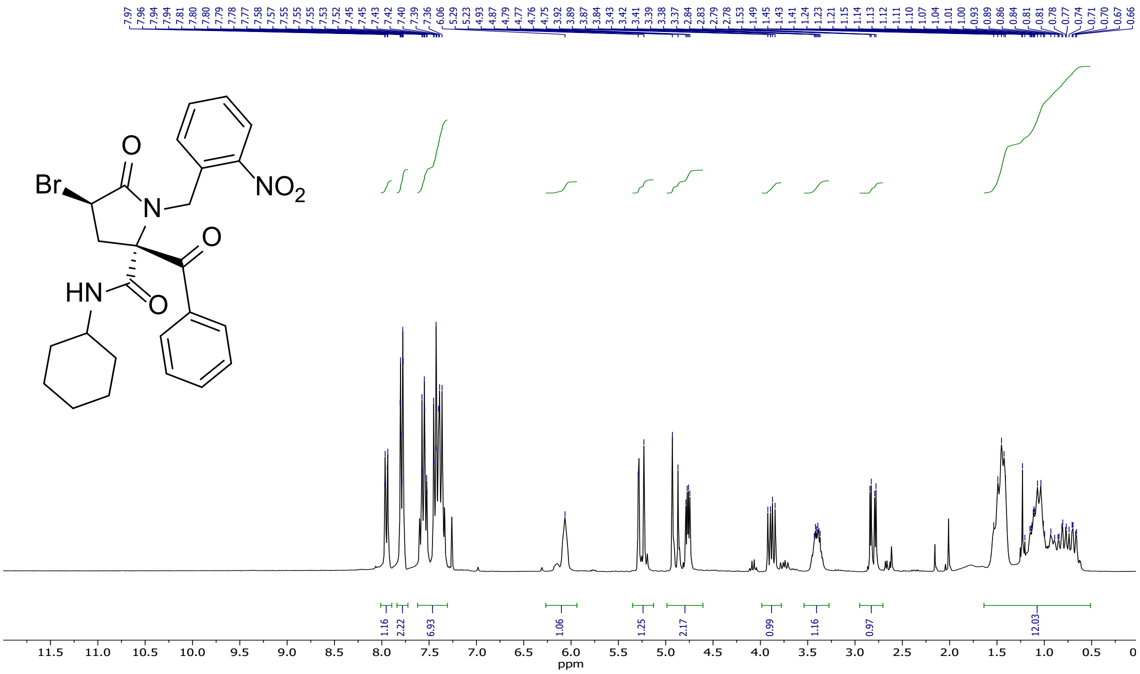

Figure S75. ^{1}H NMR spectrum of 11d, diastereomer 2 (300 MHz, $CDCl_3$).

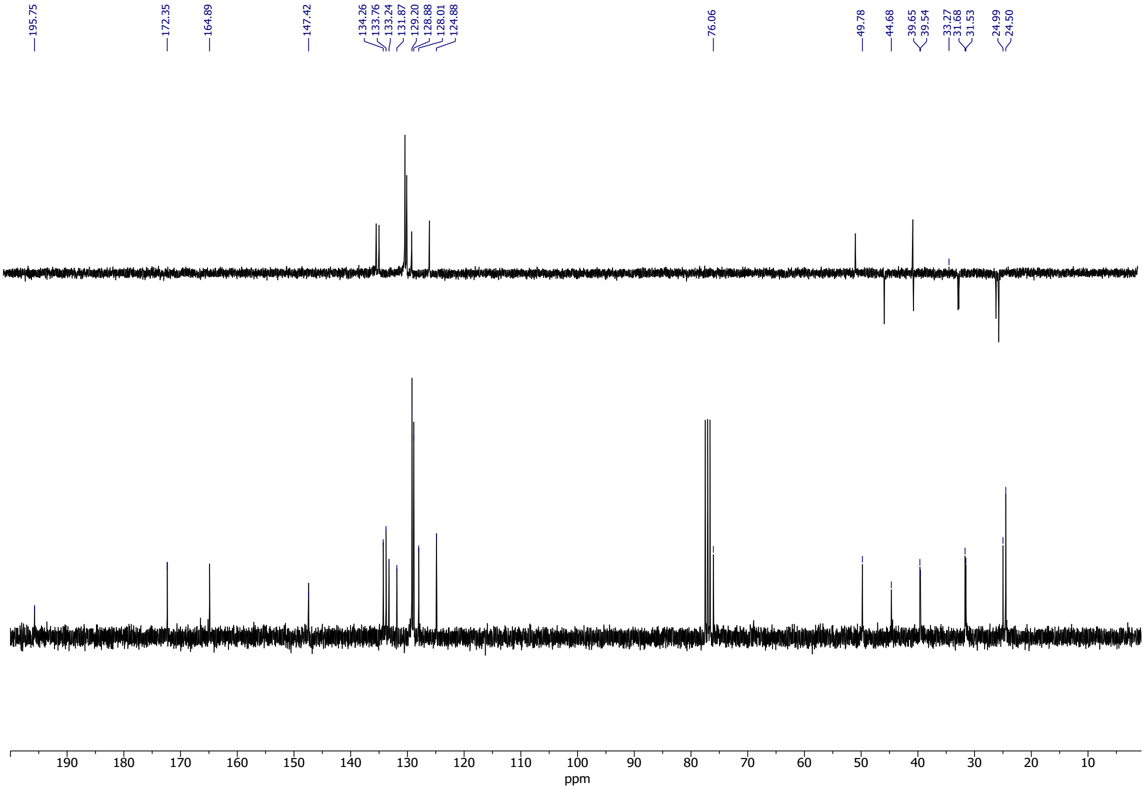

Figure S76. ^{13}C and DEPT NMR spectra of 11d, diastereomer 2 (75 MHz, $CDCl_3$).

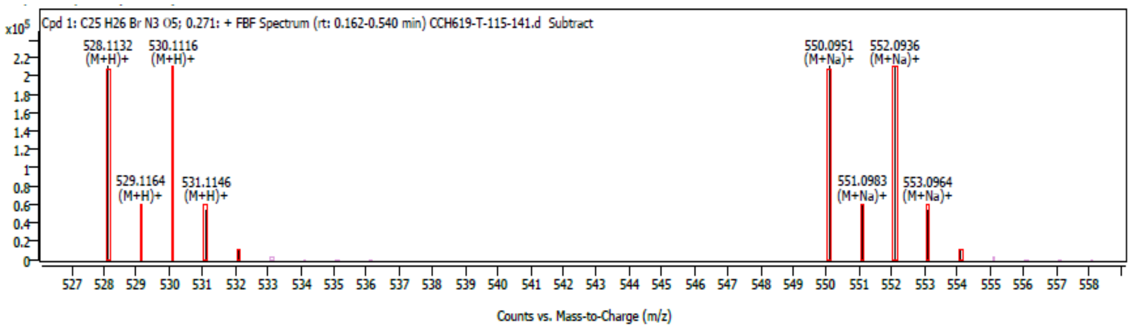

Figure S77. **HRMS spectrum of 11d, diastereomer 2.**

3-Bromo-5-(*N*-cyclohexylcarbamoyl)-5-(4-fluorobenzoyl)-1-nitrobenzyl--2-pyrrolidinone (11e).

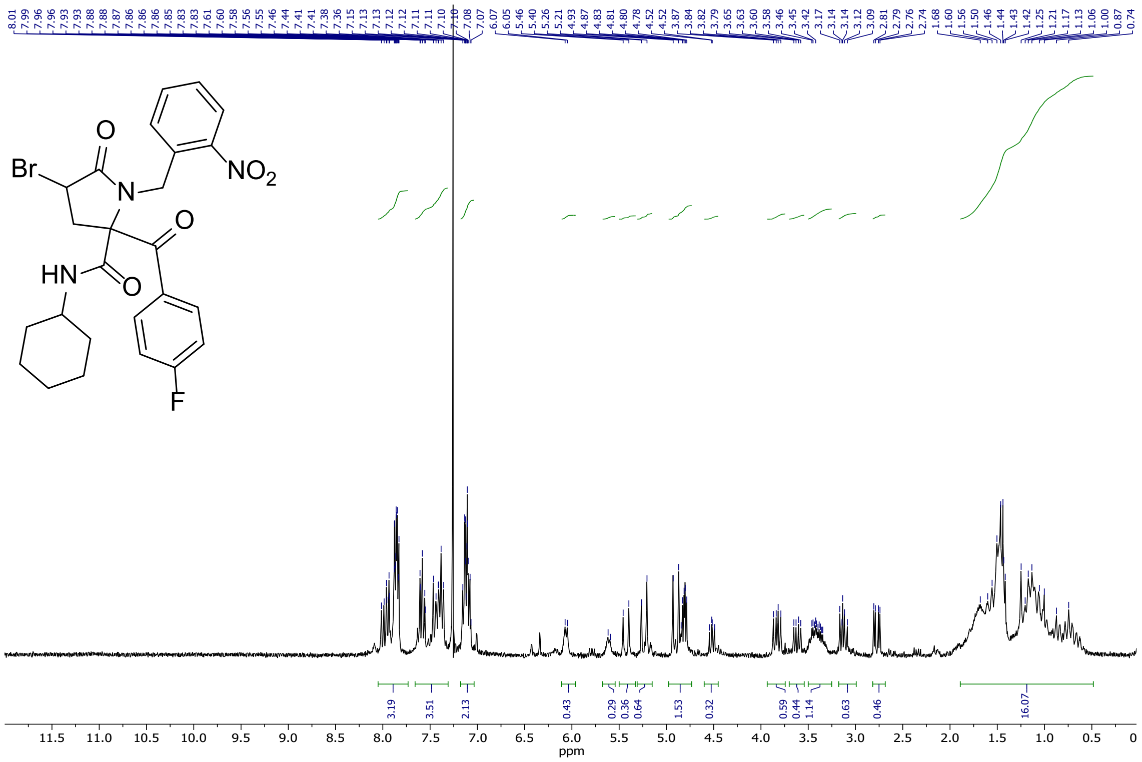

Figure S78. ^{1}H **NMR spectrum of 11e, mixture of diastereomers (300 MHz, $CDCl_3$).**

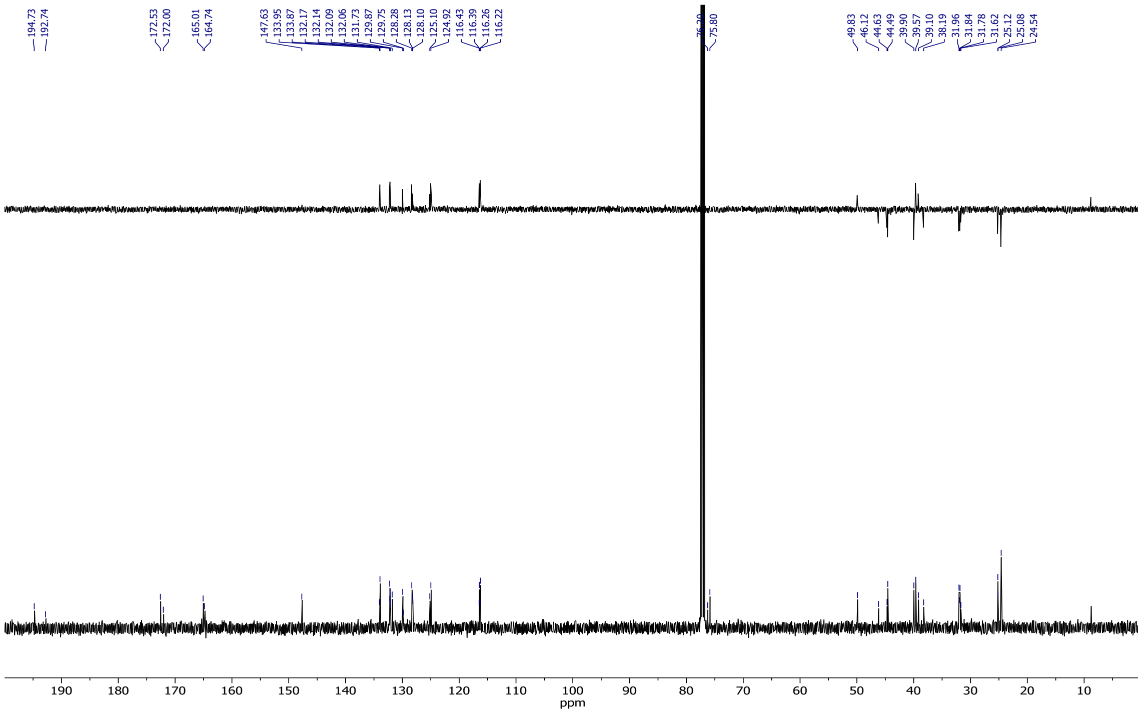

Figure S79. ^{13}C **and DEPT NMR spectra of 11e, mixture of diastereomers (75 MHz, $CDCl_3$).**

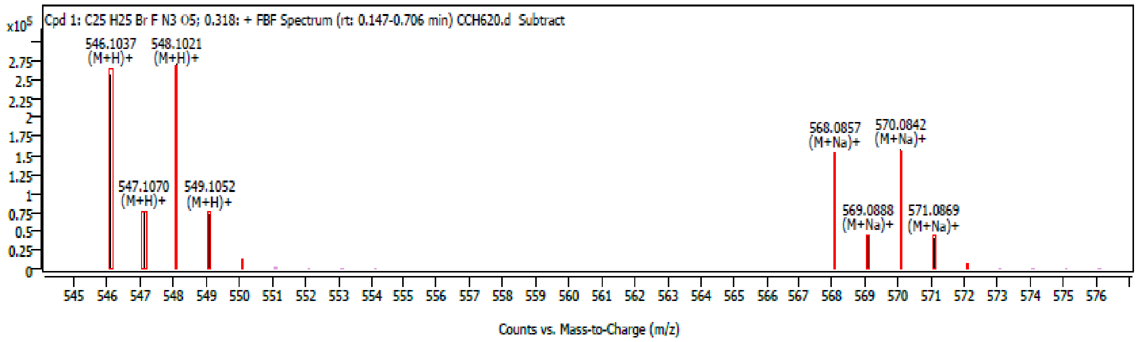

Figure S80. **HRMS spectrum of 11e.**

(1*R,3*R**,4*R**)-5-Benzyl-4-(*N*-cyclohexylcarbamoyl)-3-methoxy-3-phenyl-2-oxa-5-azabicyclo[2.2.1]heptan-6-one (12a).**

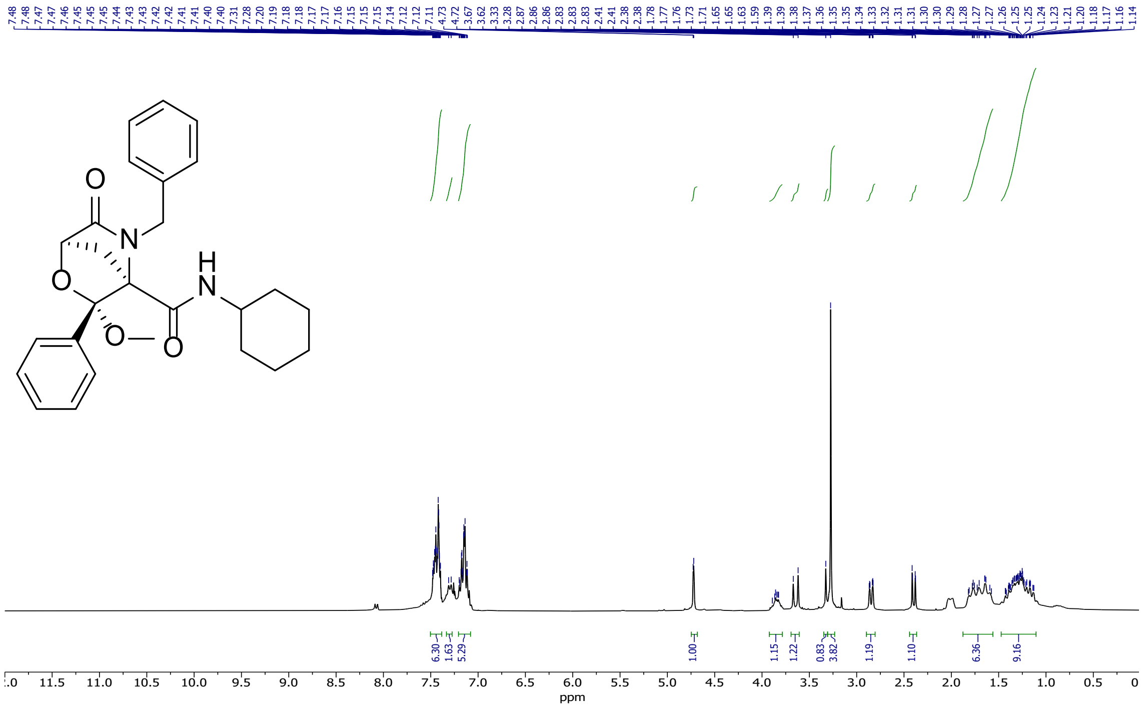

Figure S81. **^{1}H NMR spectrum of 12a (300 MHz, $CDCl_3$).**

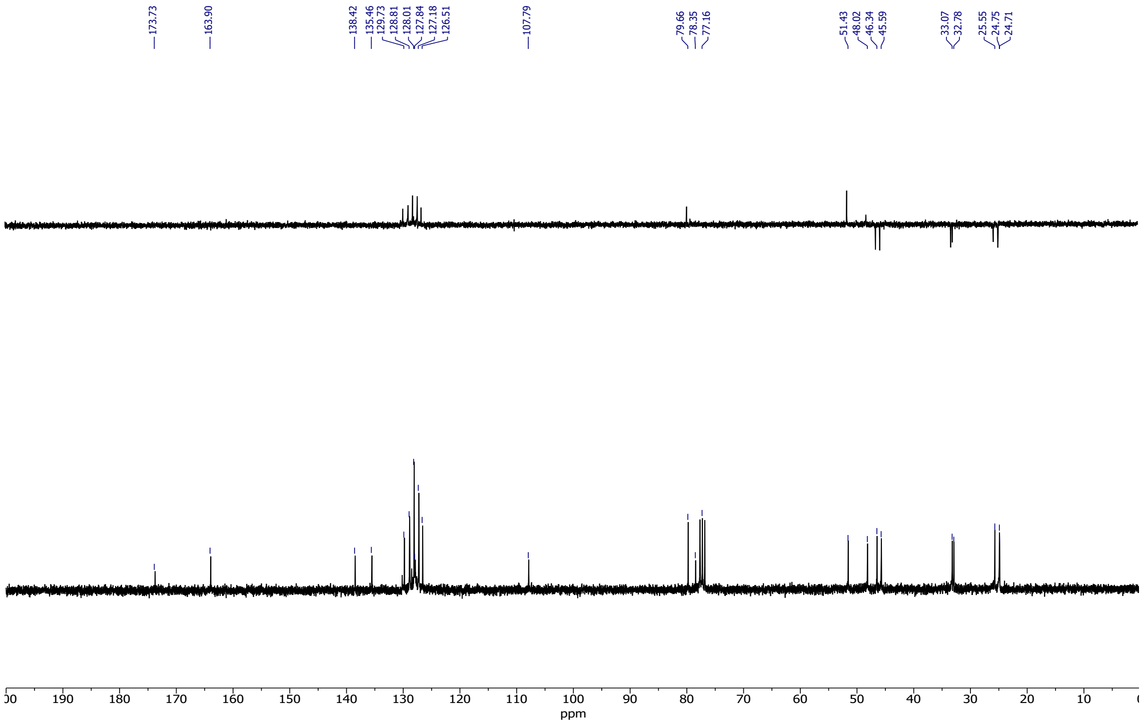

Figure S82. **^{13}C and DEPT NMR spectra of 12a (75 MHz, $CDCl_3$).**

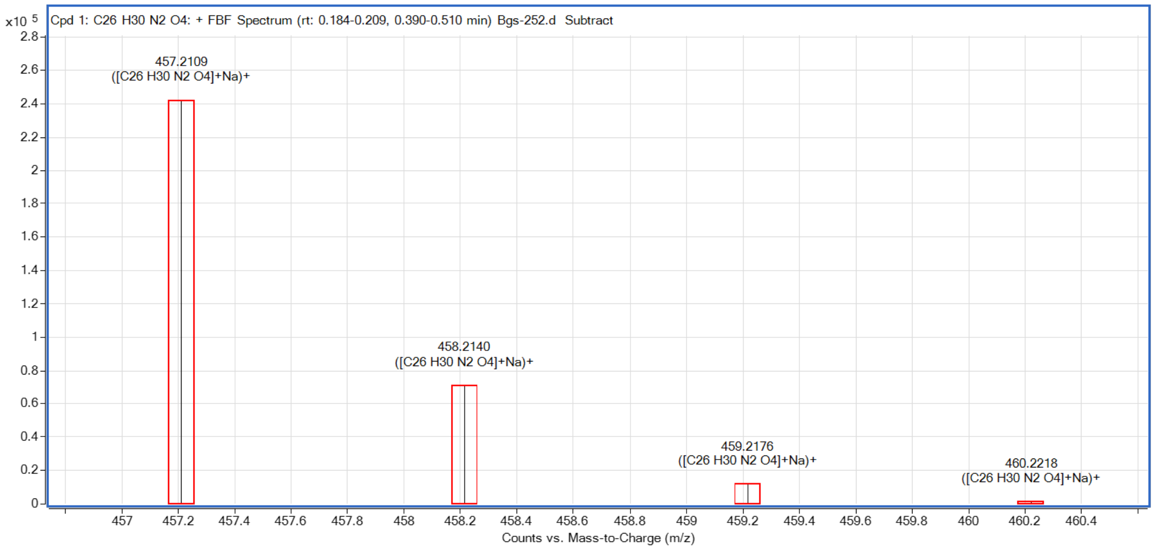

Figure S83. HRMS spectrum of 12a.

(1*R,3*R**,4*R**)-5-Benzyl-4-(*N*-cyclohexylcarbamoyl)-3-ethoxy-3-phenyl-2-oxa-5-azabicyclo[2.2.1]heptan-6-one (12b).**

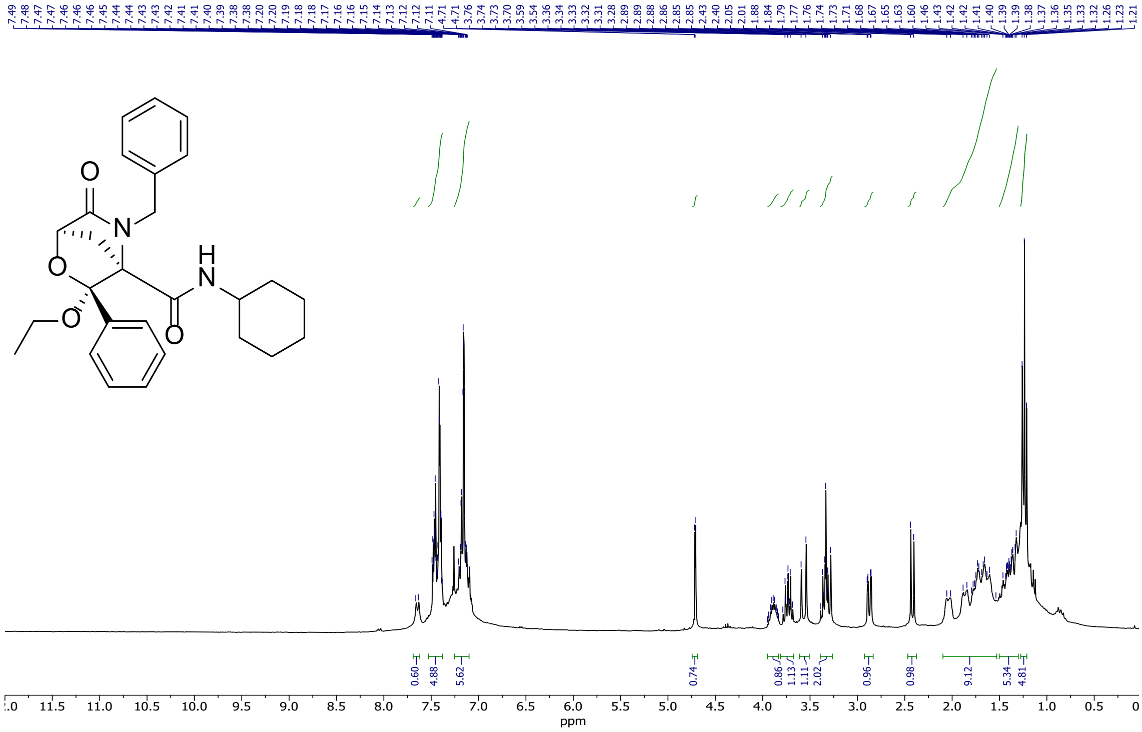

Figure S84. **^{1}H NMR spectrum of 12b (300 MHz, $CDCl_3$).**

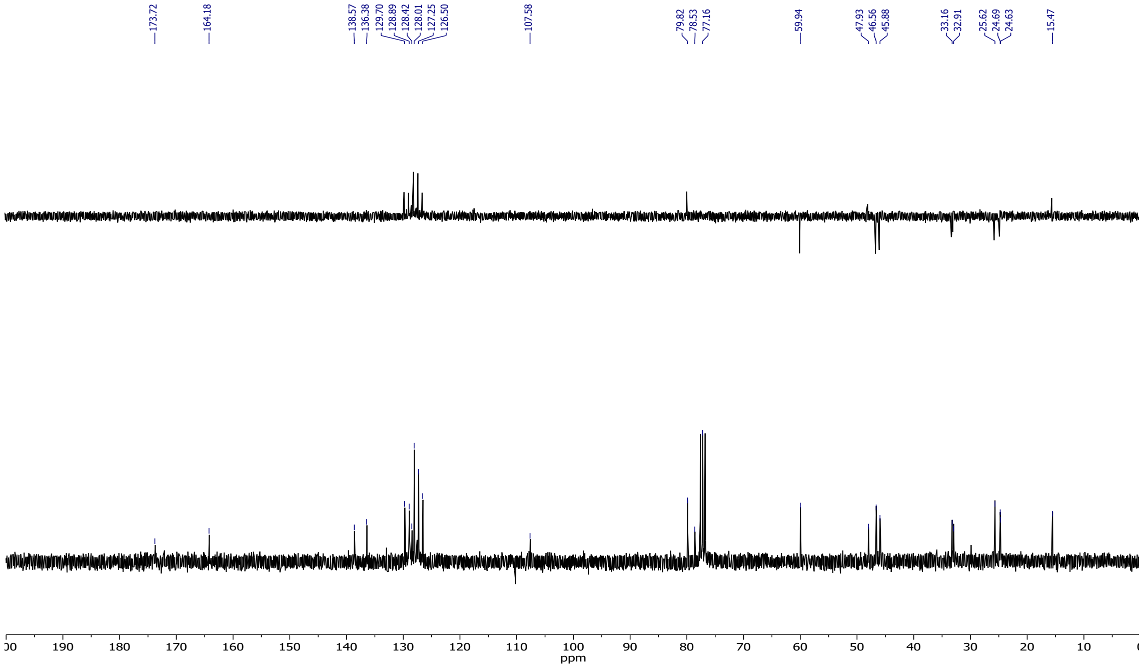

Figure S85. **^{13}C and DEPT NMR spectra of 12b (75 MHz, $CDCl_3$).**

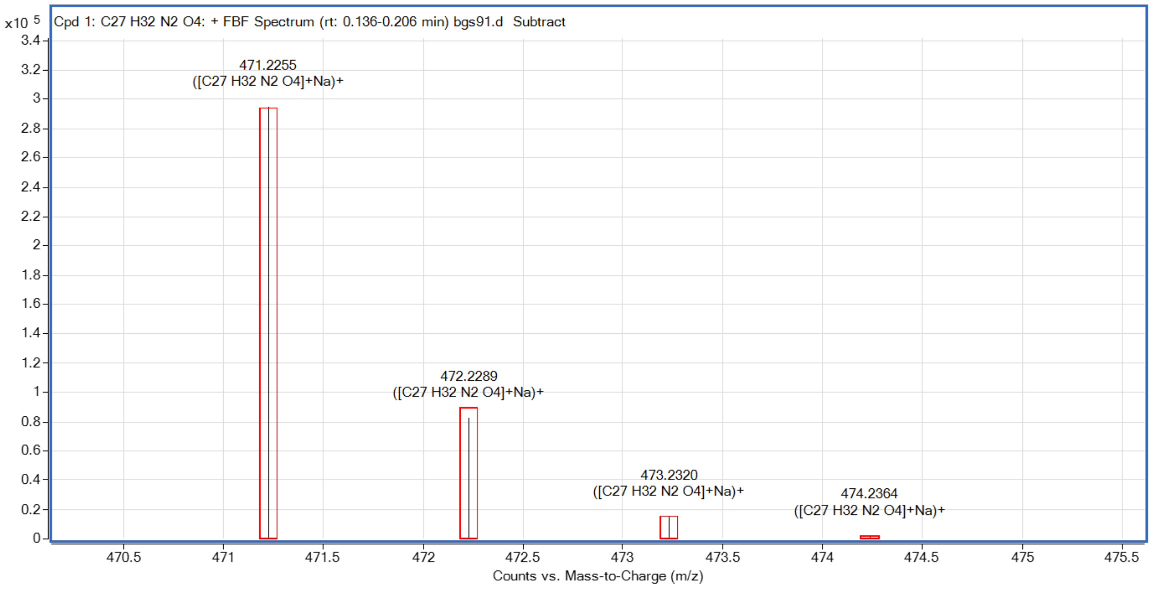

Figure S86. **HRMS spectrum of 12b.**

(1*R,3*R**,4*R**)-5-Benzyl-3-(2-chloroethoxy)-4-(*N*-cyclohexylcarbamoyl)-3-phenyl-2-oxa-5-azabicyclo[2.2.1]heptan-6-one (12c).**

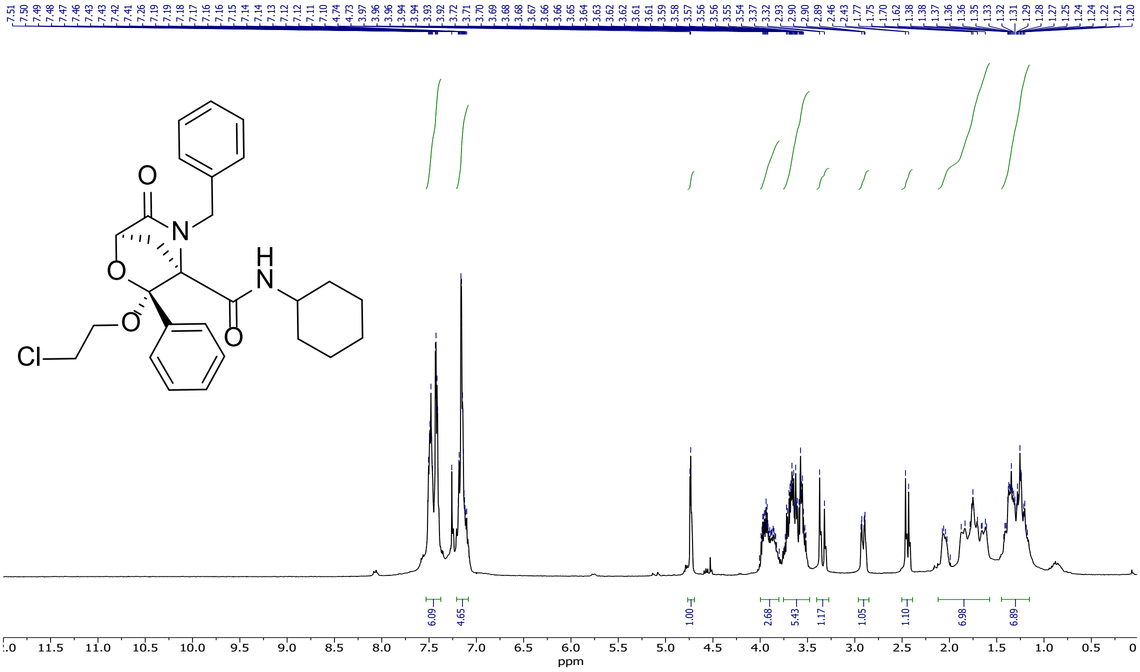

Figure S87. ^{1}H NMR spectrum of 12c (300 MHz, $CDCl_3$).

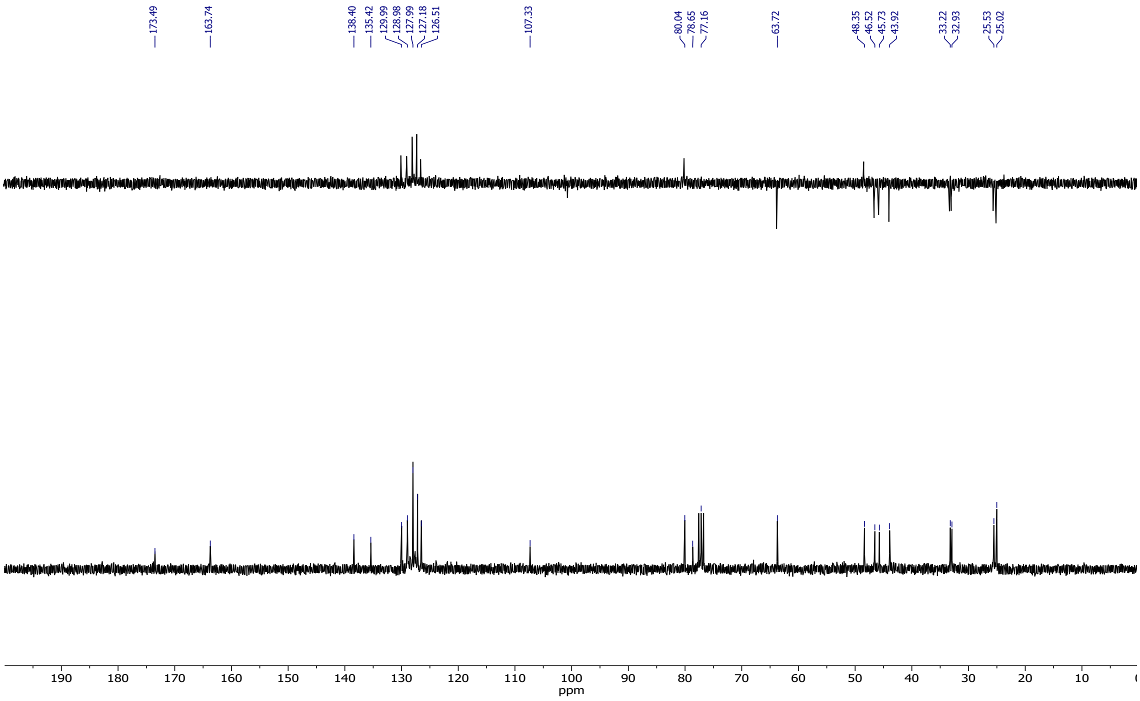

Figure S88. ^{13}C and DEPT NMR spectra of 12c (75 MHz, $CDCl_3$).

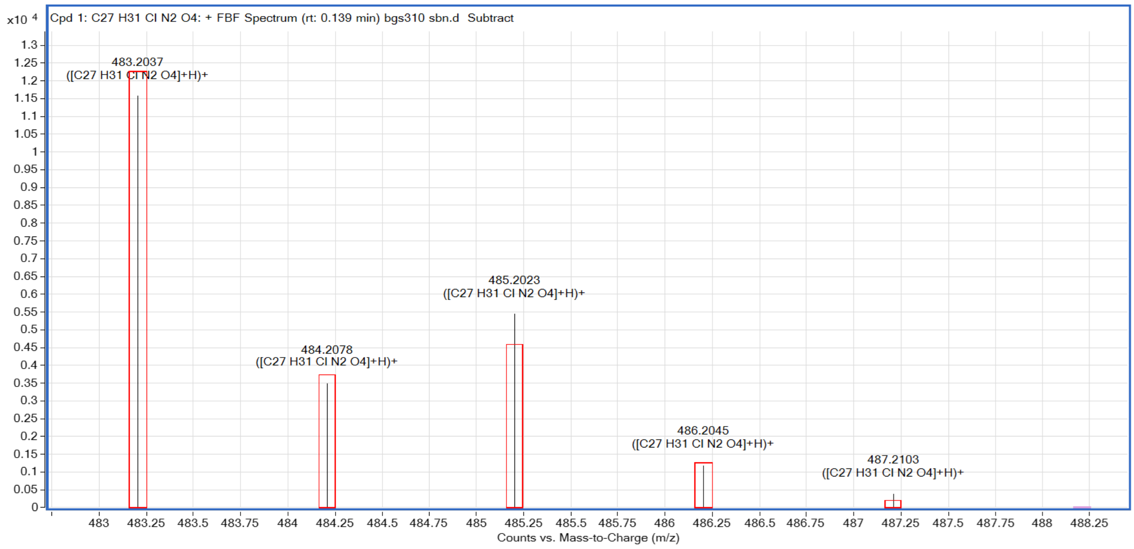

Figure S89. **HRMS spectrum of 12c.**

(1*R,3*R**,4*R**)-5-Benzyl-4-(*N*-cyclohexylcarbamoyl)-3-phenyl-3-(2,2,2-trifluoroethoxy)-2-oxa-5-azabicyclo[2.2.1]heptan-6-one (12d).**

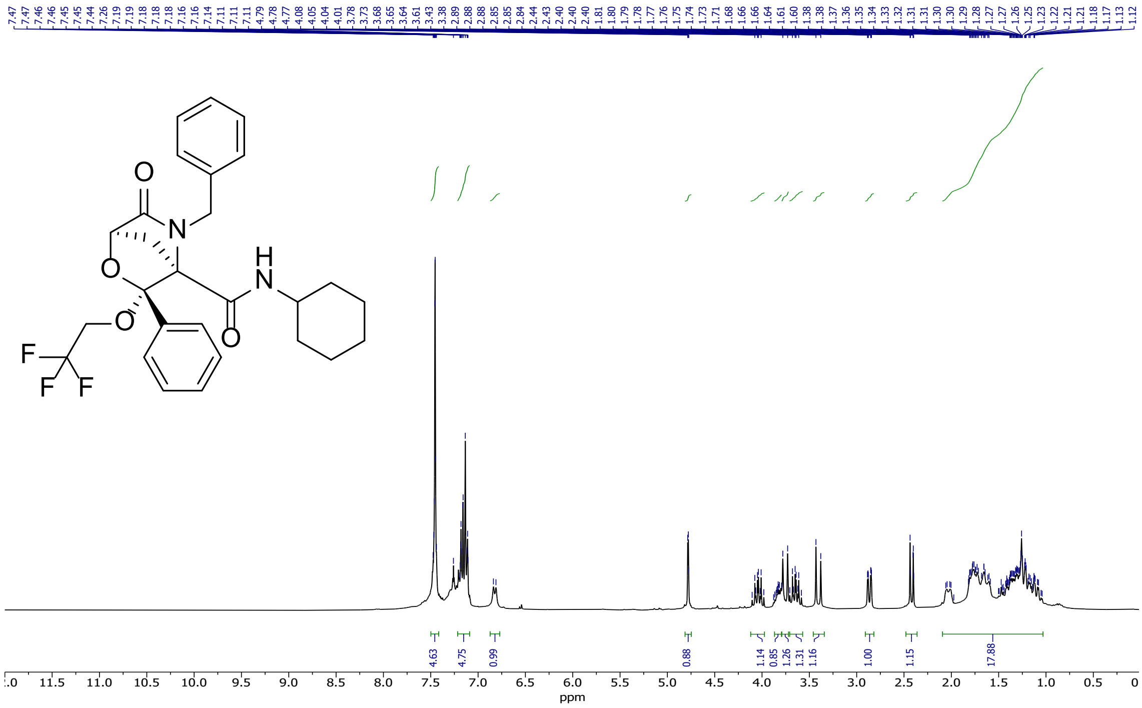

Figure S90. ^{1}H NMR spectrum of 12d (300 MHz, $CDCl_3$).

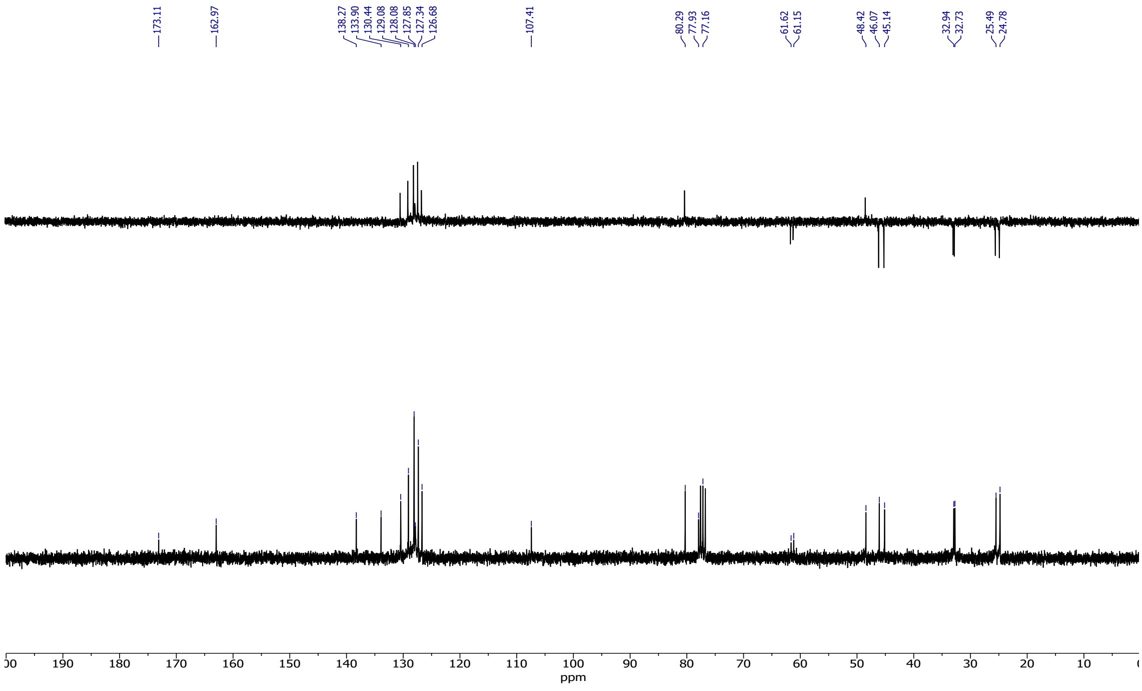

Figure S91. ^{13}C and DEPT NMR spectra of 12d (75 MHz, $CDCl_3$).

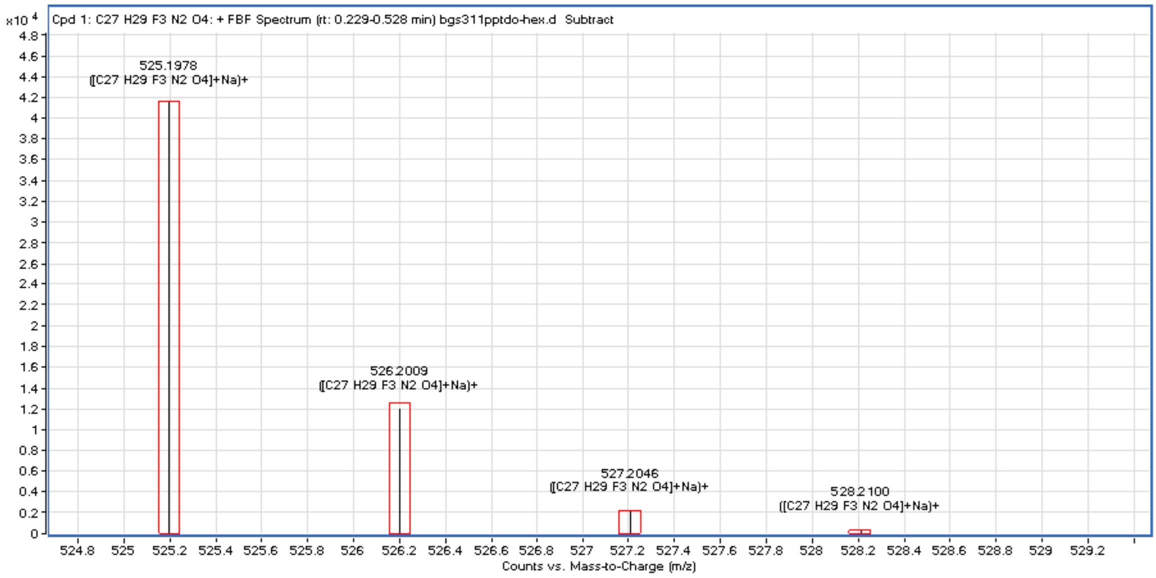

Figure S92. HRMS spectrum of 12d.

(1*R,3*R**,4*R**)-5-Benzyl-4-(*N*-cyclohexylcarbamoyl)-3-(4-fluorophenyl)-3-methoxy-2-oxa-5-azabicyclo[2.2.1]heptan-6-one (12e).**

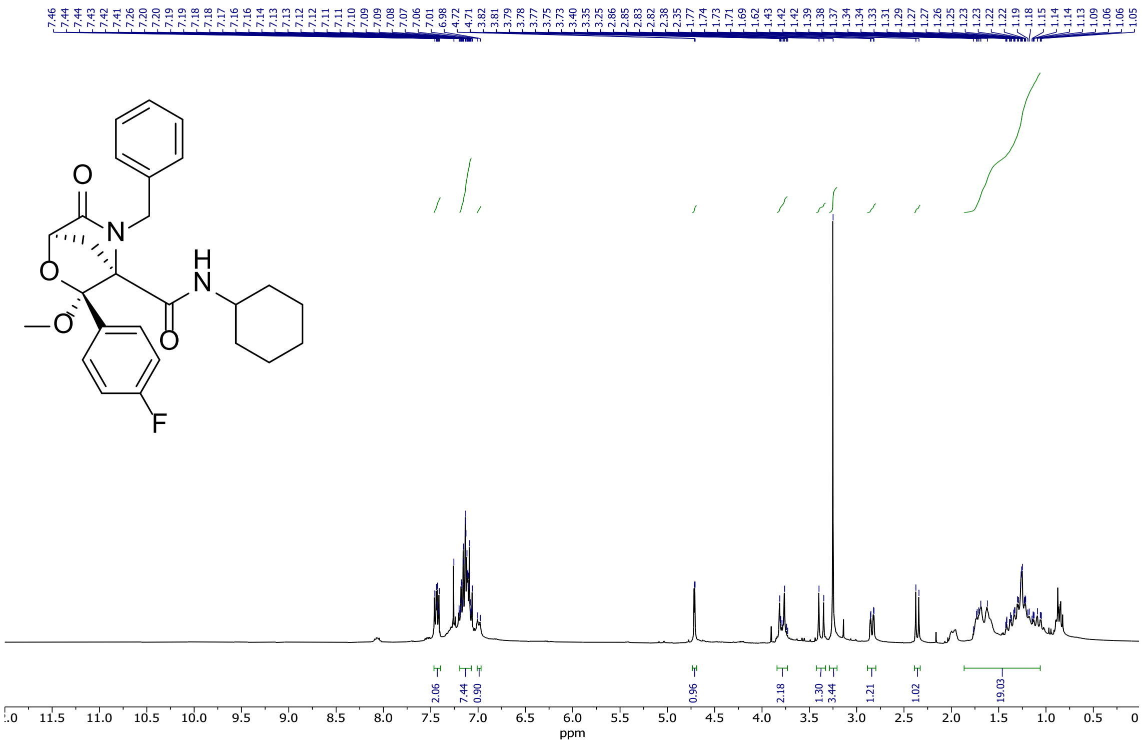

Figure S93. ^{1}**H NMR spectrum of 12e (300 MHz, CDCl$_3$).**

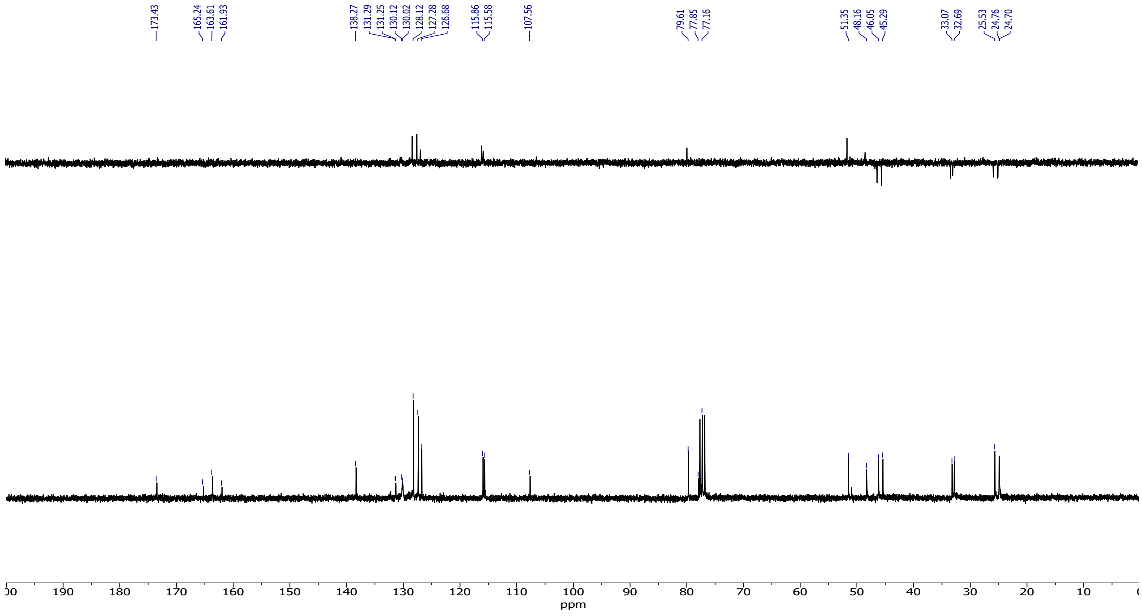

Figure S94. ^{13}**C and DEPT NMR spectra of 12e (75 MHz, CDCl$_3$).**

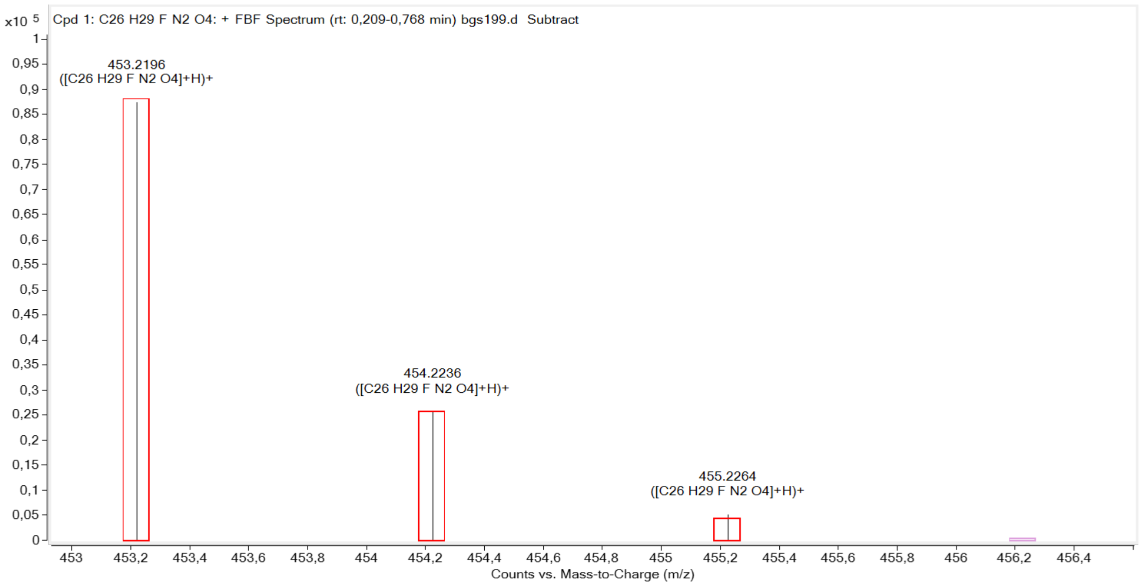

Figure S95. **HRMS spectrum of 12e.**

(1*R,3*R**,4*R**)-5-Benzyl-4-(*N*-*tert*-butylcarbamoyl)-3-methoxy-3-phenyl-2-oxa-5-azabicyclo[2.2.1]heptan-6-one (12f).**

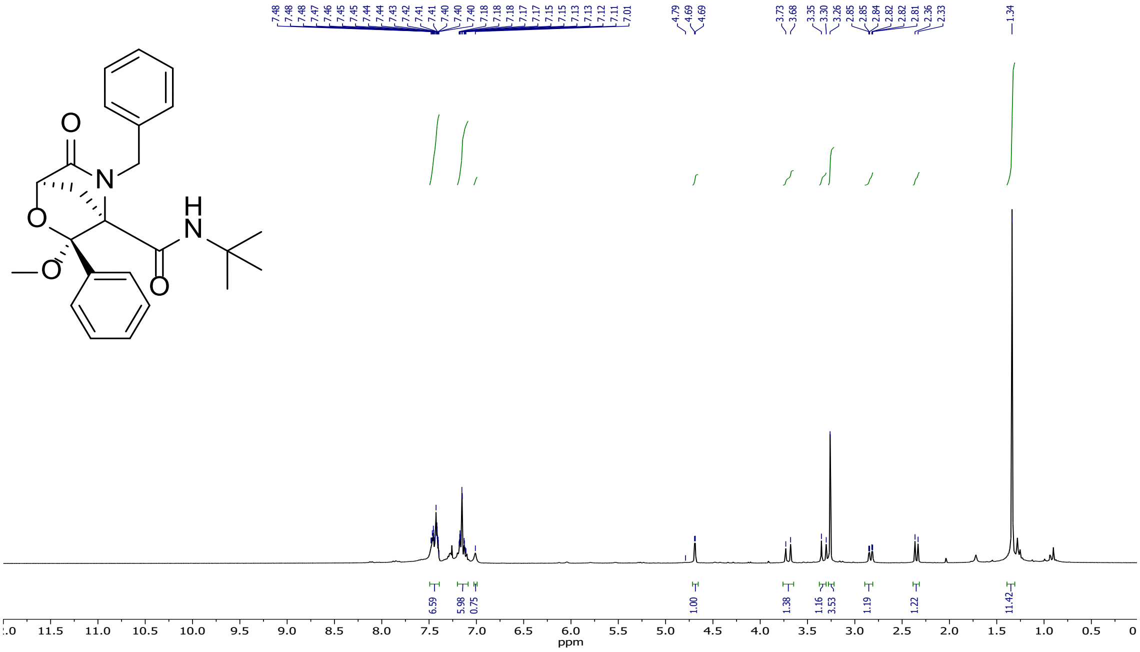

Figure S96. ^{1}H NMR spectrum of 12f (300 MHz, $CDCl_3$).

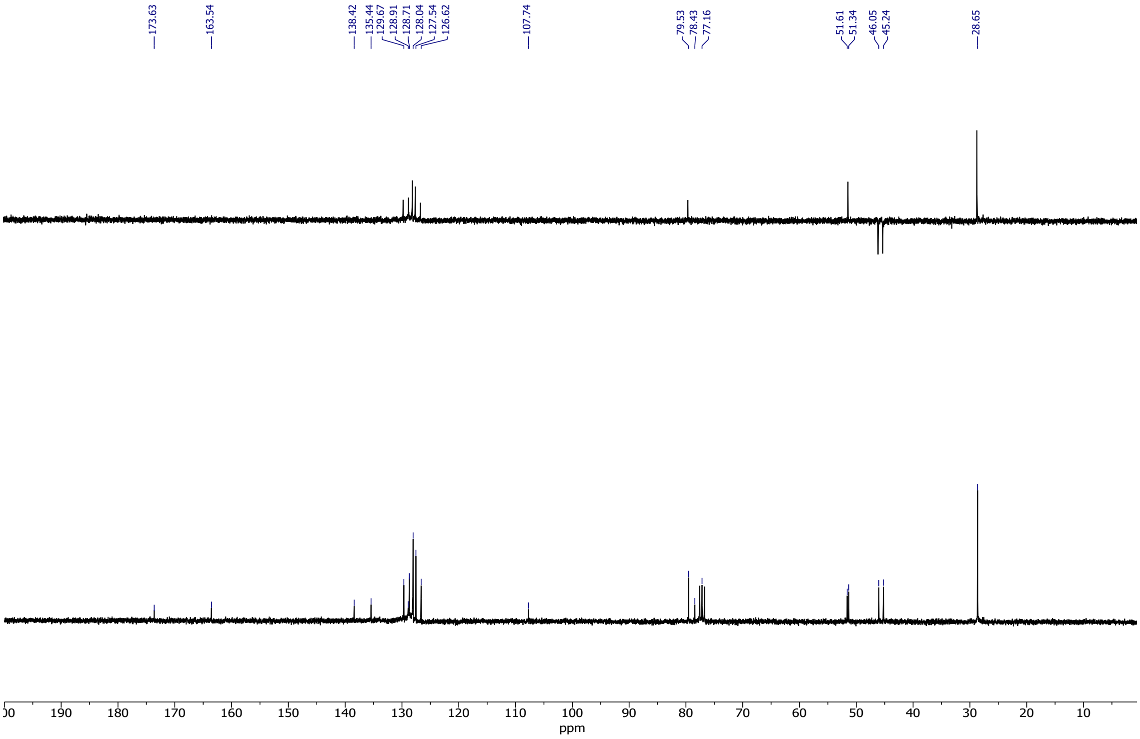

Figure S97. ^{13}C and DEPT NMR spectra of 12f (75 MHz, $CDCl_3$).

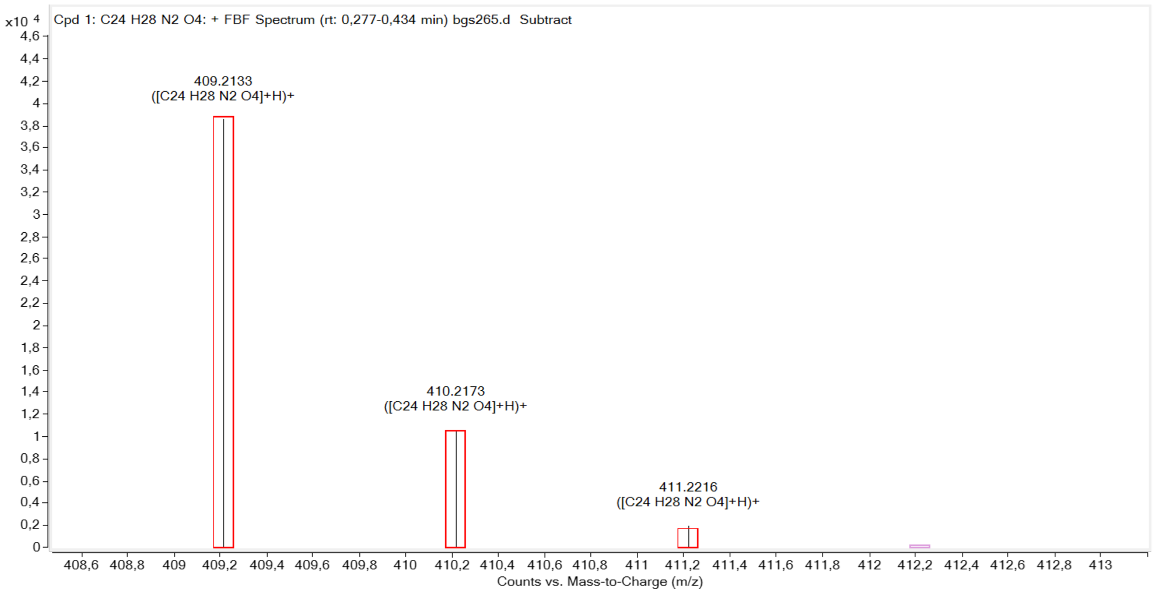

Figure S98. **HRMS spectrum of 12f.**

(1*R,3*R**,4*R**)-4-(*N*-Cyclohexylcarbamoyl)-3-methoxy-5-(2-nitrobenzyl)-3-phenyl-2-oxa-5-azabicyclo[2.2.1]heptan-6-one (12g).**

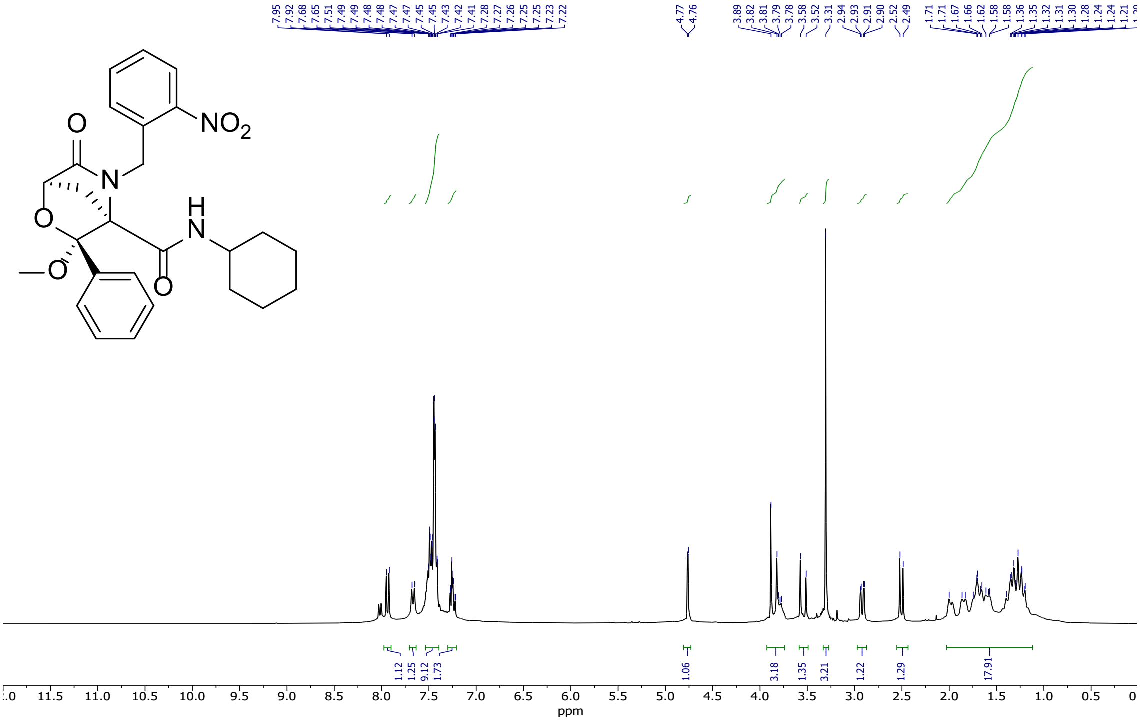

Figure S99. ^{1}H NMR spectrum of 12g (300 MHz, $CDCl_3$).

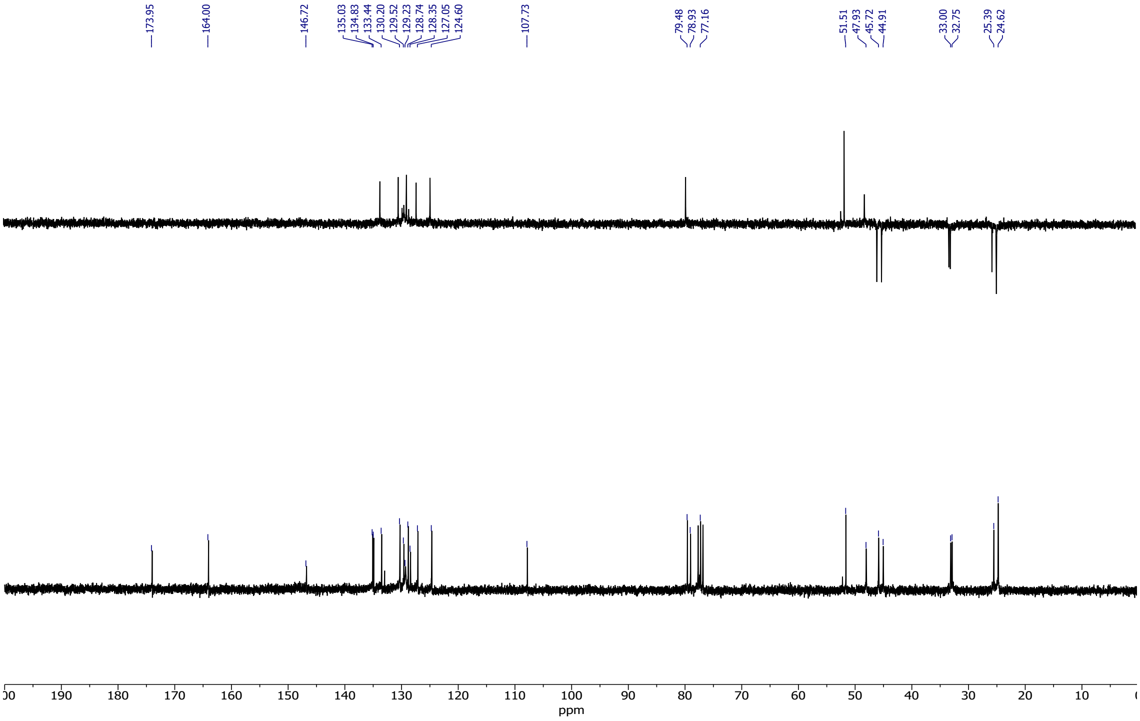

Figure S100. ^{13}C and DEPT NMR spectra of 12g (75 MHz, $CDCl_3$).

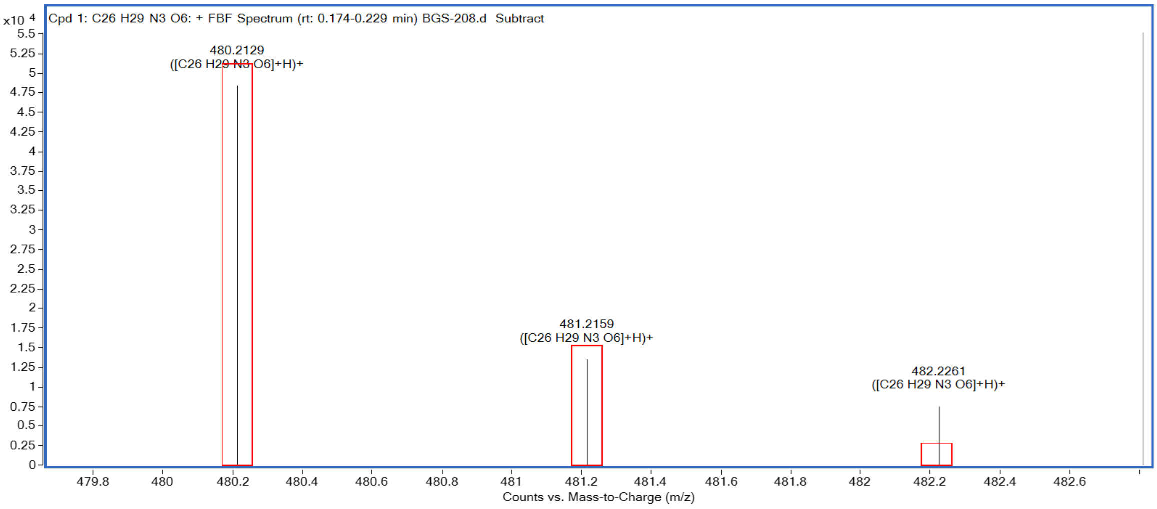

Figure S101. HRMS spectrum of 12g.

(1*R,3*R**,4*R**)-4-(*N*-Cyclohexylcarbamoyl)-3-(4-fluorophenyl)-3-methoxy-5-(2-nitrobenzyl)-2-oxa-5-azabicyclo[2.2.1]heptan-6-one (12h).**

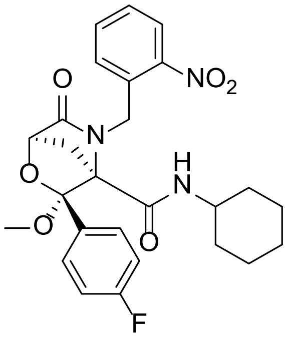

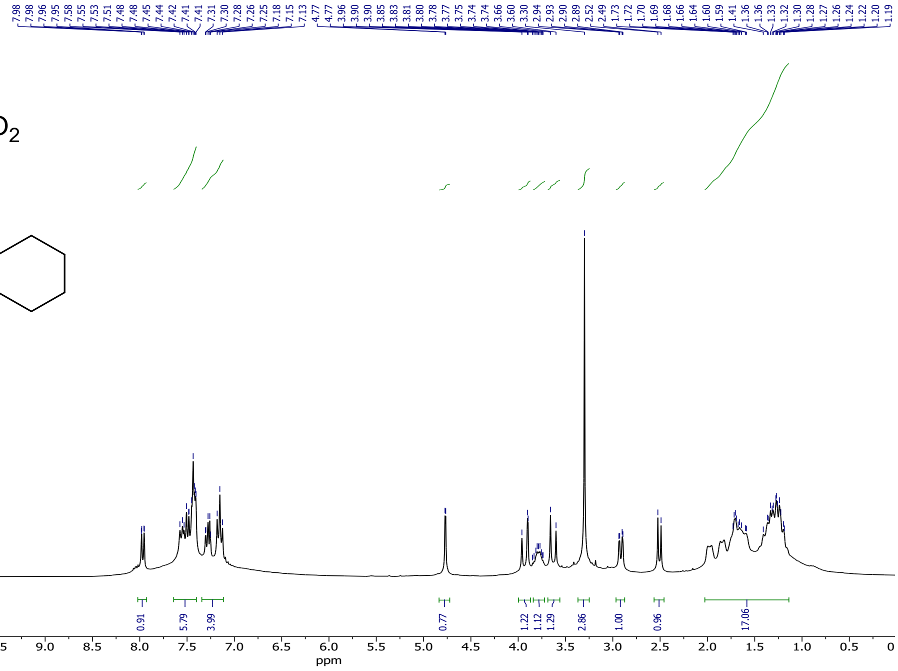

Figure S102. ^{1}H NMR spectrum of 12h (300 MHz, CDCl$_3$).

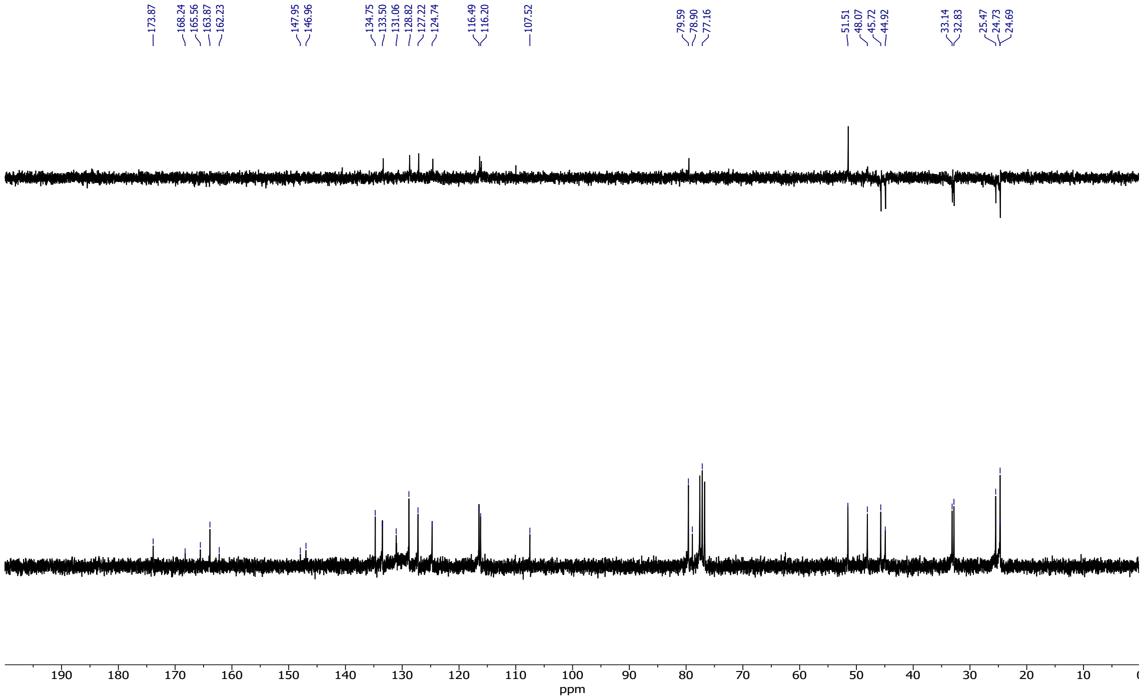

Figure S103. ^{13}C and DEPT NMR spectra of 12h (75 MHz, CDCl$_3$).

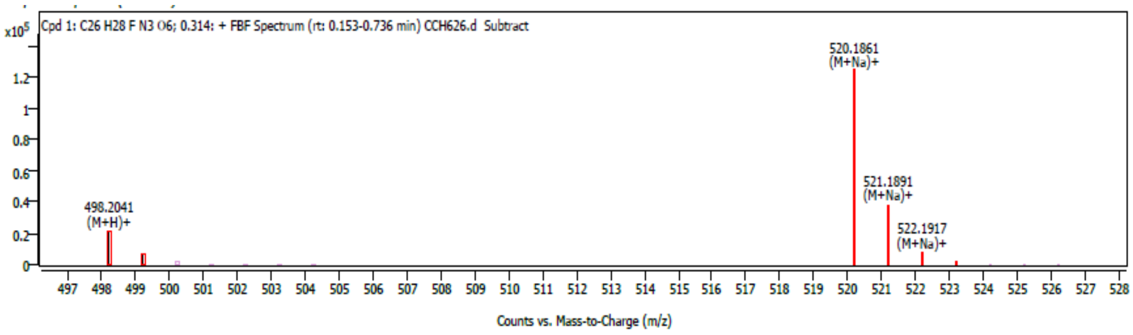

Figure S104. HRMS spectrum of 12h.

(3*R,5*R**)-5-Benzoyl-1-benzyl-5-(*N*-cyclohexylcarbamoyl)-3-hydroxy-2-pyrrolidinone (13a).**

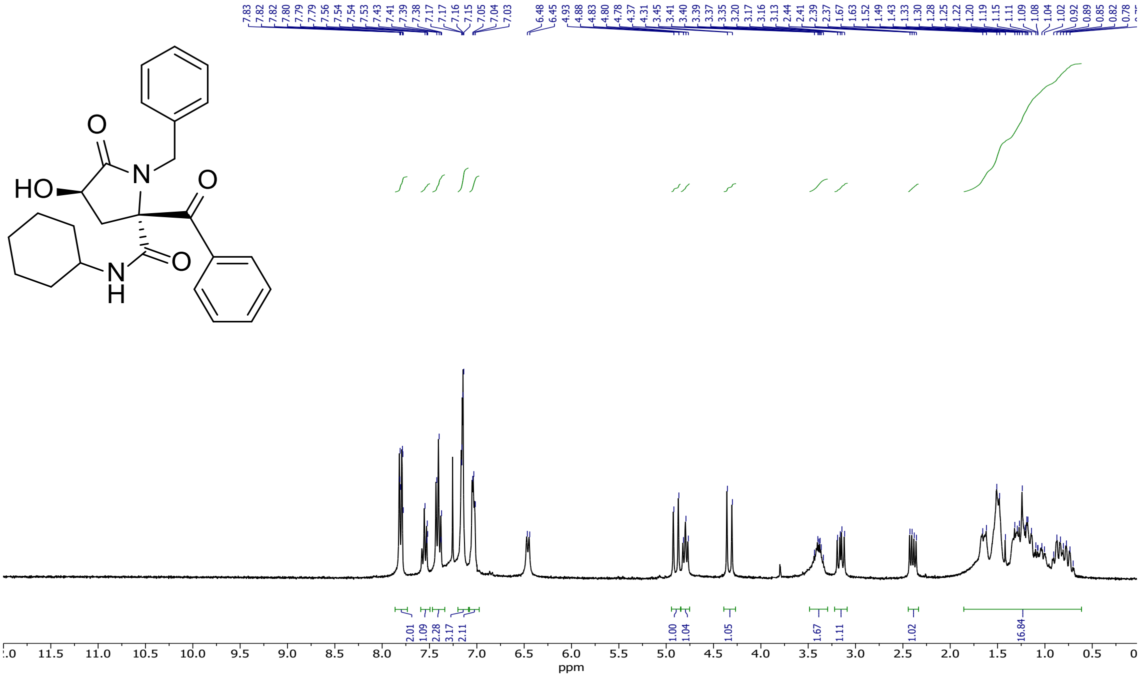

Figure S105. ^{1}H NMR spectrum of 13a (300 MHz, $CDCl_3$).

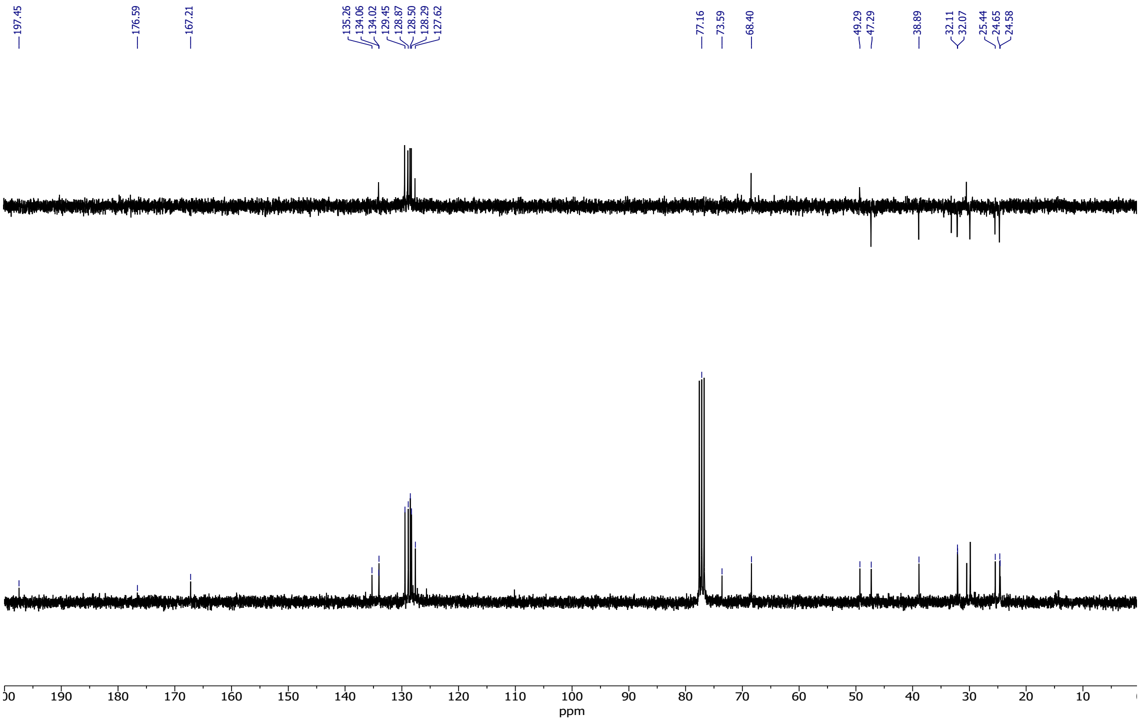

Figure S106. ^{13}C and DEPT NMR spectra of 13a (75 MHz, $CDCl_3$).

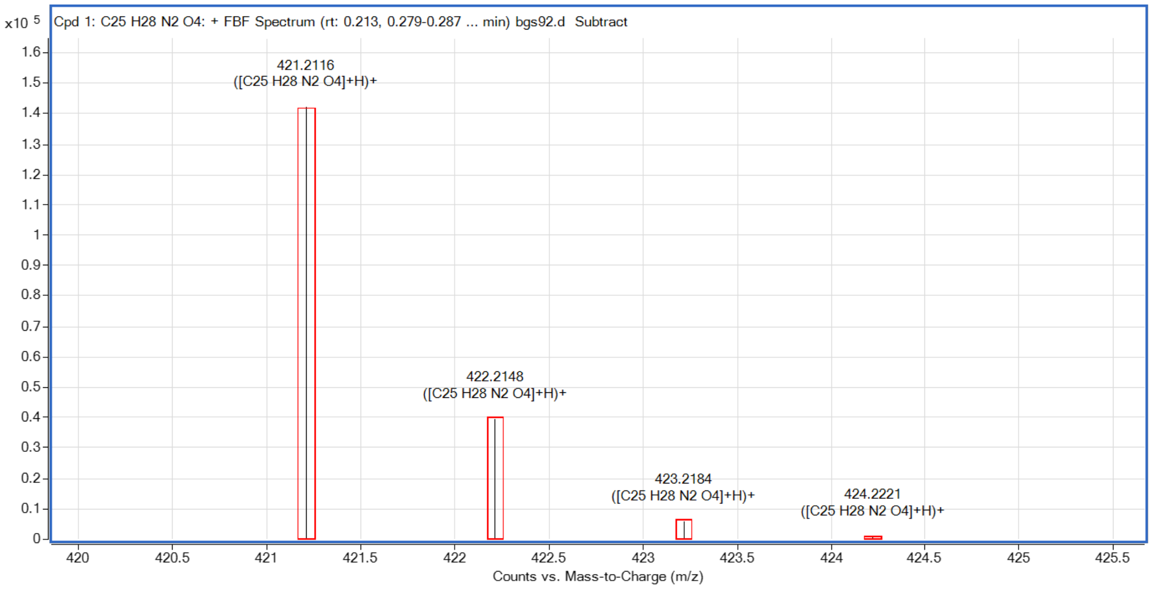

Figure S107. HRMS spectrum of 13a.

(3*R,5*R**)-1-Benzyl-5-(*N*-cyclohexylcarbamoyl)-5-(4-fluorobenzoyl)-3-hydroxy-2-pyrrolidinone (13b).**

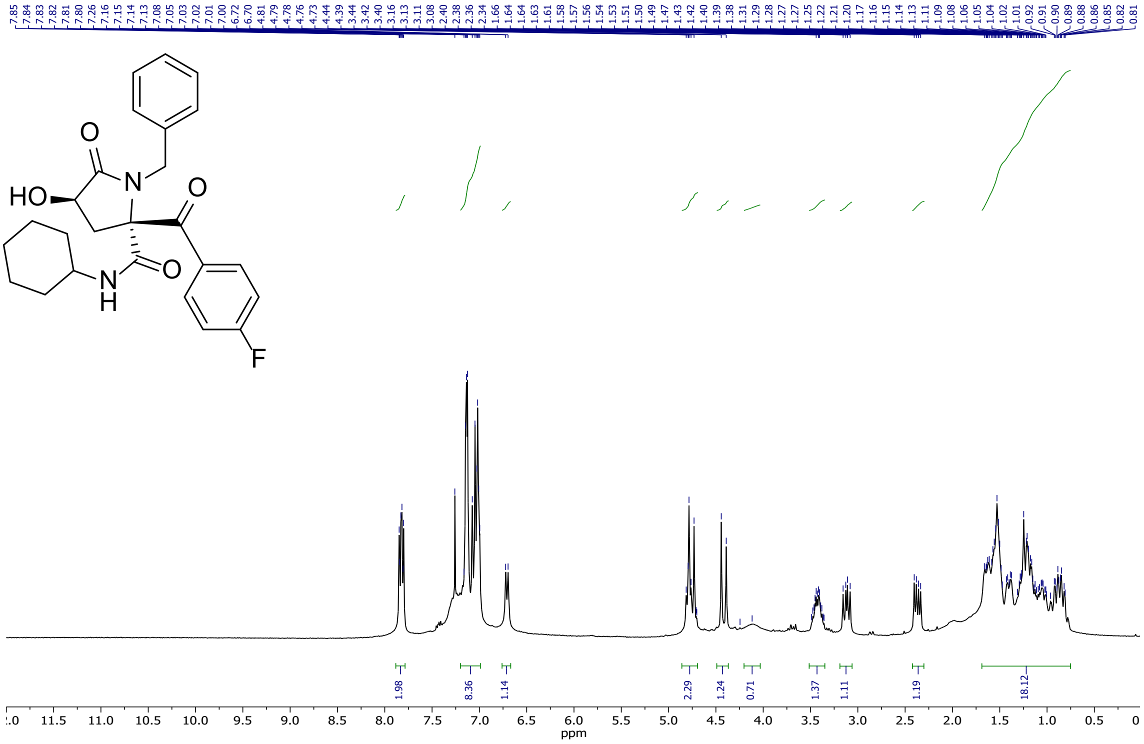

Figure S108. ^{1}**H NMR spectrum of 13b (300 MHz, CDCl$_3$).**

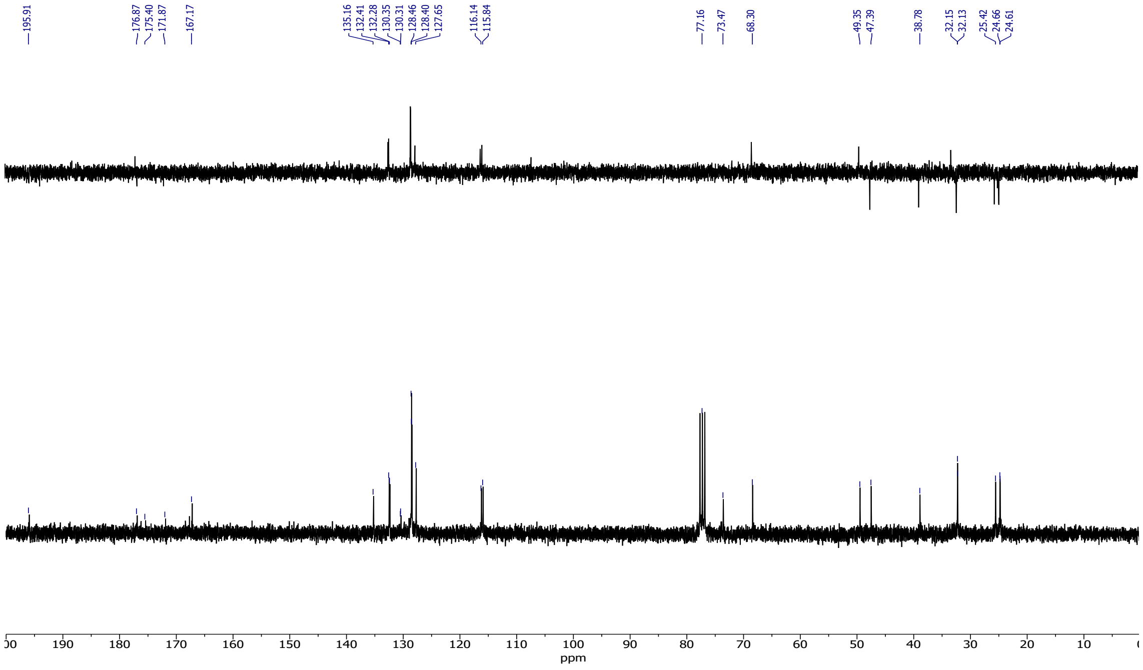

Figure S109. ^{13}**C and DEPT NMR spectra of 13b (75 MHz, CDCl$_3$).**

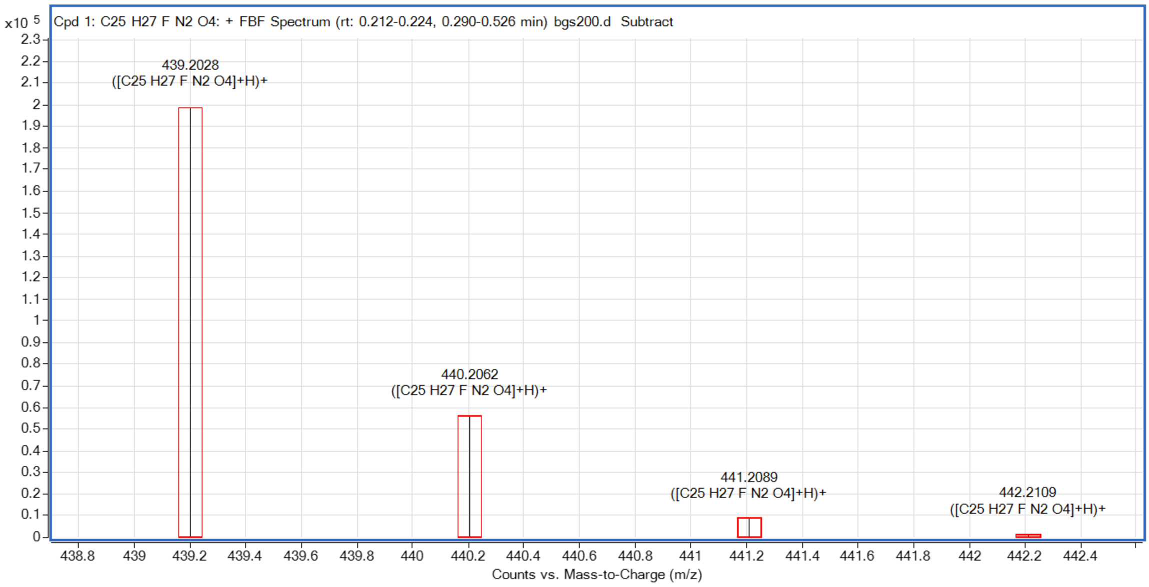

Figure S110. HRMS spectrum of 13b.

(3*R,5*R**)-5-Benzoyl-1-benzyl-5-(*N*-*tert*-butylcarbamoyl)-3-hydroxy-2-pyrrolidinone (13c).**

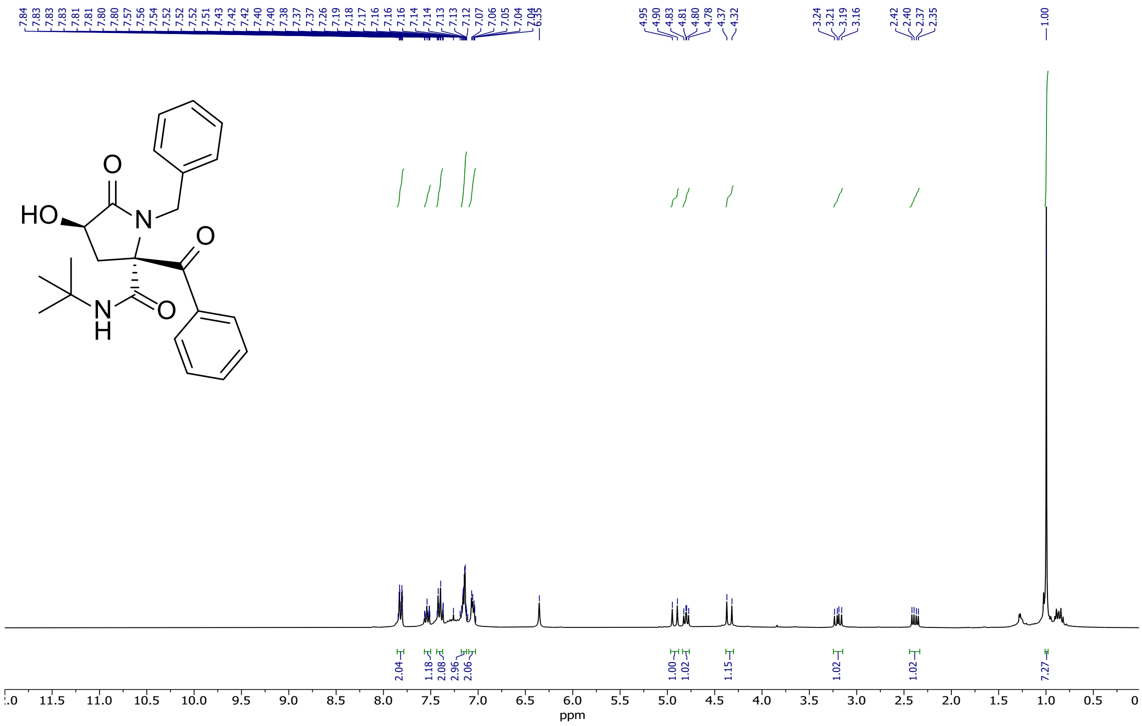

Figure S111. ^{1}H NMR spectrum of 13c (300 MHz, $CDCl_3$).

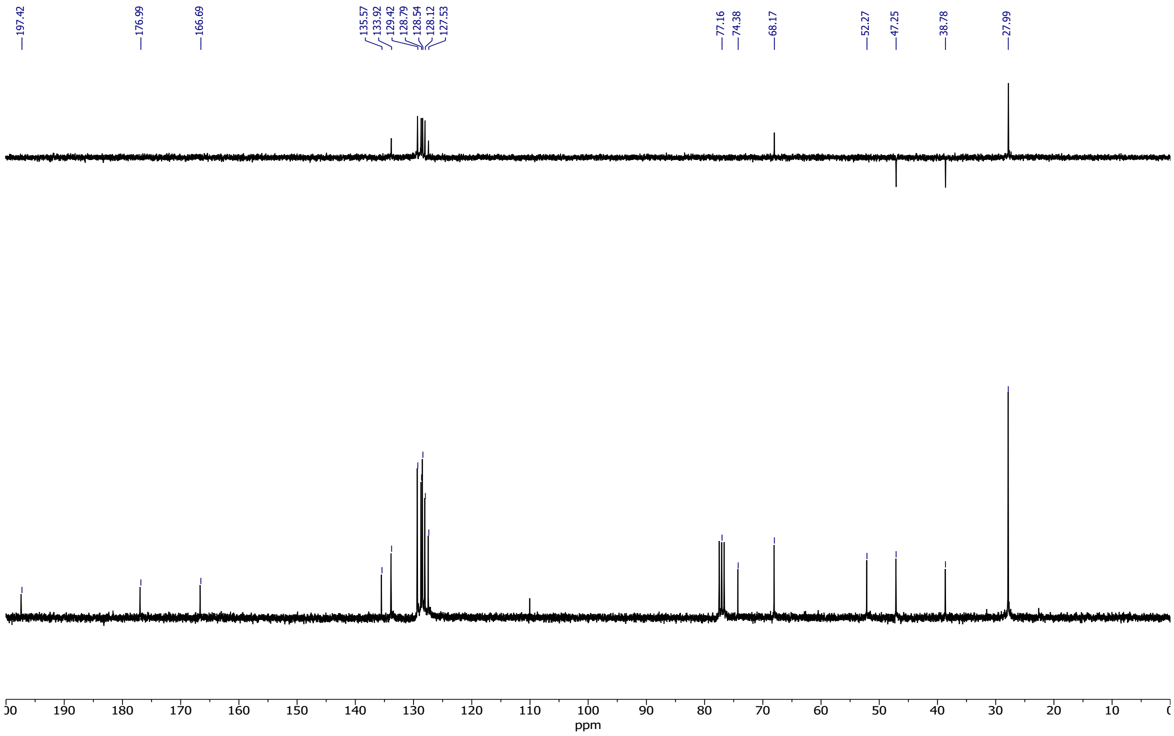

Figure S112. ^{13}C and DEPT NMR spectra of 13c (75 MHz, $CDCl_3$).

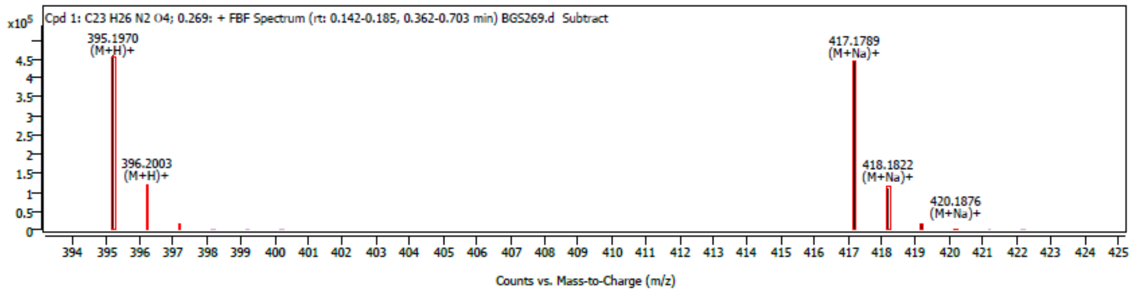

Figure S113. HRMS spectrum of 13c.

(3*R,5*R**)-5-Benzoyl-5-(*N*-cyclohexylcarbamoyl)-3-hydroxy-1-(2-nitrobenzyl)-2-pyrrolidinone (13d).**

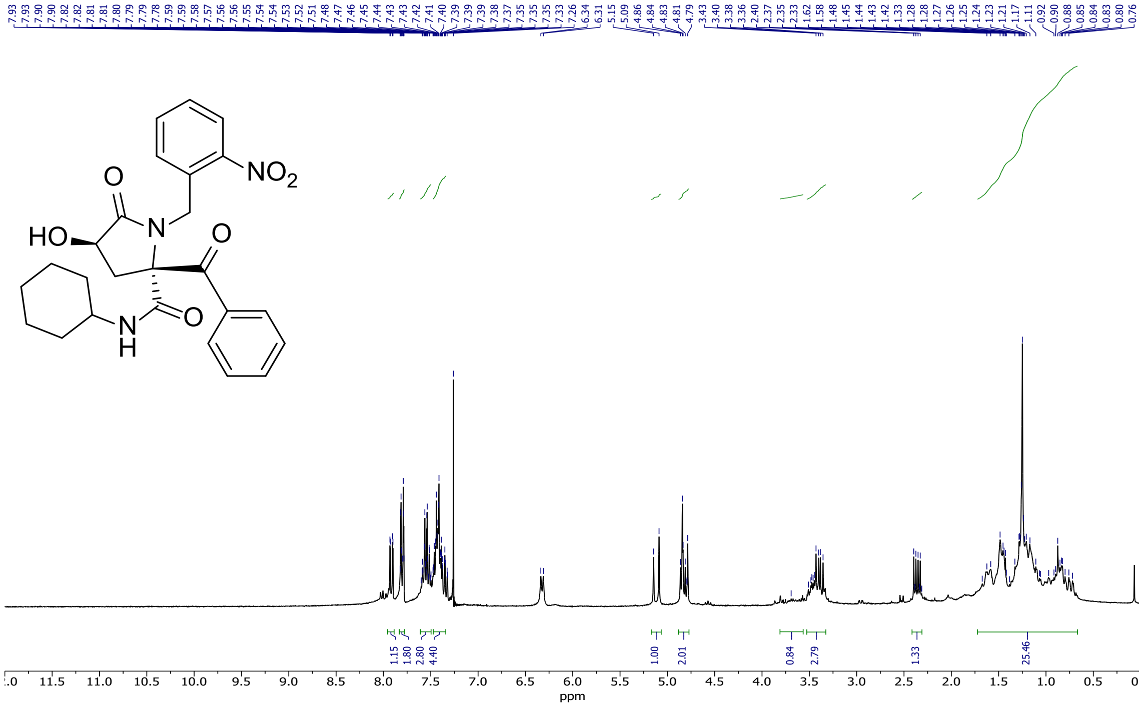

Figure S114. ^{1}H **NMR spectrum of 13d (300 MHz, $CDCl_3$).**

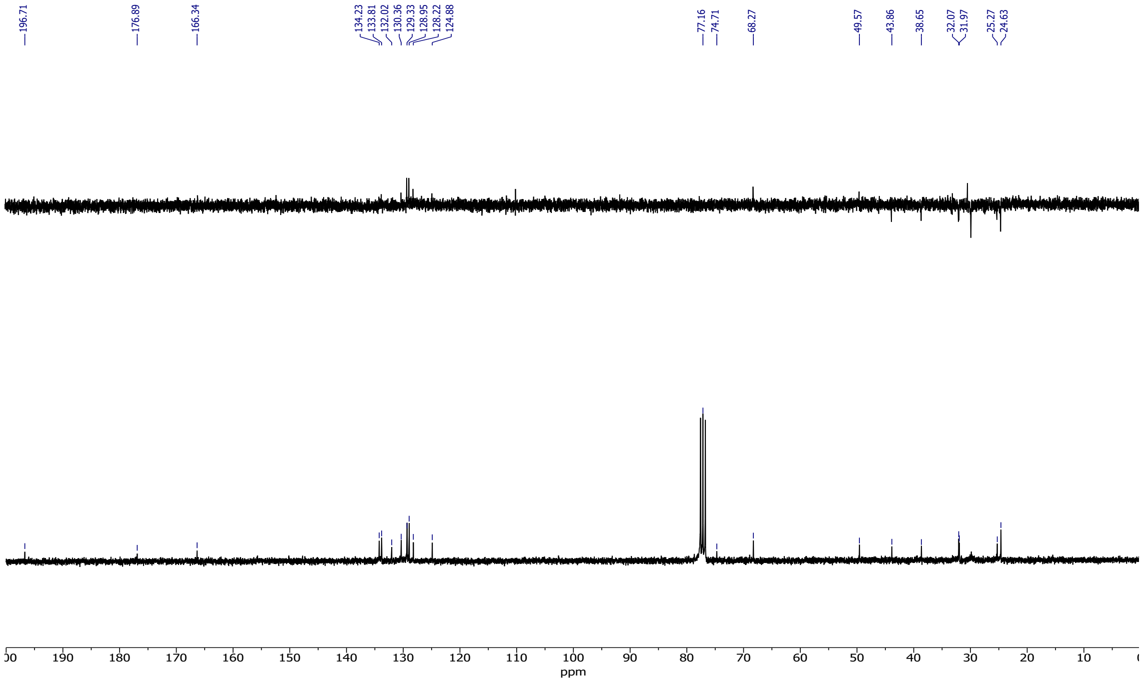

Figure S115. ^{13}C **and DEPT NMR spectra of 13d (75 MHz, $CDCl_3$).**

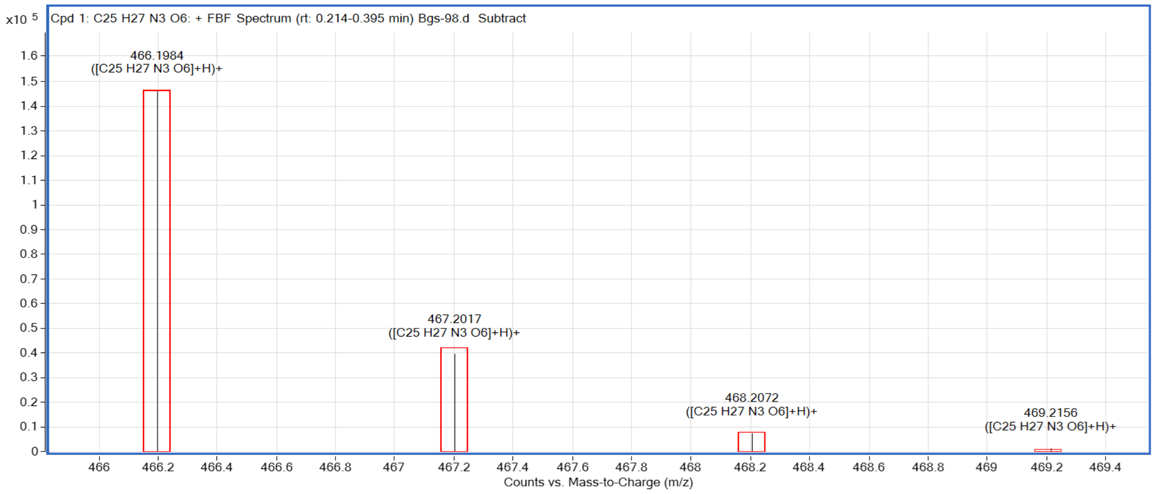

Figure S116. HRMS spectrum of 13d.

(3*R,5*R**)-5-(*N*-Cyclohexylcarbamoyl)-5-(4-fluorobenzoyl)-3-hydroxy-1-(2-nitrobenzyl)-2-pyrrolidinone (13e).**

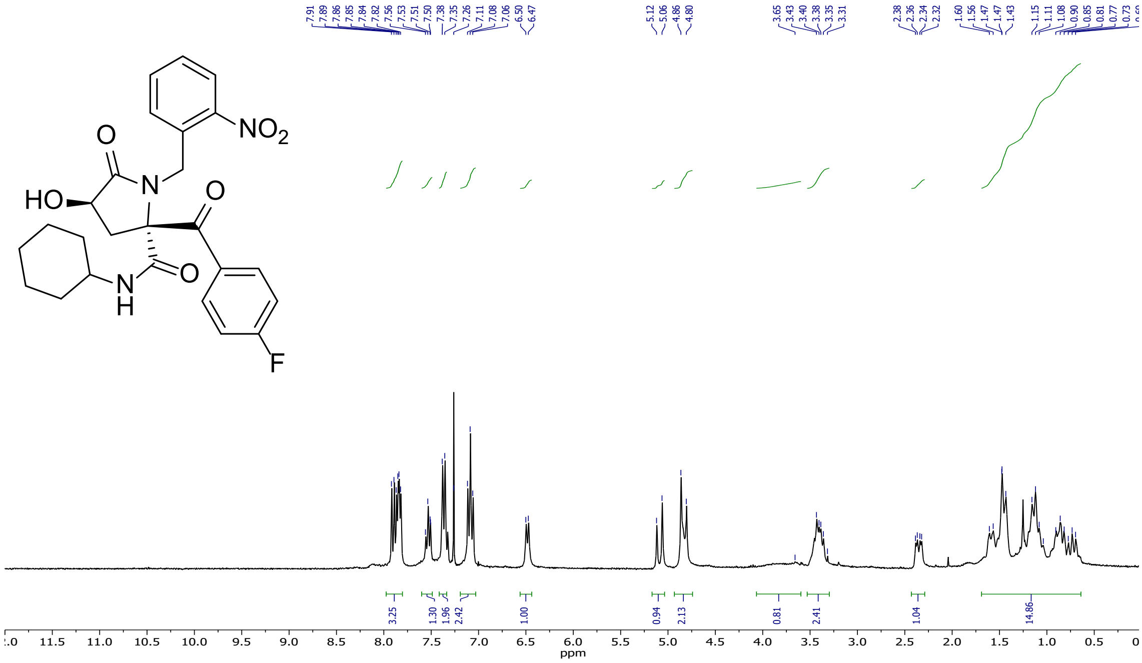

Figure S117. **^{1}H NMR spectrum of 13e (300 MHz, $CDCl_3$).**

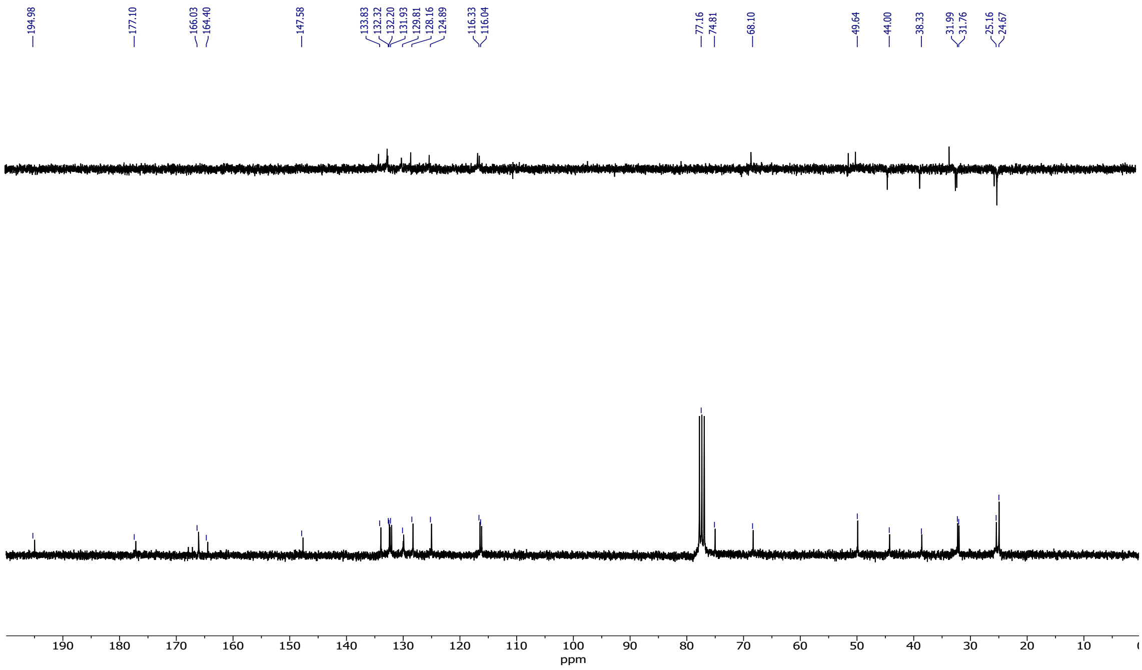

Figure S118. **^{13}C and DEPT NMR spectra of 13e (75 MHz, $CDCl_3$).**

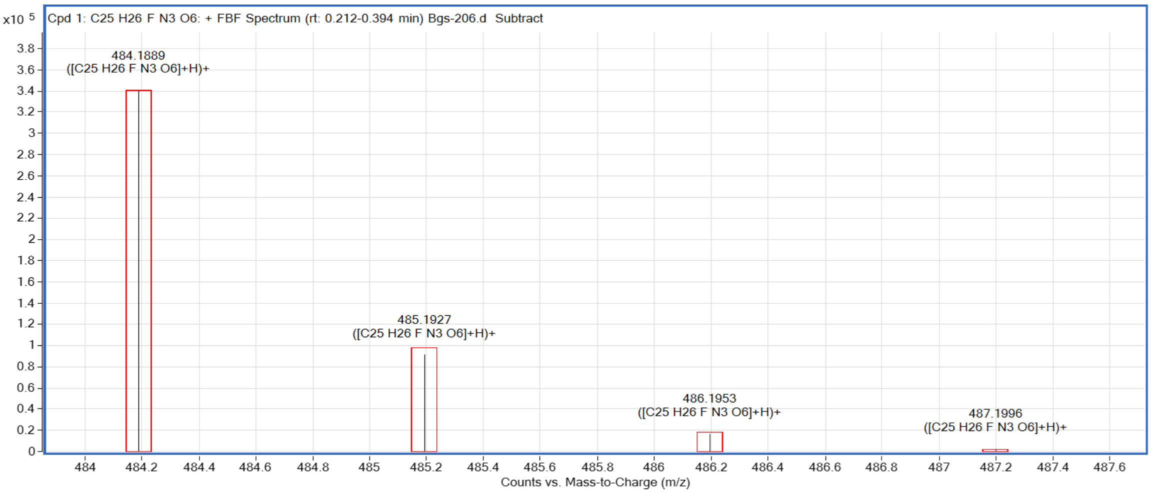

Figure S119. HRMS spectrum of 13e.

(1*R,3*R**,4*R**)-4-(*N*-Cyclohexylcarbamoyl)-3-methoxy-3-phenyl-1,3,4,6-tetrahydro-1,4-methano[1,4]oxazino[3,4-*b*]quinazoline (14a).**

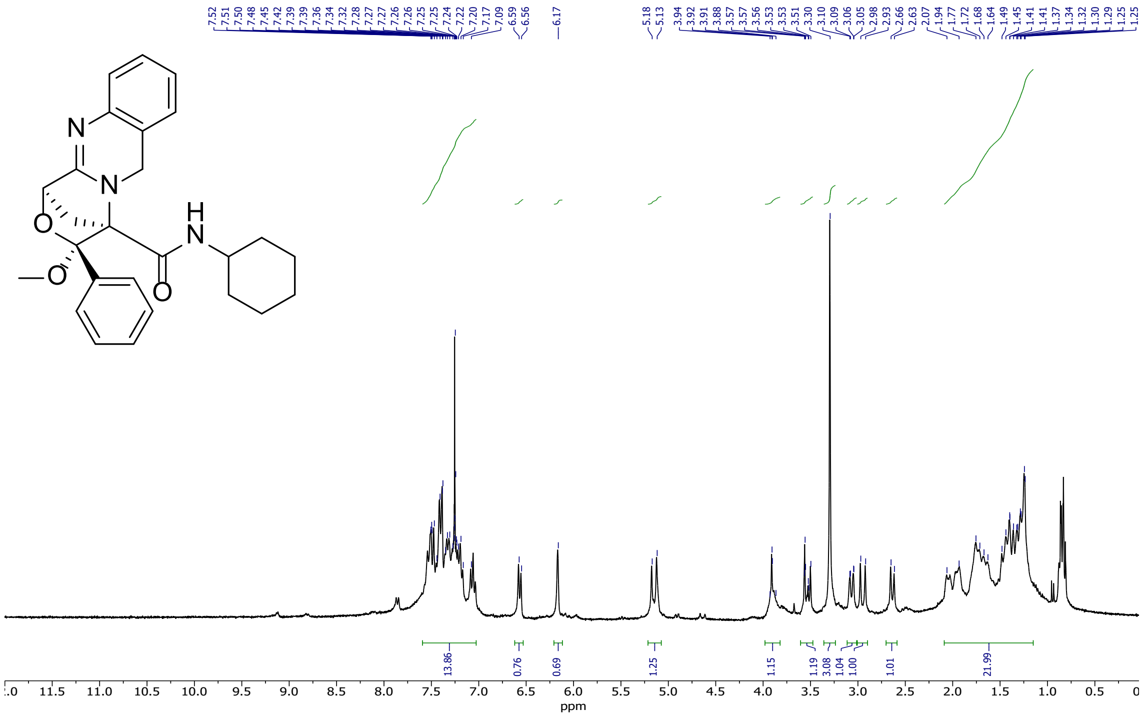

Figure S120. ^{1}H **NMR spectrum of 14a (300 MHz, $CDCl_3$).**

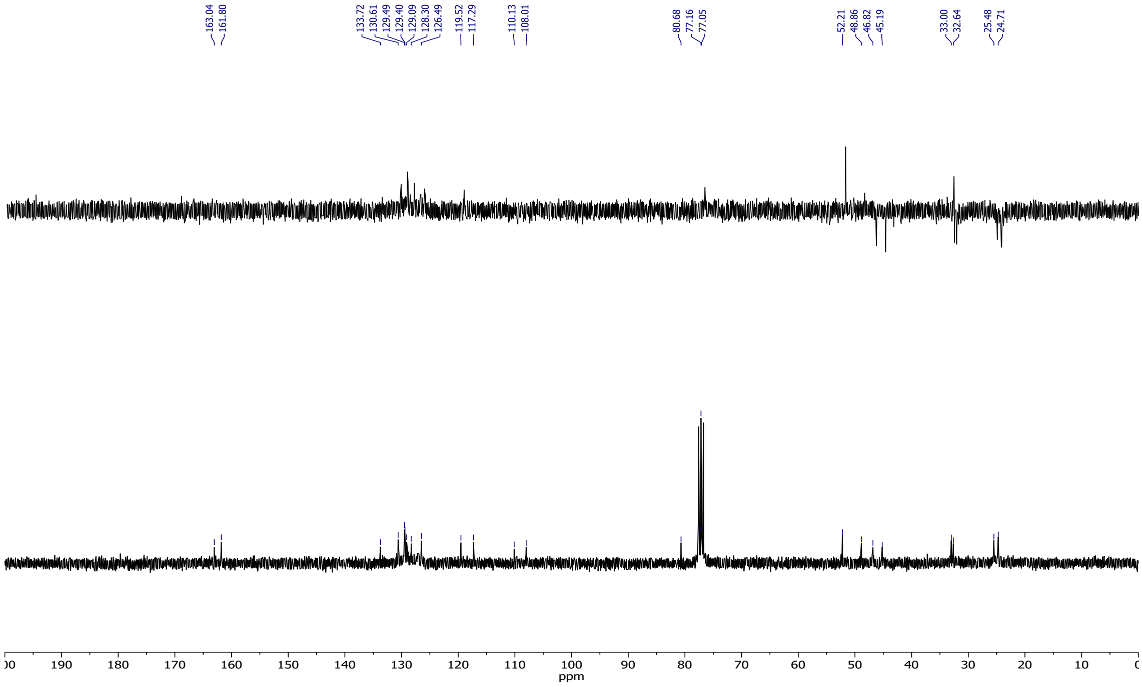

Figure S121. ^{13}C **and DEPT NMR spectra of 14a (75 MHz, $CDCl_3$).**

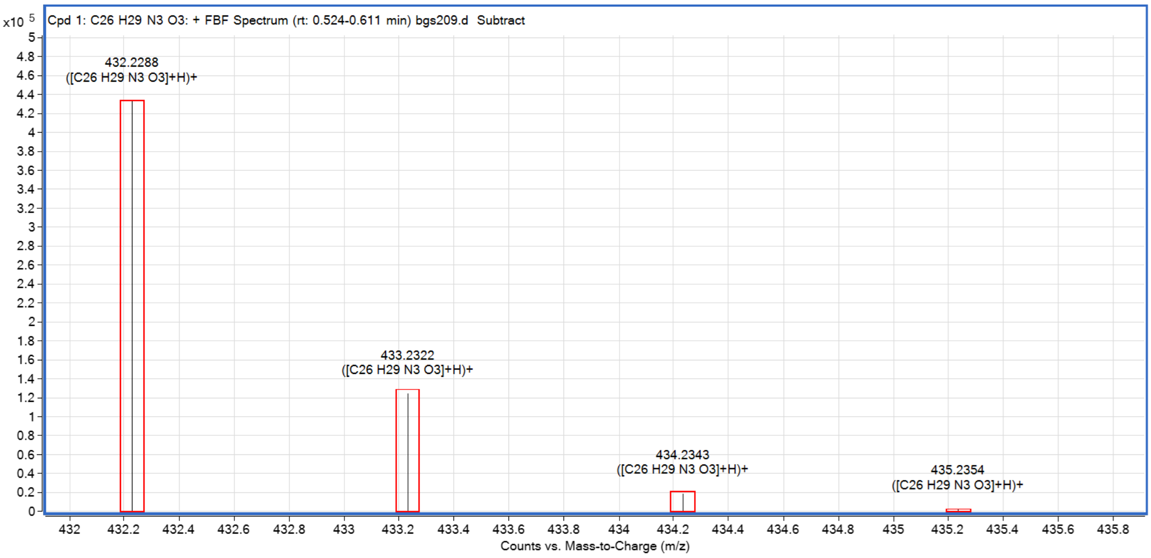

Figure S122. HRMS spectrum of 14a.

(1*R,3*R**,4*R**)-4-(*N*-Cyclohexylcarbamoyl)-3-(4-fluorophenyl)-3-methoxy-1,3,4,6-tetrahydro-1,4-methano[1,4]oxazino[3,4-*b*]quinazoline (14b).**

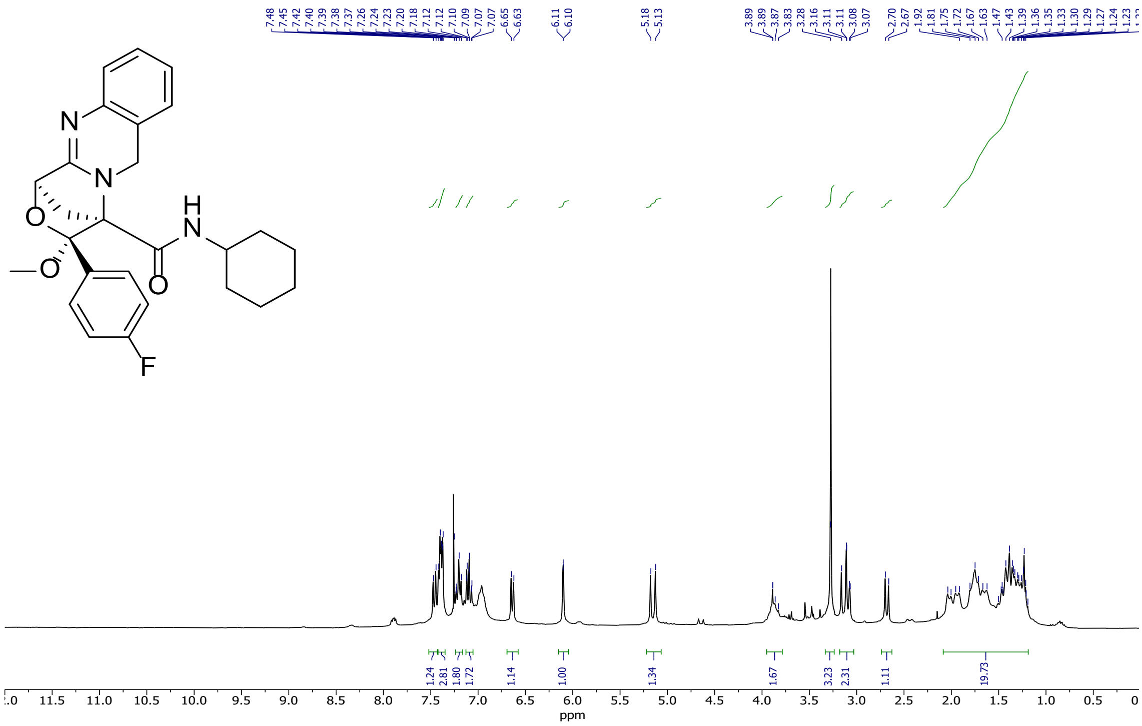

Figure S123. ^{1}H NMR spectrum of 14b (300 MHz, $CDCl_3$).

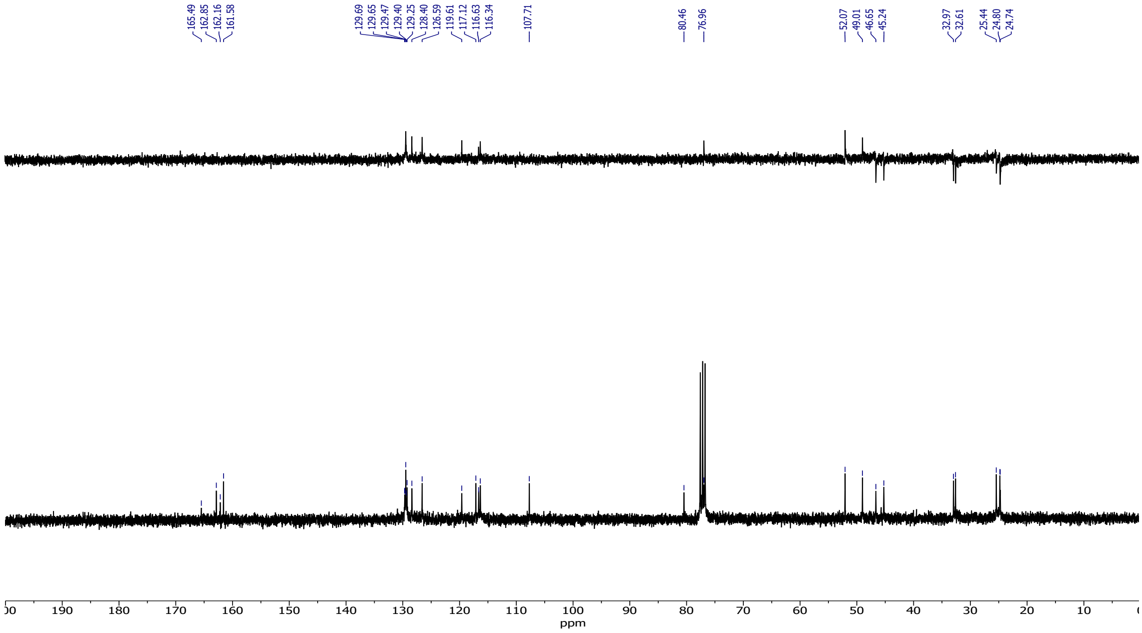

Figure S124. ^{13}C and DEPT NMR spectra of 14b (75 MHz, $CDCl_3$).

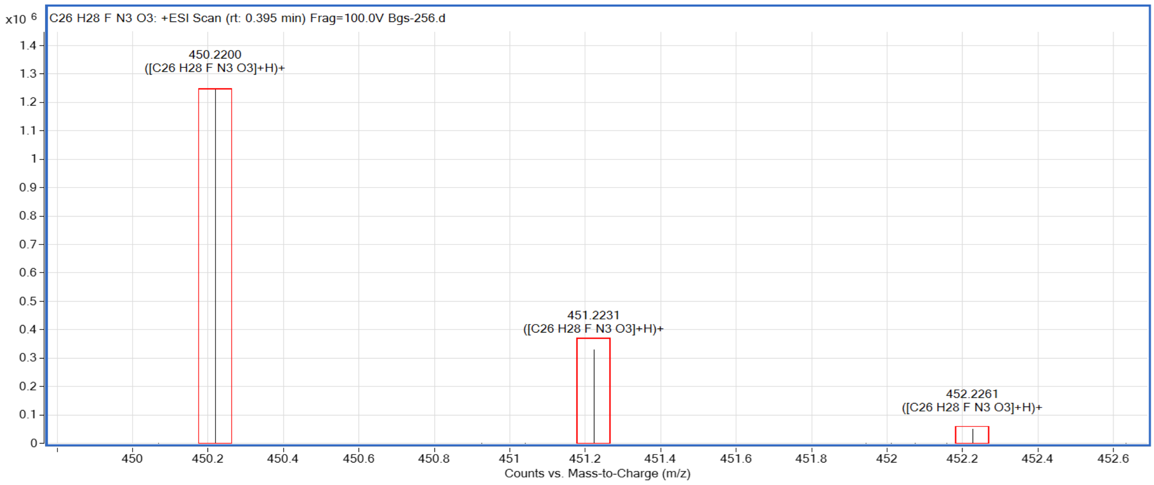

Figure S125. HRMS spectrum of 14b.

(1*R**,3*R**)-1-Benzoyl-1-(*N*-cyclohexylcarbamoyl)-3-hydroxy-1,2,3,9-tetrahydropyrrolo[2,1-*b*]quinazoline (15a).

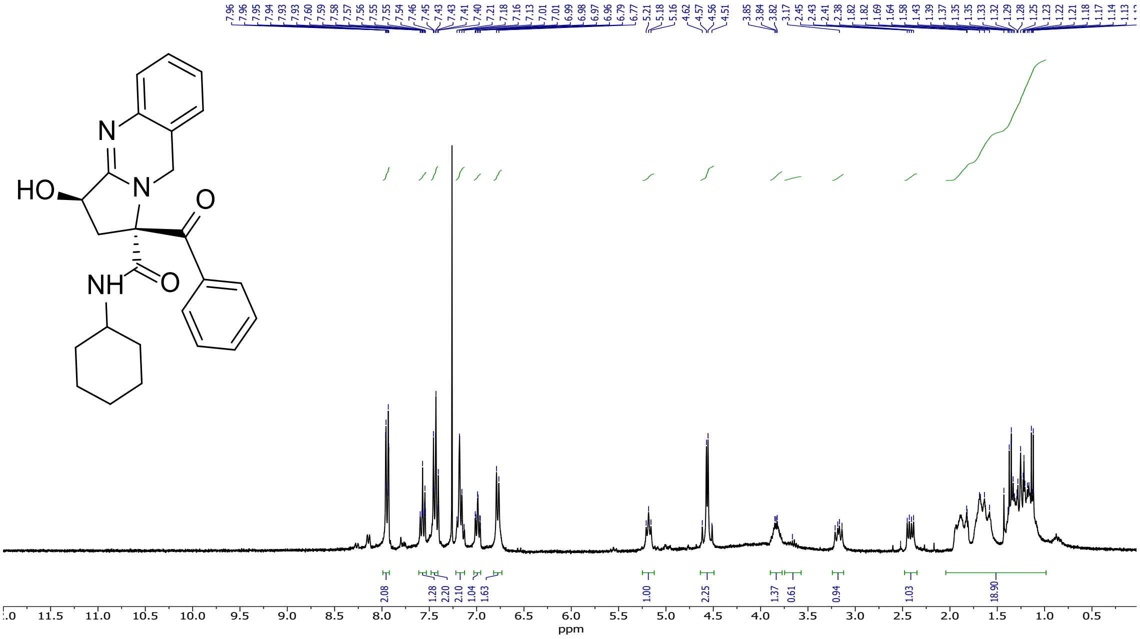

Figure S126. ^{1}H NMR spectrum of 15a (300 MHz, $CDCl_3$).

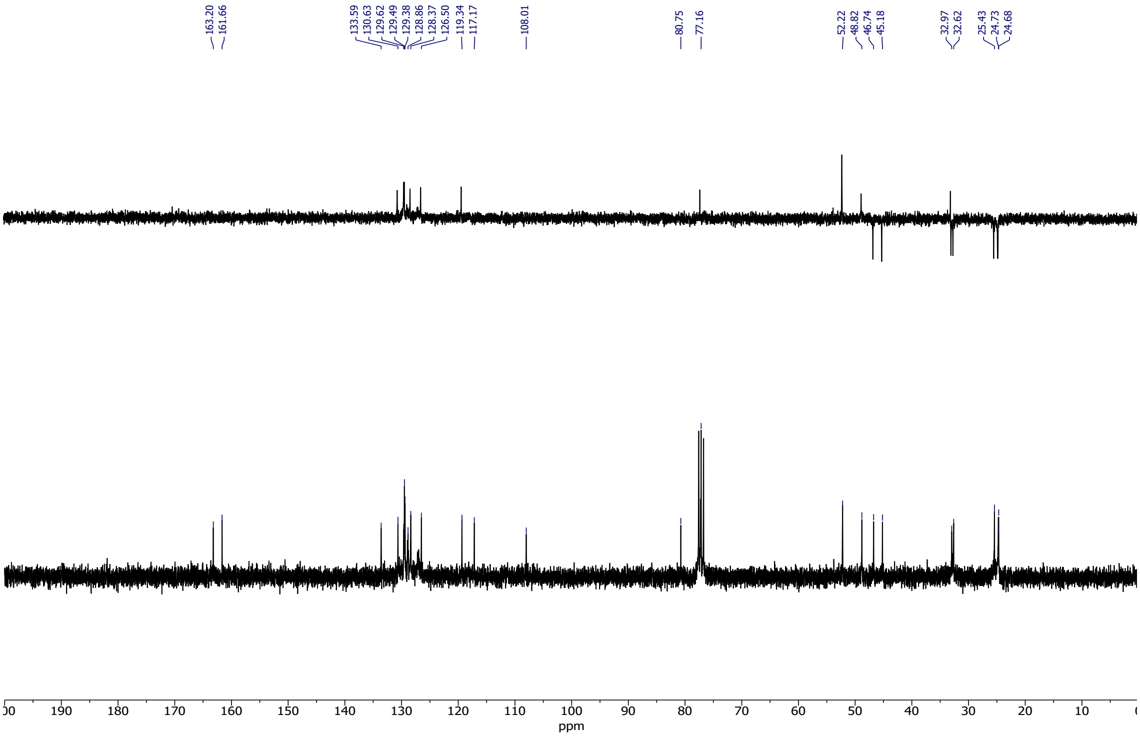

Figure S127. ^{13}C and DEPT NMR spectra of 15a (75 MHz, $CDCl_3$).

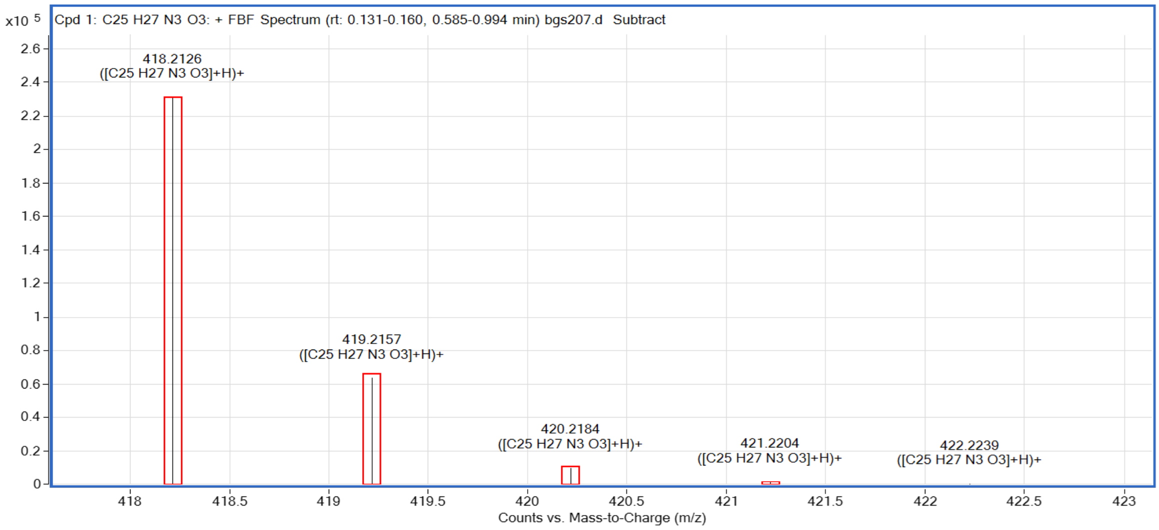

Figure S128. HRMS spectrum of 15a.

(1*R,3*R**)-1-(*N*-Cyclohexylcarbamoyl)-1-(4-fluorobenzoyl)-3-hydroxy-1,2,3,9-tetrahydropyrrolo[2,1-*b*]quinazoline (15b).**

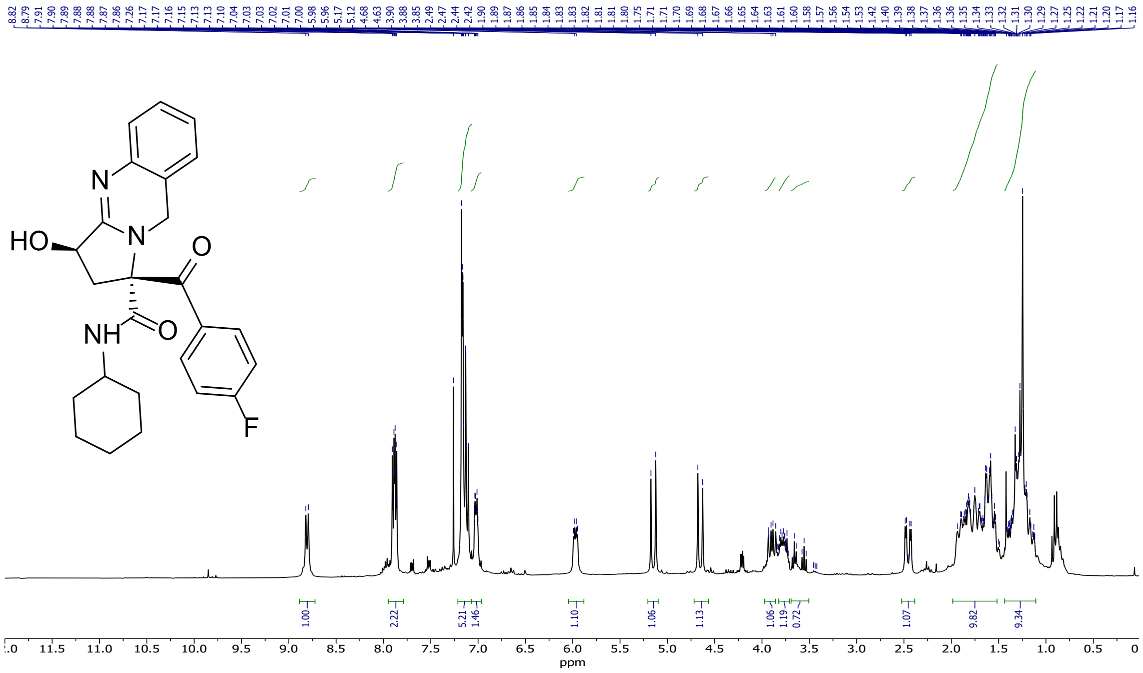

Figure S129. ^{1}H **NMR spectrum of 15b (300 MHz, CDCl$_3$).**

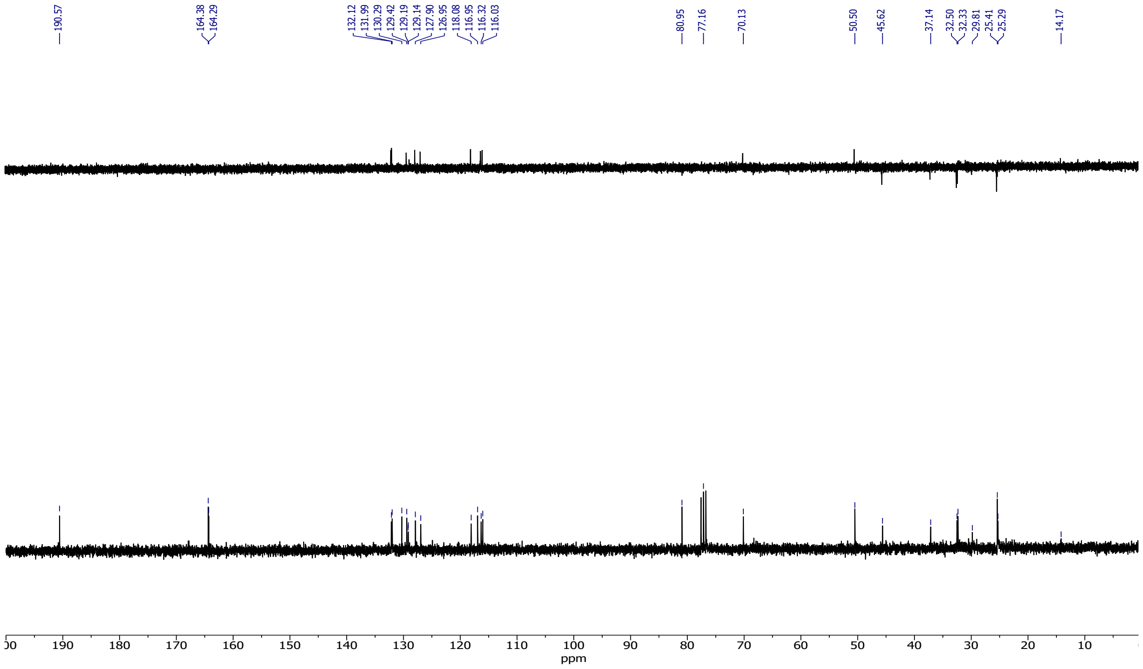

Figure S130. ^{13}C **and DEPT NMR spectra of 15b (75 MHz, CDCl$_3$).**

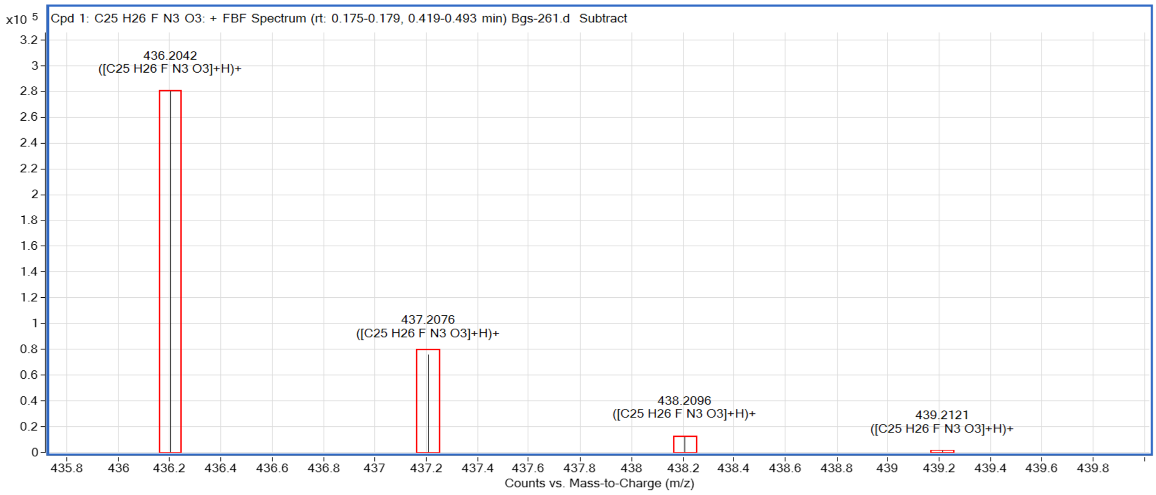

Figure S131. HRMS spectrum of 15b.

3. X-RAY DIFFRACTION STUDIES

Single crystals were grown by slow evaporation of solutions of the isolated compounds in several mixtures of solvents: methanol-butanol [(*R*)-**6b**], chloroform-hexane (**9e**), ethyl acetate-hexane (**12g**) and diethyl ether-hexane (**13a**).

Three dimensional X-ray data were collected on a Bruker D8 VENTURE diffractometer. Data were corrected for absorption effects using the multi-scan method (SADABS).[2] Complex scattering factors were taken from the SHELXL-2016[3] programme running under the Olex2[4] (**9e**, **13a**) or WinGX[5] [(*R*)-**6b**, **12g**] programmes. The structures were solved with SHELXT[6] (**9e**, **13a**), Superflip[7] [(*R*)-**6b**] or SIR92[8] (**12g**), and refined by full-matrix least-squares on F^2. All hydrogen atoms were included in calculated positions and refined in riding mode, except that of the amide's N-H fragment of (*R*)-**6b**, which was refined freely in the final stages of the refinement. EXTI correction was employed to complete the refinement of the structure of **12g**. Refinement converged with anisotropic displacement parameters for all non-hydrogen atoms. Crystal data and details on data collection and refinement are summarized in **Table S1**. The Olex2 plots of the four molecular structures are displayed in **Figures S132** to **S135**.

The X-ray molecular structures of compounds (*R*)-**6b**, **9e**, **12g** and **13a** were deposited in The Cambridge Crystallographic Data Centre (CCDC), with the following deposition numbers: 2526378 [(*R*)-**6b**], 2526379 (**9e**), 2526380 (**12g**) and 2526381 (**13a**).

Although single crystals of the *S* enantiomer of the conglomerate [(*S*)-**6b**] were also isolated and analysed by single-crystal X-ray diffraction, they were of low quality, which prevented both the deposition and publication of its structure.

---

2 SADABS: Krause, L.; Herbst-Irmer, R.; Sheldrick, G. M.; Stalke, D. *J. Appl. Cryst.* **2015**, *48*, 3-10.

3 SHELXL: Sheldrick, G. M. *Acta Cryst*. **2008**, *A64*, 112-122.

4 Olex2: Dolomanov, O. V.; Bourhis, L. J.; Gildea, R. J.; Howard, J. A. K.; Puschmann, H. *J. Appl. Cryst.* **2009**, *42*, 339-341.

5 WinGX: Farrugia, L. J. *J. Appl. Cryst.* **1999**, *32*, 837-838.

6 SHELXT: Sheldrick, G. M. *Acta Cryst.* **2015**, *A71*, 3-8.

7 SUPERFLIP: Palatinus, L.; Chapuis, G. *J. Appl. Cryst.* **2007**, *40*, 786-790.

8 SIR92: Altomare, A.; Cascarano, G.; Giacovazzo, C.; Guagliardi, A.; Burla, M. C.; Polidori, G.; Camalli, M. *J. Appl. Cryst.* **1994**, *27*, 435.

Table S1. Crystal data and refinement details for (*R*)-**6b**, **9e**, **12g** and **13a**.

	(*R*)-**6b**	**9e**	**12g**	**13a**
Empirical formula	$C_{21}H_{28}N_2O_5$	$C_{20}H_{27}FN_2O_4$	$C_{26}H_{29}N_3O_6$	$C_{25}H_{28}N_2O_4$
MW	388.45	378.43	479.52	420.49
crystal system	Triclinic	Triclinic	Monoclinic	Triclinic
space group	*P*1	*P*-1	$P2_1/n$	*P*-1
T/K	100(2)	200(2)	180(2)	100(2)
a/Å	4.8202(2)	9.1408(10)	16.559(4)	10.5040(5)
b/Å	9.5900(4)	9.7892(10)	8.5805(17)	12.6042(6)
c/Å	11.5420(5)	12.7857(14)	16.566(4)	19.4927(9)
α/deg	109.168(2)	71.885(4)	90	100.604(2)
β/deg	98.238(2)	72.983(4)	91.665(7)	92.948(2)
γ/deg	94.994(2)	70.020(4)	90	113.251(2)
V/Å^3	493.60(4)	999.42(19)	2352.9(9)	2308.54(19)
F(000)	208	404	1016	896
Z	1	2	4	4
λ, Å	1.54178	1.54178	0.71073	1.54178
D_{calc}/g cm^{-3}	1.307	1.258	1.354	1.210
μ/mm^{-1}	0.764	0.776	0.097	0.663
θ range/deg	4.93–72.25	3.72–72.07	2.46–27.58	4.09–72.32
R_{int}	0.0675	0.0689	0.1178	0.0921
reflections measured	12087	24001	31314	62932
unique reflections	3692	3886	5397	9071
reflections observed	3373	3173	3389	6507
GOF on F^2	1.050	1.046	1.061	1.020
$R1^a$	0.0435	0.0571	0.0584	0.0523
$wR2^b$	0.1135	0.1586	0.1920	0.1521
Largest ≠ peak & hole/eÅ^{-3}	0.175 and -0.232	0.234 and -0.198	0.318 and -0.350	0.245 and -0.287

^{a} $R1 = \sum||F_0| - |Fc|| / \sum|F_0|$. ^{b} w$R$2 (all data) = $\{\sum[w(||F_0|^2 - |Fc|^2)^2] / \sum[w(F_0^4)]\}^{1/2}$

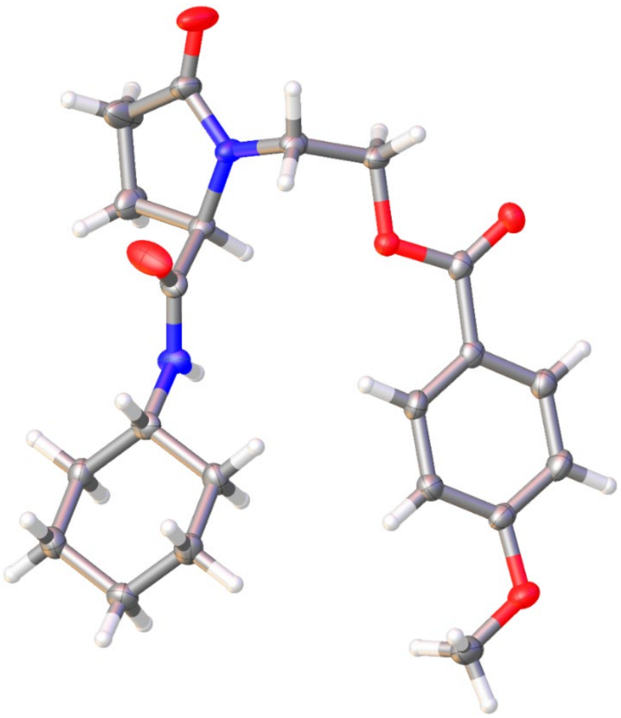

Figure S132. X-Ray molecular structure of (*R*)-6b. The Olex2 plot is at the 50 % probability level.

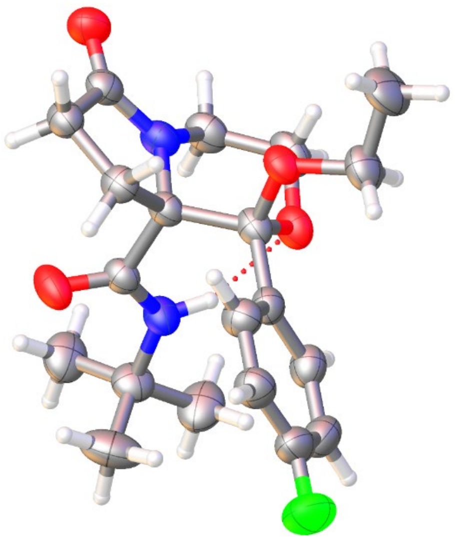

Figure S133. X-Ray molecular structure of 9e. The Olex2 plot is at the 50 % probability level. The red dotted line represents an intramolecular hydrogen bond.

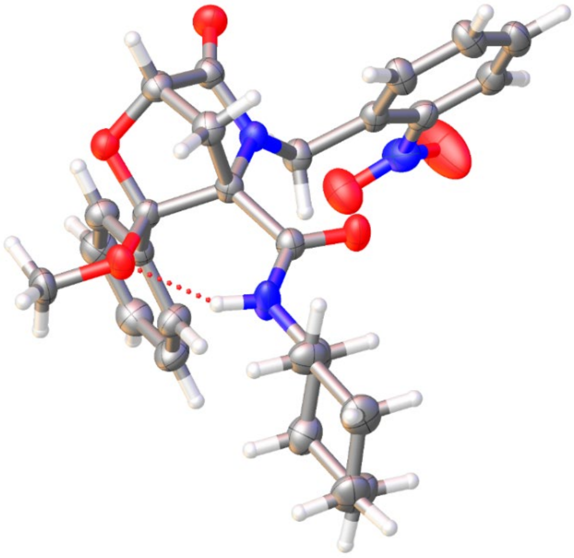

Figure S134. X-Ray molecular structure of 12g. The Olex2 plot is at the 50 % probability level. The red dotted line represents an intramolecular hydrogen bond.

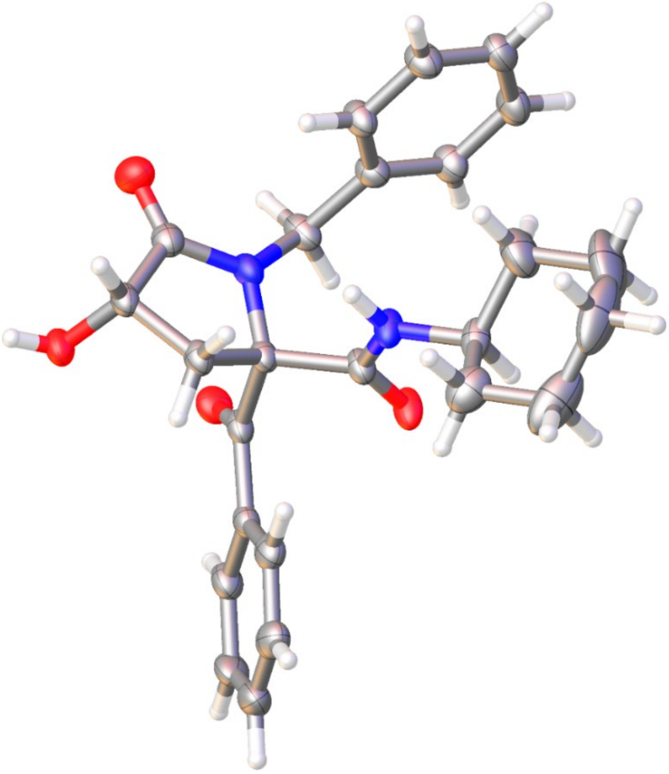

Figure S135. X-Ray molecular structure of 13a. The Olex2 plot is at the 50 % probability level. Only one of the two molecules comprising the asymmetric unit is shown.
